# Room temperature synthesis of perylene diimides facilitated by high amic acid solubility†

**DOI:** 10.1039/d1qo01723c

**Published:** 2022-01-10

**Authors:** Markus C. Kwakernaak, Marijn Koel, Peter J. L. van den Berg, Erik M. Kelder, Wolter F. Jager

**Affiliations:** Department of Chemical Engineering, Delft University of Technology Van der Maasweg 9 2629 HZ Delft The Netherlands W.F.Jager@tudelft.nl; Department of Radiation Science and Technology/Reactor Institute Delft, Delft University of Technology 2629 JB Delft The Netherlands

## Abstract

A novel protocol for the synthesis of perylene diimides (PDIs), by reacting perylene dianhydride (PDA) with aliphatic amines is reported. Full conversions were obtained at temperatures between 20 and 60 °C, using DBU as the base in DMF or DMSO. A “green” synthesis of PDIs, that runs at higher temperatures, was developed using K_2_CO_3_ in DMSO. The reaction sequence for the imidization process, *via* perylene amic acid intermediates (PAAs), has been confirmed experimentally aided by the synthesis and full characterization of stable model amic acid salts and amic esters. Kinetic studies, using absorption spectroscopy, have established that PDI formation proceeds *via* fast amic acid formation, followed by a slow conversion to imides. Solubility of the intermediate PAA salts is found to be low and rate-limiting. Based on this finding, quantitative PDI synthesis at room temperature was achieved by diluting the reaction mixture with water, the solvent in which PAA salts have better solubility. Thus, the otherwise harsh synthesis of PDIs has been transformed into an extremely convenient functional group tolerant and highly efficient reaction that runs at room temperature.

## Abstract 2

Perylene diimides (PDIs) are synthesised at room temperature and obtained in quantitative yields after a single filtration. High solubility of the intermediate amic acid salts 5 and 9 is key to the success of this novel synthesis.

## Graphical abstract

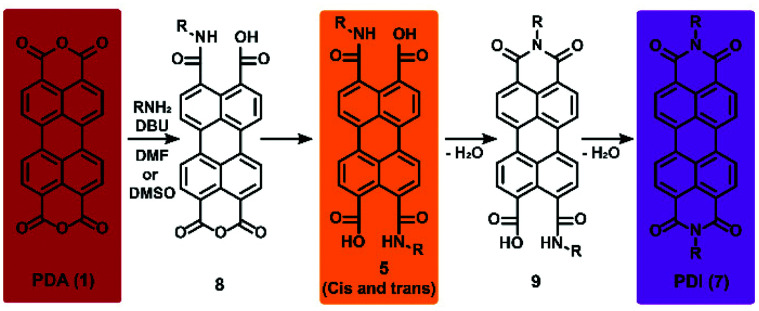

## Introduction

Perylene-3,4,9,10-tetracarboxylic acid derivatives (PTCAs) form a class of organic dyes and pigments, from which perylene-3,4,9,10-tetracarboxylic diimides (PDIs) are the most abundantly used representatives.^1^ These compounds have very benign optical properties, which has resulted in large scale application of PDIs as dyes and pigments.^2^ Moreover, PDIs are well-suited for utilization as functional materials, mostly in the optoelectronic domain.^3^ Current research efforts are focussed on the use of PDIs as non-fullerene acceptors in photovoltaics,^4^ light harvesting antenna molecules,^5^ electrode materials in batteries,^6^ fluorescent probes^7^ and photocatalysts for the dehalogenation of aromatic compounds.^8^

Despite this long history and intense interest by scientists from a wide range of disciplines, the synthesis of PDIs is generally performed under harsh conditions, with limited functional group tolerance.^9^ PDIs are routinely synthesised by the “Langhals method”, reacting perylene-3,4,9,10-tetracarcoxylic dianhydride (PDA) with primary amines in molten imidazole at 140–180 °C, using zinc acetate as a catalyst.^10^ This method has been the gold standard for decades and has proven itself to be very reliable and universally applicable. Nevertheless, a milder, greener and more user-friendly synthesis would be desirable, in order to make more PDIs accessible to a larger scientific audience.

The harsh conditions in the “Langhals method” appear to originate from either the low reactivity of the amic acid intermediates, the low solubility of starting compounds and intermediates, or both. The more soluble bay-halogenated PDAs generally undergo imidization reactions at milder conditions, typically using organic acids as catalyst and (co)solvent,^11^ while the imidization of 4-substituted naphthyl anhydrides proceeds at even milder conditions,^12^ typically in refluxing ethanol. These observations suggest that solubility is the major driver for applying harsh reaction conditions in the current PDI syntheses.

Greener methods for PDI synthesis have been reported recently, notably a solvent-less method using a twin screw extruder^13^ and a method in which water at high temperatures and elevated pressure is used.^14^ Both methods work particularly well for aliphatic amines and are definitely green(er). Still these reactions require high temperatures, which limits functional group tolerance. Interestingly, when using water at milder temperatures, around 60 °C, PDIs are not formed in significant quantities. Instead, perylene-3,4-dicarboxylic acid monoanhydride-9,10-dicarboxylic acid monoaimides (PMAMIs) are the major reaction products.^15^ Most likely, anhydride ring opening by hydroxide ions,^16^ resulting in the formation of adjacent dicarboxylates on the perylene scaffold is responsible for the observed PMAMI formation.^17–19^ It should be mentioned nevertheless, that partial hydrolysis of PDA to perylene-3,4-dicarboxylic monoanhydride-9,10-dicarboxyates, followed by a selective imidization at the anhydride position is a highly beneficial reaction that has been exploited for efficient syntheses of aliphatic PMAMIs and *N*-desymmetrized PDIs.^20^

**Scheme 1:**
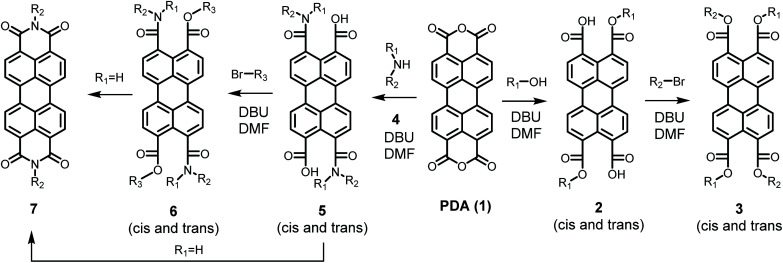
Room temperature synthesis of PTEs (3) PDAAs (5), PDAEs (6) and PDIs (7).

In contrast to imidization, esterification of PDA has been achieved at mild reaction conditions,^21^ even at room temperature,^22^ see Scheme 1. In the first step of this reaction an alcohol opens the anhydride in the presence of the strong base DBU. The perylene-3,9-dicarboxylic acid-4,10-dicarboxylic dialkyl esters 2 resulting from the first reaction step are highly soluble and subsequently react with an alkyl halide in a slower rate-determining step to produce perylene-3,4,9,10-tetracarboxylic tetra-alkyl esters (PTEs 3) in almost quantitative yields. Interestingly, in a variation of this reaction, the synthesis of an imide at room temperature, postulated to proceed *via* an amic acid, has been reported. Although the reported synthetic procedure, exploiting a 3-day aminolysis reaction, may not be the most practical one, this finding suggests that PDI synthesis at mild reaction conditions is a realistic option.^22^

Additional information suggesting that PDI synthesis at low temperatures is feasible, comes from mechanistic studies on naphthalene-1,8-dicarboxylic amic acid (NMAA)^23^ and naphthalene-1,3,4,8-tetracarboxylic diamic acid (NDAA).^24^ NMAA has been synthesised by a reaction of naphthalene-1,8-dicarboxylic anhydride (NMA) with butyl amine at low temperatures.^25^ At high pH values in water, NMAA forms the corresponding imide, naphthalene-1,8-dicarboxylic imide (NMI), while at low pH values the anhydride NMA is formed. These reactions are reversible and already proceeds at appreciable rates at temperatures as low as 30 °C. These studies suggest that formation of PDIs from PDA *via* corresponding amic acids may take place at low temperatures and high pH values, provided that a suitable polar solvent is identified. This may not be a trivial event in the light of the low solubility of most PTCA derivatives. Also it should be mentioned that the chemistry of PTCAs may differ from those of the corresponding naphthalene derivatives in other aspects than solubility. For example perylene-3,4,9,10-tetracarboxilic acid chloride has never been prepared^10*b*^ whereas the analogous naphthyl-1,8-di^26^ and 1,3,4,8-tetracid chlorides^27^ are readily available by conventional means.

In this work we report the synthesis of PDIs by reacting PDA with primary aliphatic amines at low temperatures, in a manner analogous to the synthesis of PTEs depicted in Scheme 1. Reaction conditions for the imidization of PDA under “standard” conditions, with DBU as the base in the solvent DMF, were optimized. In addition an entirely Green synthesis of PDIs, using DMSO and K_2_CO_3_, was developed. To identify the reactive intermediates formed during the imidization reaction, stable model amic acids have been synthesised by the reaction of PDA with secondary amines. The (photo)physical properties of these compounds were determined and their spectra were compared with those of the reaction intermediates to support the proposed mechanism of imide formation. A kinetic model of the imidization reactions has been constructed and tested by monitoring imidization reactions in real time. Based on the mechanistic insights obtained from these experiments the reaction conditions were optimized further to achieve quantitative imide formation at room temperature.

## Results and discussion

### General reaction and proposed reaction mechanism

When secondary amines were reacted with PDA 1, using the synthetic protocol for the synthesis of PTEs, perylene-3,4,9,10-tetracarboxylic-diamic esters PDAE 6 were formed *via* perylene-3,4,9,10-tetracarboxylic-diamic acid PDAA 5, see Scheme 1. Both compounds have been isolated and fully characterized, *vide infra*. When primary amines were employed, using the same procedure, however, PDIs 7 were obtained in appreciable yields. The fact that PDIs are formed is not surprising as such, since amic acids and amic esters are known to react further to form imides. The observation that this reaction takes place at room temperature already was surprising to us and clearly indicates that PDI synthesis can be performed at very mild conditions.

Next, it is worthwhile to establish whether alkylation of amic acids, *i.e.* amic ester formation, is a prerequisite for efficient imide formation. Reactions from amic esters to imides have been reported in the context of polyimide formation. This work, however, involved the synthesis of five-membered phthalimides and these imidization reaction have been performed by “brute force”. To the best of our knowledge, neither the temperature nor the solvent dependence of such reactions have been explored.^28^ In another work the alkylation of a perylene-3,4-monoamic acid has been reported. This reaction resulted in a double alkylation at the carboxylate and the amide nitrogen,^29^ but imide formation was not reported.

Whether the imide formation using our original procedure proceeds *via* the amic acid 5, the amic ester 6, or possibly both has been investigated by reacting 1 with 2-ethyl-hexyl amine 4b, in the presence and in the absence of butyl bromide. For both reactions PDI 7b was isolated, in yields of 60% and 25%, respectively, see Table 2 entries 3 and 4. This outcome demonstrated convincingly that addition of an alkylating reagent significantly increased the yield of PDI 7b, and thereby proofs that imidization *via* PDAE 6 is the faster route to form PDIs.^30^

Although significantly higher yields have been obtained by adding an alkylating reagent, we decided to further develop mild imidization reactions without the use of alkylating reagents. This choice has been made because alkylation requires an extra reaction step, uses a highly reactive alkylating reagent and thereby severely limits functional group tolerance. As for the reaction without an alkylating reagent, the imide formation itself already has a conversion around 50%,^31^ and that is a good starting point for obtaining satisfactory yields after optimizing the reaction conditions. It should be noted that the use of alkylating reagents for highly unreactive systems, such as electron deficient or sterically hindered amines, is still a viable option.

**Scheme 2:**
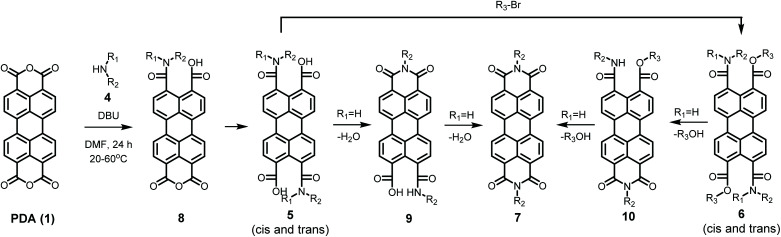
Imidization reaction starting from PDA (1) including all anticipated intermediates.

**Scheme 3:**
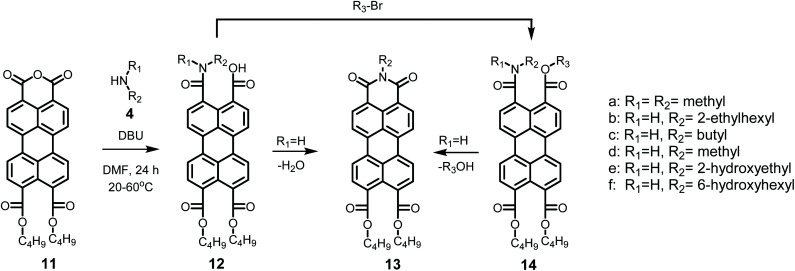
Imidization reaction starting from PMADE (11) including all anticipated intermediates.

The detailed reaction schemes for the imidization reactions, starting from PDA 1 or model compound perylene-3,4-dicardoxylic anhydride-9,10-dicarboxylic dibutyl ester PMADE 11,^32^ are depicted in Schemes 2 and 3. The imidization reaction of PMADE 11 is included in this work, because this reaction proceeds *via* a single amic acid intermediate compound 12, and yields reaction products, perylene-3,4-dicarboxylic monoimide-9,10-dicarboxylic diesters (PMIDEs 13), that are soluble in common organic solvents. The more complex imidization of PDA (1) proceeds through four intermediates; compound 8, *cis*- and *trans*-5 and compound 9. In addition, the resulting PDIs 7 generally are not soluble in organic solvents, which prevents easy identification of the reaction product(s).

### Synthesis and characterization of model amic acids

In order to validate the reaction mechanism depicted in Schemes 2 and 3, the potassium salts of the amic acids 12a, 5a and 9a were synthesised and characterised. These compounds are model compounds for the intermediate amic acid salts in the imidization reaction. Potassium salts were synthesised, because these compounds are easier to isolate and characterise than the corresponding DBU salts. It has been reported that hydrolysis of structurally similar amic acids yield anhydrides under acidic conditions.^23,29^ Under basic conditions amic acids derived from primary amines form imides, but this reaction is blocked for amic acids derived from secondary amines. Therefore amic acids salts K12a, K_2_5a and K9a are expected to be stable under basic conditions.

At room temperature the formation of the amic acid salt K12a from PMADE 11 proceeds within 60 minutes, as the pale reaction mixture, in which PMADE 11 is sparsely soluble, is converted to a bright orange solution. Isolation of compound K12a was achieved by precipitation, using water-free conditions, see entry 1, Table 3. Formation of the perylene monoamide triester 14a, a thermally and hydrolytically stable compound, was accomplished by adding butylbromide to the reaction mixture. This reaction is highly efficient and without any purification efforts compound 14a was isolated in 86% yield, see entry 2 in Table 3. Compounds K12a and 14a were characterised by NMR, absorption and emission spectroscopy and mass spectrometry. These techniques provided solid proof of the molecular structure of these compounds, see ESI.† With the successful synthesis and characterization of compounds K12a and 14a it has been proven that the amic acid 12 in an intermediate in the imidization of PMADE 11, which strongly supports the validity of Scheme 3. Also, the synthesis and spectral characterization of K12a facilitates the identification of other perylene monoamic acid diesters salts of 12.

When PDA 1 is reacted with dimethylamine 4a soluble amic acids are formed even faster. The initial reaction mixture, a red suspension, is transformed to a deep orange viscous liquid within 20 minutes. Amic acid salt K_2_5a was isolated by precipitation in dry acetone, see entry 1, Table 2. The dibasic salt K_2_5a is soluble in polar solvents, including water, and exhibits an absorption spectrum that is very similar to that of PTE 3 and perylene-3,4,9,10-tetracarboxylate.^16^ When butylbromide was added after formation of PDAA 5a, PDAE 6a was obtained in 76% yield, virtually free of contaminants, see Table 2, entry 2 and Fig. S1 and S2† in the ESI.† The *cis* and *trans* regioisomers of compound 6a were not distinguishable in the ^1^H NMR spectrum. Broadening of the resonances of the aromatic protons is the sole indication of the presence of two isomers, a result that is in line with the ^1^H NMR spectrum reported for a similar amic acid,^33^ see Fig. S1.† The ^13^C NMR spectrum, however, clearly revealed the formation of two isomers by the presence of two amide and two carboxylate carbons. Also the number of aromatic carbons atoms in the ^13^C NMR spectrum exceeds the 10 expected for either the *cis* or the *trans* isomer, see Fig. S2.† The successful isolation of compounds K_2_5a and 6a confirms that diamic acid salt of 5 is an intermediate in the imidization of PDA and supports the reaction mechanism in Scheme 2.

**Scheme 4:**
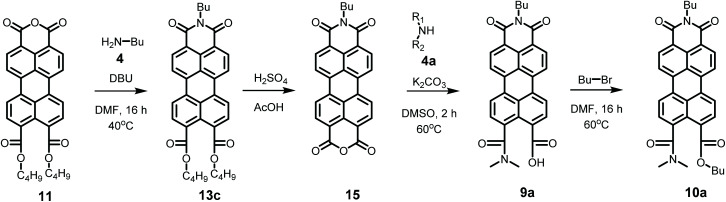
Synthesis of model compounds K9a and 10a.

Model compounds K9a and 10a were synthesized according to Scheme 4. This synthesis starts with the imidization of compound 11 using our mild imidization protocol and produced 13c in almost quantitative yield. Cleavage of the ester functionalities in concentrated sulphuric acid gave compound 15 in high yield.^34^ Ring opening, using dimethylamine 4a, yielded model compound K9a, that was isolated *via* an anhydrous precipitation. The hydrolytically stable and highly soluble compound 10a was obtained from K9a*in situ*, by addition of bromobutane. The identity of compounds 10a and K9a was unambiguously proven by NMR spectroscopy and mass spectrometry, see ESI.†

To provide proof of the intermittency of compound 9 in the imidization reaction we have reacted compound 15 with butylamine under standard conditions (DBU, DMF) to form compound 9c*in situ*, see Scheme S1.† The conversion of this compound to PDI 7c was followed by absorption spectroscopy, *vide infra*, and this experiment has confirmed the intermediary of compound 9c in Scheme 2.

**Fig. 1:**
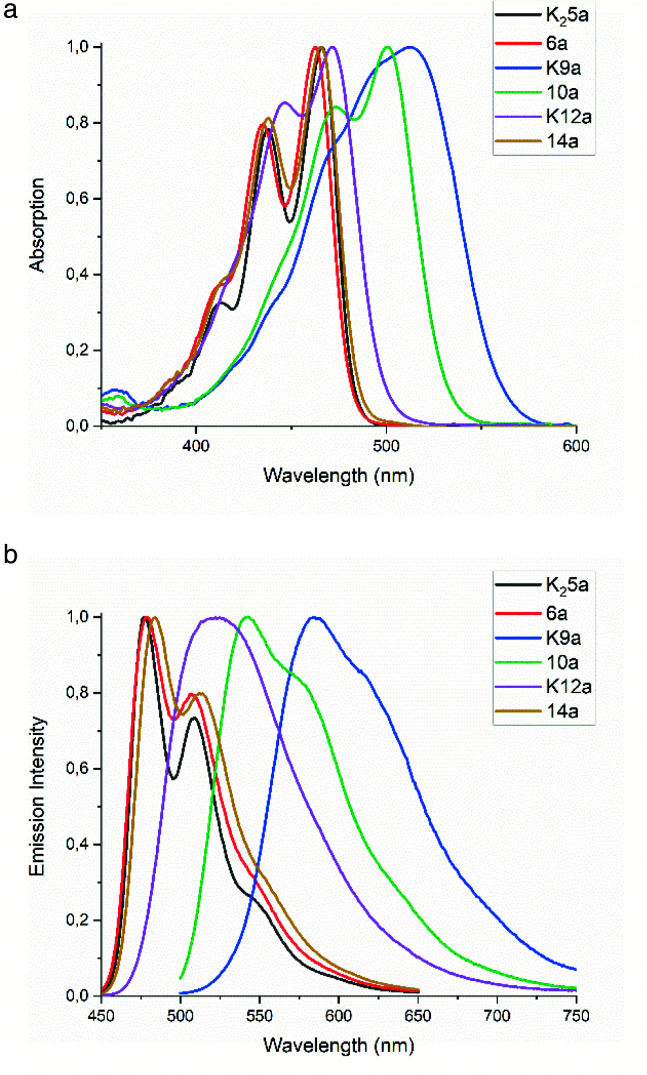
(a) Normalized UV-Vis absorption and (b) normalized fluorescence emission spectra of compounds 3, K_2_5a, 6a, K9a, 10a, K12a and 14a in ethanol.

The absorption and emission spectra of the amic acid salts K_2_5a, K9a, K12a were recorded in water, DMF and ethanol, see Fig. S14 and S16,† while the spectra of the corresponding apolar model compounds 6a, 10a and 14a were recorded in ethanol and chloroform, see Fig. S15.† The spectra of all compounds in the common solvent EtOH are presented in Fig. 1, while photo physical data are presented in Table S3.† The absorption spectra of compounds 3, 13c and 7c in chloroform are depicted in Fig. S3.† The spectra in this Figure serve as a point of reference, for the spectra expected for molecularly dissolved perylene diamic acids, perylene monoamic acid monoimides and perylene diimides, respectively.

The absorption spectra in ethanol of diamic acid salt K_2_5a, diamic ester 6a and the diester monoamic ester 14a resemble that of tetraester 3, with a clear vibronic structure and absorption maxima around 470 and 440 nm. Substitution of a butyl ester by a dimethyl amide (3 to 14a, 6a) resulted in ∼2 nm blue shifts per substitution, whereas substitution of an amic ester by an amic acid salt (6a, 14a to 12a, 5a) resulted in ∼3 nm red shifts per substitution. The absorption of the monoamic ester diester 10a is 32 nm red shifted as compared to tetraester 3, see also Fig. S3.† The absorption spectra of the amic acid salts K12a and K9a are red shifted and show a diminished vibrational structure, as compared to their amic ester analogues. Amic acids, obtained after protonation of their potassium salts, exhibit blue shifted absorption spectra, but are too unstable for full characterisation, see Fig. 8, S12 and S13.†

The emission spectra of PTE 3 and compounds 6a, 9a and 14a are very similar. In all cases the emission spectra are mirror images of the absorption spectra and fluorescence quantum yields *Φ*_F_ are around 0.85. The emission spectra of the amic acid salts K12a and K9a show diminished vibrational structure and quantum yields *Φ*_F_ of 0.96 and 0.65, respectively.

Since the amic acid salts of 5 and 9 are intermediates in the PDI synthesis, it is worthwhile to investigate the (photo) physical data of compounds K_2_5a and K9a in different solvents, see Table 1. Surprisingly, these amic acid salts have poor solubility in DMF, the solvent used for the imide formation, while solubility in water is much higher. Fluorescence quantum yields of compound K_2_5a are very high in water and ethanol, which indicates that this dianionic compound is molecularly dissolved. In DMF, however, the fluorescence quantum yield is a meagre 0.16. For compound K9a the structure-less absorption and emission spectra, as compared to those of compound 10a and 13c, may be indicative for aggregate formation. Fluorescence quantum yields are high in ethanol, but in DMF and water quantum yields of 0.14 and 0.19 reflect substantial quenching. Solutions of K9a in water form a strong foam layer after shaking, which indicates that this compound acts as a surfactant and presumably forms micelles. The corresponding DBU salts of 5a and 9a have not been isolated, but spectra from diluted reaction mixtures containing these DBU salts have been recorded. Absorption and emission spectra as well as fluorescence quantum yields taken from these reaction mixtures closely resemble those of the corresponding potassium salts.

**Table 1:** Spectral data of amic acid salts K_2_5a and K9a in different solvents

Compound	Solvent	*λ*_abs_	*λ*_em_	*Φ*_F_
K_2_5a	DMF	475, 443	487	0.16
K_2_5a	EtOH	466, 438	477.5, 509	0.93
K_2_5a	Water^a^	463, 435	475, 505	1.0
K9a	DMF	521	581	0.14
K9a	EtOH	513	584	0.65
K9a	Water^a^	505	591	0.19

a 0.01 M K_2_CO_3_.

To determine the relative rates of the reaction steps in the imidization process, we have reacted PDA 1 with butyl amine 4c at room temperature and monitored the reaction by sampling the reaction mixture at regular intervals. Subsequently, these samples were diluted and analysed by absorption spectroscopy. Highly fluorescent yellow solutions were obtained after 20 minutes of reaction, both in water and chloroform, as seen in Fig. S4.† This result indicates that all PDA had reacted, and that all reaction products are soluble, both in water and chloroform. The absorption and emission spectra of the reaction product closely resembled those of diamic acid salt K_2_5a, indicating that the reaction product is the DBU salt of 5c. Amic acid formation using K_2_CO_3_ in DMSO, proceeded at a similar rate.

Upon heating the reaction to 60 °C, the amount of diamic acid salt 5c slowly decreased. It is anticipated that the DBU salt of 9c is formed. However, this compound has not been identified in the absorption spectra. Poor solubility of compound 9c in chloroform, the solvent used for dilution, along with the presence of considerable quantities of compounds 5c and 7c, may be the reason for this. The formation of PDI 7c, however, is clearly visible when the imidization reaction is performed, see Fig. S4.†^35^

In summary, these preliminary studies of the reaction of PDA 1 with butylamine 4c, revealed a fast formation of diamic acid 5c in 15–20 minutes at room temperature. At prolonged reaction times and elevated temperatures, typically a few hours at 60 °C, the concentration of diamic acid 5 decreased and monoamic acid monoimide 9c was formed (but not detected). Subsequently the formation of PDI 7c has been observed. It was concluded that the fast ring opening reactions, forming amic acid 5c, and the subsequent slow imidization processes, forming intermediate 9c and eventually the PDI 7c, are decoupled processes that take place at markedly different time scales.

### Reaction optimization by variation of reaction conditions

After validation of Scheme 3 and the establishment of the relative rates of the reaction steps, the imidization reaction was optimized by changing the reaction parameters. The reaction of PDA 1 with 2-ethyl-hexyl amine 4b was chosen for this purpose, because the product of this reaction, PDI 7b, is highly soluble in chloroform and conveniently characterised by NMR spectroscopy. Isolation of the reaction product was extremely convenient; pouring the reaction mixture in water, filtering the precipitate and washing the product with dilute base to remove water-soluble amic acids, yielded pure PDI.^36^

Using the standard conditions (4 molar equivalents of amine and DBU and DMF as the solvent), increasing the reaction yields was achieved by increasing the temperature to 60 °C or the reaction time to 7 days, see entries 5 and S1† in Tables 2 and S1.† From a practical point of view the elevated temperature is preferred. In the original work by Langhals,^10^ Zinc Acetate Zn(OAc)_2_ was used to catalyse the reaction. In our experiments Zn(OAc)_2_ leads to higher yields at room temperature, but addition of this compound no longer has a pronounced effect at elevated temperatures, see entries 2 and 3 in Table S1.† Therefore, and also because we want to keep the reactions clean and simple, we refrain from the addition of Zn(OAc)_2_. In summary, the optimized imidization proceeds at 60 °C, achieves quantitative yields and takes approximately 24 hours.

**Table 2:** Formation of PDIs 7 from the reaction of PDA 1 with different amines, using various reaction conditions

Entry	Perylene anhydride	Reagents	Base	Solvent	Reaction conditions	Yield, in %
1	PDA (1)	4a	K_2_CO_3_	DMSO	24 h	K_2_5a
	0.5 mmol	2 mmol	2 mmol	4 ml	RT	66%
2	PDA (1)	4a	DBU	DMF	24 h	6a
	0.5 mmol	2 mmol	2 mmol	4 ml	RT	76%
		BuBr, 2 mmol				
3	PDA (1)	4b	DBU	DMF	24 h	7b
	0.5 mmol	2 mmol	2 mmol	4 ml	RT	25%
4	PDA (1)	4b	DBU	DMF	24 h	7b
	0.5 mmol	2 mmol	2 mmol	4 ml	RT	60%
		BuBr, 4 mmol				
5	PDA (1)	4b, 4c, 4d^a^, 4f	DBU	DMF	24 h	7b, 7c, 7d, 7f
	0.5 mmol	2 mmol	2 mmol	4 ml	60 °C	>97%
6	PDA (1)	4b, 4c	DBU	DMSO	24 h	4b, 4c
	0.5 mmol	2 mmol	2 mmol	4 ml	60 °C	>97%
7	PDA (1)	4b, 4c, 4d,^a^4e,^b^4f	K_2_CO_3_	DMSO	24 h	7b, 7c, 7d, 7e, 7f
	0.5 mmol	2 mmol	2 mmol	4 ml	100 °C	>97%
8	PDA (1)	4b, 4c	K_2_CO_3_	DMSO	24 h	7b 93%
	0.5 mmol	1.1 mmol	2 mmol	4 ml	100 °C	7c >97%
9	PDA (1)	4e	DBU	DMF	8 h	7e
	0.5 mmol	2 mmol	2 mmol	4 ml	RT	>97%
10	PDA (1)	4b,^c^4c, 4d, 4f	DBU	DMSO	24 h	7b, 7c, 7d >97%
	0.5 mmol	2 mmol	2 mmol	4 ml	RT	4f 90%
				Water^d^, 4 ml		

a First hour kept at room temperature.

b At 80 °C.

c 2 ml of water added.

d Water was added to the reaction after half an hour.

Our next objective is to convert the mild, convenient and efficient imidization reaction into a green(er) and more sustainable process.^37^ This has been accomplished by substitution of the solvent DMF and the base DBU by greener alternatives. Substitution of toxic DMF by DMSO, a non-toxic solvent with similar physical properties, did not affect the reaction yields (entry 6, Table 2). With regard to the substitution of DBU by organic bases, it was observed that DBU is distinctly the best base for this reaction, as was the case for the PTE formation as well.^38^ Reaction with TEA or DIPEA, resulted in significantly lower yields, typically around 20–25% after 24 hours at 60 °C, entry S4, Table S1.† Since most organic bases have toxicity issues similar to those of DBU, substitution of DBU by organic bases was not explored further. Finally, the use of the non-toxic inorganic base K_2_CO_3_ in combination with DMSO, was investigated. At 60 °C, significantly lower yield were observed than with DBU as a base, see entry S5, Table S1.† By raising the reaction temperature to 100 °C, however, the decreased reaction rate was fully compensated for and the reaction gave quantitative yields, see entry 7 in Table 2. This result demonstrates that an entirely green and sustainable process for the imidization of PDA has been developed. Apart from starting material PDA 1, the amine 4b and the reaction product PDI 7b, all inherent to this reaction, the solvent and other reactant are non-toxic and environmentally benign.

Next, imidization reactions of PDA 1 were undertaken using the amines 4c–4f. The PDIs obtained by these reactions bear small alkyl chains (7c–7d), or hydroxyalkyl chains (7e–7f) and only the PDIs 7c and 7f are sparsely soluble in chloroform.^39^ With butylamine 4c, the room temperature reaction was incomplete, but the yield of 7c, (48%) was considerably higher than that of 7b (25%). By increasing the reaction temperature to 60 °C, the reaction yielded 7c in quantitative yield, both in DMF and DMSO. Using the green conditions, DMSO as a solvent and K_2_CO_3_ as a base, quantitative yields were obtained at 100 °C, see entries 5–7 and S6 in Tables 2 and S1.† In order to make the DMSO/K_2_CO_3_ reaction entirely green, the excess of amine was limited to 10%. After facilitating the amic acid formation, by stirring one hour at room temperature, the reaction mixture was heated to 100 °C and PDI 7c was isolated in quantitative yield, see entry 8 Table 2. With this modification only minute amounts of unreacted amine are present in the final reaction mixture. This procedure of forming the amic acid at room temperature prior to imidization at higher temperatures, may also be extremely suited for working with volatile or expensive amines.

In the synthesis of 7d, methylamine 4d dissolved in methanol was used as a reactant. The room temperature reaction yielded 7d in 89% yield. When the reaction was stirred at room temperature for 1 hour, to ensure amic acid formation and prevent evaporation of the amine, quantitative yields were obtained for the DBU reactions at 60 °C as well as the K_2_CO_3_ reaction at 100 °C, see entries 5,7 and S7 in Tables 2 and S1.†^19^ For the reaction with 6-amino-1-hexanol 4f, the yield of the DMF/DBU reaction was 66% at room temperature. Quantitative yields are obtained by raising the temperature to 60 °C, while the K_2_CO_3_ reaction gave quantitative yields at 100 °C, see entries 5, 7 and S8 in Tables 2 and S1.†

**Fig. 2:**
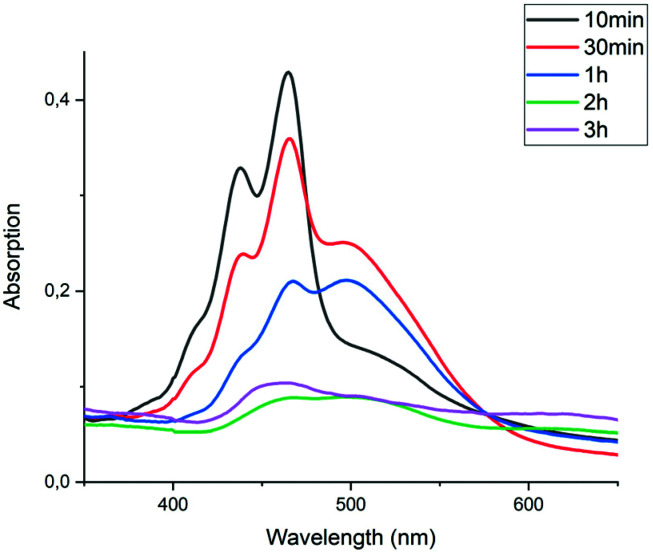
UV-Vis spectra of the reaction of PDA (1) with ethanolamine (4e) in DMF using DBU as the base. Spectra were taken from samples of the reaction mixture diluted in water. The baseline is lifted due to the presence of insoluble PDA 1 and/or PDI 7e. Absorptions do not accurately scale with concentrations in the reaction mixture due to sampling from a heterogeneous reaction mixture.

The imidization reaction with ethanolamine 4e proceeded much faster and quantitative conversion at room temperature was achieved after 8 hours already. When this reaction was sampled and diluted in water, the resulting absorption spectra clearly showed that amic acid formation and imidization both take place at room temperature and that amic acid formation and imidization are no longer decoupled. The initial rise in the concentration of amic acid 5e is reversed within 20 minutes and a broad structure-less red-shifted absorption emerges, which closely resembles that of compound 9a, see Fig. 2 an S16.† Also, a purple precipitate originating from PDI 7e is formed upon diluting the reaction mixture in water early in the reaction. For the same reaction in DMSO, using K_2_CO_3_ as the base, amic acid formation took place within 20 minutes, but imidization was not observed at room temperature, see Fig. S6.† For this reaction quantitative yields were obtained at 80 °C, see entries 7 and 9, Table 2.

The mild imidization reaction with model compound PMADE 11 and the aliphatic amines 4b–4f was also examined. Reactions were performed at room temperature and yields, after purification by column chromatography are displayed in Table 3. Purity and identity of compounds 13b–f were conveniently proven by NMR spectroscopy. From the reported yields in Table 3 it is concluded that in all cases the reaction yields are well over 50% at room temperature, and that ethanolamine 4e is the most reactive amine. These room temperature yields correlate well with those obtained for the bisimides and may be indicative for the reactivity of the amines in the imidization of perylene anhydrides. By increasing the reaction temperatures to 40 °C, quantitative yields can be obtained overnight, as demonstrated with the synthesis of compound 13c, *vide supra*.

**Table 3:** Reaction of amines 4a–4f with PMADE 11

Entry	Perylene anhydride	Reagents	Base	Solvent	Reaction conditions	Yield, in %
1	PMADE (11)	4a	K_2_CO_3_	DMSO	24 h	K12a
	0.19 mmol	0.38 mmol	0.38 mmol	2 ml	RT	61%
2	PMADE (11)	4a	DBU	DMF	24 h	14a
	0.19 mmol	0.38 mmol	0.38 mmol	2 ml	RT	86%
		BuBr, 0.76 mmol				
3	PMADE (11)	4b	DBU	DMF	24 h	13b
	0.19 mmol	0.38 mmol	0.38 mmol	2 ml	RT	55%
4	PMADE (11)	4c	DBU	DMF	24 h	13c
	0.19 mmol	0.38 mmol	0.38 mmol	2 ml	RT	65%
5	PMADE (11)	4d	DBU	DMF	24 h	13d
	0.19 mmol	0.38 mmol	0.38 mmol	2 ml	RT	75%
6	PMADE (11)	4e	DBU	DMF	24 h	13e
	0.19 mmol	0.38 mmol	0.38 mmol	2 ml	RT	80%
7	PMADE (11)	4f	DBU	DMF	24 h	13f
	0.19 mmol	0.38 mmol	0.38 mmol	2 ml	RT	75%

In summary, the reactions of PDA 1 with all aliphatic amines are very convenient and quantitative yields are obtained with DBU in DMF or DMSO at 60 °C and with K_2_CO_3_ in DMSO at 100 °C. Quantitative PDI formation resulted in a convenient workup in which the filtrate is transparent, virtually colourless and free from PTCA derivatives. These results indicate that these reaction conditions are universally applicable for aliphatic amines. For all reactions the ring opening by the amines is fast while the imidization from the amic acid intermediates is rate-determining. Therefore, differences in amine reactivity must be due to differences in rates of imidization of the respective amic acids.

### Reaction kinetics; modelling and measuring

Based on the reaction sequence for the imidization of PDA in Scheme 2, a kinetic model has been constructed. With this kinetic model the reaction mechanism can be validated and the rate constants for the different reaction steps determined. Furthermore, this model may also help to understand why our novel synthetic approach produces PDIs at room temperature already. In addition, factors that limit the rate of the imidization step, can be identified and potentially resolved to make the process even faster at mild and green conditions.

The imidization of PDA consists of 4 reaction steps, two consecutive second-order amic acid forming ring opening reactions, followed by two intramolecular consecutive first-order imidization reactions, see eqn (1). These consecutive reaction steps are identical by mechanism and their rate constants should have near-identical values, *i.e. k*_1_ ≈ *k*′_1_ and *k*_2_ ≈ *k*′_2_. It should also be noted that the stable amic acids salts K_2_5a and K9a, have been synthesised and characterized, which facilitated easy identification of analogous amic acid intermediates.1
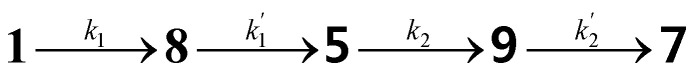


Bimolecular ring opening reactions to form amic acids are fast, *vide supra*, and we have observed that these ring opening reactions are generally decoupled from the slower unimolecular imidization reactions. Therefore, both reactions, the amic acid formation from 1 to 5 and the imidization from 5 to 7, will be described separately.

The first part of the reaction, from PDA to diamic acid 5, is treated in detail in the ESI.† In deriving the kinetic equations, we assume that compound 1 is insoluble, whereas compound 8 will be somewhat soluble. Therefore, the conversion from PDA 1 to compound 8 is the rate-limiting step in this sequence. Accumulation of compound 8, whose spectroscopic signature in molecular solutions is expected to be close to that of compound 9, apart from a ∼4 nm blue shift,^20*b*^ is not anticipated. The rate of consumption of compound 1 and the rate of formation of compound 5 according to eqn (S3) and (S8),† are identical and depicted in Fig. S7.† Experimental data, shown in Fig. S3 and S6,† do not resemble those in Fig. S7,† most likely because the data used for constructing Fig. S3 and S6,† were obtained from sampling inhomogeneous reaction mixtures.

Compounds 5 and 9, participating in the second part of the reaction, are partly soluble in the reaction medium, as indicated by the deep red colour that is developed throughout the reaction. Eqn (2)–(4) describe the rates of the individual reaction steps.2
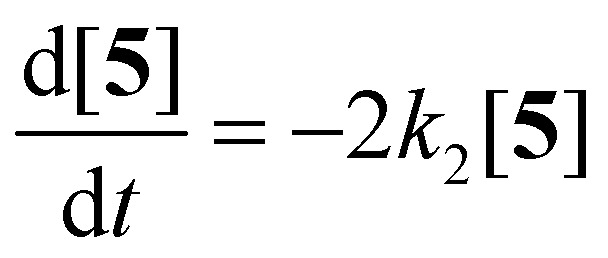
3
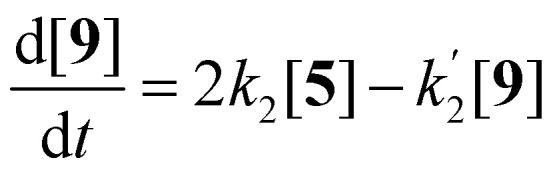
4
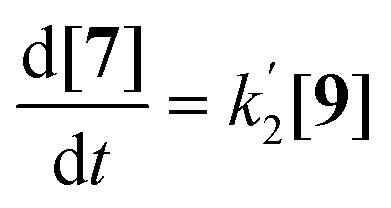


Solutions for eqn (2)–(4) are given by eqn (5)–(7).^40^

5 [5] = [5]_0 _exp^−2*k*_2_*t*^

6
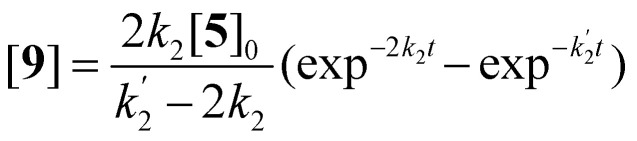
7
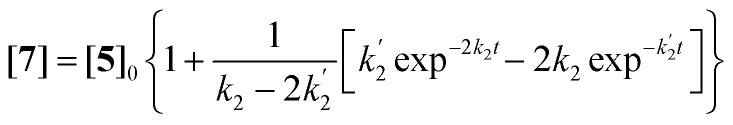


The kinetic model for the imidization of PMADE 11 or PMAMI 15, in which the same reaction rate constants *k*_1_ and *k*_2_ are employed, is described in the ESI (eqn (S9)–(S15)†).

**Fig. 3:**
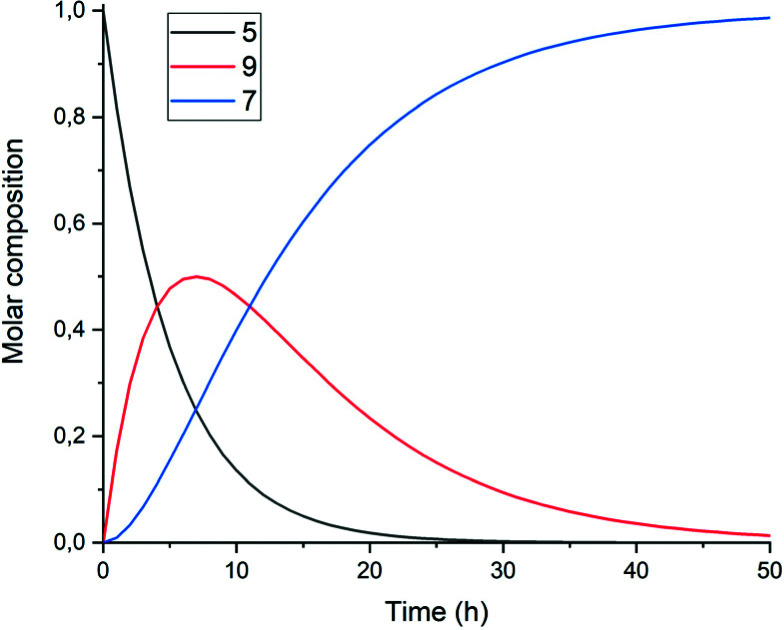
Consumption of diamic acid 5, formation and disappearance of reaction intermediate 9 and formation of PDI 7 according to eqn (5)–(7).

A graphical representation of the composition of the reaction mixture, according to eqn (5)–(7), is given in Fig. 3. The decay of the diamic acid 5 follows first order kinetics, the amic acid monoimide 9 is an intermediate whose concentration builds up to nearly 50% and subsequently declines, while the formation of PDI 7 distinctly lags behind the disappearance of compound 5. Experimental data, to support our kinetic model have not been obtained so far. Qualitatively the consumption of compound 5c and formation of compound 7c have been demonstrated but detection, let alone quantification of the amount of compound 9c has not been achieved.

It should be noted that the amic acid salts of compounds 5 and especially 9 have limited solubility in DMF and DMSO and form aggregates and precipitates at the high concentrations used for the standard reactions. Thus solubility and possibly the type of aggregation of the amic acid salts of 5 and 9,^41^ will govern the rate of the imidization reaction. The solubility and aggregation behaviour of these salts depends on the reaction temperature, the solvent, the substituent R_2_ and the counter ions. Therefore, we anticipate that these are the major factors that determine rates of imide formation.

**Fig. 4:**
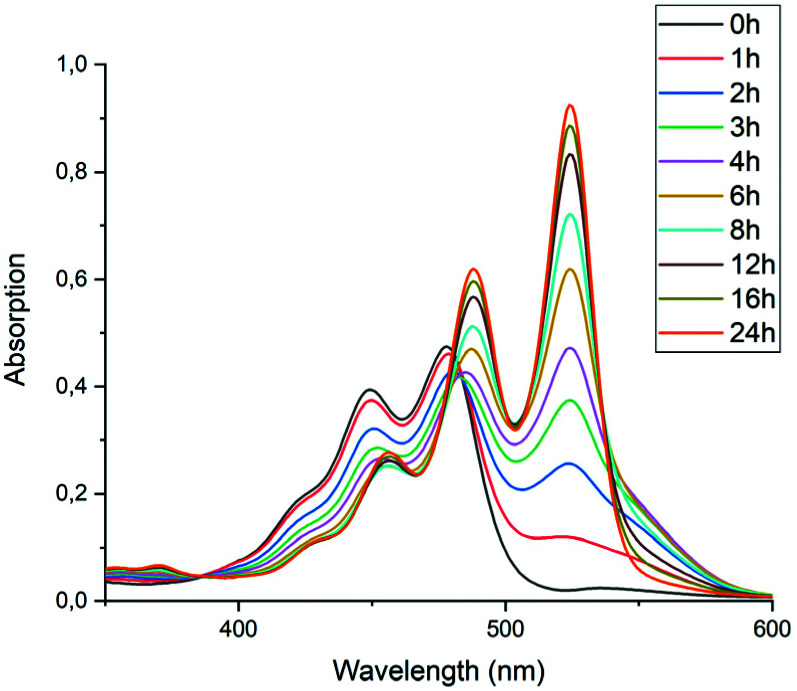
Absorption spectra as a function of time of the reaction of the DBU salt of diamic acid 5c in DMF at room temperature (20 °C) at concentrations around 10^−5^ M.

To test the hypothesis that solubility of amic acid salts limits the rate of imidization reaction, we decided to perform the second stage of this reaction in a highly diluted medium. The imidization reaction with butylamine 4c at room temperature was conducted under standard conditions (DMF, DBU) for 30 minutes, until the formation of the diamic acid 5c was completed. At this stage the reaction mixture was a thick orange slurry, a clear indication that the amic acid salts derived from compound 5c did not fully dissolve. By strongly diluting the reaction mixture with DMF, reaching concentrations in the 10^−5^ molar range,^42^ the remainder of the reaction at room temperature was followed *in situ* by absorption spectroscopy, see Fig. 4.^43^ While the normal reaction reaches 48% conversion in 24 hours, the diluted reaction reaches this conversion in approximately 6 hours and achieves a near-quantitative conversion in 24 hours. The diamic acid salt of 5c, absorbing at 438 and 466 nm, is consumed swiftly, and a broad absorption (extending up to 600 nm), which closely resembles the absorption of model compound K9a, emerges. In time this broad absorption, which originates from the salt of compound 9c, builds up and disappears, while eventually PDI 7c, with distinct absorptions at 488 and 524 nm, is formed. At the end of the reaction a clear, brightly fluorescent solution that contains PDI 7c is obtained. The good solubility of PDI 7c in DMF is really surprising, although it is obvious that these solutions are oversaturated.^44^

**Fig. 5:**
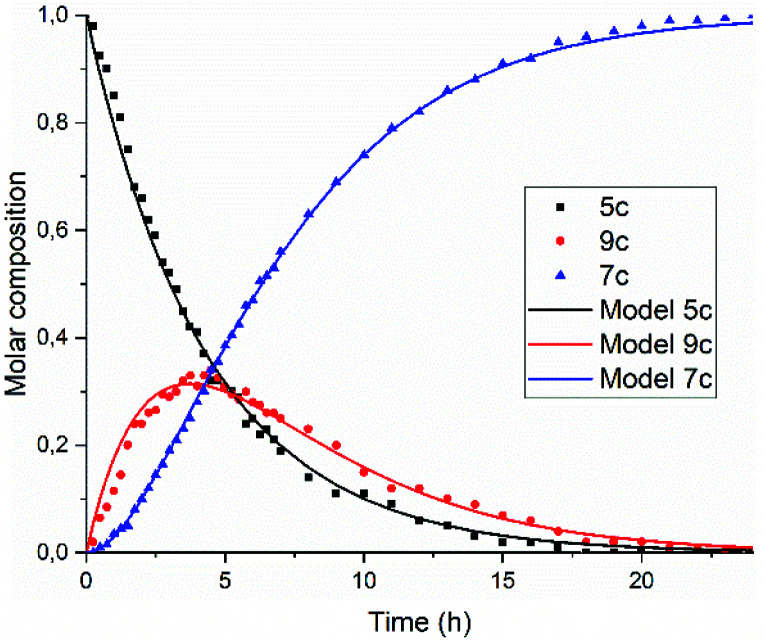
Composition of the reaction mixture obtained from the data displayed in Fig. 5. Solid lines represent the fitting curves obtained using eqn (5)–(7). The solid squires, circles and triangles are concentrations of compounds 5c, 9c and 7c, respectively obtained by analysing the absorption spectra.

In Fig. 5, the composition of the reaction mixture as a function of time is displayed. For determining the composition of the reaction mixture, it was assumed that the spectrum taken at *t* = 0 is that of the DBU salt of 5c and that the spectrum taken after 24 hours is from 7c. Relative concentrations of 5c and 7c were determined by manual fitting of the absorption spectra. The remaining absorption spectrum, obtained by subtracting the contributions from compounds 5c and 7c, is assigned to reaction intermediate 9c. The fitting was done such that the absorption spectra of compound 9c were consistent throughout the series, and closely resembled the spectra that we have previously obtained from compound K9a, see Fig. S14.† Using this procedure, determining the concentration of PDI 7c is fairly consistent and reliable. However, for determining [5c] and [9c] independently this procedure is not suited.^45^

The experimental data in Fig. 5 resemble those predicted by our model depicted in Fig. 3, qualitatively; the monotonic decrease of compound 5c, the delayed formation of compound 7c and the intermediate formation of compound 9c are clearly visible. The curve fitting of the data in Fig. 5 was accomplished by using values for the rate constants *k*_2_ and *k*′_2_ of 3 × 10^−5^ s^−1^and 8 × 10^−5^ s^−1^, respectively. The blue curve for the formation of PDI 7 was constructed by taking identical values for *k*_2_ and *k*′_2_ of 5 × 10^−5^ s^−1^. We anticipate that these seemingly different values for the rate constants *k*_2_ and *k*′_2_ are artefacts that emerged because the concentrations of diamic acid 5c and the monoamic acid 9c could not be determined independently.

**Fig. 6:**
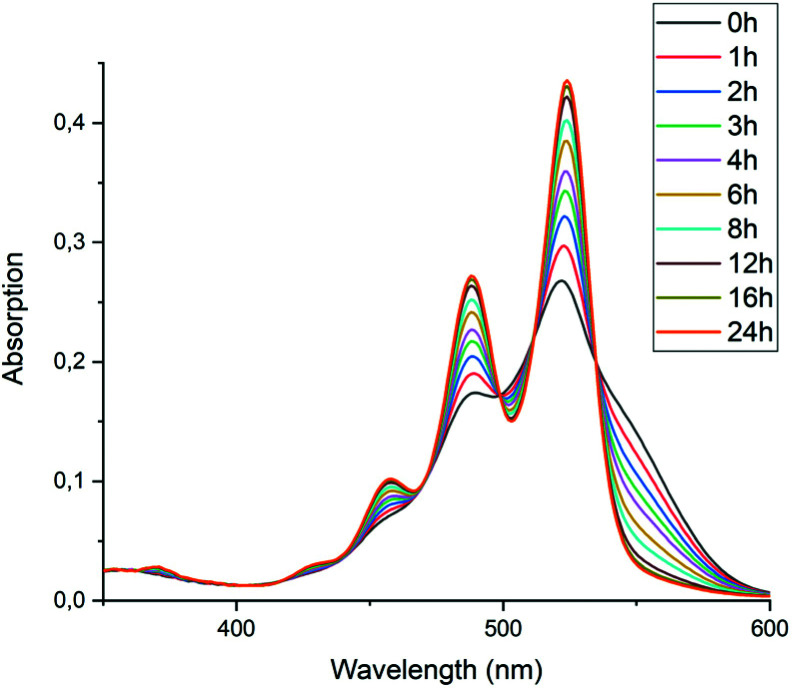
Absorption spectra as a function of time of the reaction of the DBU salt of amic acid monoimide 9c in DMF at concentrations around 5 × 10^−6^ M. The starting solution at *t* = 0 already contains traces of the product 7c. The sharp isosbestic points, at 499, 512 and 535 nm, clearly indicate the presence of only two compounds; 9c and 7c.

**Fig. 7:**
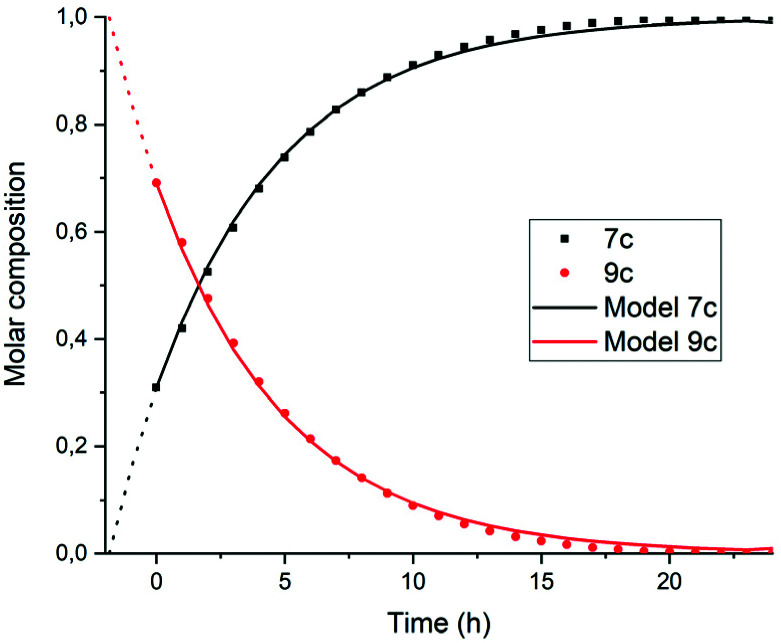
Composition of the reaction mixture obtained from the data displayed in Fig. 7. Solid lines represent the theoretical fitting curves obtained using a first-order decay obtained using eqn (S11) and (S12).†

**Fig. 8:**
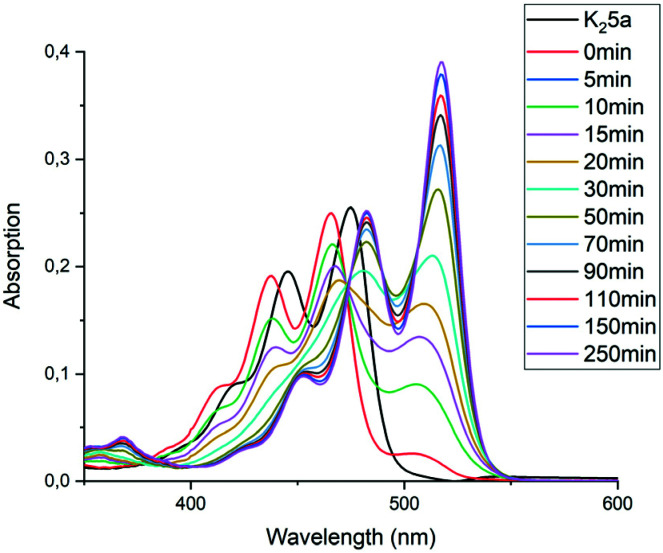
Absorption spectra of compound K_2_5a (black) in DMF and 5a (red), formed after acidification with 1 drop 1 N HCl. In the time-dependent absorption spectra the reaction from 5a (*λ*_max_ = 466 and 438 nm) *via* compound 8a to compound 1 (*λ*_max_ = 517.5 and 482 nm) is visible. Please note that the first spectrum after acidification already contains traces of compound 8a.

To find out if this assumption is correct, the rate constant *k*′_2_ was determined independently by monitoring the reaction from compound 9c to 7c. To this end, monoanhydride 15 was reacted with butylamine 4c under standard conditions for 30 minutes to form compound 9c quantitatively, see Scheme S1.† After dilution with DMF, the conversion from intermediate 9c to PDI 7c by a first order process was monitored, see Fig. 6 and 7. In Fig. 6 three isosbestic points at 499, 512 and 535 nm are observed, which proofs that 9c is converted to 7c directly. The rate constant *k*′_2_ was determined to be 5 × 10^−5^ s^−1^, as demonstrated in Fig. 7. In addition this reaction rate constant was also determined from the reaction of monoamic acid diester 12c to PMIDE 13c, see Fig. S9 and S10.† The imidization rate constant for this reaction had the same value, 5 × 10^−5^ s^−1^. Combining these results it is obvious that the rate constants *k*_2_/*k*′_2_ for the transformation of an amic acid to an imide on the perylene scaffold has a value of 5 × 10^−5^ s^−1^ and is not significantly affected by the substituent(s) at the opposing *peri*-position(s). It is also concluded that kinetic measurements, starting from compound 5c are not appropriate for determining *k*_2_ and *k*′_2_ independently. With the assumption that *k*_2_ = *k*′_2_, however, the reaction rate constant can be determined from such kinetic measurements. To investigate the stability of amic acids under acidic conditions, dilute solutions of the model amic acid salts K_2_5a, K9a and K12a in DMF were acidified with HCl. In line with the reported behaviour of naphthyl-1,8-dicarboxylic amic acids, perylene amic acids were highly unstable.^46^ Protonation was visible by a distinct 9 nm blue shift in the absorption spectrum and formation of 5a was immediately followed by the appearance of an absorption around 505 nm, see Fig. 8. After 30 minutes the spectrum of PDA 1, the end product of the acid decomposition process, became visible and the reaction was finished within 5 hours. From this experiment, the spectrum of the so far undetected monoamic acid monoanhydride 8a was obtained, by subtracting the spectra of compounds 5a and 1 from those of the reaction mixture, in a procedure similar to the one used to analyse the imidization of compound 5c, see Fig. S11.† It appears that compound 8a is molecularly dissolved, as its absorption spectrum exhibits weak vibrational structure and is neither broadened nor red shifted.^47^ Similar experiments, monitoring the acid catalysed anhydride formation from amid acids 9a and 12a are depicted in Fig. S12 and S13.† For these reactions clear and unambiguous isosbestic points were observed, due to the absence of reaction intermediates. PDA, PMAMI and PMADE, formed by amic acid hydrolysis, were molecularly dissolved in DMF and formed highly fluorescent oversaturated solutions. Rate constants for these reactions were not determined, because pH values, on which these reaction rates depend, have not been determined.

Finally it should be noted that fast anhydride formation from amic acids, implies that workup of reaction products in the PDI synthesis must be performed under basic conditions, until all amic acids are removed. Otherwise anhydrides are formed that cannot be removed.

### Room temperature imidization

The dilution experiments depicted in Fig. 4–7 have demonstrated that imidizations of PDA proceed at room temperature and have low activation barriers for imide formation. The overall reaction rate under standard conditions is limited by the solubility of the intermediate amic acid salts of 5 and 9. At high dilution^48^ the imide formation for the DBU reaction in DMF is strongly accelerated, with a rate constants *k*_2_ around 5 × 10^−5^ s^−1^. This allows the reaction to proceed to a >97% conversion in 24 hours at room temperature. It appears, from visual inspection of the reaction mixtures, that the DBU salts of the amic acids 5 and 9 have better solubility, as compared to the potassium salts, and this increased solubility may be the cause of the apparent DBU catalysis of the imidization reaction.

Our findings, that the rate of imidization is limited by the solubility of the amic acid salts 5 and 9, and that dilution has a highly beneficial effect on the overall reaction rate, can be readily exploited for improving the synthesis of PDIs. Taking into account that amic acids 5 and 9 have higher solubility in water (as compared to DMF and DMSO) and that for imidization of NMA and NDA water is an excellent solvent, dilution with water appears to be a viable option to increase the imidization rate even further.

When the synthesis of PDI 7c was started employing the normal reaction protocol (DBU in DMSO) quantitative formation of PDAA 5c was achieved within an hour at room temperature. Subsequently, one equivalent of water (4 ml) was added, resulting in instantaneous dissolution of the DBU salt of 5c to form a deep red solution.^49^ After 24 hours of reaction at room temperature, PDI 7c was obtained in quantitative yield, entry 10 Table 1. It should be noted that this reaction has a 50% yield without water addition. Water is the ideal solvent to add, because in our synthetic protocol water should be added anyway for separating the solid product from the reaction mixture. For amines 4b, 4d and 4f, the same procedure, adding water after amic acid formation was examined. For the formation of 7d, imide formation was quantitative as well, while for the formation of 7f, the yield was high, 90%, but not quantitative. For the formation of 7b, we only added 2 mL of water because the long hydrophobic 2 ethyl hexyl tails limit water solubility. The yield for this reaction was quantitative.^50^

These results imply that aliphatic PDIs can be synthesised in quantitative yields at room temperature, without (7e) or with the addition of water (7b–d). When stable aliphatic amines, such as 4b–f are used, synthesis at 60–100 °C is a reliable and practical option. As it has been reported that PDI synthesis with amino acids under Langhals conditions does not affect the stereochemistry of the appended amino acid,^51^ the 60–100 °C “high temperature” synthesis is appropriate for these compounds as well. However, when temperature sensitive amines are used, imidization at room temperature will be the preferred route.

## Discussion

In this work, we have demonstrated that imidization of PDA, contrary to previous experience, is a process that is conveniently and efficiently performed at moderate temperatures, if need be at room temperature. The first step of the process, formation of diamic acid, is facile and fast in DMF or DMSO with all bases that we have explored. Conditions for PDAA synthesis found in the literature are generally harsh,^33^ but structurally similar amic acids, such as 1,8-naphthyl-dicarboxylic amic acids and 1,3,4,8-naphthyl-tetracarboxylic diamic acids, have been synthesised under much milder conditions.^25^ Also the formation of compound 2, the ester analogue of perylene diamic acid 5, is a process that runs at extremely mild conditions. Therefore the observation that amic acid formation is the easy step in the imide synthesis is not very surprising and in line with our expectations.

Imide formation from amic acids, on the other hand, is anticipated to be much more difficult to achieve. It is reasonable to assume that limited solubility of PTCA derivatives is a major factor necessitating the high temperatures in the conventional PDI syntheses, since imidization from more soluble analogous (di)anhydride substrates is reported at lower temperatures.^11,12^ The activation barrier for the imidization step has been largely unexplored, since all PDI forming reactions have been performed far above room temperature. In contrast to the data obtained from synthetic sources, mechanistic research on the reactivity of 1,8-naphthyl-dicarboxylic amic acids in water indicates that the activation barriers for analogous imidization reactions are low. The conversion from napththyl amic acids to naphthyl imides is a process that takes place reversibly at temperatures as low as 30 °C. These data suggest that in water, a solvent in which PTCA derivatives tend to be poorly soluble, imidization is a process with a low activation barrier. So if polar solvents in which perylene amic acids are soluble can be found, imidization reactions of PDA may proceed at low temperatures.

Our experiments, in which diamic acid salts of 5 were fully dissolved in DMF at high dilution, gave full conversion to PDI 7 at room temperature. This proofs that amic acid salt solubility is the major rate limiting factor for the imization of PDA. Absorption spectra taken at high dilution have clearly demonstrated that all reaction products and intermediates are visible. The diamic acid salts of 5 and even PDI 7 are molecularly dissolved, but monoimide amic acid salts of 9 still forms aggregates at these high dilutions. This finding suggests that the solubility of compound 9 is the major factor limiting the imidization rate. Since we have observed that the solubility of compound 5 is also limited under the standard reaction conditions, the solubility of this compound may be rate limiting as well. For amic acid salts of 5 and 9 it has been observed that potassium salts are less soluble than DBU salts and this solubility difference correlates with a lower reactivity of the reactions when K_2_CO_3_ is used. Another case where high solubility and high reactivity are correlated is the unusual fast reaction of ethanolamine 4e with PDA under standard conditions. The more intense red colour of the reaction mixture observed in this synthesis obviously reflects a higher solubility of the intermediate amic acid salts. Once more, the rate for this reaction is higher when DBU is used as the base. Based on these observations, we conclude that the rate enhancing effect of DBU primarily originates from the higher solubility of the DBU amic acid salts in the reaction medium.

For optimal results, in terms of materials used and rate of the reaction, the bimolecular amic acid formation from PDA is best performed at high concentrations, using only a small excess of amine in as little solvent as possible. For this reaction DMF and DMSO are the only useful solvents identified so far. The second step, the unimolecular imide formation, is limited by the solubility of the amic acid salts and therefore dilution with an appropriate polar solvent is required to speed up this part of the reaction. Enhancing the yield has been proven by strongly diluting the reaction mixture with DMSO or DMF. By adding just one equivalent amount of water the same result, *i.e.* quantitative PDI formation at room temperature, was obtained as well. The use of water in this respect is extremely beneficial, as water is green, cheap and is already used for precipitating the reaction product PDI 7 out of the solution. Further reaction optimization along these lines will be the subject of coming research in our group.

Looking ahead further, attractive opportunity emerge in broadening the scope of the mild imidization reactions. For example in terms of the amines and anhydrides used as starting compounds. It is known from literature that aromatic amines are less nucleophilic and generally have lower yields in imidization reactions.^10,13,14^ Preliminary results of reactions on PMADE 11 have shown that at elevated temperatures aromatic imides can be formed.^52^ Reactions of NDI with butyl amine demonstrated the formation of dibutyl NDI at room temperature already. In the literature this reaction is reported in DMF or acetic acid at temperatures above 100 °C.^53^ Attaching delicate and sensitive (bio)molecules, such as amino terminated nucleic acids and proteins, to strongly fluorescent perylene anhydride moieties like compound 11 is another attractive prospect of our novel synthetic procedure. Very mild reaction conditions can be employed, which is advantageous in view of the high value and limited stability of NH_2_-terminated biomolecules. An additional advantage of the mild imidization protocol is that every step of the process can be monitored and examined in great detail using absorption spectroscopy. This facilitates optimization of the reaction by tuning the individual reaction steps.

## Conclusions

The imidization of PDA 1 has been converted from a harsh “molten imidazole” process, performed at 140–180 °C, into a highly practical, efficient process that runs at strongly decreased temperatures. When using primary aliphatic amines, DBU and either DMF or DMSO as the solvent, full conversion within 24 hours is achieved in the temperature range between 20–60 °C. By using K_2_CO_3_ in DMSO, a truly green and user-friendly synthesis has been developed at reaction temperatures between 80–100 °C.

The reaction sequence for the imidization process *via* various amic acid intermediates, as displayed in Scheme 2, has been confirmed experimentally, aided by the synthesis and full characterization of stable model amic acid salts and amic esters. The formation of diamic acid salts is a fast process that is completed within 30 minutes. Conversion from amic acids to imides is rate determining, due to the low solubility of the intermediate amic acid salts. Diluting the reaction mixture after amic acid formation, strongly increases the reaction rates. The reaction rate constant for the imidization process in DMF was determined to be 5 × 10^−5^ s^−1^ at 20 °C. An improved synthesis, that yields quantitative conversion at room temperature, was developed by adding water after amic acid is formed. An equivalent amount of water is enough to solubilise these amic acids and run the reaction to completion.

Finally, the convenience of the novel synthetic protocol needs to be emphasized. A protective atmosphere, special equipment and advanced synthetic skills are no longer required. When run at the proper temperature, full conversion is obtained and work-up comes down to product precipitation in water followed by a simple filtration. Current research efforts are focussed on broadening the scope of our synthetic protocol by exploring the synthesis of aromatic PDIs and the use of delicate, temperature sensitive amines.

## Experimental

Perylene-3,4-dicarboxylic monoanhydride-9,10-dicarboxylic dibutyl ester (11) was synthesized according to literature procedures.^32*a*^ All other reagents utilized in the syntheses were used as received from the manufacturers, unless otherwise stated. The solvents used were of reagent grade. PDA (1), 97% purity, was obtained from Sigma Aldrich.

### Instrumentation and characterization

The NMR spectra were recorded with 400 MHz pulsed Fourier transform NMR spectrometer in CDCl_3_, DMSO-d6 or MeOH-d4 at room temperature. The chemical shift values are given in ppm and J values in Hz. High-resolution mass spectra were collected on an AccuTOF GCv 4G, JMS-T100GCV, Mass spectrometer (JEOL, Japan). The FD/FI probe (FD/FI) was equipped with an FD Emitter, Carbotec (Germany), FD 10 μm. Typical measurement conditions were as follow: Current rate 51.2 mA min^−1^ over 1.2 min; Counter electrode −10 kV; Ion source 37 V. The samples were prepared in dichloromethane. Absorption measurements were performed using a PerkinElmer Lambda 365 UV−Vis spectrophotometer. Photoluminescence studies were done in Jobin Horiba SPEX Fluorolog 111 Spectrofluorometer. For quantum yield measurements, the formula for optically dilute solutions was used.^54^ Fluorescence quantum yields were determined by the comparative method using perylene-3,4,9,10-tetracarboxylic tetrabutylester (*Φ*_F_ = 0.95 in CH_2_Cl_2_) and *N*,*N*′-di(1-hexylheptyl)perylene-3,4,9,10-tetracarboxy bisimide (*Φ*_F_ = 0.99 in CHCl_3_) and perylene-3,4,9,10-tetracarboxylic tetrapotassium salt (*Φ*_F_ = 1.0 in water) as reference compounds.^16^ Melting points (uncorrected) were recorded on a Gallenkamp melting point apparatus.

Kinetic measurements were performed on the formation of imides from amic acids at low concentrations in 3 mL quartz cuvettes with a 10 mm pathway. Reactions from the anhydrides 1, 11 or 15 to the amic acid salts of 5c, 12c and 9c were performed at room temperature (20 °C) at the smaller scale (200 or 100 mg starting compound) in DMF using DBU as the base at the conditions specified in the synthetic section. For the synthesis of 9c, 100 mg of 15, 2.0 equivalents of DBU and amine in 2 mL DMF were used. After 1 hour of reaction a droplet of the reaction mixture was diluted with DMF and a few droplets of this solution were added to a cuvette placed in a dual beam spectrophotometer. Absorption spectra were taken at room temperature every 5 minutes over a period of 24 hours.

### Synthesis

#### Perylene-3,4,9,10-tetracarboxylic acid 3,9-dimethylamide dipotassium salt (and its 3,10 isomer) K_2_5a

In a 50 mL round bottom flask equipped with a magnetic stirring bar, PDA 1 (200 mg, 0.51 mmol), K_2_CO_3_ (564 mg, 4.08 mmol) and dimethylamine hydrochloride (4a, 166 mg, 2.09 mmol) were suspended in 4 mL DMSO. The suspension was stirred for 2 hours at 60 °C. The reaction mixture was cooled to room temperature. Methanol (10 mL) was added under vigorous stirring and subsequently, acetone (200 mL) was added. The precipitate was filtered off and washed with acetone. The yellow solid was dissolved in methanol (20 mL) and filtered to remove the remaining K_2_CO_3_. The organic solvent was removed from the filtrate by rotary evaporation. This yielded a yellow solid that was dried in a vacuum oven.

Yield: 188 mg, 0.34 mmol, 66%. Mp >350 °C. ^1^H NMR (MeOH-d4, 400 MHz): 8.40 (2H, *J* = 8 Hz, d), 8.38 (2H, *J* = 8 Hz, d), 7.79 (2H, *J* = 8 Hz, d), 7.51 (2H, *J* = 8 Hz, d), 3.10 (6H, s), 3.05 (6H, s). MS (ESI): [M]^2−^ Calculated for C_28_H_20_N_2_O_6_, 240.0666; found: 240.0668 [KM]^−^ Calculated for C_28_H_20_KN_2_O_6_: 519.0964; found: 519.0965.

#### Perylene-3,4,9,10-tetracarboxylic acid 3,9-dimethylamide 4,10-dibutyl ester and perylene-3,4,9,10-tetracarboxylic acid 3,10-dimethylamide 4,9 dibutyl ester 6a

In a 50 mL round bottom flask equipped with a magnetic stirring bar, PDA 1 (200 mg, 0.51 mmol), DBU (621 mg, 4.08 mmol) and dimethylamine hydrochloride (4a, 166 mg, 2.04 mmol) were suspended in 4 mL DMF. The suspension was stirred for 1 hours at 60 °C. 1-bromobutane (699 mg, 5.10 mmol) was added and the reaction mixture was left to stir for 18 hours at 60 °C. The mixture was cooled to room temperature. Water (40 mL) was added under strong stirring, which caused precipitation of the crude product. The reaction product was filtered and the obtained solid was washed extensively with water, until almost no colour was observed in the filtrate, yielding an orange solid that was dried in a vacuum oven.

Yield: 230 mg, 0.39 mmol, 76%. Mp = 190–198 °C. ^1^H NMR (CDCl_3_, 400 MHz): 8.24 (4H, m), 7.86 (4H, *J* = 8 Hz, d), 7.52 (2H, *J* = 8 Hz, d), 4.33 (4H, *J* = 8 Hz, t), 4.15 (6H, s), 3.12 (6H, s), 1.82 (4H, *J* = 8 Hz, quin), 1.52 (4H, m), 1.01 (6H, *J* = 8 Hz, t). ^13^C NMR (CDCl_3_, 101 MHz): 172.13, 172.09, 169.04, 169.02, 133.60, 133.29, 132.73, 131.12, 130.43, 129.44, 129.28, 128.82, 128.32, 127.81, 127.66, 120.97, 120.80, 120.75, 77.04, 76.72, 65.37, 39.80, 35.06, 30.68, 19.30, 13.83. MS (FD): [M]^+^ Calculated for C_36_H_38_N_2_O_6_, 594.2730; found: 594.2764, [M_2_]^+^ Calculated for C_72_H_77_N_4_O_12_ 1188.5460; found: 1188.5600.

#### Perylene-3,4,9,10-tetracarboxylic acid diimides 7

Standard reactions were performed using 200 mg of PDA. Under optimal conditions larger scale reactions for synthetic purposed were performed, using up to 5 grams of PDA. Reaction yields were independent of the scale.

In a 50 mL round bottom flask equipped with a magnetic stirring bar, PDA 1 (200 mg, 0.51 mmol), base (DBU or K_2_CO_3_, 2.04 mmol) and amine 4 (2.04 mmol) were suspended in 4 mL solvent. Reactions with methylamine 4d used a 40% solution in MeOH and were stirred for one hour at RT prior to heating. The suspension was stirred for 24 hours at the desired temperature. The reaction mixture was cooled to room temperature. Water (40 mL) was added under strong stirring, which caused a precipitation of the crude product. The precipitate was left to settle for a maximum of 24 hours. The reaction product was filtered and the obtained solid was washed extensively with 0.01 M K_2_CO_3_ until almost no color was observed in the filtrate and subsequently with water. When the reaction involved the more hydrophobic amine 4b, the filtrate was washed two more times with dilute HCl (1 M, 10 mL) and two more times with demineralized water (25 mL). The solid was dried in a vacuum oven.

##### DBU/DMSO water addition

After addition of the reagents, the suspension was stirred for 1 hour at room temperature. Subsequently 4 mL of water was added (2mL for the synthesis of 7b) and the reaction mixture was stirred for another 23 hours at room temperature. Workup as described above.

#### *N*,*N*′-Di(2-ethylhexyl) perylene-3,4,9,10-tetracarboxylic acid diimide 7b

*DBU*/*DMF method*, RT, from 200 mg PDA: Purple solid, yield 79 mg, 0.13 mmol, 25%.

*DBU*/*DMF method*, 60 °C, from 1.00 g (2.55 mmol) PDA: Purple solid, yield 1.534 g, 2.50 mmol, 98%.

*K*_*2*_ *CO*_*3*_/*DMSO method*, 100 °C, from 1.00 g (2.55 mmol) PDA: Purple solid, yield 1.548 g, 2.52 mmol, 99%.

*DBU*/*DMSO water addition*, from 200 mg PDA: Purple solid, yield 307 mg, 0.50 mmol, 98%.

^1^H NMR (*δ*_H_[ppm], CDCl_3_, 400 MHz): 8.61 (4H, *J* = 8 Hz, d), 8.52 (4H, *J* = 8 Hz, d), 4.14 (4H, m), 1.97 (2H, m), 1.42–1.32 (18H, m), 0.96 (6H, *J* = 8 Hz, t), 0.90 (6H, *J* = 8 Hz, t). ^13^C NMR (*δ*_C_[ppm], CDCl_3_, 101 MHz): 163.70, 134.44, 131.35, 126.30, 123.25, 122.98, 44.34, 37.96, 30.77, 28.71, 24.08, 23.08, 14.10, 10.64.

#### *N*,*N*′-Dibutyl perylene-3,4,9,10-tetracarboxylic acid diimide 7c

*DBU*/*DMF method*, RT, from 200 mg PDA: Purple solid, yield 123 mg, 0.24 mmol, 48%.

*DBU*/*DMF method*, 60 °C, from 1.00 g (2.55 mmol) PDA: Purple solid, yield 1.249 g, 2.49 mmol, 98%.

*K*_*2*_ *CO*_*3*_/*DMSO method*, 100 °C, from 1.00 g (2.55 mmol) PDA: Purple solid, yield 1.239 g, 2.47 mmol, 97%.

*DBU*/*DMSO water addition*, from 200 mg PDA: Purple solid, yield 249 mg, 0.50 mmol, 98%.

^1^H NMR (*δ*_H_[ppm], CDCl_3_, 400 MHz): *δ* 8.74 (4H, *J* = 8 Hz, d), 8.67 (4H, *J* = 8 Hz, d), 4.23 (4H, *J* = 8 Hz, t), 1.74 (4H, *J* = 8 Hz, quin), 1.47 (4H, *J* = 8 Hz, sex), 0.99 (6H, *J* = 8 Hz, t).

#### *N*,*N*′-Dimethyl perylene-3,4,9,10-tetracarboxylic Diimide 7d

*DBU*/*DMF method*, RT, from 200 mg PDA: Red solid, yield 180 mg, 0.43 mmol, 84%.

*DBU*/*DMF method*, RT, from 200 mg PDA: Red solid, yield 210 mg, 0.50 mmol, 98%.

*K*_*2*_ *CO*_*3*_/*DMSO method*, 100 °C, from 200 mg PDA: Red solid, yield 207 mg, 0.49 mmol, 92%.

*DBU*/*DMSO water addition*, from 200 mg PDA: Red solid, yield 214 mg, 0.51 mmol, 100%.

^1^H NMR (*δ*_H_[ppm], D_2_SO_4_, 400 MHz): 9.15 (4H, *J* = 8 Hz, d), 9.10 (4H, *J* = 8 Hz, d), 3.93 (6H, s). FTIR: 742, 808, 850, 863, 1021, 1053, 1127, 1156, 1184, 1237, 1284, 1326, 1358, 1400, 1446, 1507, 1578, 1594, 1659, 1698.

#### *N*,*N*′-Di-(2-hydroxyethyl) perylene-3,4,9,10-tetracarboxylic acid diimide 7e

*DBU*/*DMF method*, RT, 1.00 g (2.55 mmol) PDA: Dark purple solid, yield 1.196 g, 2.40 mmol, 98%.

*K*_*2*_ *CO*_*3*_/*DMSO method*, 80 °C, 1.00 g (2.55 mmol) PDA: Dark purple solid, yield 1.177 g, 2.36 mmol, 97%.

^1^H NMR (*δ*_H_[ppm], D_2_SO_4_, 400 MHz): 9.21–9.07 (8H, m), 5.64 (4H, *J* = 8 Hz, t), 4.85 (4H, *J* = 8 Hz, t).

#### *N*,*N*′-Di-(6-hydroxyhexyl) perylene-3,4,9,10-tetracarboxylic acid diimide 7f

*DBU*/*DMF method*, RT, 200 mg PDA: Purple solid, yield 199 mg, 0.34 mmol, 66%.

*DMF*/*DBU method*, 60 °C, 200 mg PDA: Purple solid, yield 297 mg, 0.50 mmol, 97%.

*K*_*2*_ *CO*_*3*_/*DMSO method*, 100 °C, 200 mg PDA: Purple solid, yield 296 mg, 0.50 mmol, 97%.

*DBU*/*DMSO water addition*, 200 mg PDA: Purple solid, yield 270 mg, 0.46 mmol, 90%.

^1^H NMR (CDCl_3_, 400 MHz): 8.77 (4H, *J* = 8 Hz, d), 8.72 (4H, *J* = 8 Hz, d), 4.37 (4H, *J* = 8 Hz, t), 4.25 (4H, *J* = 8 Hz, t), 8.69 (8H, m), 1.50 (8H, m).

#### *N*-Butyl perylene-3,4,9,10-tetracarboxylic acid 3,4-imide 9-dimethylamide potassium salt K9a

In a 25 mL round bottom flask equipped with a magnetic stirring bar, PMAMI 15 (50 mg, 0.11 mmol), K_2_CO_3_ (650 mg, 0.47 mmol) and dimethylamine hydrochloride (4a, 20 mg, 0.25 mmol) were suspended in 1 mL DMSO. The suspension was stirred for 2 hours at 60 °C. The mixture was cooled to room temperature. Methanol (1 mL) was added under vigorous stirring and subsequently 20 mL of a 1 : 1 acetone/petroleum ether mixture was added. The precipitate is filtered off and washed with acetone/petroleum ether. The purple solid was dissolved in ethanol (5 mL) and filtered to remove the remaining K_2_CO_3_. The organic solvent was removed from the filtrate by rotary evaporation. This yielded a yellow solid that was dried in a vacuum oven.

Yield: 42 mg, 0.08 mmol, 73%. Mp >350 °C. ^1^H NMR (MeOD, 400 MHz): 8.59–8.45 (6H, m), 7.88 (1H, *J* = 8 Hz, d), 7.59 (1H, *J* = 4 Hz, d), 4.16 (2H, *J* = 8 Hz, t), 3.13 (3H, s). 3.10 (3H, s), 1.73 (2H, *J* = 8 Hz, quin), 1.47 (2H, *J* = 8 Hz, sex), 1.02 (3H, *J* = 8 Hz, t). MS (ESI): [M]^−^ Calculated for C_28_H_20_N_2_O_6_, 240.0666; found: 240.0668 [KM]^−^ Calculated for C_30_H_23_N_2_O_5_, 491.1612; found: 491.1598.

#### *N*-Butyl perylene-3,4,9,10-tetracarboxylic acid 3,4-butylimide 9-dimethylamide 10-butylester 10a

In a 25 mL round bottom flask equipped with a magnetic stirring bar, PMAMI 15 (50 mg, 0.11 mmol), DBU (680 mg, 0.45 mmol) and dimethylamine hydrochloride 4a (20 mg, 0.25 mmol) were suspended in 1 mL DMF. The suspension was stirred for 1 hours at 60 °C. 1-bromobutane (153 mg, 1.12 mmol) was added and the reaction mixture was left to stir for 3 hours at 60 °C. The mixture was cooled to room temperature. Water (10 mL) was added under strong stirring, which caused precipitation of the crude product. The suspension was transferred to a separation funnel and chloroform (10 mL) was added. The organic layer was collected, and washed two times with salt solution (10 mL, 2 M NaCl). The organic layer was collected and dried on Na_2_SO_4_. After filtration, the organic solvent was removed by rotary evaporation, yielding a red solid.

Yield: 53 mg, 0.097 mmol, 77%. Mp = 153–154 °C. ^1^H NMR (CDCl_3_, 400 MHz): 8.63 (2H, *J* = 8 Hz, d), 8.46 (4H, m), 7.95 (1H, *J* = 8 Hz, d), 7.63 (1H, *J* = 8 Hz, d), 4.35 (2H, *J* = 8 Hz, t), 4.22 (2H, *J* = 8 Hz, t), 3.18 (3H, s), 3.13 (3H, s), 1.84 (2H, m), 1.75 (2H, m), 1.47 (2H, m), 1.01 (6H, m). ^13^C NMR (CDCl_3_, 101 MHz): 168.56, 163.38, 163.34, 135.33, 135.14, 132.43, 131.51, 131.01, 130.90, 129.92, 129.22, 127.84, 125.48, 122.68, 122.44, 121.55, 121.31, 121.22, 120.94, 65.74, 40.24, 39.84, 35.12, 30.67, 30.20, 20.44, 19.31, 13.87, 13.85. MS (FD): [M]^+^ Calculated for C_34_H_32_N_2_O_5_, 548.2311; found: 548.2326, [M_2_]^+^ Calculated for C_68_H_62_N_4_O_10_, 1096.4622; found: 1096.4767.

#### Perylene-3,4,9,10-tetracarboxylic acid 3-dimethylamide 9,10-dibutylester potassium salt K12a

In a 25 mL round bottom flask equipped with a magnetic stirring bar, PMADE 11 (50 mg, 0.096 mmol), K_2_CO_3_ (56 mg, 0.40 mmol) and dimethylamine hydrochloride (4a, 17 mg, 0.21 mmol) were suspended in 1 mL DMSO. The suspension was stirred for 2 hours at 60 °C. The mixture was cooled to room temperature. While vigorously stirring, methanol (1 mL) was added. Subsequently, 20 mL of a 1 : 1 acetone/petroleum ether mixture was added. The resulting yellow precipitate was left to settle and filtered off. The yellow solid was dissolved in ethanol (5 mL) and filtered to remove the remaining K_2_CO_3_. The organic solvent was removed from the filtrate by rotary evaporation. This yielded a yellow solid that was dried in a vacuum oven.

Yield: 35 mg, 0.063 mmol, 61%. Mp >350 °C. ^1^H NMR (MeOD, 400 MHz): 8.40 (2H, *J* = 8 Hz, d), 8.36 (2H, *J* = 4 Hz, d), 7.76 (2H, *J* = 8 Hz, d), 7.81 (1H, *J* = 8 Hz, d), 7.51 (1H, *J* = 8 Hz, d), 4.30 (4H, *J* = 8 Hz, t), 3.12 (3H, s), 3.08 (3H, s), 1.78 (4H, *J* = 8 Hz, quin), 1.52 (4H, *J* = 8 Hz, sex), 1.01 (6H, *J* = 8 Hz, t). MS: (ESI): [M]^−^ Calculated for C_34_H_32_NO_7_, 566.2148; found: 566.2165, [KM_2_]^−^ Calculated for C_68_H_62_KN_2_O_14_, 1171.4000; found: 1171.3961.

#### Perylene-3,4,9,10-tetracarboxylic acid-3,4-monoimide-9,10-dibutylesters 13

In a 25 mL round bottom flask equipped with a magnetic stirring bar, PMADE 11, (100 mg, 0.19 mmol), DBU (58 mg, 0.38 mmol) and amine (4, 0.38 mmol) were suspended in 2 mL DMF. The reaction mixture was stirred at room temperature for 24 hours. Afterwards the clear solution was poured in a sodium bicarbonate solution (20 mL, 1 M) and left for at least 30 minutes. The resulting suspension was filtered on a paper filter and the solid was washed with water until the filtrate was colorless. The solid was dried in a vacuum oven and the product was dissolved in DCM and subsequently purified using normal phase column chromatography (SiO_2_/DCM/MeOH).

#### *N*-(2-Ethylhexyl)perylene-3,4,9,10-tetracarboxylic acid 3,4-imide 9,10-dibutylester 13b

Column chromatography on silica/0.5% MeOH in DCM yielded a red waxy solid.

Yield: 66 mg, 0.10 mmol 55%. Mp = 178 °C. ^1^H NMR (CDCl_3_, 400 MHz): 8.41 (2H, d, 8 Hz), 8.20 (2H, d, 4 Hz), 8.17 (2H, d, 4 Hz), 7.97 (2H, *J* = 8 Hz, d), 4.36 (4H, *J* = 8 Hz, t), 4.11 (2H, m), 1.96 (1H, *J* = 8 Hz, sep), 1.82 (4H, *J* = 8 Hz, quin), 1.53 (4H, *J* = 8 Hz, sex), 1.42–1.32 (8H, m), 1.02 (6H, *J* = 8 Hz, t), 0.96 (3H, *J* = 8 Hz, t), 0.90 (3H, *J* = 8 Hz, t). ^13^C NMR (CDCl_3_, 101 MHz): 168.20, 163.78, 135.06, 131.86, 131.78, 131.18, 130.15, 129.04, 128.93, 128.83, 125.67, 122.37, 121.95, 121.60, 77.31, 76.99, 76.67, 65.54, 44.18, 37.97, 30.78, 30.62, 28.71, 24.09, 23.08, 19.25, 14.09, 13.79, 10.64. MS (FD): [M]^+^ Calculated for C_40_H_43_NO_6_, 633.3090; found: 633.3073.

#### *N*-Butyl perylene-3,4,9,10-tetracarboxylic acid 3,4-imide 9,10-dibutylester 13c

Column chromatography on silica/0.5% MeOH in DCM yielded a red waxy solid. Yield: 71 mg, 0.12 mmol, 65%. Mp = 265 °C.

Synthesis for compound 15, performed at 40 °C overnight (16 h) starting from 200 mg (0.38 mmol) 11: Yield: 215 mg, 0.37 mmol, 97% red solid. Traces of PDI 7c are visible in the NMR spectrum of the crude reaction product, presumably originating from PDA contaminant in the starting material. Yield after column chromatography, 204 mg, 0.35 mmol, 92%.

^1^H NMR (CDCl_3_, 400 MHz): 8.17 (2H, *J* = 8 Hz, d), 7.91 (2H, *J* = 8 Hz, d), 7.84 (4H, m), 4.36 (4H, *J* = 8 Hz, t), 4.12 (2H, *J* = 8 Hz, t), 1.82 (4H, *J* = 8 Hz, quin), 1.74 (2H, *J* = 8 Hz, quin), 1.52 (6H, m), 1.03 (9H, m). ^13^C NMR (CDCl_3_, 101 MHz): 168.12, 163.15, 134.61, 131.67, 131.47, 130.73, 129.97, 128.81, 128.47, 128.44, 125.21, 122.14, 121.67, 121.27, 77.33, 77.01, 76.70, 65.56, 40.26, 30.64, 30.17, 20.43, 19.28, 13.86, 13.82. MS (FD): [M]^+^ Calculated for C_38_H_39_NO_7_, 577.2464; found: 577.2443.

#### *N*-Methyl perylene-3,4,9,10-tetracarboxylic acid 3,4-imide 9,10-dibutylester 13d

Column chromatography on silica/1.0% MeOH in DCM yielded a red waxy solid.

Yield: 77 mg, 0.14 mmol, 75%. Mp = 263 °C. ^1^H NMR (CDCl_3_, 400 MHz): 8.32 (2H, *J* = 8 Hz, d), 8.11 (2H, *J* = 8 Hz, d), 8.07 (2H, *J* = 8 Hz, d), 7.93 (2H, *J* = 8 Hz, d), 4.35 (4H, *J* = 8 Hz, t), 3.50 (3H, s), 1.81 (4H, *J* = 8 Hz, quin), 1.51 (4H, *J* = 8 Hz, sex), 1.01 (6H, *J* = 8 Hz, t). ^13^C NMR (CDCl_3_, 101 MHz): 168.15, 163.53, 135.09, 131.83, 131.69, 131.02, 130.13, 128.96, 128.66, 128.63, 125.52, 122.37, 121.63, 121.48, 77.30, 76.99, 76.67, 65.57, 30.61, 26.98, 19.25, 13.79. MS (FD): [M]^+^ Calculated for C_33_H_29_NO_6_, 535.1995; found: 535.1983.

#### *N*-(2-Hydroxyethyl)perylene-3,4,9,10-tetracarboxylic acid 3,4-imide 9,10-dibutylester 13e

Column chromatography on silica/0.5 to 2.5% MeOH in DCM yielded a red waxy solid.

Yield: 82 mg, 0.14 mmol, 80%. Mp = 305 °C. ^1^H NMR (CDCl_3_, 400 MHz): 7.92 (2H, *J* = 8 Hz, d), 7.69 (2H, *J* = 8 Hz, d), 7.59 (2H, *J* = 8 Hz, d), 4.36–4.31 (6H, m), 4.03 (2H, *J* = 4 Hz, t), 2.89 (br, 1H), 1.84 (4H, *J* = 8 Hz, quin), 1.54 (4H, *J* = 8 Hz, sex), 1.04 (6H, *J* = 8 Hz, t). ^13^C NMR (CDCl_3_, 101 MHz): 168.00, 163.73, 134.40, 131.68, 130.84, 130.63, 129.84, 128.46, 127.95, 127.95, 124.61, 122.12, 120.99, 120.98, 77.33, 77.01, 76.69, 65.62, 61.27, 42.72, 30.63, 19.28, 13.84. MS (FD): [M]^+^ Calculated for C_34_H_31_NO_7_, 565.2101; found: 565.2100.

#### *N*-(6-Hydroxhexyl)perylene-3,4,9,10-tetracarboxylic acid 3,4-imide 9,10-dibutylester 13f

Column chromatography on silica/0.5 to 2% MeOH in DCM yielded a red waxy solid.

Yield: 88 mg, 0.14 mmol, 75%. Mp = 195 °C. ^1^H NMR (CDCl_3_, 400 MHz): 8.09 (2H, *J* = 8 Hz, d), 7.84 (2H, *J* = 8 Hz, d), 7.80 (2H, *J* = 8 Hz, d), 7.75 (2H, *J* = 8 Hz, d), 4.36 (4H, *J* = 8 Hz, t), 4.08 (2H, *J* = 8 Hz, t), 3.86 (2H, *J* = 8 Hz, t), 1.83 (6H, m), 1.76 (2H, m), 1.64 (2H, m), 1.52 (6H, m), 1.03 (6H, *J* = 8 Hz, t). ^13^C NMR (CDCl_3_, 101 MHz): 168.11, 163.11, 134.57, 103.66, 129.96, 128.74, 128.37, 128.30, 125.06, 122.17, 121.47, 121.19, 65.58, 62.70, 40.22, 32.56, 30.64, 27.88, 26.64, 25.22, 19.28, 13.83. MS (FD): [M]^+^ Calculated for C_38_H_39_NO_7_, 621.2727; found: 621.2703.

#### Perylene-3,4,9,10-tetracarboxylic acid 3-dimethylamide 4,9,10-tributylester 14a

In a 25 mL round bottom flask equipped with a magnetic stirring bar, PMADE 11 (100 mg, 0.19 mmol), DBU (117 mg, 0.77 mmol) and dimethylamine hydrochloride (4a, 31 mg, 0.38 mmol) were suspended in 2 mL DMF. The suspension was stirred for 1 hours at 60 °C. 1-bromobutane (131 mg, 0.96 mmol) was added and the reaction mixture was left to stir for 18 hours at 60 °C. The mixture was cooled to room temperature. Water (40 mL) was added under strong stirring, which caused a precipitation of the crude product. The reaction product was filtered and the obtained solid was washed extensively with water, until almost no colour was observed in the filtrate, yielding a yellow solid that was dried in a vacuum oven.

Yield: 103 mg, 0.17 mmol, 86%. Mp = 123–127 °C. ^1^H NMR (CDCl_3_, 400 MHz): 8.06 (4H, m), 7.92 (2H, m), 7.90 (1H, *J* = 8 Hz, d), 7.75 (1H, *J* = 8 Hz, d), 7.42 (1H, d), 4.34 (6H, *J* = 8 Hz, t), 3.15 (3H, s), 3.13 (3H, s), 1.87–1.76 (6H, m), 1.57–1.48 (6H, m), 1.05–0.99 (9H, m). ^13^C NMR (CDCl_3_, 101 MHz): 171.92, 168.90, 168.65, 134.11, 133.22, 133.07, 132.63, 130.92, 130.41, 130.31, 130.00, 129.68, 129.31, 128.89, 128.83, 128.69, 128.43, 127.75, 121.42, 121.33, 120.95, 120.68, 65.49, 65.23, 39.80, 35.08, 30.66, 19.29, 13.80. MS (FD): [M]^+^ Calculated for C_38_H_41_NO_7_, 623.2883; found: 623.2886.

#### *N*-(2-Butyl)perylene-3,4,9,10-tetracarboxylic acid 3,4-anhydride 9,10-imide 15

Concentrated sulphuric acid (7 mL) was added to a 50 mL round bottom flask, closed off with a drying tube charged with calcium chloride. 13c (298 mg, 0.517 mmol) was then dissolved by stirring at room temperature for a few minutes. The mixture was left to stir at room temperature overnight. The strongly acidic mixture was slowly poured on ice. After the ice melted, the obtained suspension was filtered washed with water and dried by air and further dried in a vacuum oven. This yielded a red solid.

Yield: 215 mg, 93%. ^1^H NMR (CDCl_3_, 400 MHz): 8.81 (8H, m), 4.26 (2H, *J* = 8 Hz, t), 1.76 (2H, *J* = 8 Hz, quin), 1.49 (2H, *J* = 8 Hz, sex), 1.00 (3H, *J* = 8 Hz, t).

## Conflicts of interest

There are no conflicts to declare.

## Supplementary Material

QO-009-D1QO01723C-s001
