# Supplementary material for: Room temperature synthesis of perylene diimides facilitated by high amic acid solubility
Source: Org Chem Front. 2022 Jan 10;9(4):1090–108. doi: 10.1039/d1qo01723c (PMC8846406; doi:10.1039/d1qo01723c)
Supplement: QO-009-D1QO01723C-s001 [file QO-009-D1QO01723C-s001.pdf]

Electronic Supplementary Material (ESI) for Organic Chemistry Frontiers.
This journal is © the Partner Organisations 2022

Supplementary Material for

Room temperature synthesis of perylene diimides facilitated by high amic acid solubility

Markus C. Kwakernaak, Marijn Koel, Peter J. L. van den Berg, Erik M. Kelder and Wolter F. Jager*

Department of Chemical Engineering, Delft University of Technology, Van der Maasweg 9, 2629 HZ Delft, The Netherlands.

* W.F.Jager@tudelft.nl

Table of Contents

1. Experimental Procedures……………………………………..………3

2. Kinetic Modelling……………………………………………………..4

3. Graphs and Figures……………………………………………………6

4. Syntheses……………………………………………………………….18

5. UV-Vis Absorption and Fluorescence Spectra…………………..….19

6. NMR and Mass Spectra………………………..…………………..23

1. Experimental Procedures

Kinetic Measurements

Analysis of the absorption spectra was performed using MS Excel. For the simple reactions (from **12c** to **13c** or **9c** to **7c**), the first spectrum (obtained at t=0) was assumed to be from the starting compound (amic acid) and the last spectrum (obtained at t= 24h) was from the product (imide). Cleanup of the spectrum of the starting compound may be required, because the starting compound already contains small amounts of the reaction product. This cleanup generally comes down to subtracting a fraction of the product spectrum from the starting compound spectrum. The spectra of the reaction mixtures were simulated by a linear combination of the spectra from the starting compound and the reaction product:

Spectrum reaction mixture= x*Spectrum starting compound+(1-x)* Spectrum product

$0 \leq x \leq 1$

The best fit was obtained when the simulated spectra, calculated according to the formula above, were identical to the experimental spectra. This was visualized by subtracting the simulated spectrum from that of the reaction mixture. A flat base line should result and if need be this process is automated by requiring the choice of x such that the sum of all absorption data points is minimised.

For the more complex reaction from diamic acid **5c** to diimide **7c** via the intermediate **9c**, finding the composition of the reaction mixture was less accurate. Here we assume that the first spectrum (obtained at t=0) was from the starting compound (diamic acid **5c**) and the last spectrum (obtained at t= 24h) was from the product (diimide **7c**). The spectrum from the reaction intermediate **9c** was unknown, but is expected to resemble that of compound **9a**. Cleanup of the spectrum of the starting compound may be required, because the starting compound already contains a little of the intermediate product **9c**. This cleanup generally comes down to flattening the spectrum of compound **5c** above 520 nm.

Simulated reaction mixture= x*Spectrum **5c**+ y*Spectrum **9c** +(1-(x+y))* **7c**

$0 \leq (x+y) \leq 1$

Since the spectrum of **9**c is unknown, various values of x and 1-(x+y) are tested. By subtracting x***5c**+ (1-(x+y))***7c** from the spectrum of the reaction mixture, the spectrum of y***9c** is obtained. Doing this in such a manner that the spectrum of **9c** is consistent in shape and resembling that of **9a**, resulted in values of x and y for the different reaction times and thereby the molecular composition. This process is more complex, less accurate (in independently finding x and y) and cannot be automated easily.

2. Kinetic Modelling

In the first part of the reaction from PDA **1** to the diamic acid **5**, all steps are second order reactions, as described by Equations S1-3.

$$\frac{d[\mathbf{1}]}{dt} = -2k_1[\mathbf{1}][\mathbf{4}] \approx -k[\mathbf{4}]; \quad k << k_1[\mathbf{1}] \qquad eq\ S1$$

$$\frac{d[\mathbf{8}]}{dt} = k[\mathbf{4}] - k_1^{'}[\mathbf{8}][\mathbf{4}] \qquad eq\ \mathrm{S2}$$

$$\frac{d[\mathbf{5}]}{dt} = k_1^{'}[\mathbf{8}][\mathbf{4}] \qquad eq\ \mathrm{S3}$$

Because PDA **1** is insoluble (in DMF or DMSO), the conversion to compound **8** is not a second order reaction but a heterogeneous reaction. This reaction is best described as a pseudo-first order reaction, whose rate scales with the concentration of amine **4**. The apparent rate constant for this reaction k is much smaller than k_1[**1**]. Equations S1 and S3 describe the consumption of PDA **1** and the formation of diamic acid **5**. For setting up Eqs S4-S8, we have made the assumption that compound **8**, to some extent, is soluble in the reaction mixture and will be consumed by a fast second-order reaction. This reaction consumes a second molecule of amine **4** , which results in the relation between [**1**] and [**4**] formulated in Equation S4.

$$[\mathbf{4}] = 2[\mathbf{1}]_0 + 2[\mathbf{1}]) \qquad eq\ S4$$

$$\frac{d[\mathbf{1}]}{dt} = -k(2[\mathbf{1}]_0 + 2[\mathbf{1}]) \qquad eq\ S5$$

$$[\mathbf{1}] = 2[\mathbf{1}]_0 \exp^{-2kt} - [\mathbf{1}]_0 \qquad eq\ S6$$

$$\frac{d[\mathbf{8}]}{dt} = k[\mathbf{4}] - k_1^{'}[\mathbf{8}][\mathbf{4}] \approx o \qquad eq\ \mathrm{S7}$$

$$[\mathbf{5}] = 2[\mathbf{1}]_0 - 2[\mathbf{1}]_0 \exp^{-2kt} \qquad eq\ \mathrm{S8}$$

The rates of consumption of compound **1** and the rate of formation of compound **5** according to Equations S6 and S8, are depicted in Figure S11. Experimental data concerning the formation of **5c** and **5e** are obtained from UV experiments are depicted in Figures S4b and S8b, respectively. These experimental data, which demonstrate that formation of amic acid **5** is completed in 15-20 minutes, are crude due to non-reproducible sampling from a heterogeneous reaction mixture and do not correlate well with the prediction from our model.

The imide forming reactions from **9** to **7** and **12** to **13** are first-order reactions. The reaction rate constants for these reactions should be very similar and equal k_2 and k'_2 from the reactions that forms PDI **7** from amic acid **5**. The formation of compounds **7** and **13** and the consumption of compounds **9** and **12** are described by simple exponential functions, as illustrated for the latter reaction in Eqs. S9-S12.

$$\frac{d[\mathbf{9}]}{dt} = -k_2[\mathbf{9}] \qquad \text{eq S9}$$

$$\frac{d[\mathbf{7}]}{dt} = \quad k_2[\mathbf{7}] \qquad \text{eq S10}$$

$$[\mathbf{9}] = [\mathbf{9}]_0 \exp^{-k_2 t} \qquad \text{eq S11}$$

$$[\mathbf{7}] = [\mathbf{9}]_0 (1 - \exp^{-k_2 t}) \qquad \text{eq S12}$$

$$\frac{d[\mathbf{12}]}{dt} = -k_2[\mathbf{12}] \qquad \text{eq S9}$$

$$\frac{d[\mathbf{13}]}{dt} = \quad k_2[\mathbf{13}] \qquad \text{eq S13}$$

$$[\mathbf{12}] = [\mathbf{12}]_0 \exp^{-k_2 t} \qquad \text{eq S14}$$

$$[\mathbf{13}] = [\mathbf{12}]_0 (1 - \exp^{-k_2 t}) \qquad \text{eq S15}$$

3. Graphs and Figures

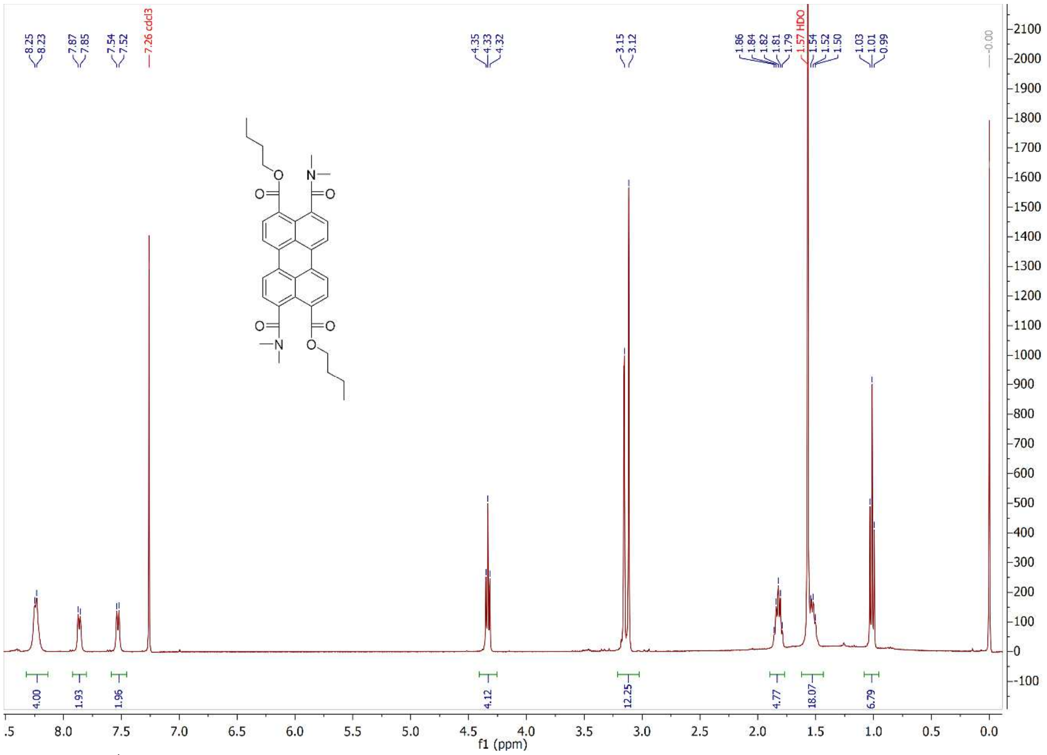

Figure S1. [1]H Spectrum of compound **6a**.

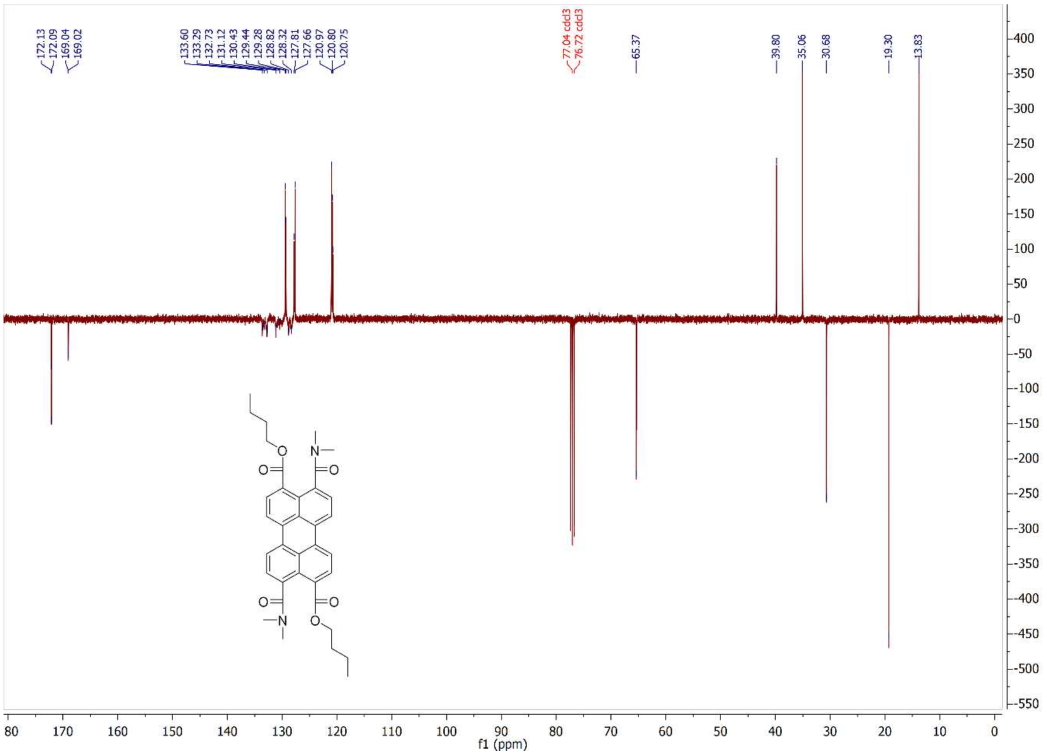

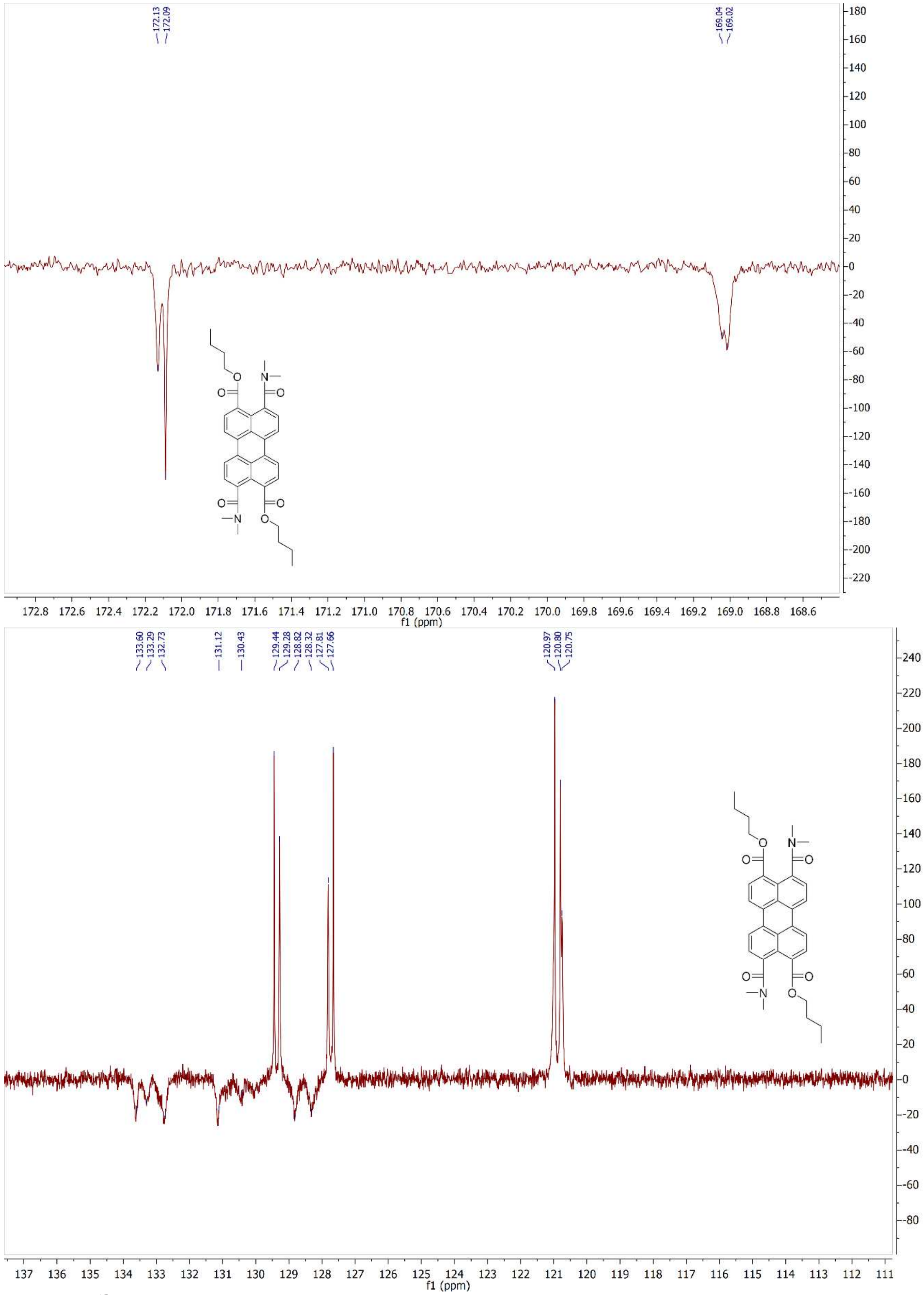

Figure S2. ^{13}C Spectrum of compound **6a**.

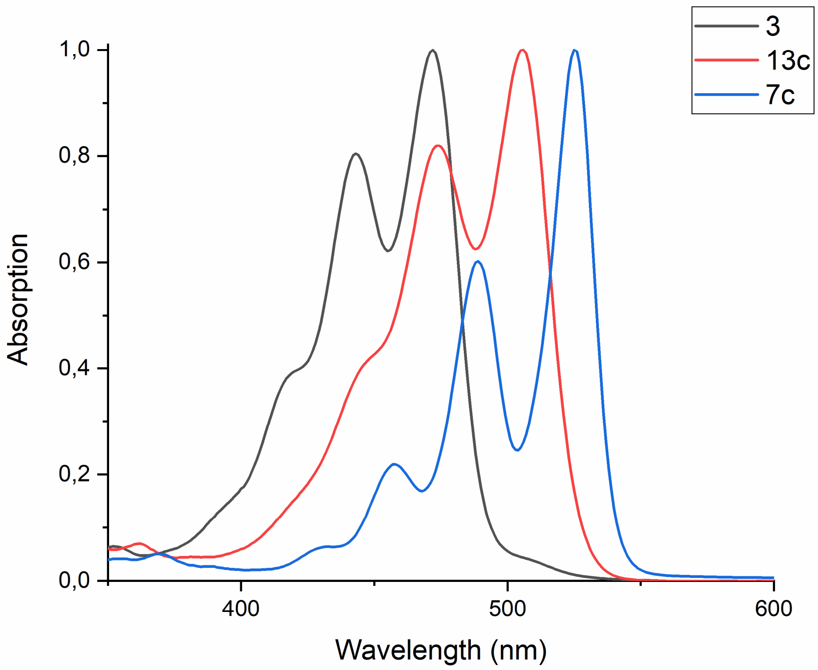

Figure S3. Normalized absorption spectra of compounds **3**, **13c** and **7c** in Chloroform. All compounds are molecularly dissolved and these spectra are representative for PTEs PMIDEs (and corresponding amic esters and amic acids) and PDIs.

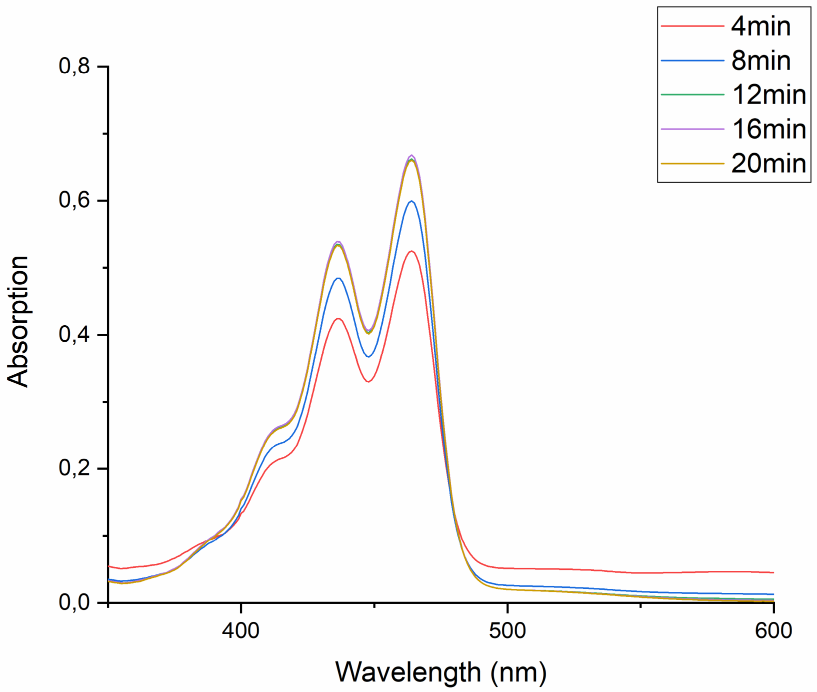

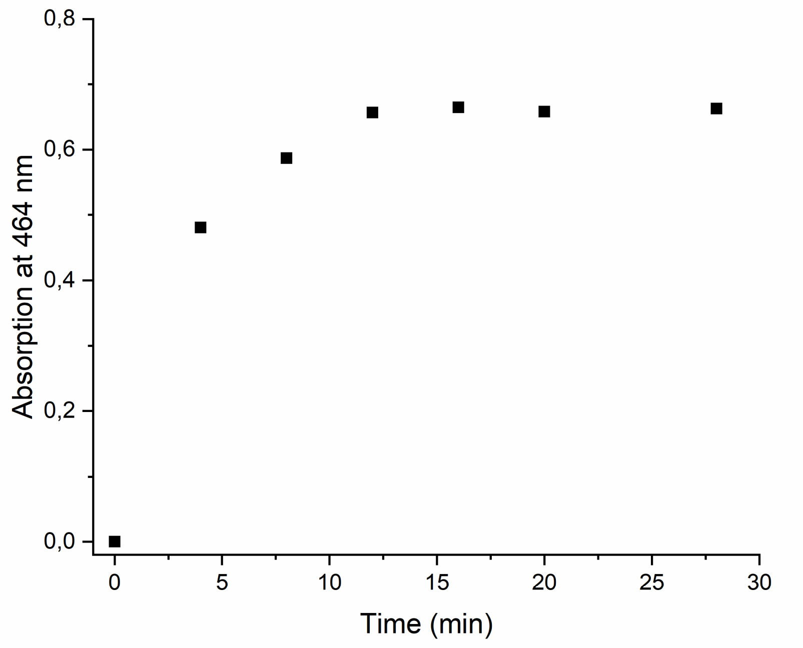

Figure S4.a: Absorption spectra taken by adding a fixed volume of the reaction of PDA with amine **4c** (Solvent DMF, base DBU, room temperature) in water at regular intervals. The compound that is visible is compound **5c**. The baseline is shifted, in particular at short reaction times, due to the presence of insoluble PDA. b: Absorption at 464 nm, corrected for the shifted baseline, as a function of time.

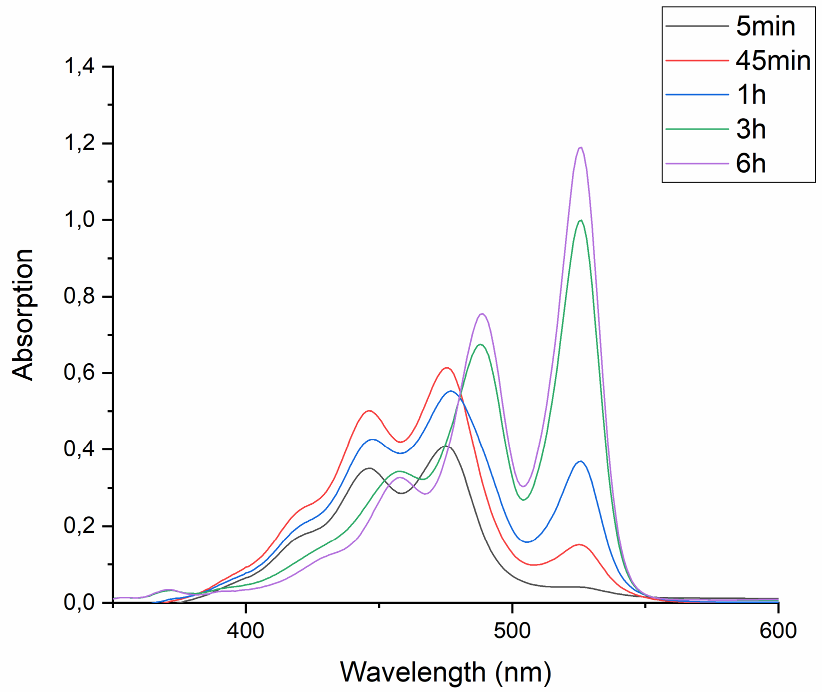

Figure S5. Absorption spectra taken by adding a fixed volume of the reaction of PDA with amine **4c** (solvent DMF, base DBU, temperature 60ºC) in chloroform at regular intervals. The compounds that are visible are the DBU salt of **5c**, **(HDBU)$_2$5c** (446 and 475 nm) and **7c** (489 and 526 nm).

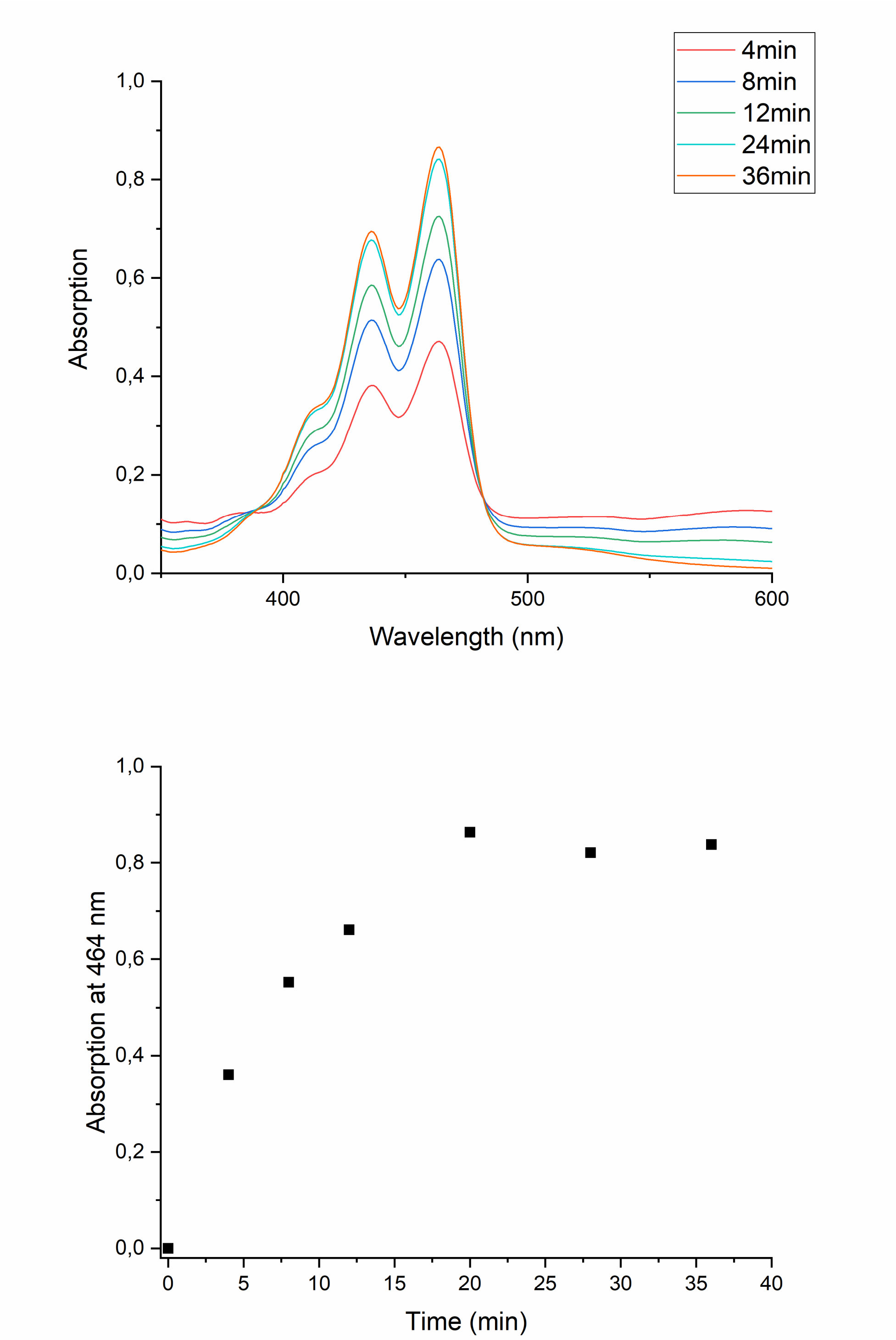

Figure S6. a: Absorption spectra taken by adding a fixed volume of the reaction of PDA with amine **4e** (Solvent DMSO, base K_2CO_3, room temperature) in water at regular intervals. The compound that is visible is compound **5e**. The baseline is shifted due to the presence of insoluble PDA. b: Absorption at 464 nm, corrected for the shifted baseline, as a function of time.

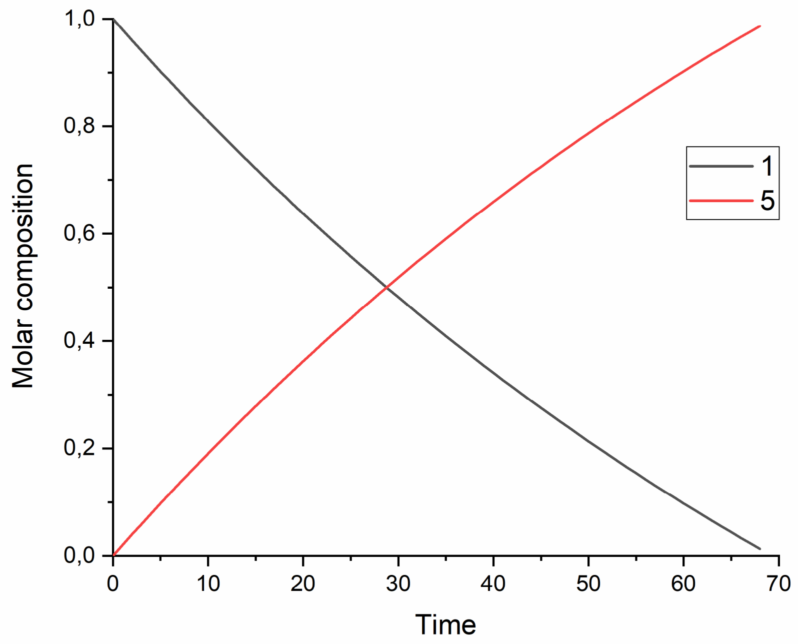

Figure S7. Consumption of PDA **1** and formation of diamic acid **5** according to equations 6 and 9. The time axis has arbitrary units.

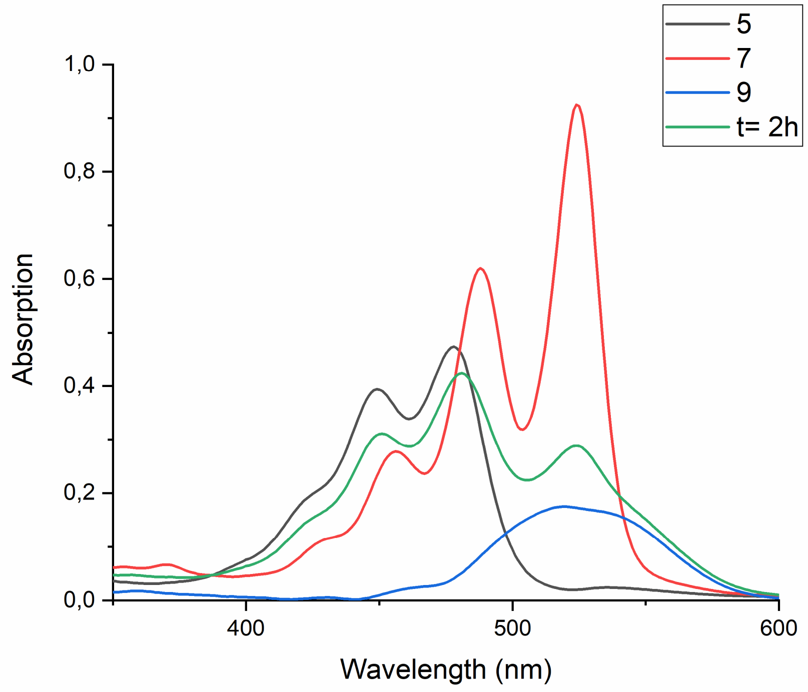

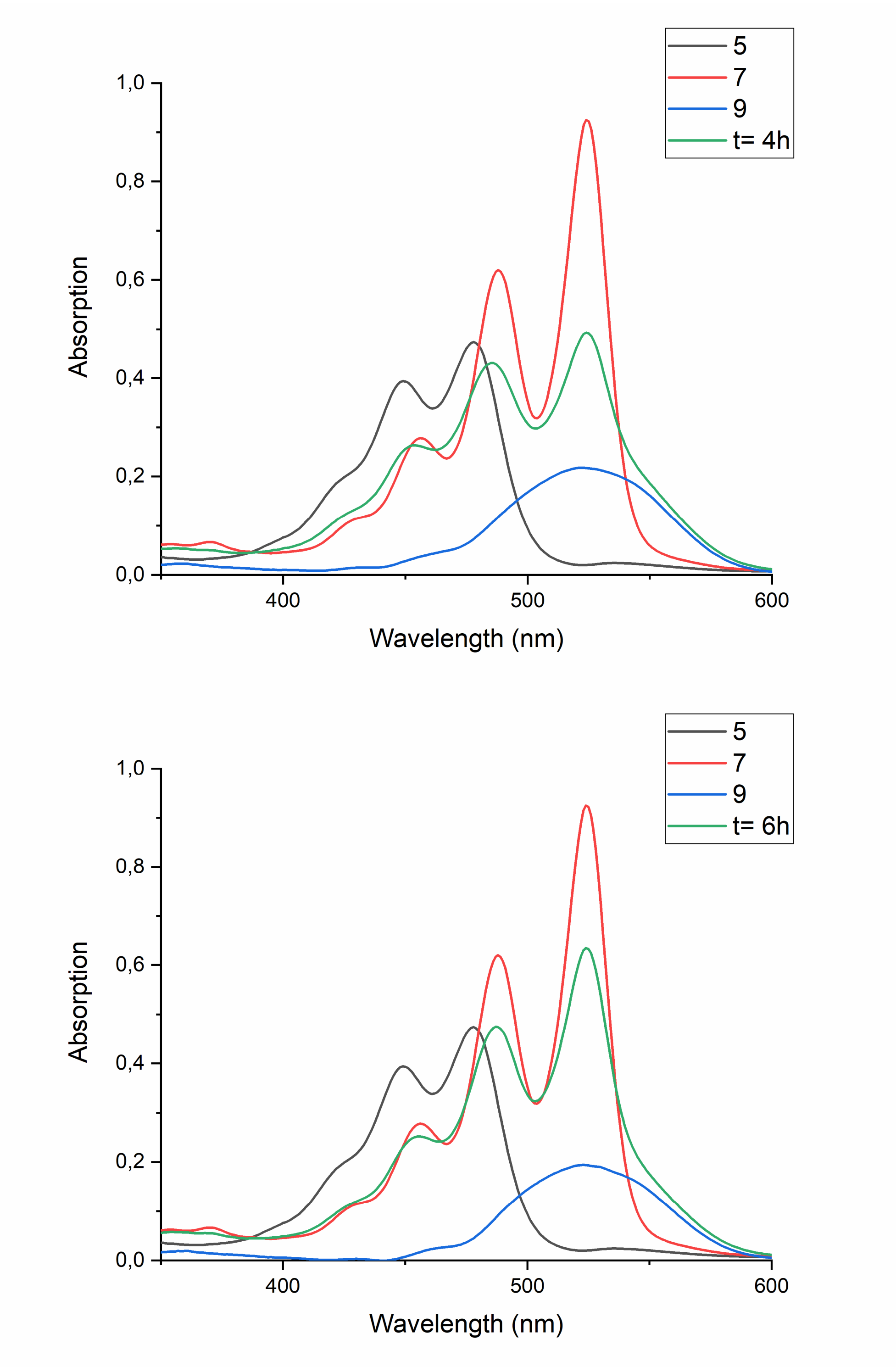

Figure S8. Spectra taken from the imidization of the DBU salt of **5c** in DMF taken 2, 4 and 6 hours, after diluting the reaction mixture (in green). The spectra of starting compound, the DBU salt of **5c** (black) and final product **7c** (red) are depicted as well. After subtracting the contributions of starting compound, the DBU salt of **5c**, and product **7c** from that of the reaction mixture, the broad, the contributions of the intermediate, the DBU salt of **9c**, are revealed (blue). In this manner the composition of the reaction mixture is determined.

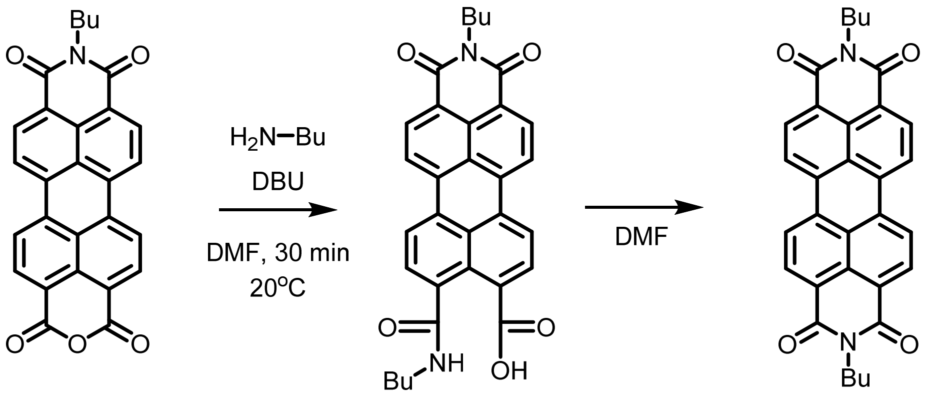

Scheme S1. Imidization of compound **15** using butylamine **4c** via amic acid **9c**. The last step of this reaction has been monitored using absorption spectroscopy.

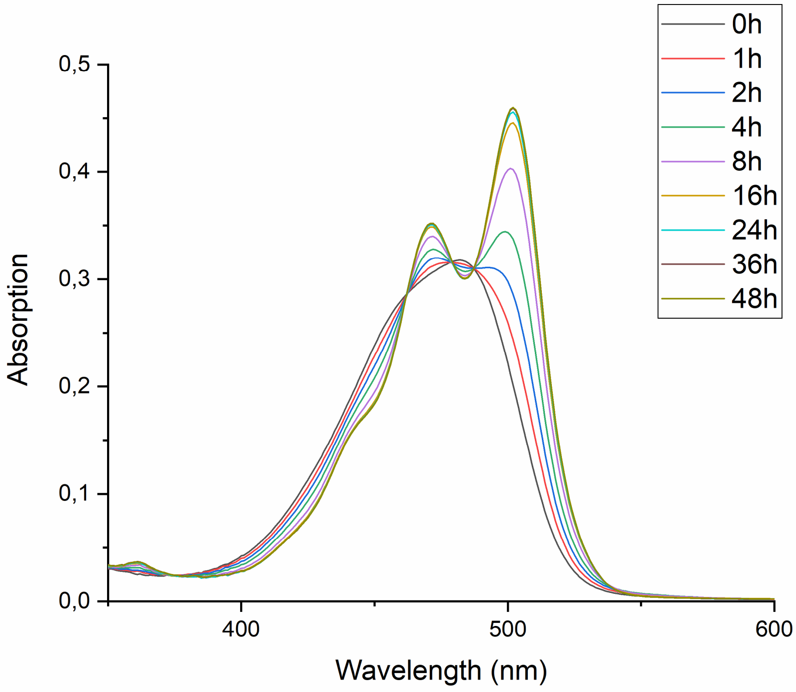

Figure S9. Absorption spectra as a function of time of the reaction of the DBU salt of amic acid **12c** in DMF at concentrations around $1*10^{-5}$ M. Isosbestic points are observed at 462, 479 and 487 nm.

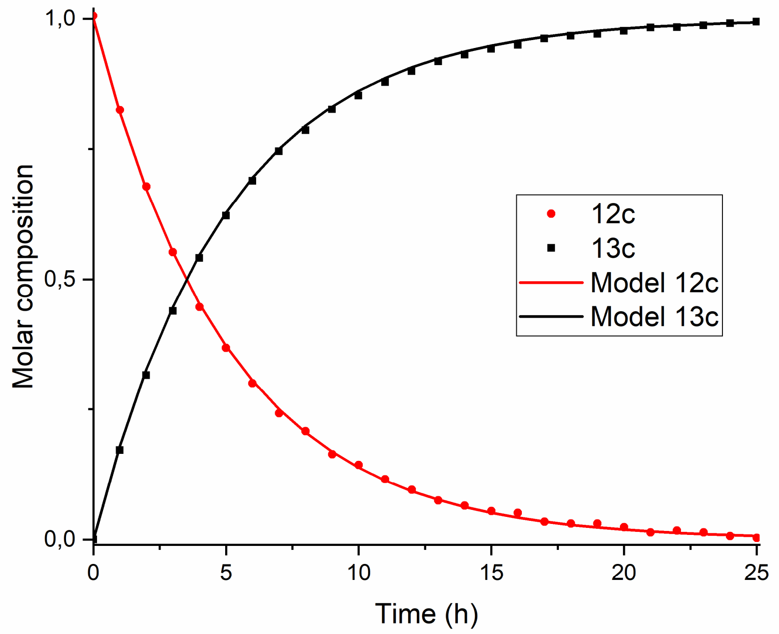

Figure S10. Composition of the reaction mixture obtained from the data displayed in Figure S9. Solid lines represent the theoretical fitting curves obtained using a first-order decay obtained using equations S14 and S15.

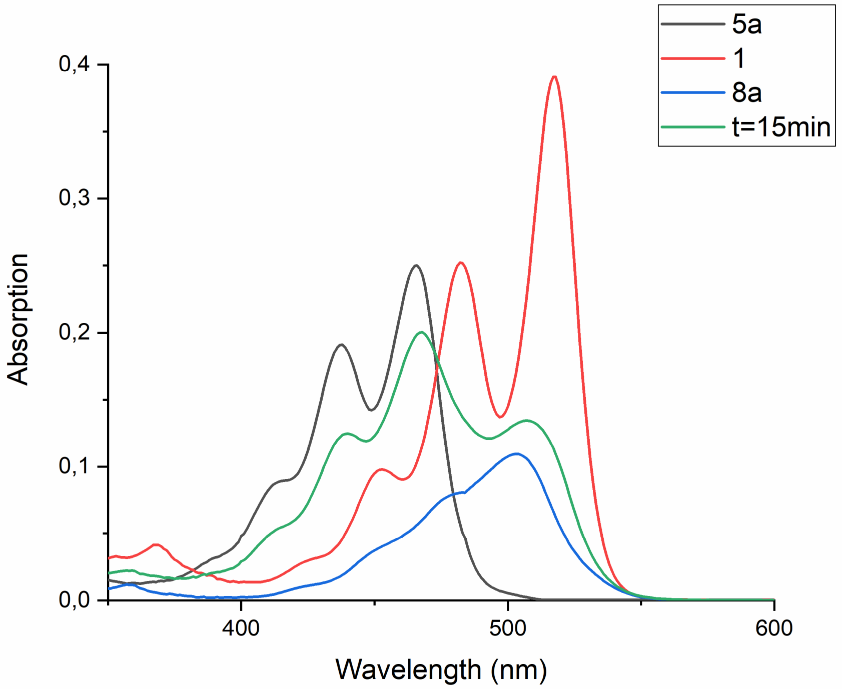

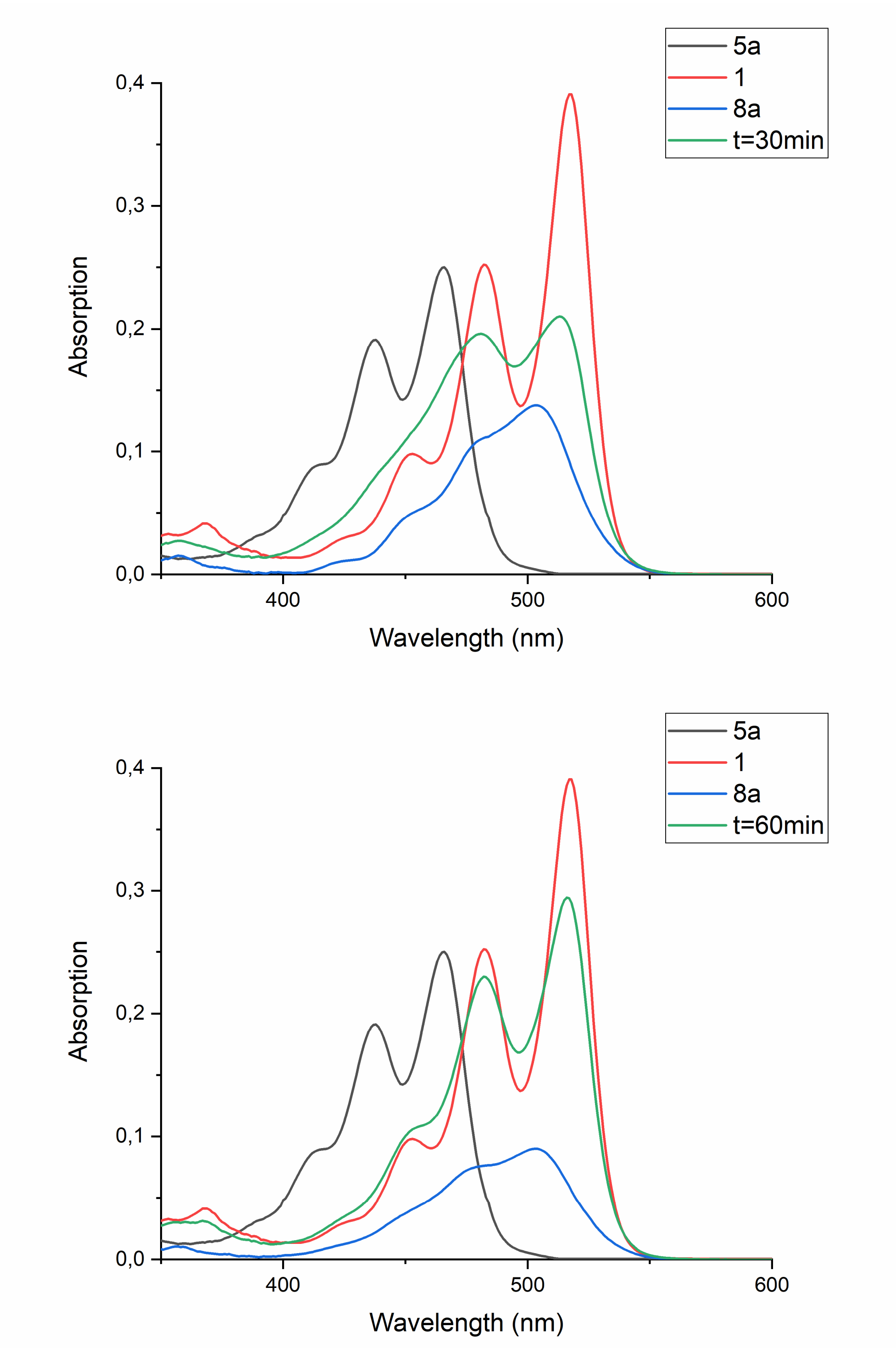

Figure S11. Spectrum taken from the acid hydrolysis of compound **5a** in DMF after 15, 30 and 60 minutes of reaction (blue). The spectra of starting compound **5a** (black) and final product **1** (red) are depicted as well. are After subtracting the contributions of starting compound **5a** and product **1** from the absorption spectra of the reaction mixture (green), the contributions of the intermediate **8a** are revealed (blue).

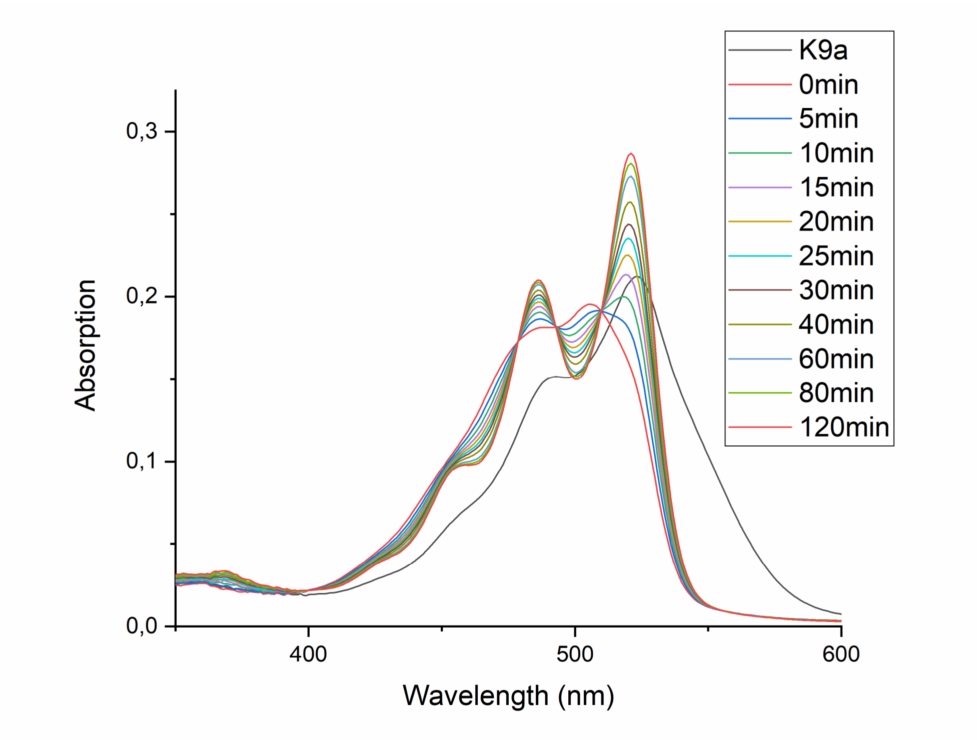

Figure S12. Absorption spectra of compound **K9a** (black) in DMF and **9a** (red), formed after acidification with 1 drop 1N HCl. In the time-dependent absorption spectra the reaction from **9a** (λ_{max}= 506 nm) to compound **15** (λ_{max}= 521 and 486 nm) is visible. Please note that the first spectrum after acidification already contains (traces of) compound **15**. Isosbestic points are observed at 479, 493 and 510 nm.

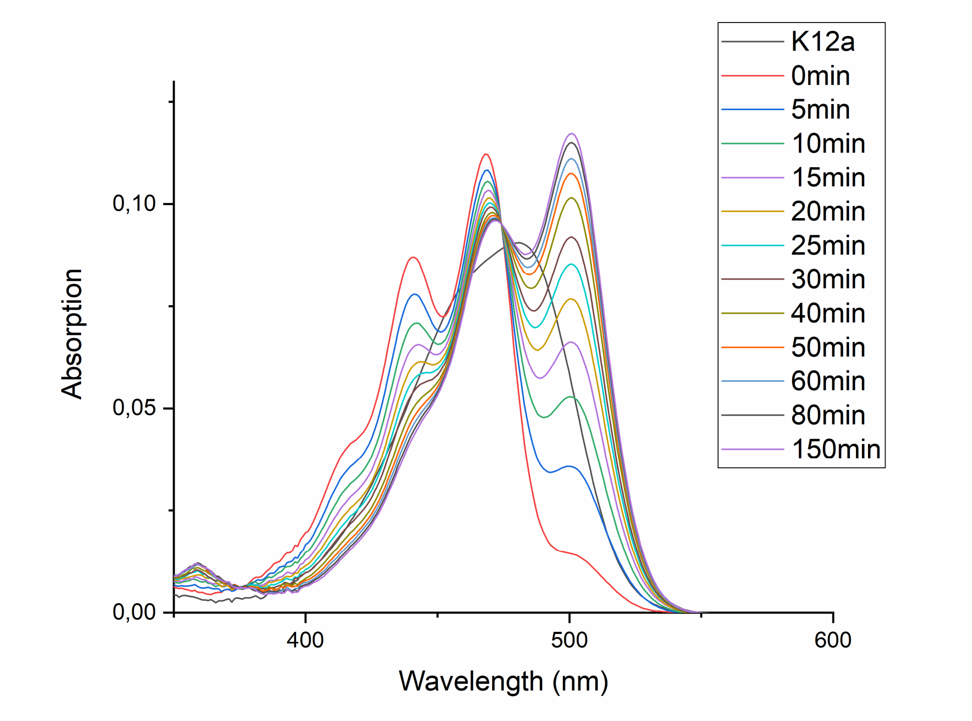

Figure S13. Absorption spectra of compound **K12a** (black) in DMF and **12a** (red), formed after acidification with 1 drop 1N HCl. In the time-dependent absorption spectra the reaction from **12a** (λ_{max}= 468 and 441 nm) to compound **11** (λ_{max}= 472 and 501 nm) is visible. Please note that the first spectrum after acidification already contains (traces of) compound **11**. An isosbestic points is observed at 475 nm.

4. Syntheses

Table S1

Entry	Perylene anhydride	amine	base	solvent	Reaction conditions	Yield, in % (conversion)
S1	PDA (**1**) 0.5 mmol	**4b** 2 mmol	DBU 2 mmol	DMF 4 ml	168h RT	**7b** 87%
S2	PDA (**1**) 0.5 mmol	**4b** 2 mmol	$Zn(Ac)_2$, 0.6 mmol DBU, 2 mmol	DMF 4 ml	24h RT	**7b** 52%
S3	PDA (**1**) 0.5 mmol	**4b** 2 mmol	$Zn(Ac)_2$, 0.6 mmol DBU, 2 mmol	DMF 4 ml	24h 60˚C	**7b** 78%
S4	PDA (**1**) 0.5 mmol	**4b** 2 mmol	TEA 2 mmol	DMSO 4 ml	24h 60˚C	**7b** 20%
S5	PDA (**1**) 0.5 mmol	**4b** 2 mmol	K_2CO_3 2 mmol	DMSO 4 ml	24h 60˚C	**7b** 32%
S6	PDA (**1**) 0.5 mmol	**4c** 2 mmol	DBU 2 mmol	DMSO 4 ml	24h RT	**7c** 52%
S7	PDA (**1**) 0.5 mmol	**4d** 2 mmol	DBU 2 mmol	DMF 4 ml	24h RT	**7d** 89%
S8	PDA (**1**) 0.5 mmol	**4f** 2 mmol	DBU 2 mmol	DMF 4 ml	24h RT	**7f** 66%

5. UV-Vis Absorption and Fluorescence Spectra

Table S3. Spectral data of amic acids, amic acid salts and amic esters

Compound	Solvent	λ_{abs}	λ_{em}	Φ_F
**3**	EtOH	468, 440	485, 515	0.86
**K$_2$5a**	DMF	475, 443	487	0.16
**K$_2$5a**	EtOH	466, 438	477.5, 509	0.93
**K$_2$5a**	Water[a]	463, 435	475, 505	1.0
**6a**	CHL	466, 438	482, 511.5	0.88
**6a**	EtOH	463, 435	479, 507	0.85
**K9a**	DMF	521	581	0.14
**K9a**	EtOH	513	584	0.65
**K9a**	Water[a]	505	591	0.19
**10a**	CHL	507, 477,5	530, 566	0.73
**10a**	EtOH	501, 472	542	0.66
**K12a**	DMF	481	522	0.19
**K12a**	EtOH	472, 447	522	0.96
**K12a**	Water[a]	468, 444	524	~0.6[b]
**14a**	CHL	470, 441.5	487, 517	0.92
**14a**	EtOH	466, 438	484, 512	0.87

a: 0.01 M K_2CO_3 b: inaccurate measurement due to low solubility

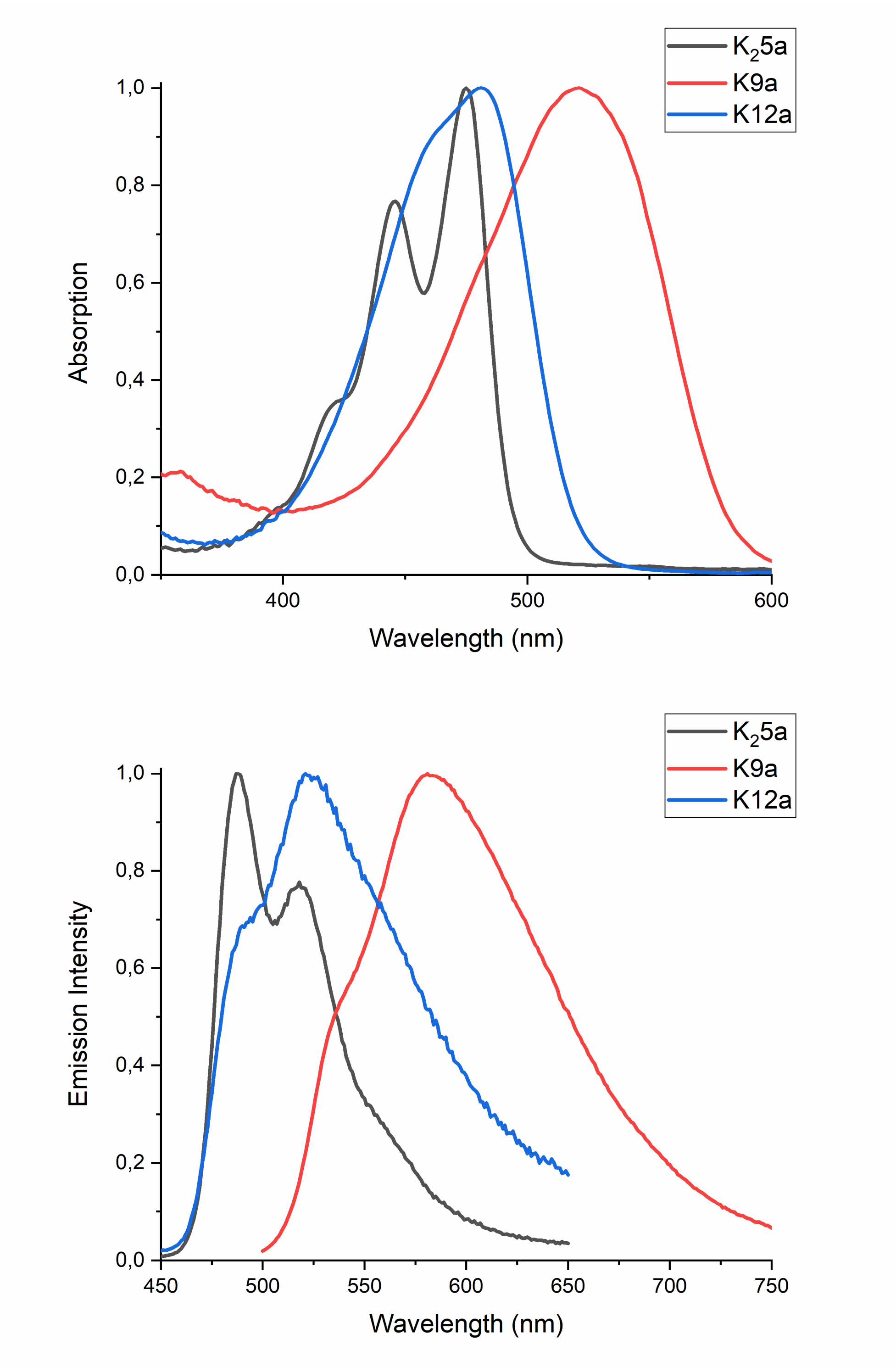

Figure S14. a) Normalized UV-Vis absorption and b) normalized fluorescence emission spectra of the amic acid salts **$K_2$5a**, **K9a** and **K12a** in DMF.

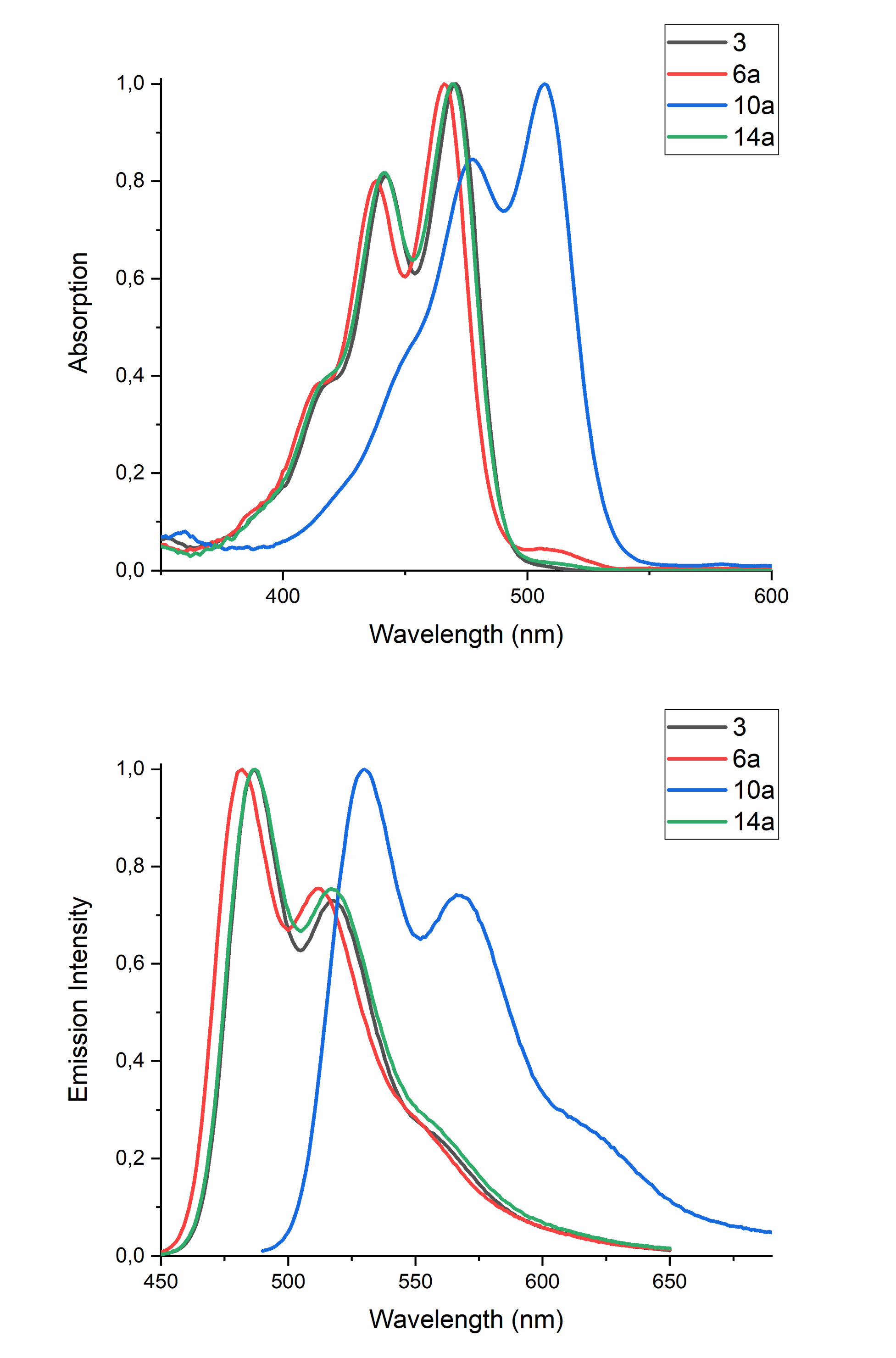

Figure S15. a) Normalized UV-Vis absorption and b) normalized fluorescence emission spectra of amic esters **6a**, **10a** and **14a** along with PTE **3** in chloroform.

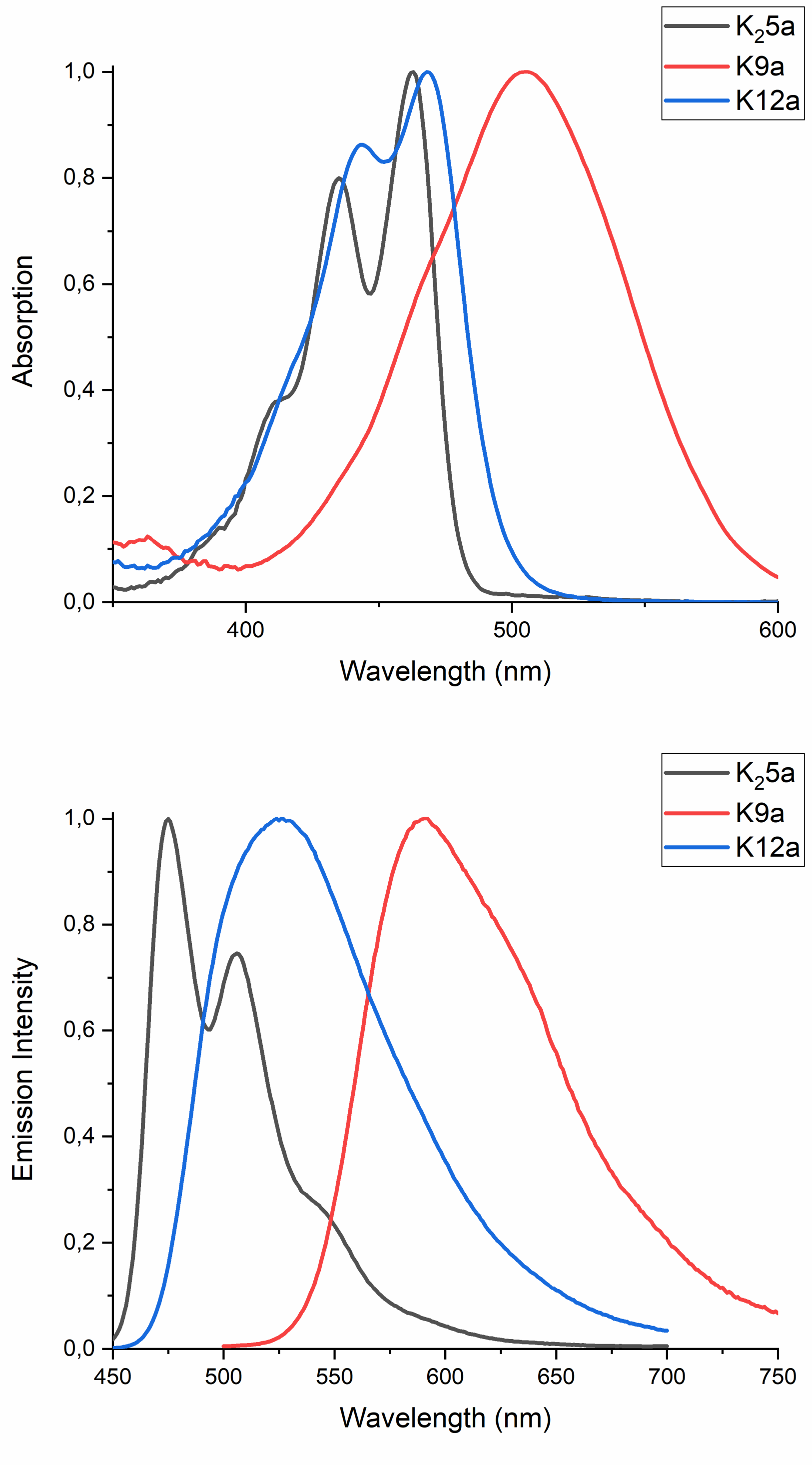

Figure S16. a) Normalized UV-Vis absorption and b) normalized fluorescence emission spectra of the amic acid salts **$K_2$5a**, **K9a** and **K12a** in water.

6 NMR and Mass Spectra

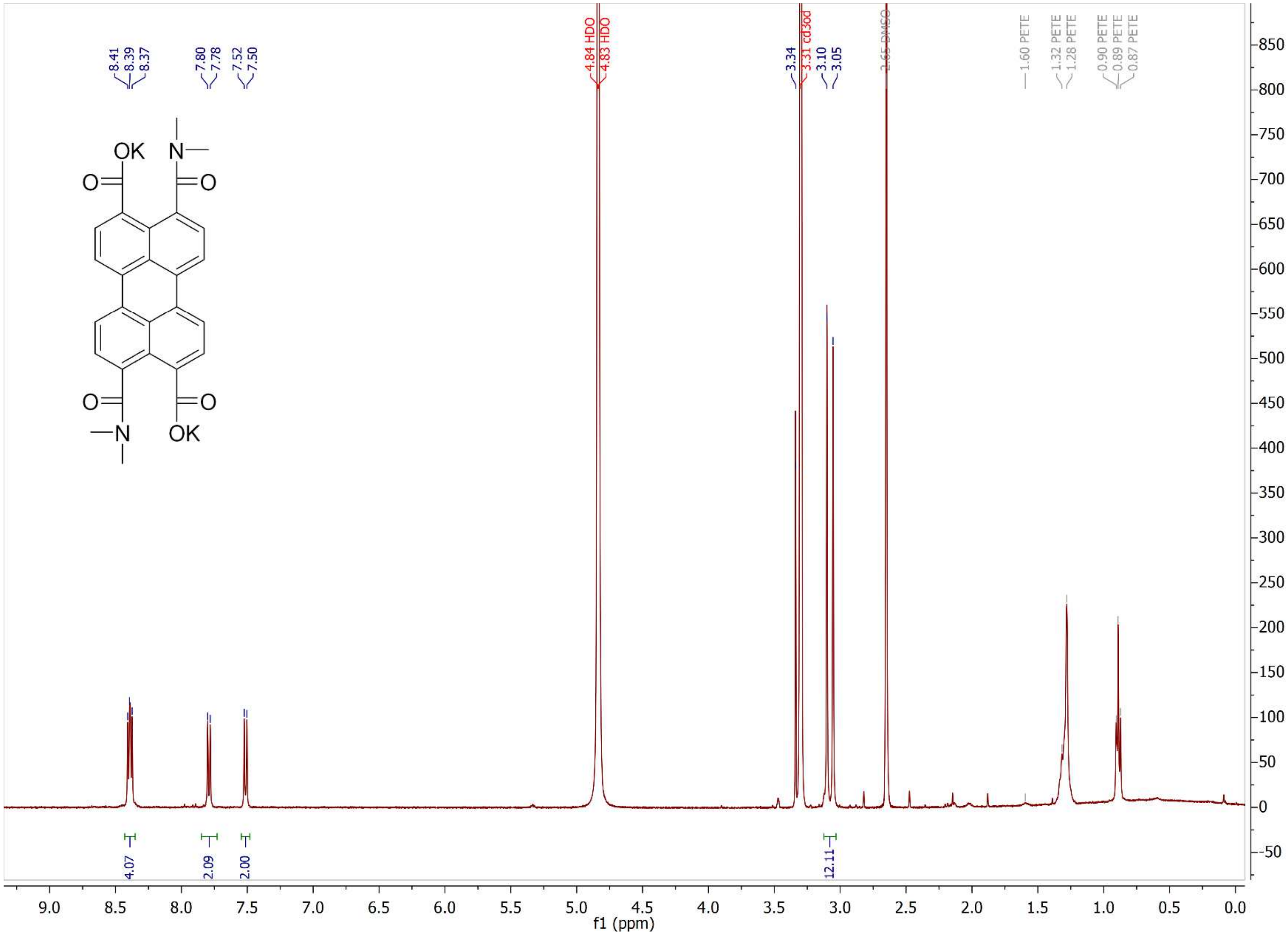

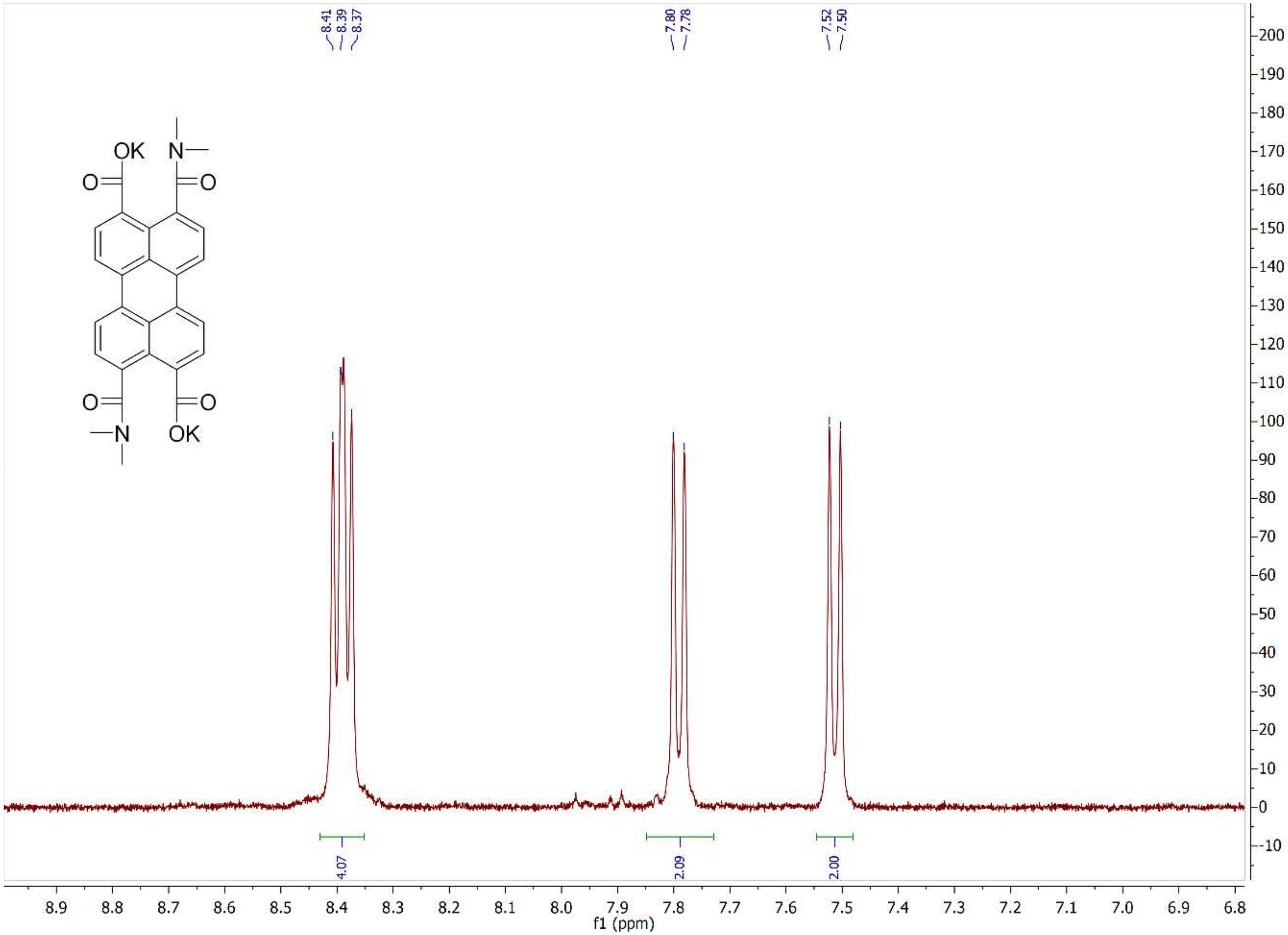

Figure S17. ^{1}H NMR spectrum of **5a** in MeOD. Contains DMSO and impurities from petroleum ether.

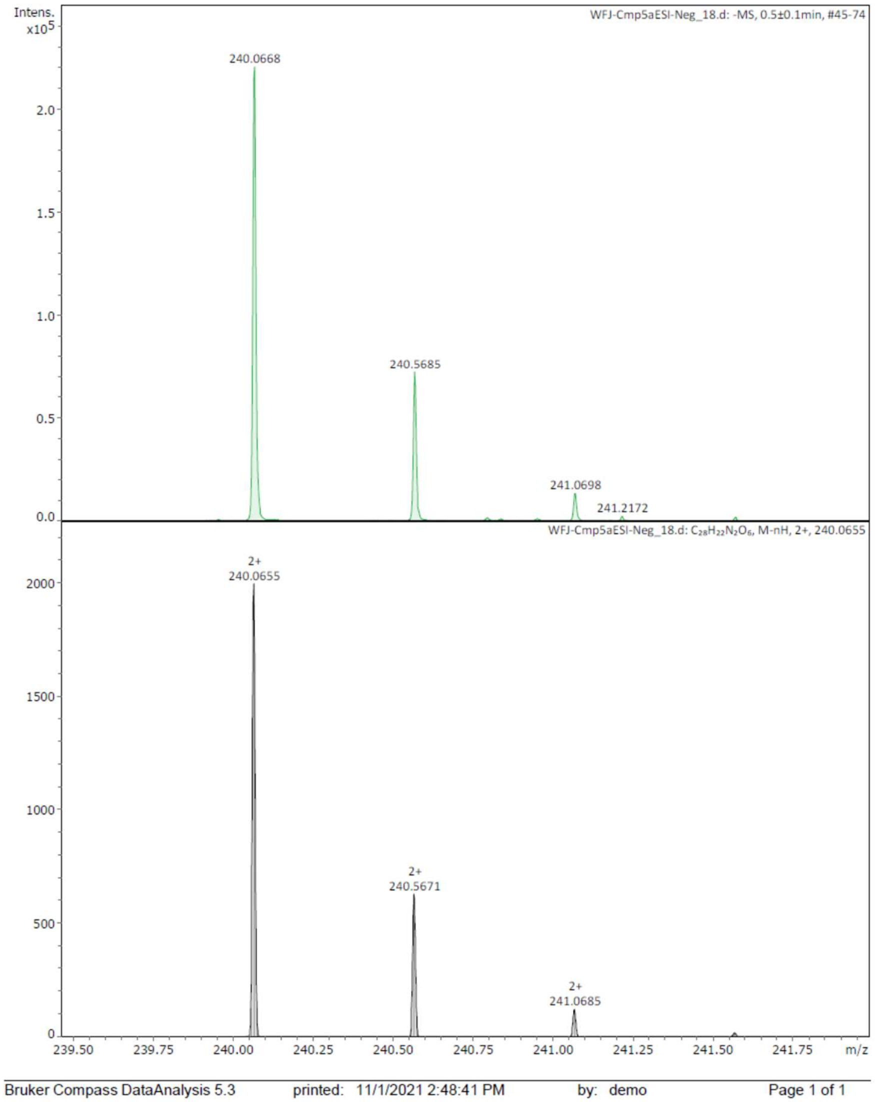
Intens.
x10^5
WFJ-Cmp5aESI-Neg_18.d: -MS, 0.5±0.1min, #45-74
240.0668
240.5685
241.0698
241.2172
2.0
1.5
1.0
0.5
0.0
WFJ-Cmp5aESI-Neg_18.d: $C_{28}H_{22}N_2O_6$, M-nH, 2+, 240.0655
2+
240.0655
2+
240.5671
2+
241.0685
2000
1500
1000
500
0
239.50
239.75
240.00
240.25
240.50
240.75
241.00
241.25
241.50
241.75
m/z
Bruker Compass DataAnalysis 5.3
printed: 11/1/2021 2:48:41 PM
by: demo
Page 1 of 1

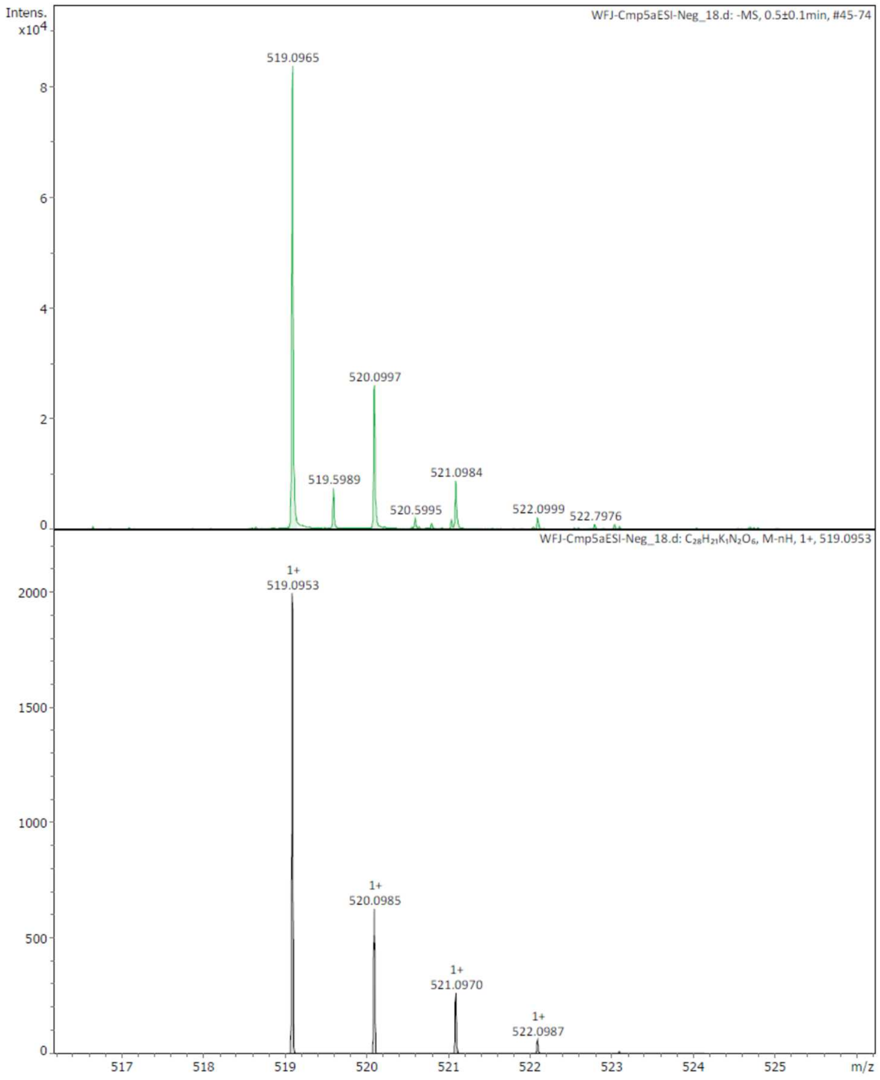

Bruker Compass DataAnalysis 5.3 printed: 11/1/2021 2:48:07 PM by: demo Page 1 of 1

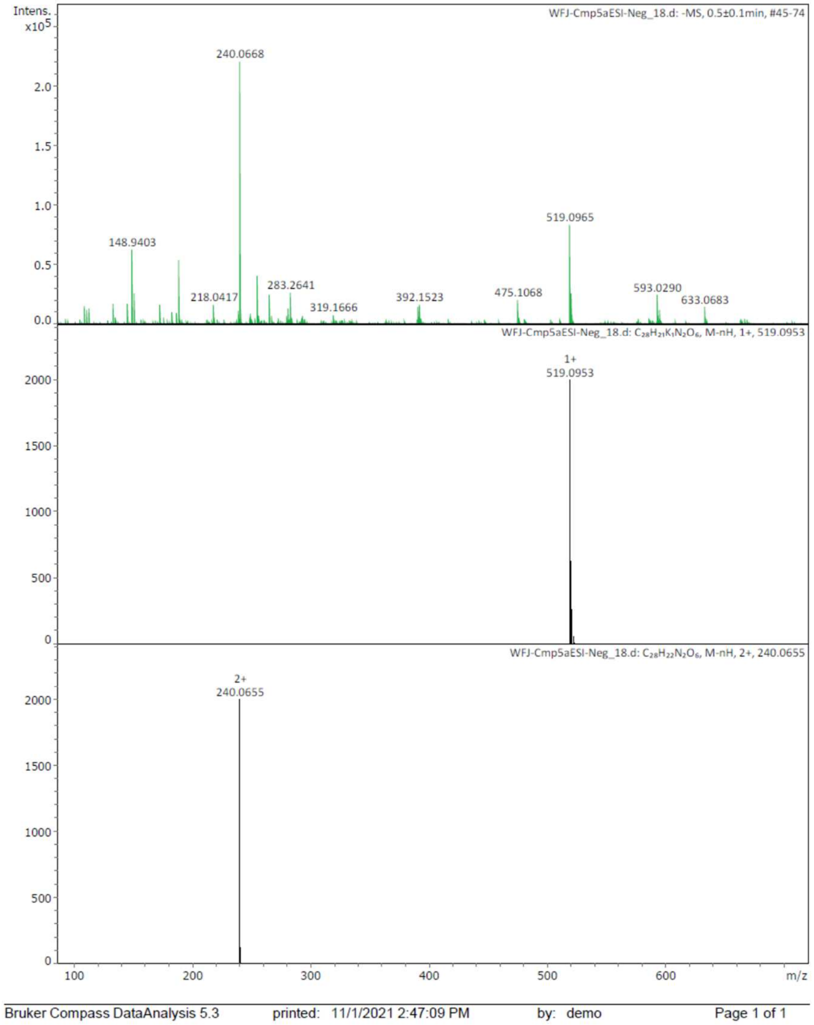

Figure S18. Mass spectrum of **5a**. Calculated masses are: **5a**$^{2-}$; 481.14, **K5a**$^{-}$; 963.30.

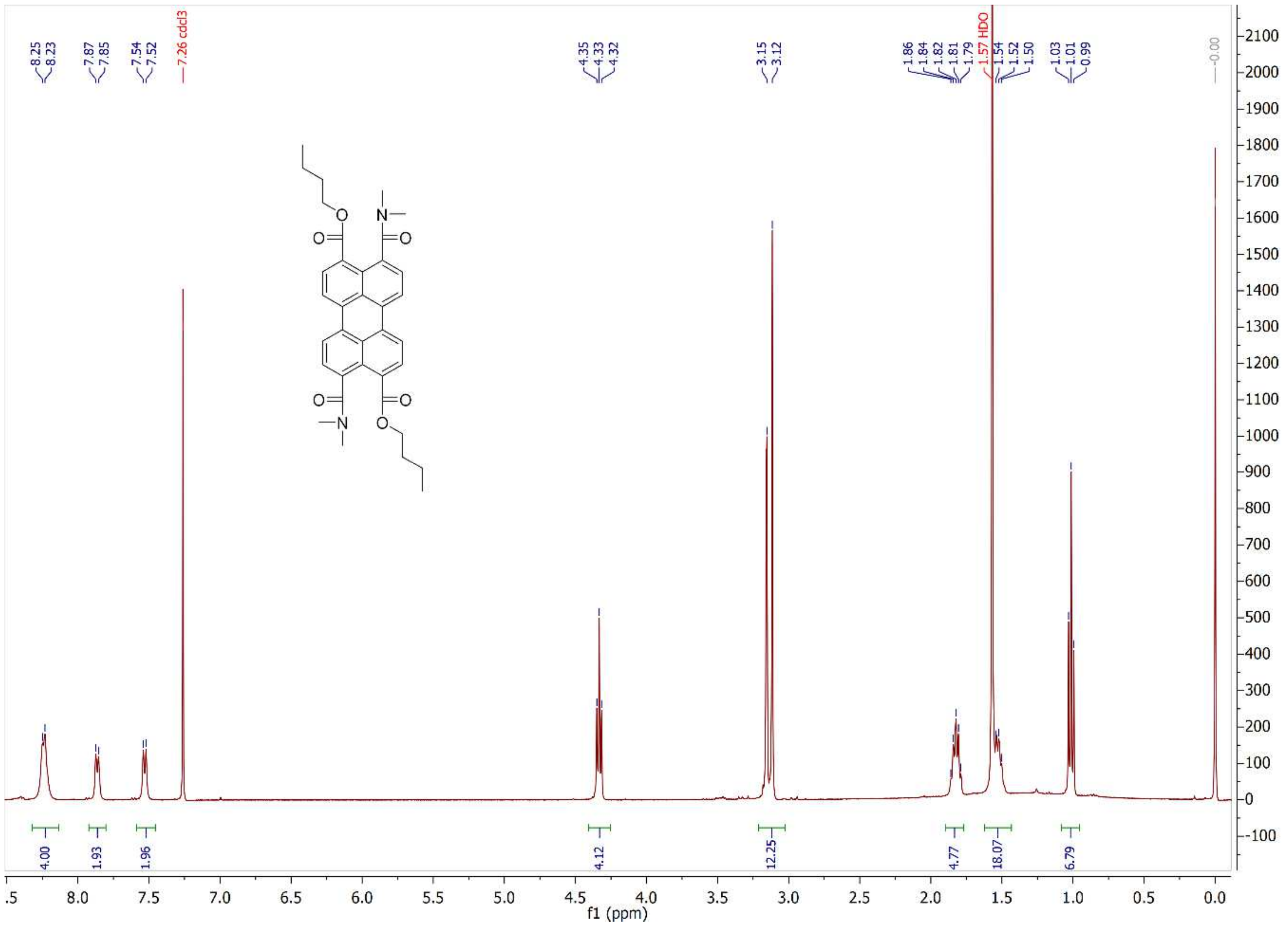
8.25
8.23
7.87
7.85
7.54
7.52
7.26 cdcl3
4.35
4.33
4.32
3.15
3.12
1.86
1.84
1.82
1.81
1.79
1.57 HDO
1.54
1.52
1.50
1.03
1.01
0.99
-0.00
4.00
1.93
1.96
4.12
12.25
4.77
18.07
6.79
f1 (ppm)

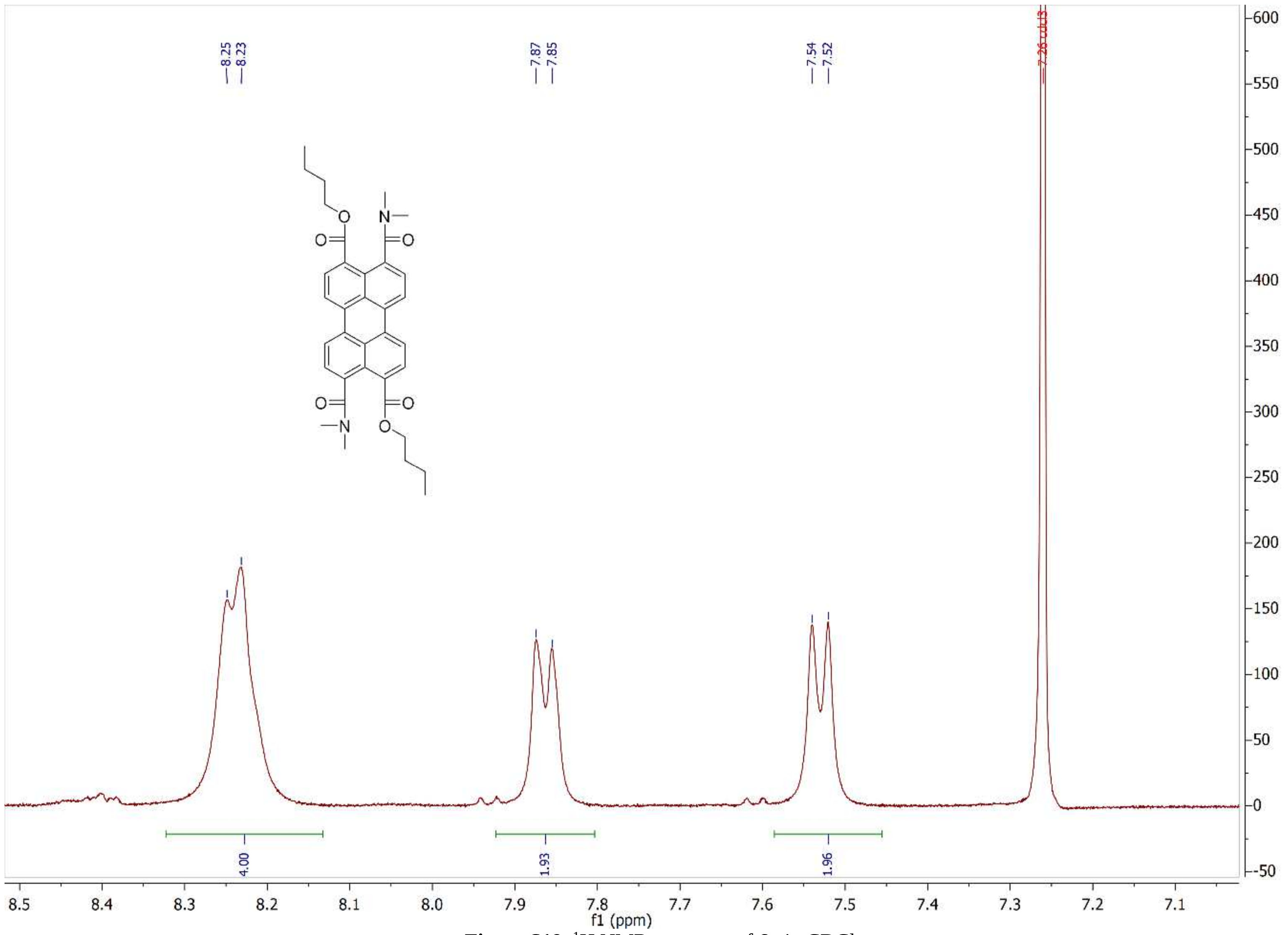

Figure S19. ^{1}H NMR spectrum of **6a** in $CDCl_3$.

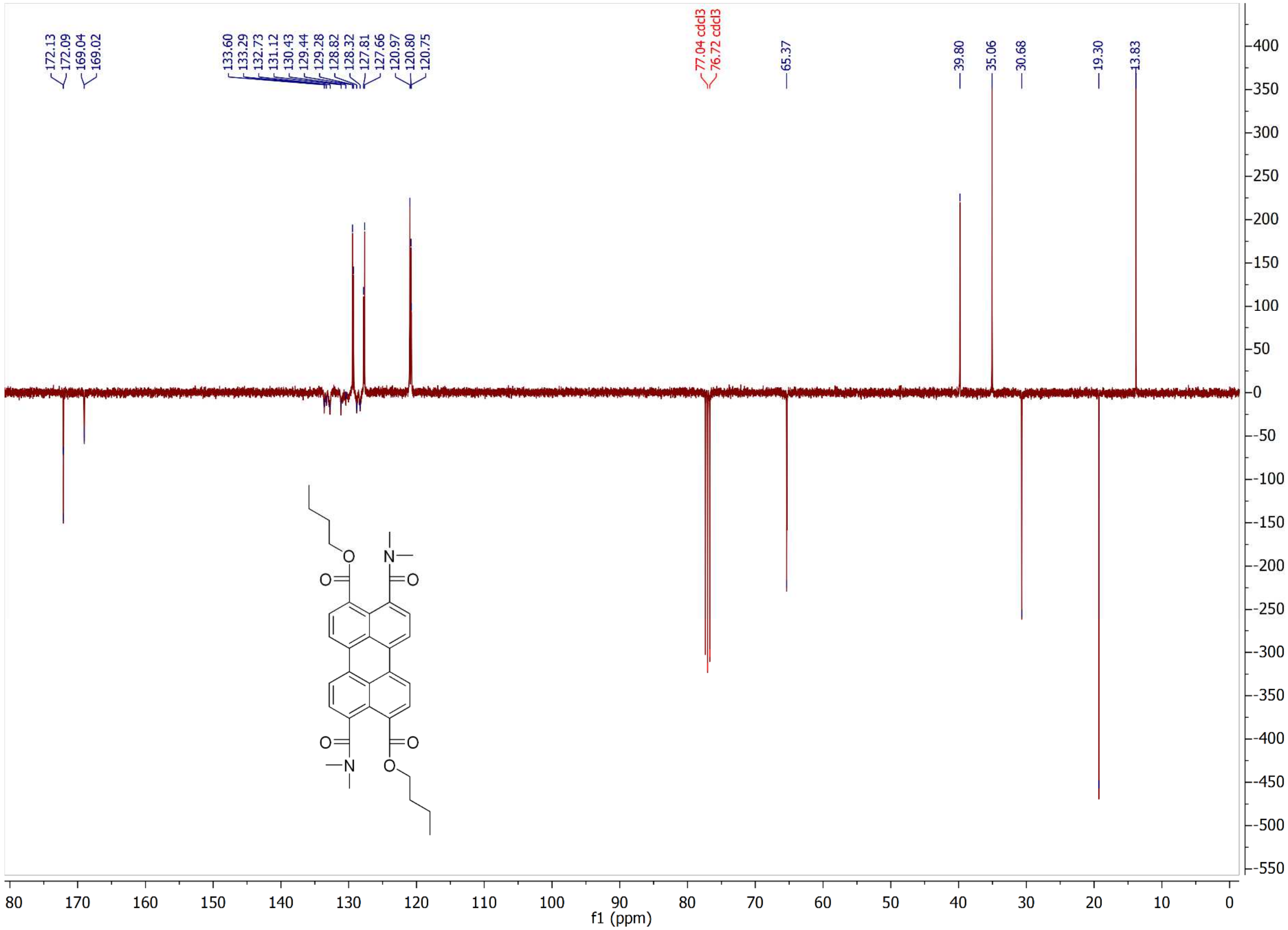

172.13
172.09
169.04
169.02
133.60
133.29
132.73
131.12
130.43
129.44
129.28
128.82
128.32
127.81
127.66
120.97
120.80
120.75
77.04 cdcl3
76.72 cdcl3
65.37
39.80
35.06
30.68
19.30
13.83
f1 (ppm)

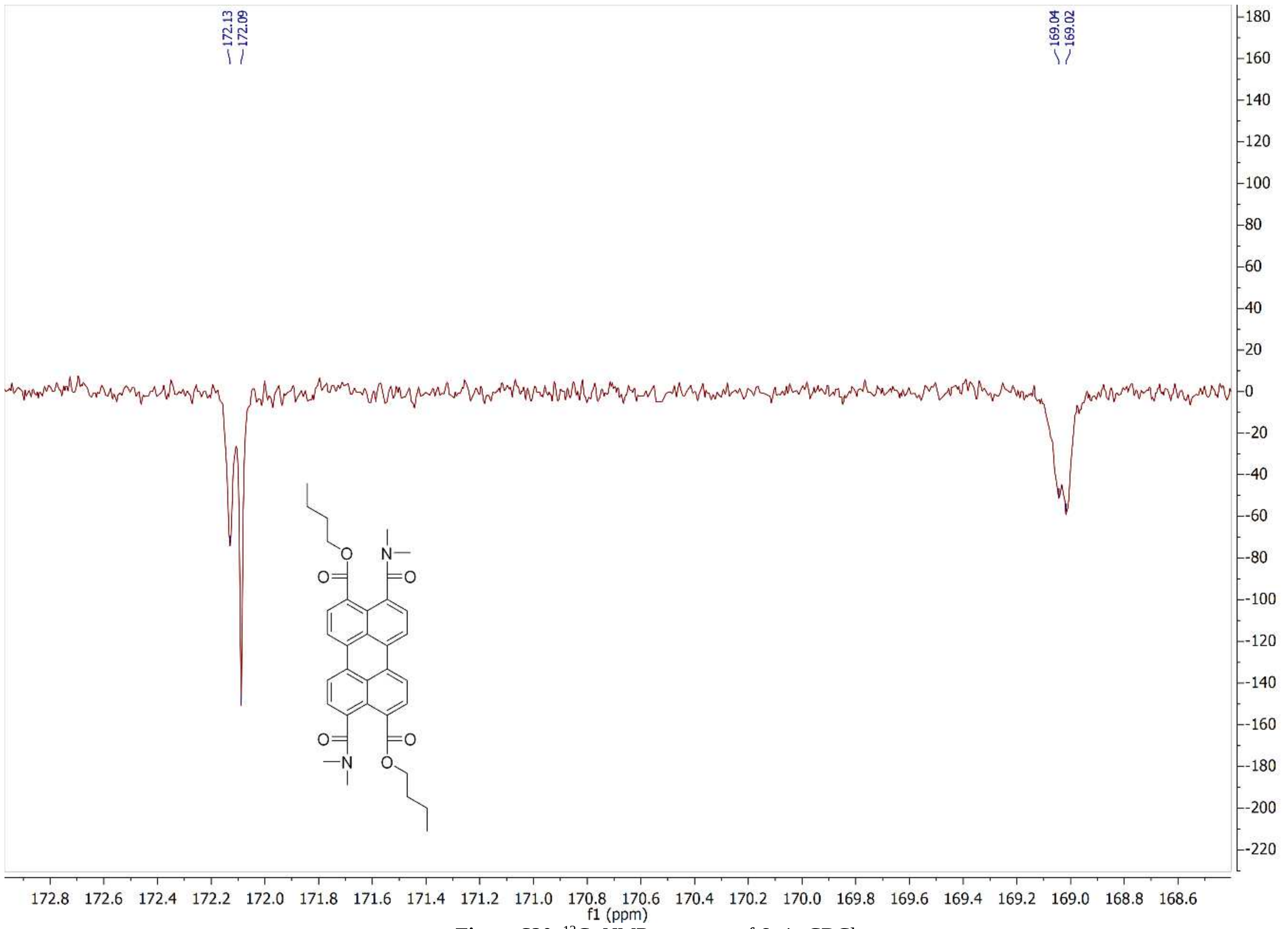

Figure S20. ^{13}C- NMR spectrum of **6a** in $CDCl_3$.

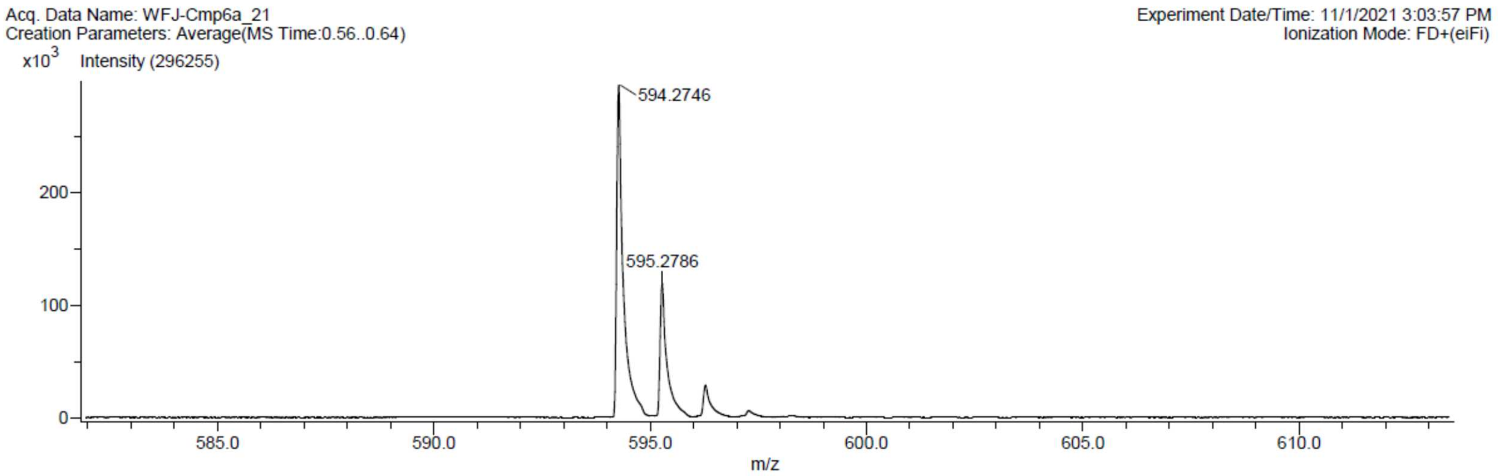
Acq. Data Name: WFJ-Cmp6a_21
Creation Parameters: Average(MS Time:0.56..0.64)
Experiment Date/Time: 11/1/2021 3:03:57 PM
Ionization Mode: FD+(eiFi)
x10^3 Intensity (296255)
594.2746
595.2786
200
100
0
585.0
590.0
595.0
600.0
605.0
610.0
m/z

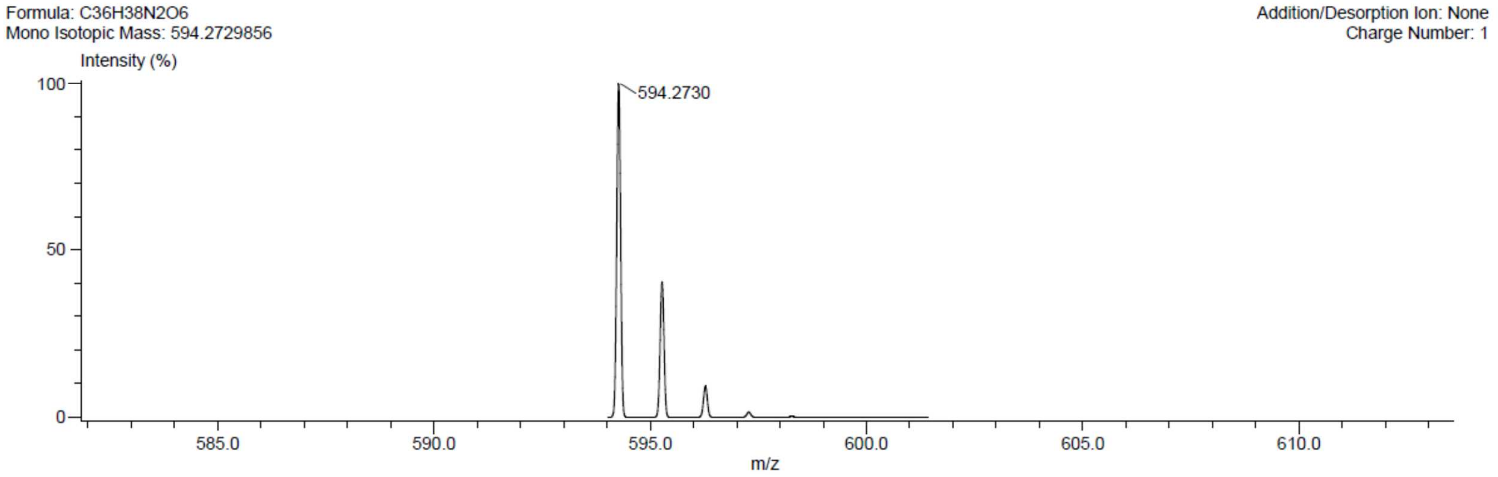
Formula: C36H38N2O6
Mono Isotopic Mass: 594.2729856
Addition/Desorption Ion: None
Charge Number: 1
Intensity (%)
594.2730
100
50
0
585.0
590.0
595.0
600.0
605.0
610.0
m/z

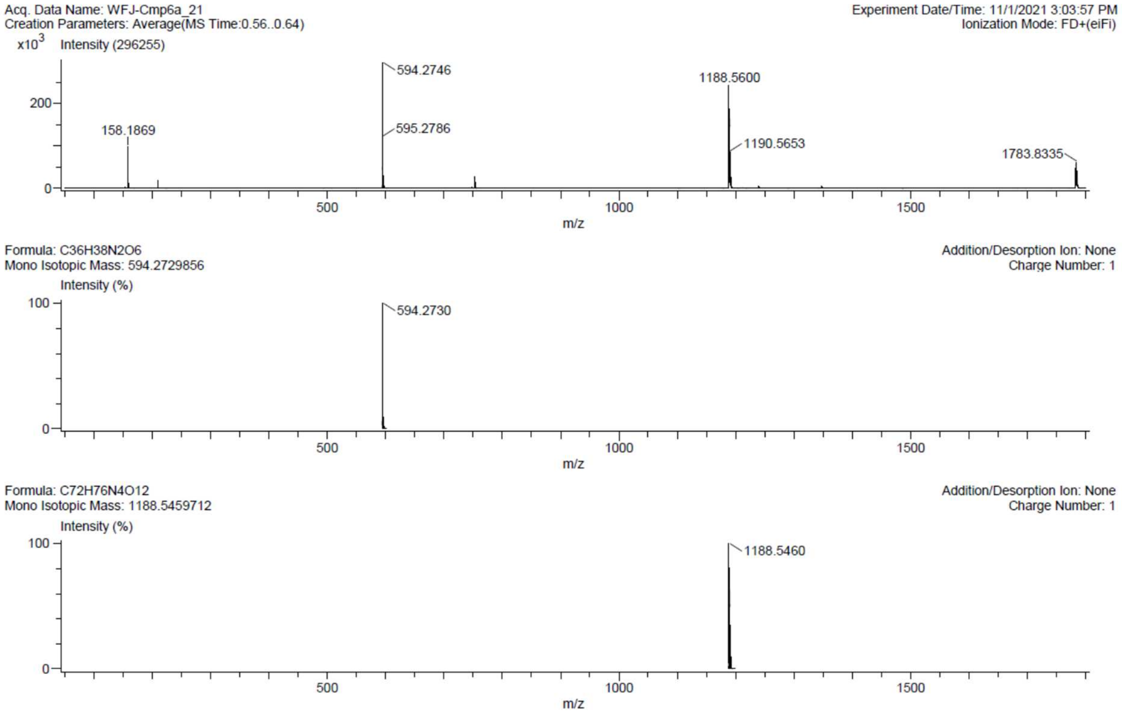

Figure S21. Mass spectrum of **6a**. Calculated **6a**; 594.2730, **6a$_2$**=1188.5460, **6a$_3$**= 1783.8222.

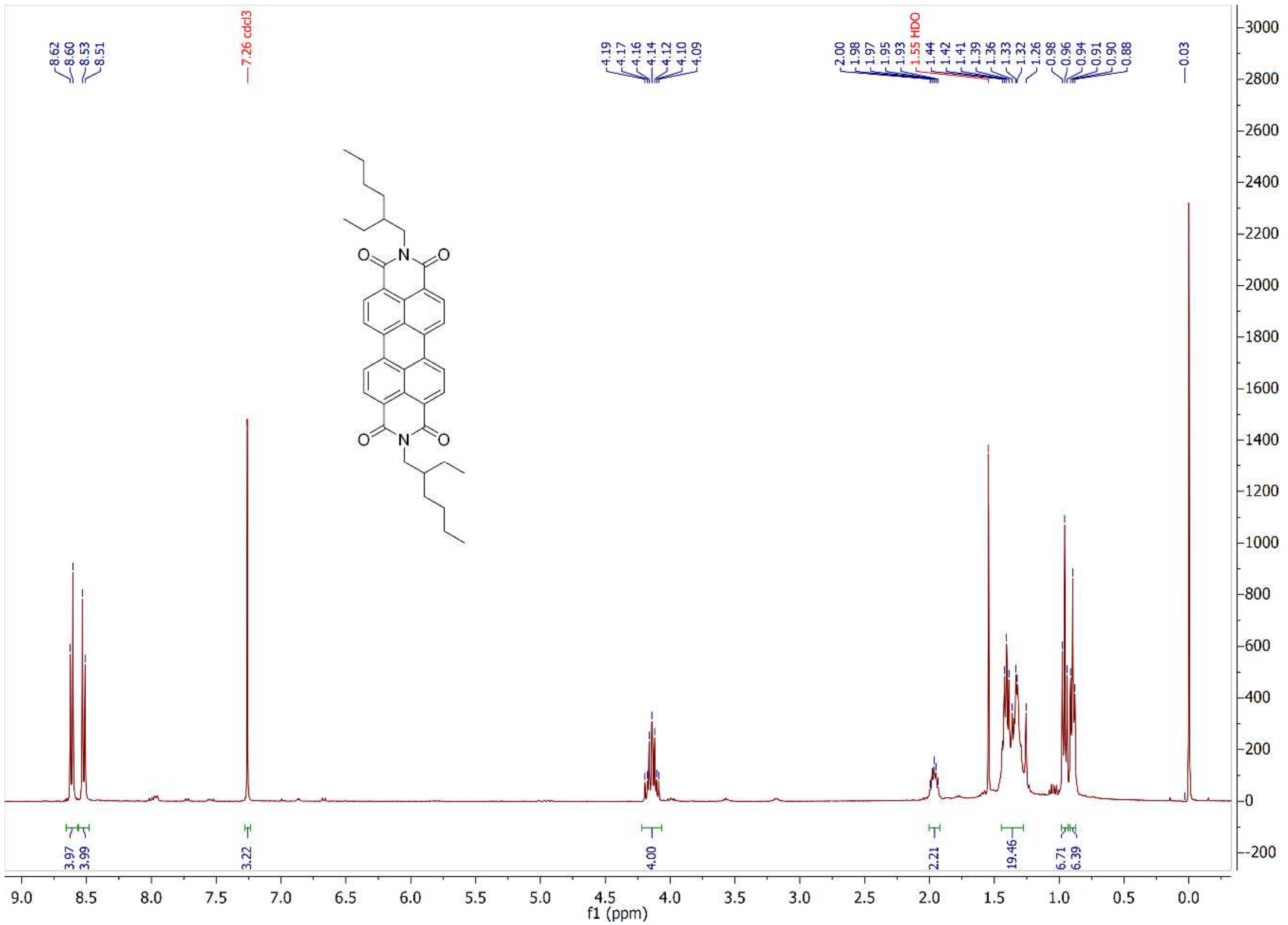
8.62
8.60
8.53
8.51
7.26 cdcl3
4.19
4.17
4.16
4.14
4.12
4.10
4.09
2.00
1.98
1.97
1.95
1.93
1.55 HDO
1.44
1.42
1.41
1.39
1.36
1.33
1.32
1.26
0.98
0.96
0.94
0.91
0.90
0.88
0.03
3.97
3.99
3.22
4.00
2.21
19.46
6.71
6.39
f1 (ppm)

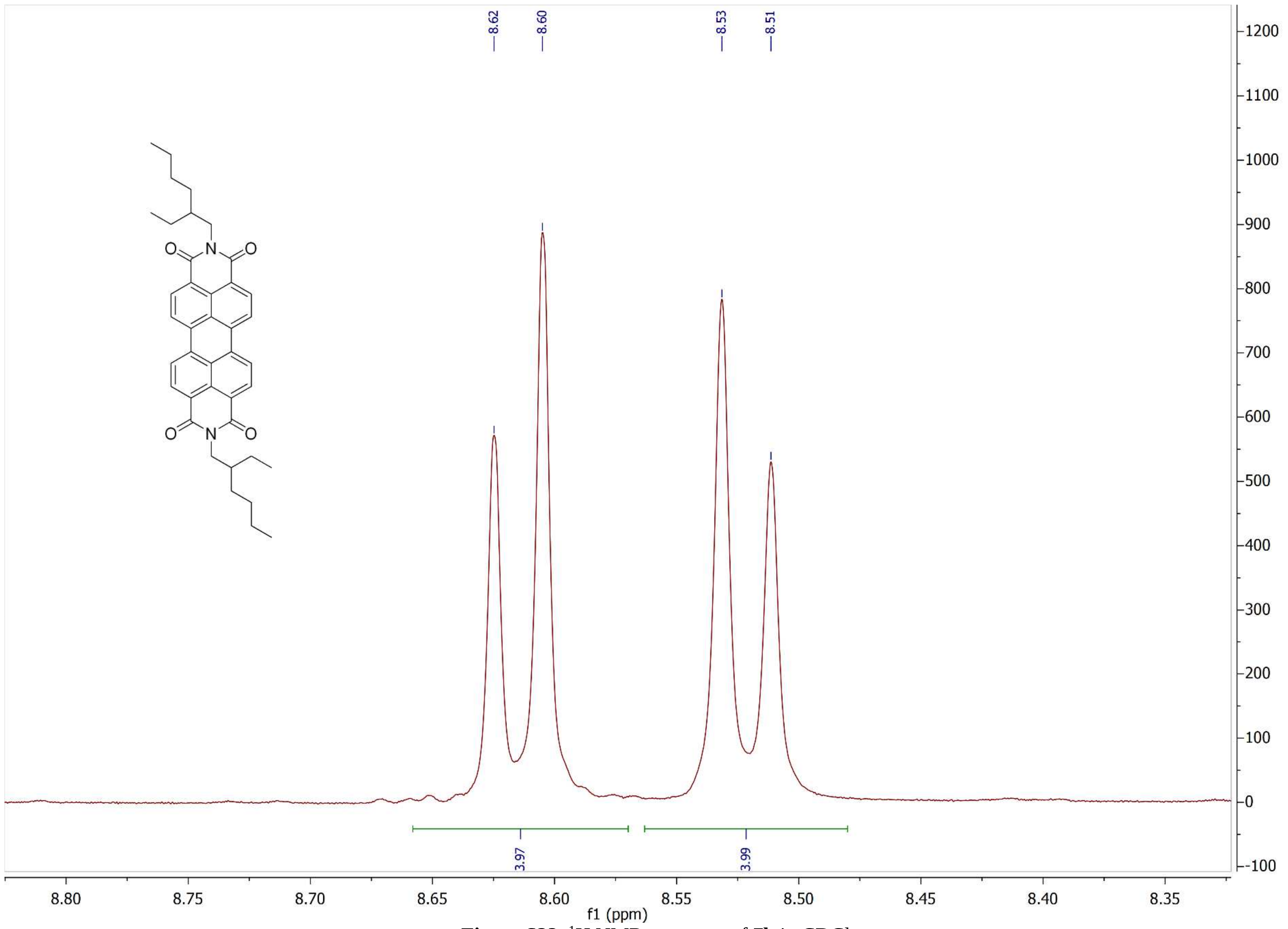

Figure S22. ^{1}H NMR spectrum of **7b** in $CDCl_3$.

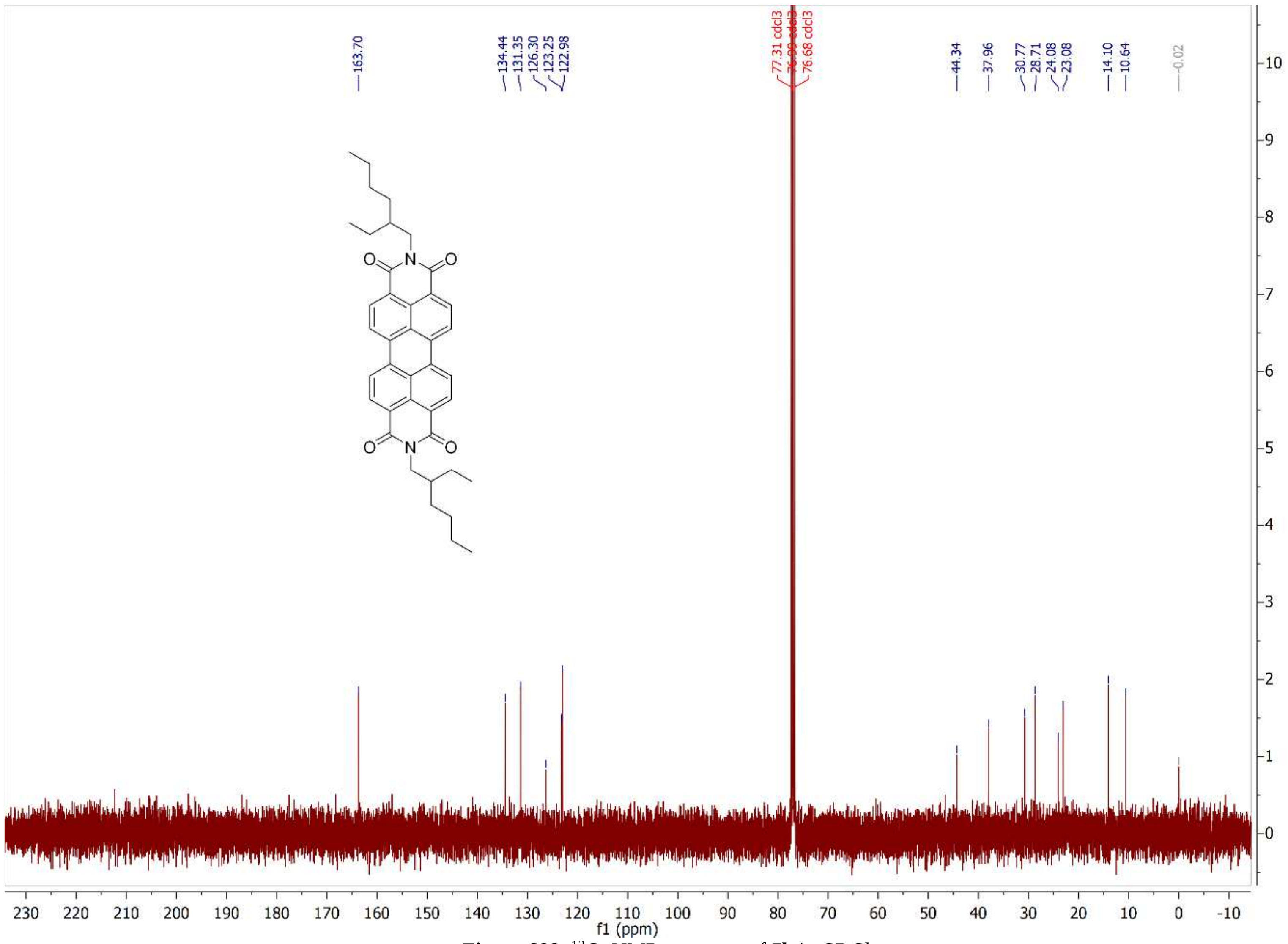

Figure S23. ^{13}C- NMR spectrum of **7b** in $CDCl_3$.

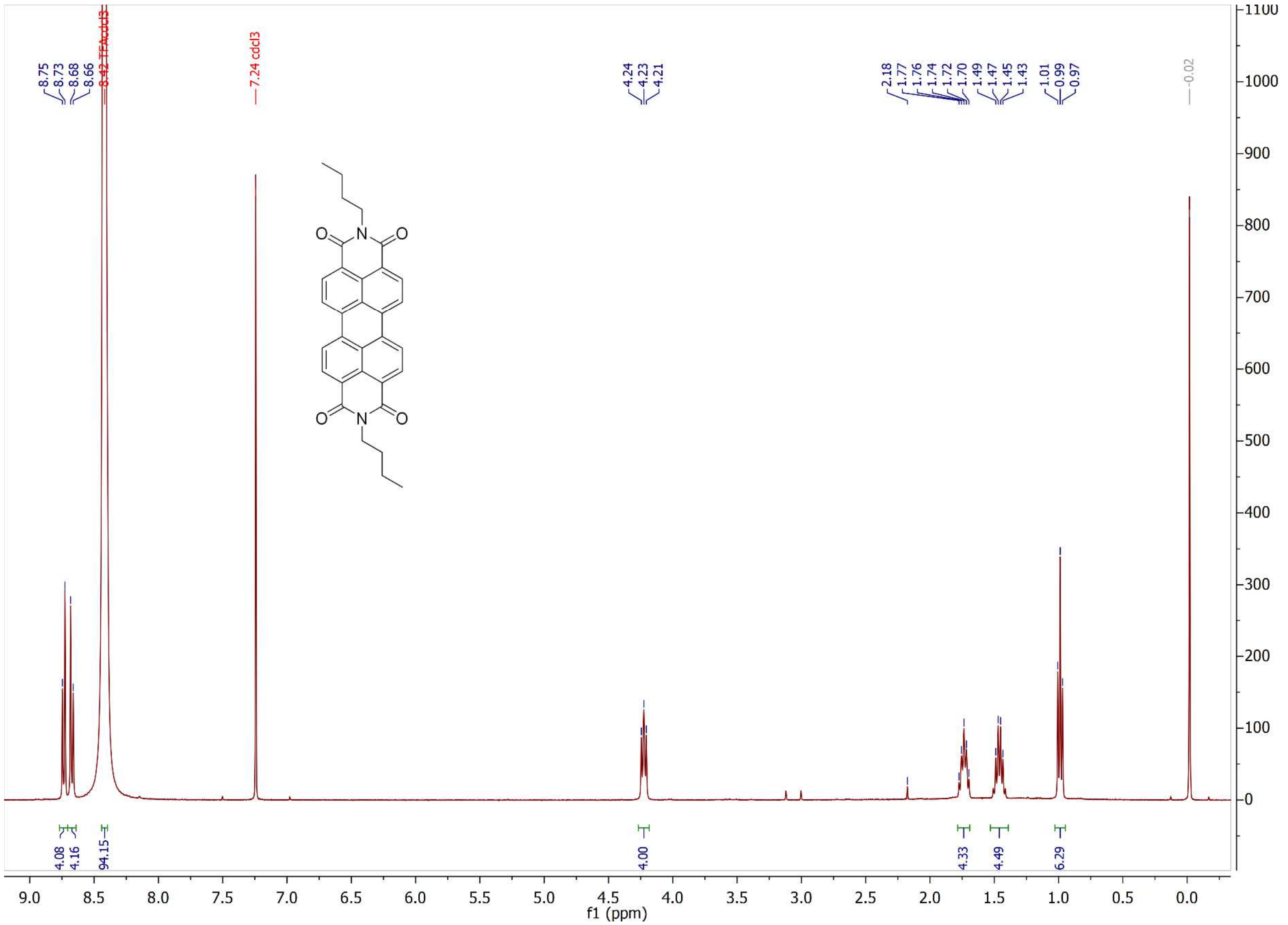

8.75
8.73
8.68
8.66
8.42 TFAcdcl3
7.24 cdcl3
4.24
4.23
4.21
2.18
1.77
1.76
1.74
1.72
1.70
1.49
1.47
1.45
1.43
1.01
0.99
0.97
-0.02
O
N
O
O
N
O
4.08
4.16
94.15
4.00
4.33
4.49
6.29
9.0 8.5 8.0 7.5 7.0 6.5 6.0 5.5 5.0 4.5 4.0 3.5 3.0 2.5 2.0 1.5 1.0 0.5 0.0
f1 (ppm)
1100
1000
900
800
700
600
500
400
300
200
100
0

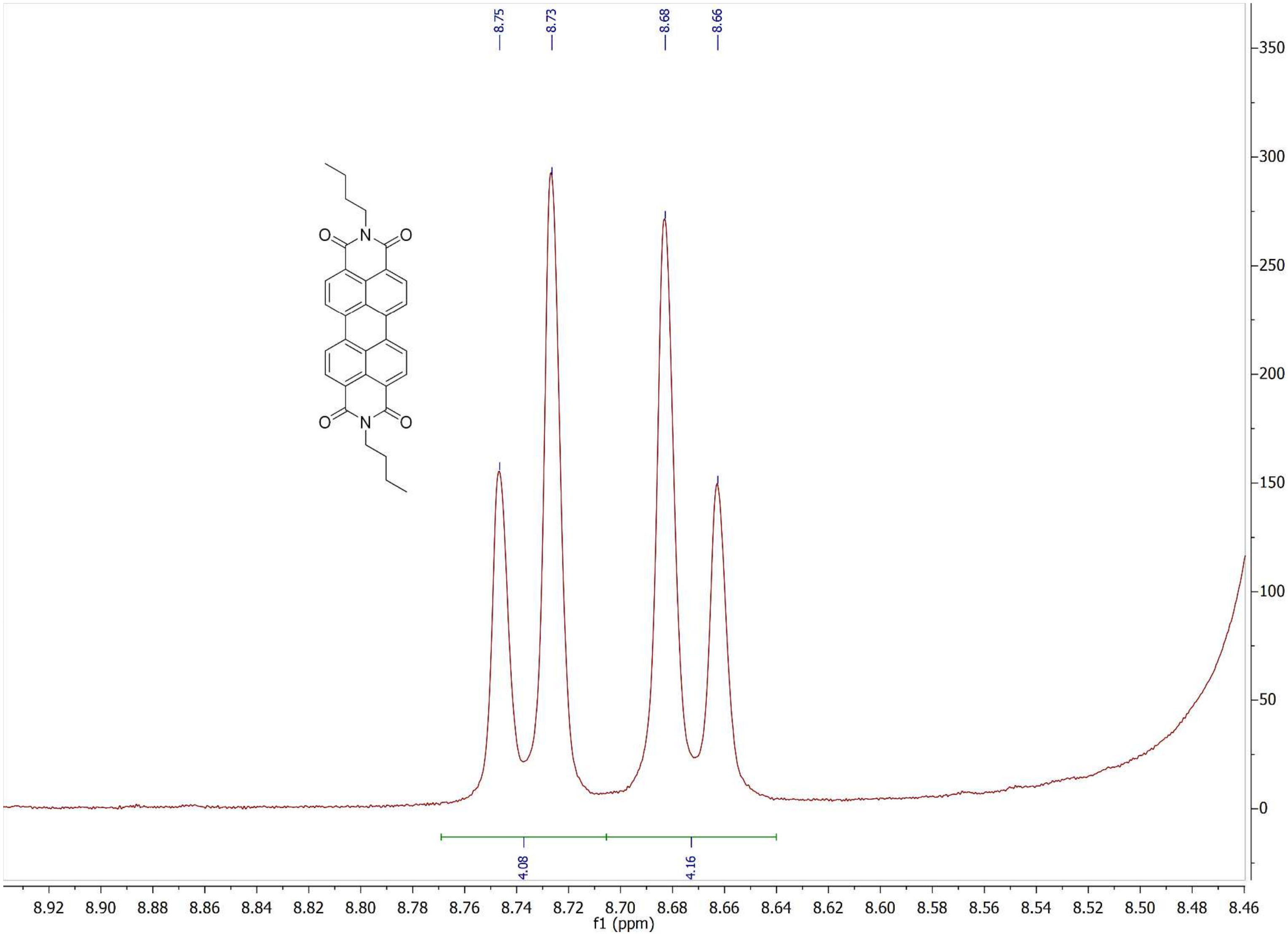

Figure S24. ^{1}H NMR spectrum of **7c** in $CDCl_3$ (with 1 drop of TFA).

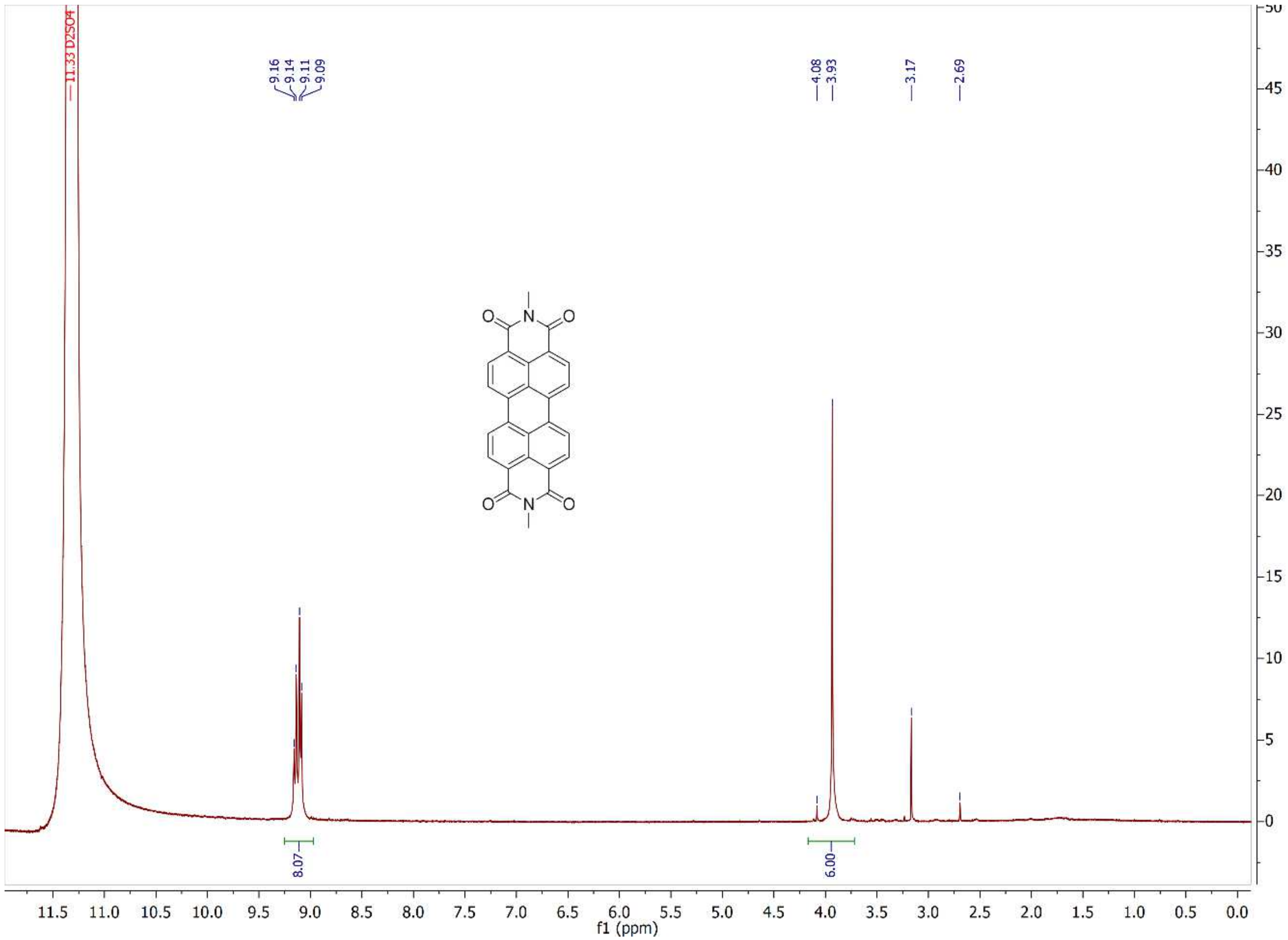
11.33 D2SO4
9.16
9.14
9.11
9.09
4.08
3.93
3.17
2.69
8.07
6.00
11.5 11.0 10.5 10.0 9.5 9.0 8.5 8.0 7.5 7.0 6.5 6.0 5.5 5.0 4.5 4.0 3.5 3.0 2.5 2.0 1.5 1.0 0.5 0.0
f1 (ppm)
50 45 40 35 30 25 20 15 10 5 0
O
N
O
O
N
O

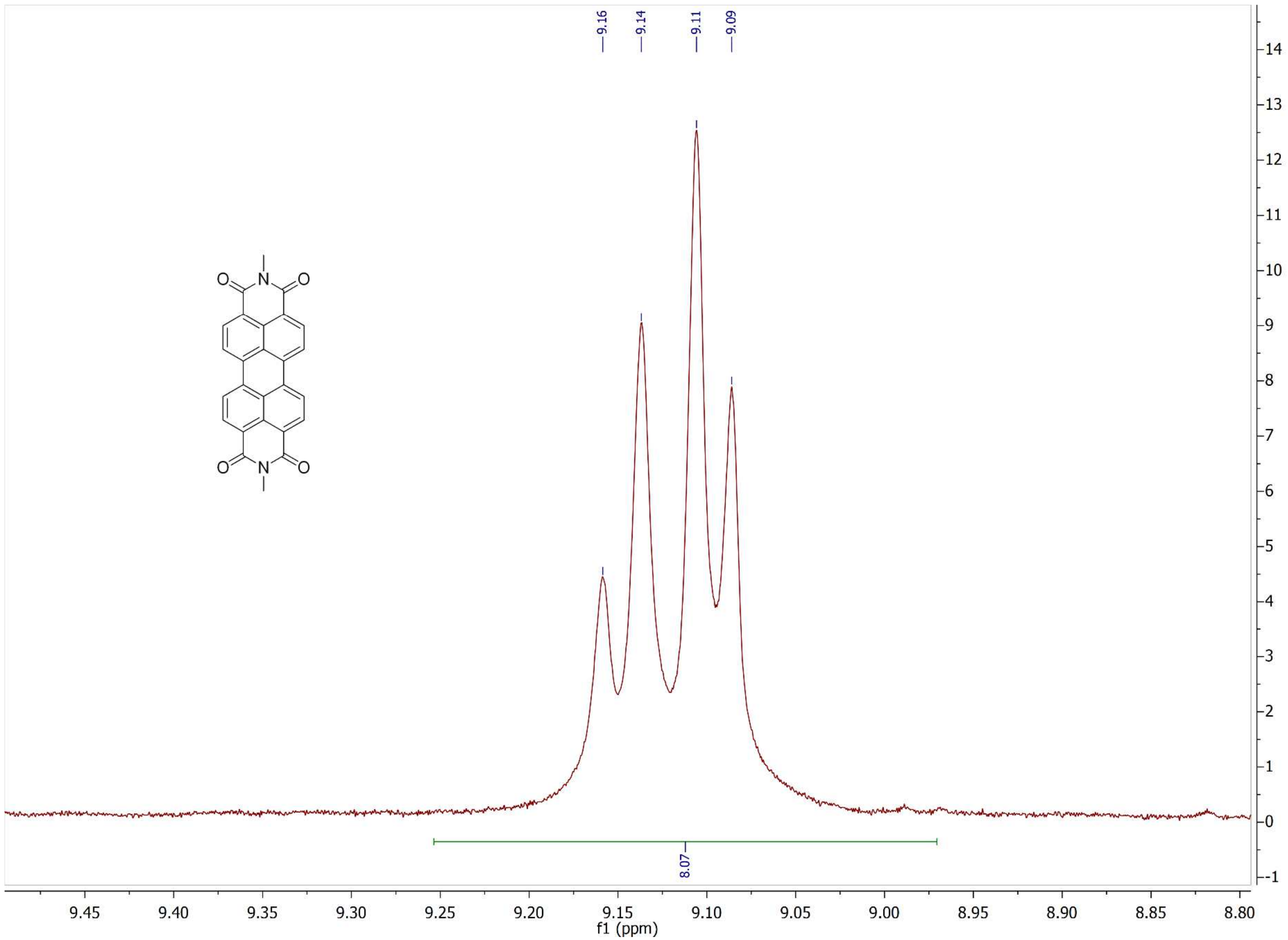

Figure S25. ^{1}H NMR spectrum of **7d** in D_2SO_4.

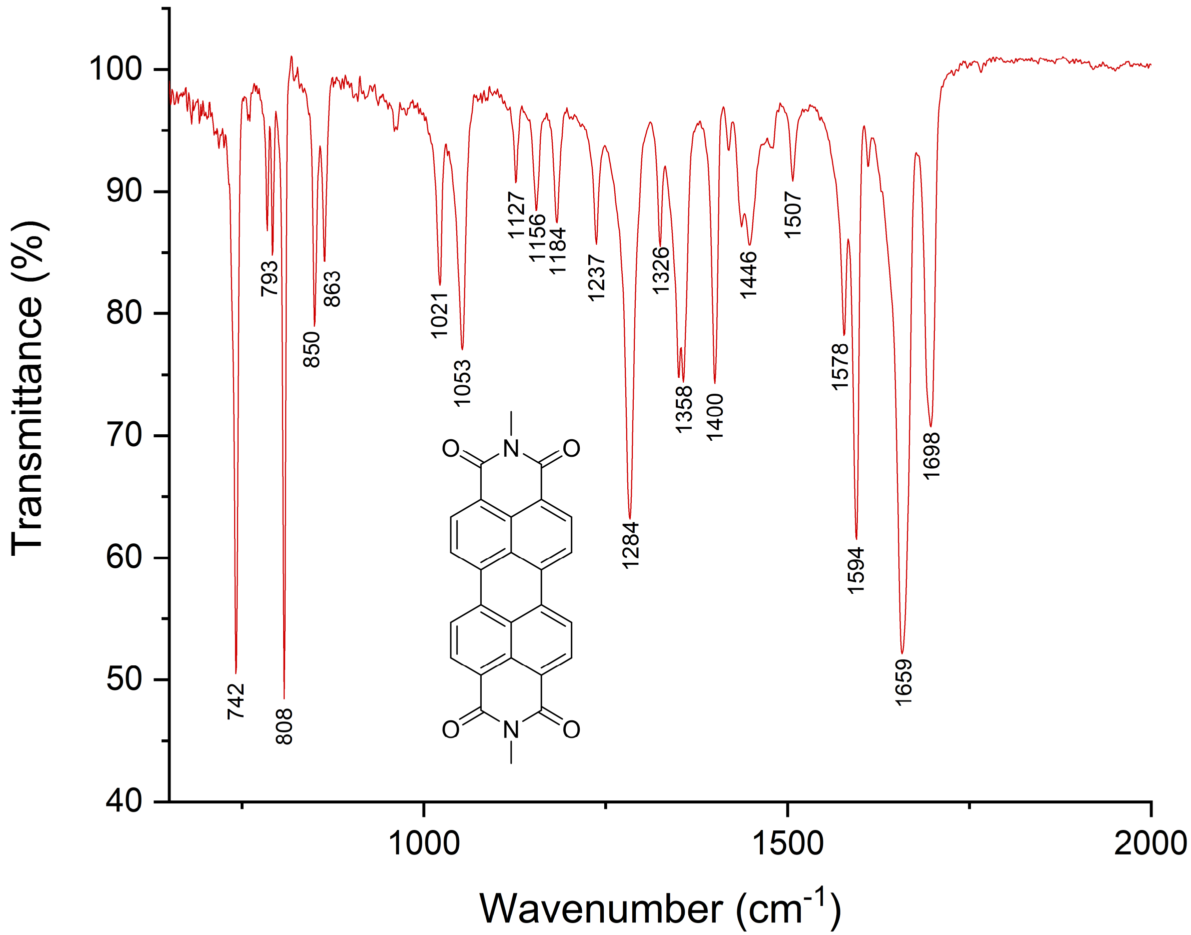

Figure S26. FTIR spectrum of **7d**.

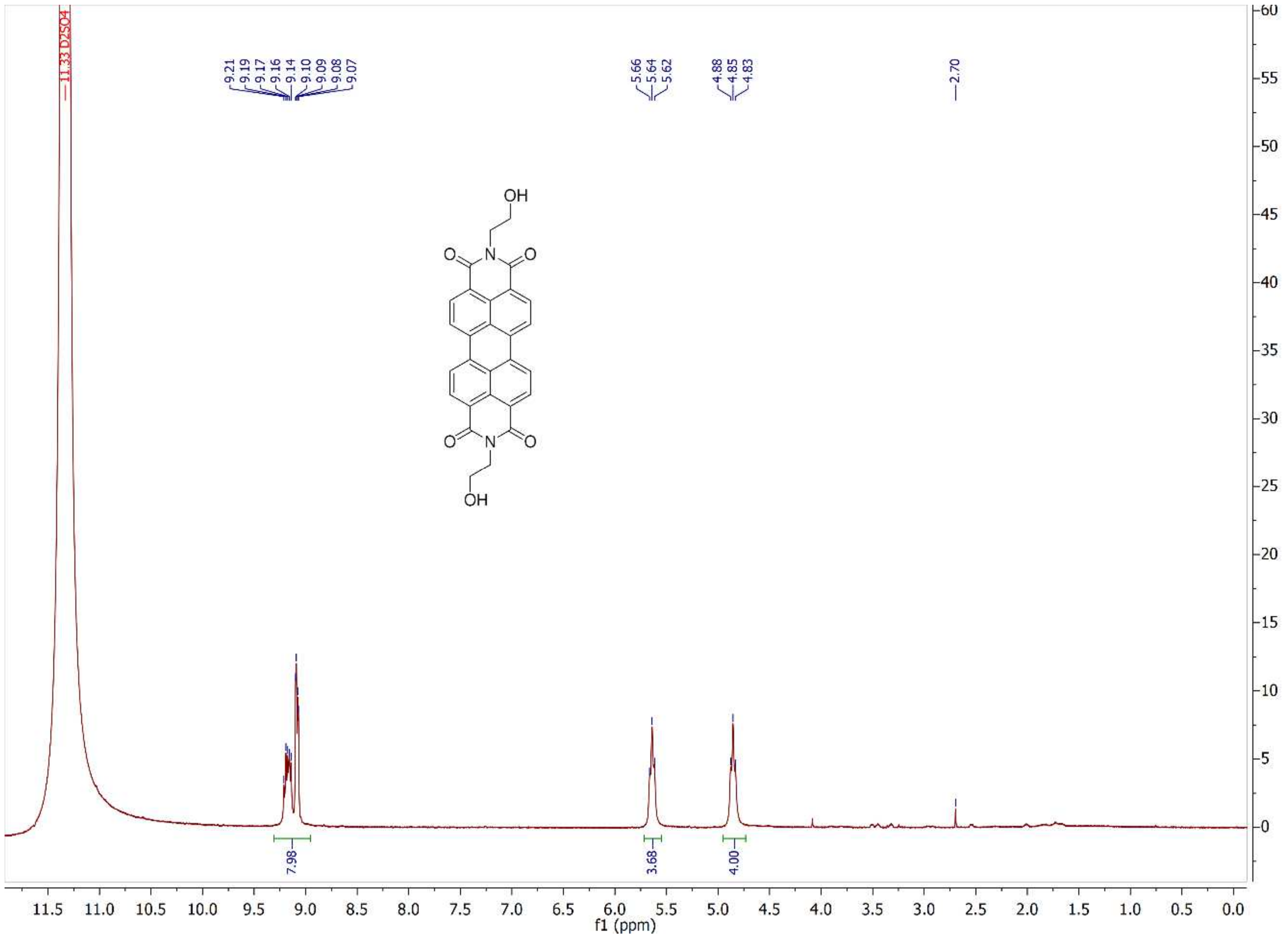
11.33 D2SO4
9.21 9.19 9.17 9.16 9.14 9.10 9.09 9.08 9.07
5.66 5.64 5.62
4.88 4.85 4.83
2.70
7.98
3.68
4.00
f1 (ppm)

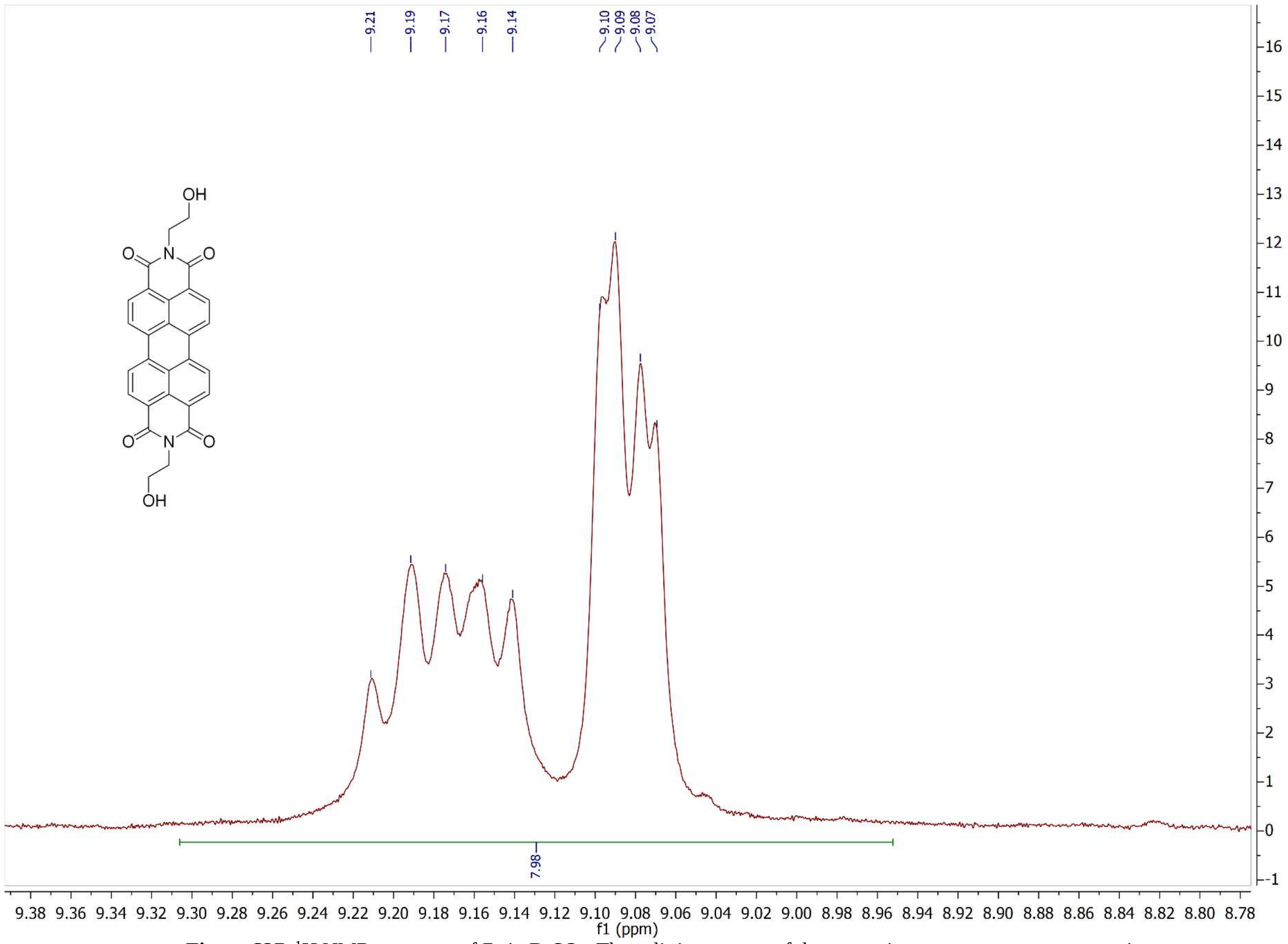

Figure S27. ^{1}H NMR spectrum of **7e** in D_2SO_4. The splitting pattern of the aromatic protons suggests aggregation.

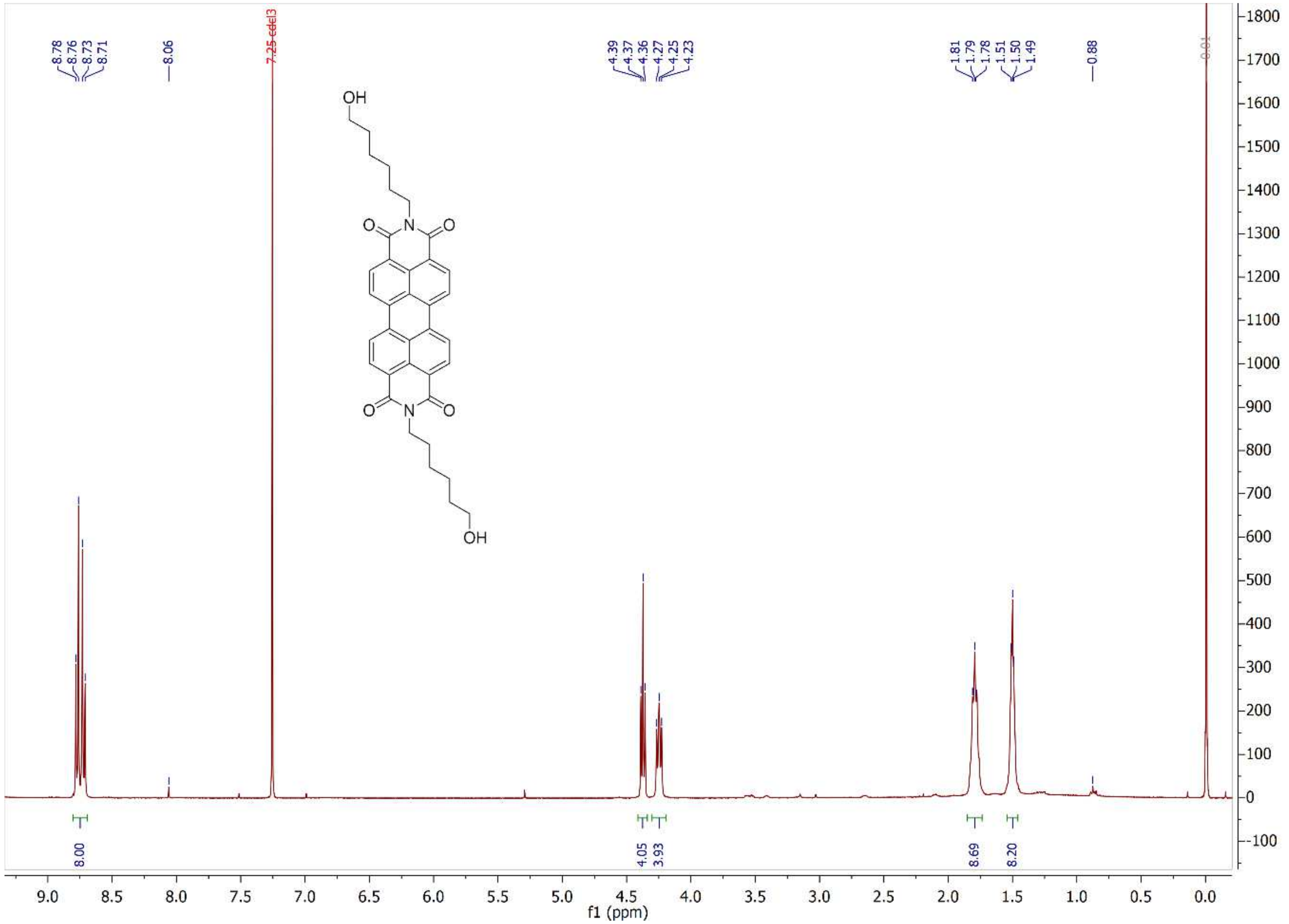
8.78
8.76
8.73
8.71
8.06
7.25 cdcl3
4.39
4.37
4.36
4.27
4.25
4.23
1.81
1.79
1.78
1.51
1.50
1.49
0.88
0.01
OH
O
N
O
O
N
O
OH
8.00
4.05
3.93
8.69
8.20
9.0
8.5
8.0
7.5
7.0
6.5
6.0
5.5
5.0
4.5
4.0
3.5
3.0
2.5
2.0
1.5
1.0
0.5
0.0
f1 (ppm)
1800
1700
1600
1500
1400
1300
1200
1100
1000
900
800
700
600
500
400
300
200
100
0
-100

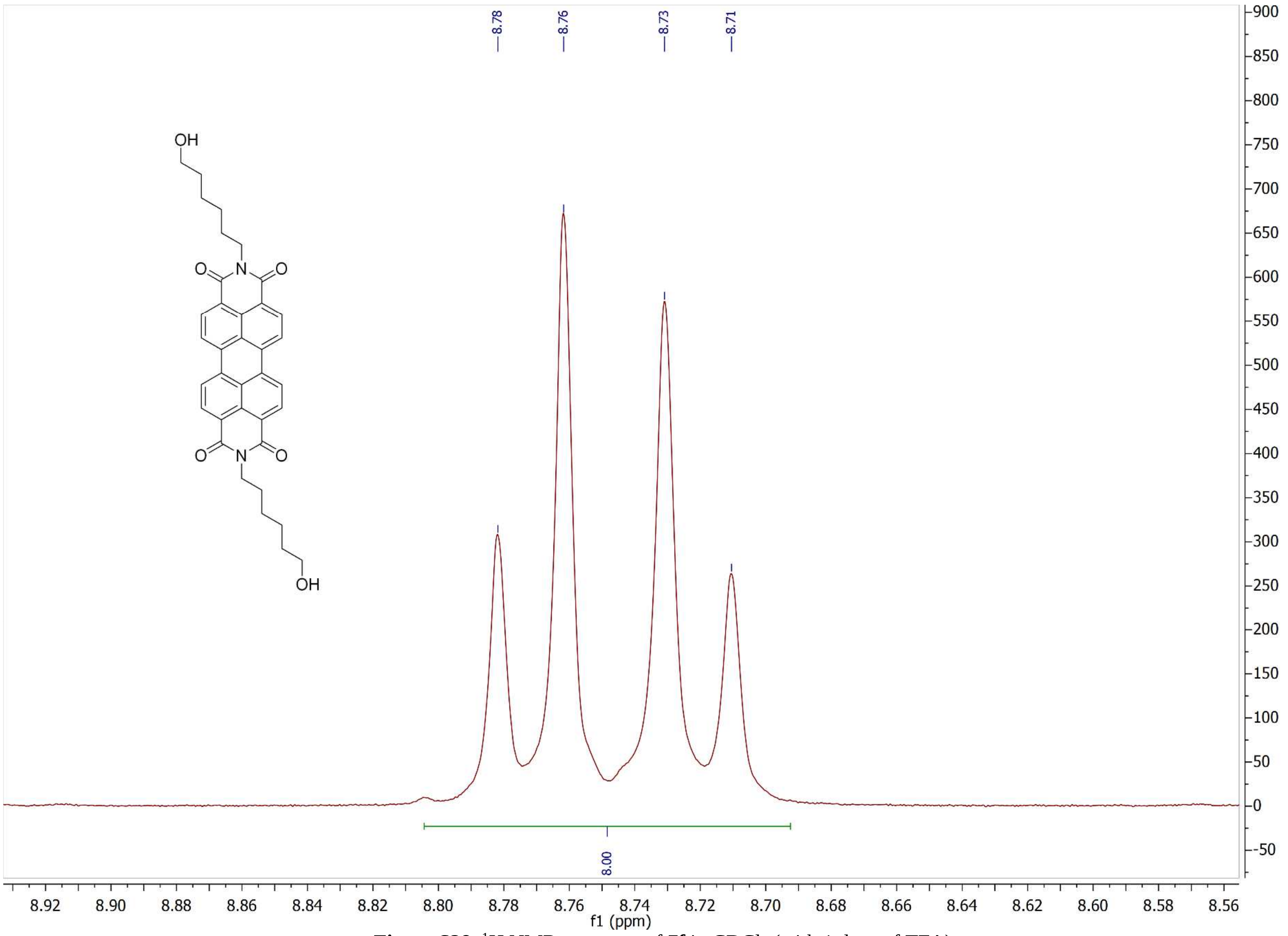

Figure S28. ^{1}H NMR spectrum of **7f** in $CDCl_3$ (with 1 drop of TFA).

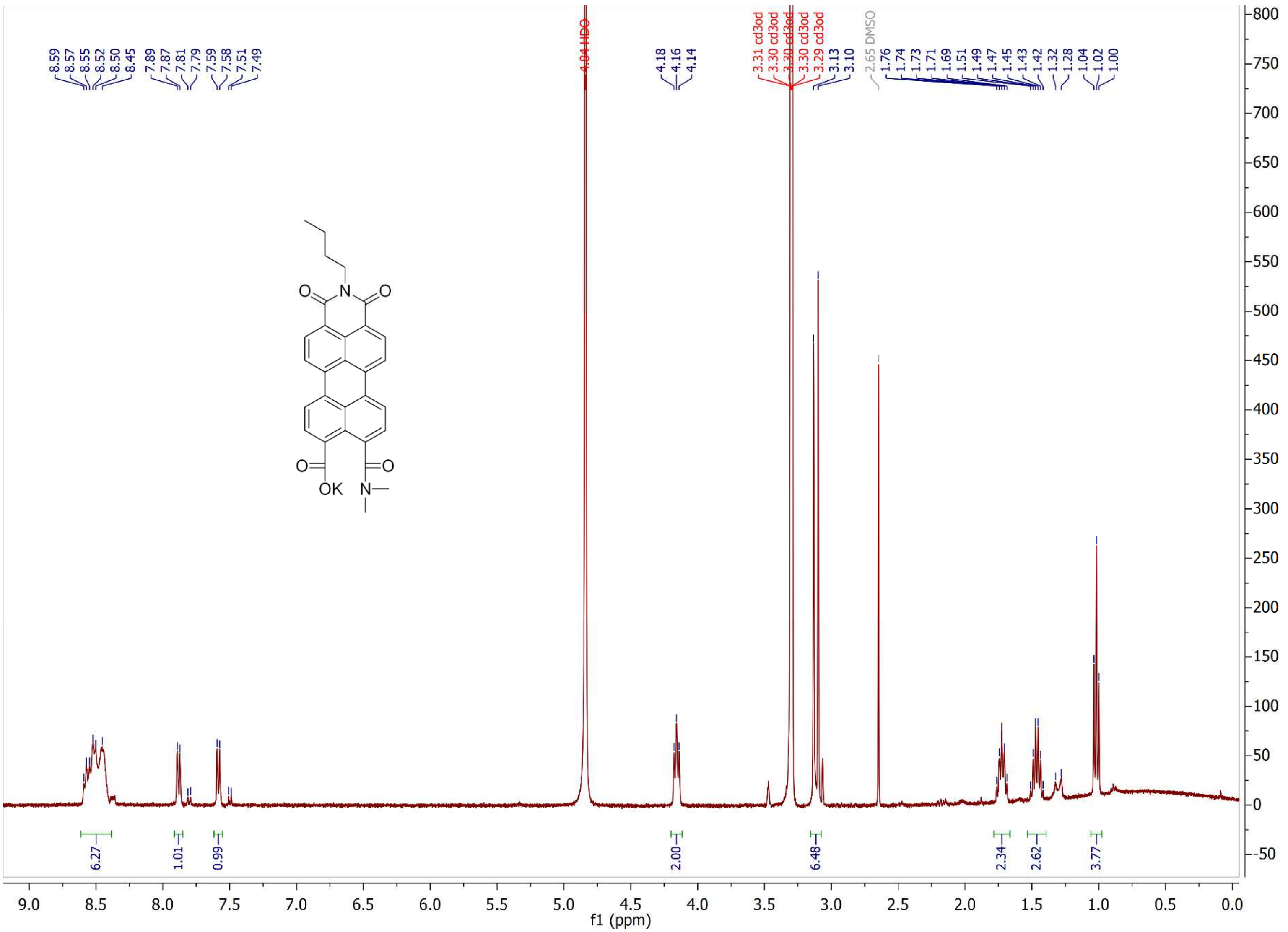

8.59
8.57
8.55
8.52
8.50
8.45
7.89
7.87
7.81
7.79
7.59
7.58
7.51
7.49
4.84 HDO
4.18
4.16
4.14
3.31 cd3od
3.30 cd3od
3.30 cd3od
3.30 cd3od
3.29 cd3od
3.13
3.10
2.65 DMSO
1.76
1.74
1.73
1.71
1.69
1.51
1.49
1.47
1.45
1.43
1.42
1.32
1.28
1.04
1.02
1.00
O
N
O
O
OK
O
N
6.27
1.01
0.99
2.00
6.48
2.34
2.62
3.77
f1 (ppm)

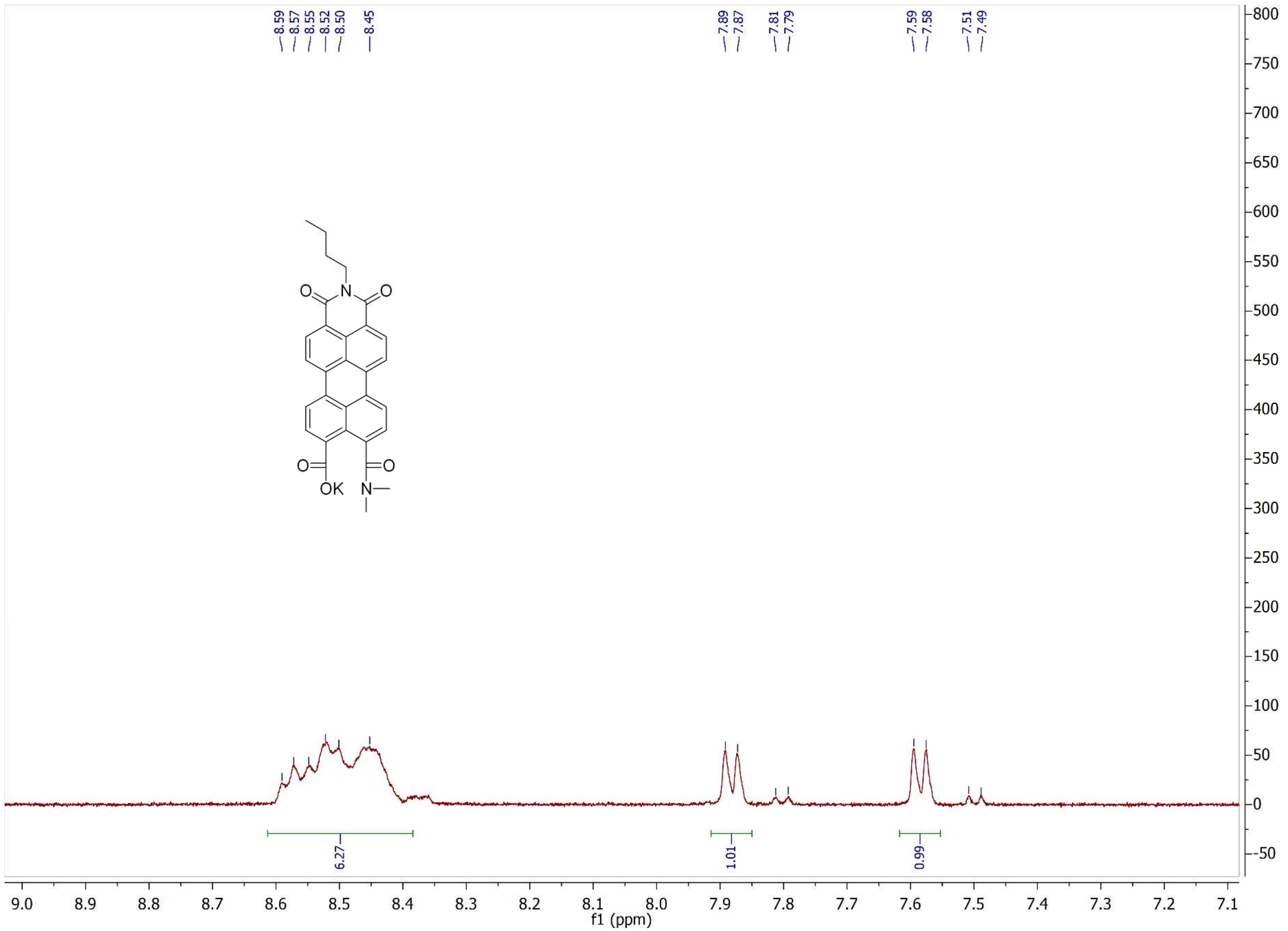

Figure S29. ^{1}H NMR spectrum of **9a** in MeOD. NMR contains methanol and water from the solvent, DMSO, and traces of compound **5a**.

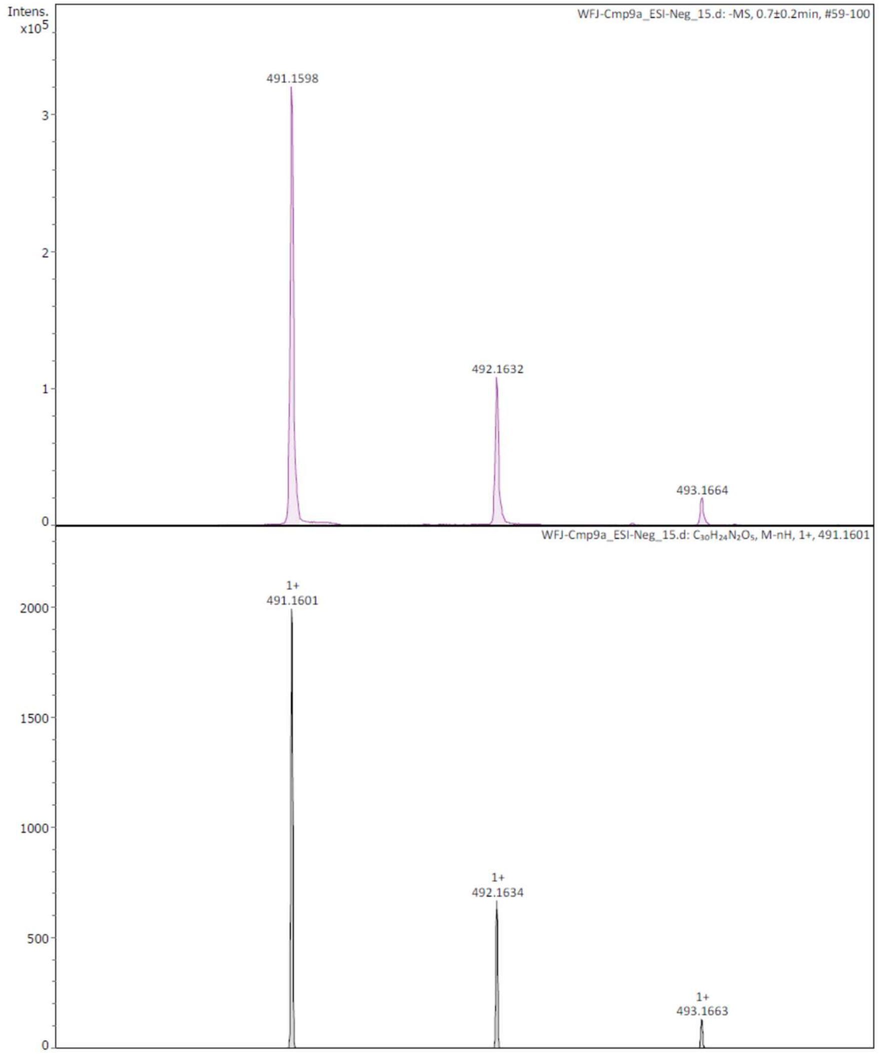
Intens.
x10^5
WFJ-Cmp9a_ESI-Neg_15.d: -MS, 0.7±0.2min, #59-100
491.1598
492.1632
493.1664
WFJ-Cmp9a_ESI-Neg_15.d: C30H24N2O5, M-nH, 1+, 491.1601
1+
491.1601
1+
492.1634
1+
493.1663
3
2
1
0
2000
1500
1000
500
0

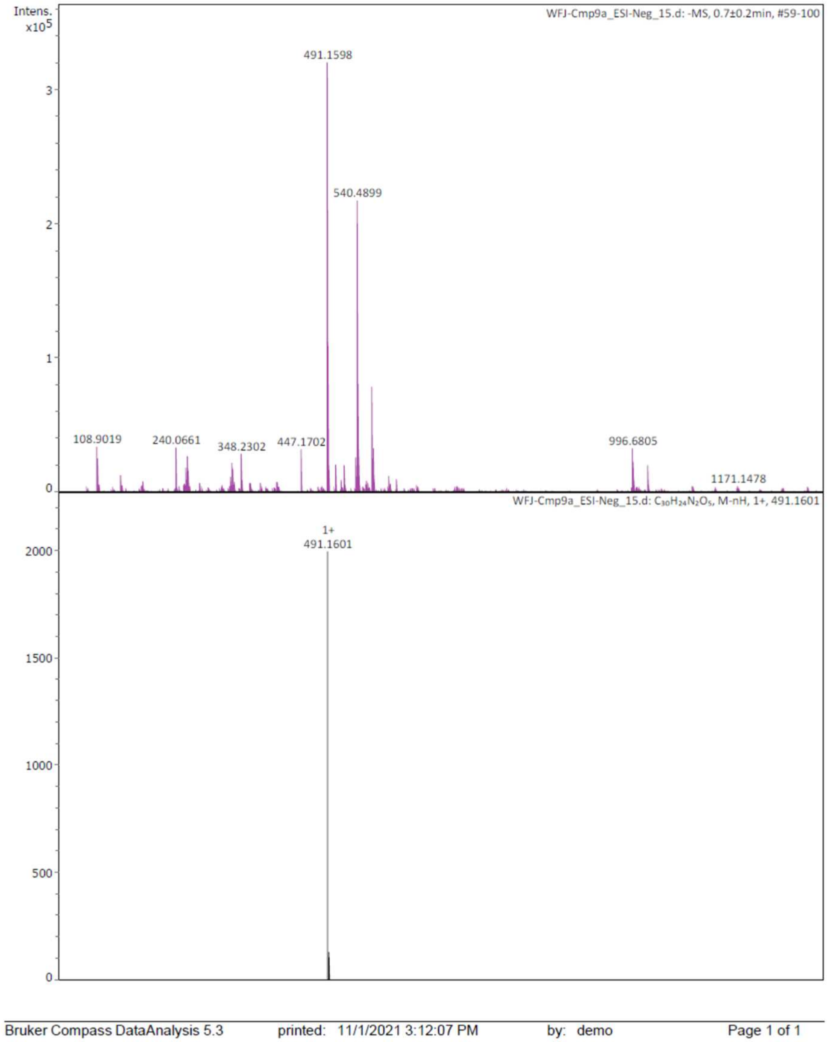

Figure S30. Mass spectrum of **9a** determined by direct injection. Calculated masses are: **9a**; 491.16, **9a$_2$H**; 983.33. Compound **15** formed after amic acid hydrolysis has a mass of 447.11.

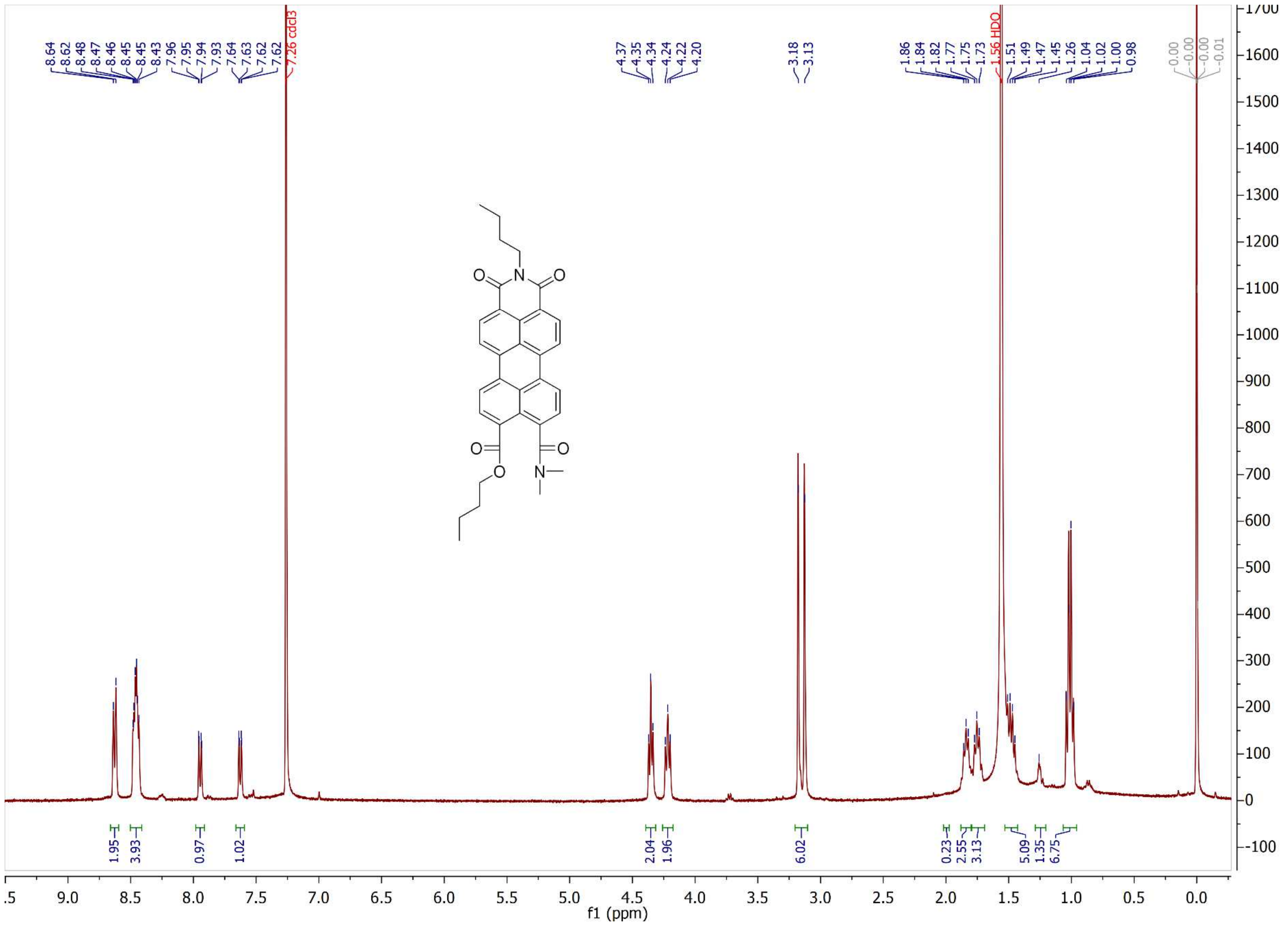
8.64
8.62
8.48
8.47
8.46
8.45
8.45
8.43
7.96
7.95
7.94
7.93
7.64
7.63
7.62
7.62
7.26 cdcl3
4.37
4.35
4.34
4.24
4.22
4.20
3.18
3.13
1.86
1.84
1.82
1.77
1.75
1.73
1.56 HDO
1.51
1.49
1.47
1.45
1.26
1.04
1.02
1.00
0.98
0.00
-0.00
-0.00
-0.01
1.95
3.93
0.97
1.02
2.04
1.96
6.02
0.23
2.55
3.13
5.09
1.35
6.75
f1 (ppm)

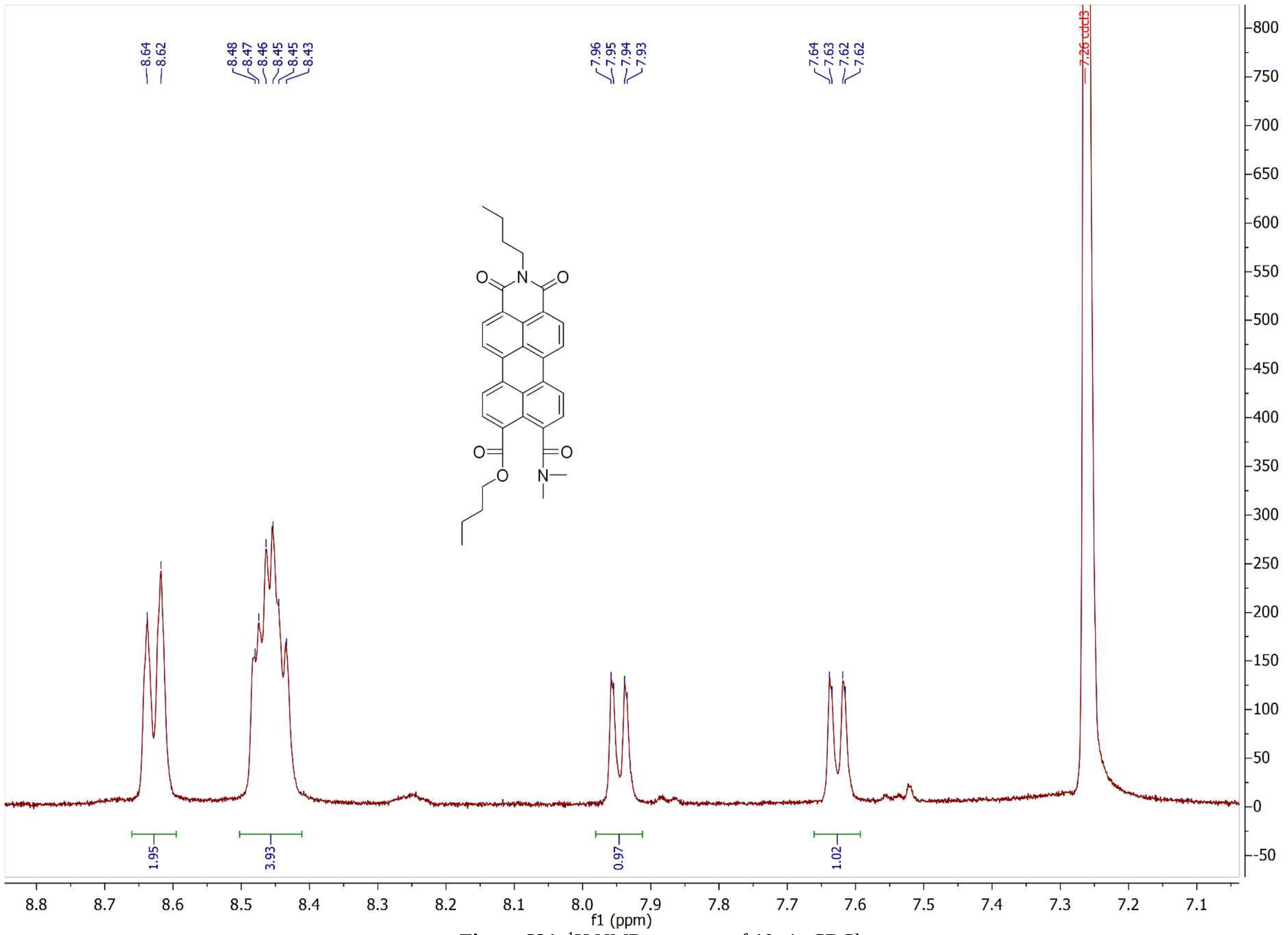

Figure S31. ^{1}H NMR spectrum of **10a** in $CDCl_3$.

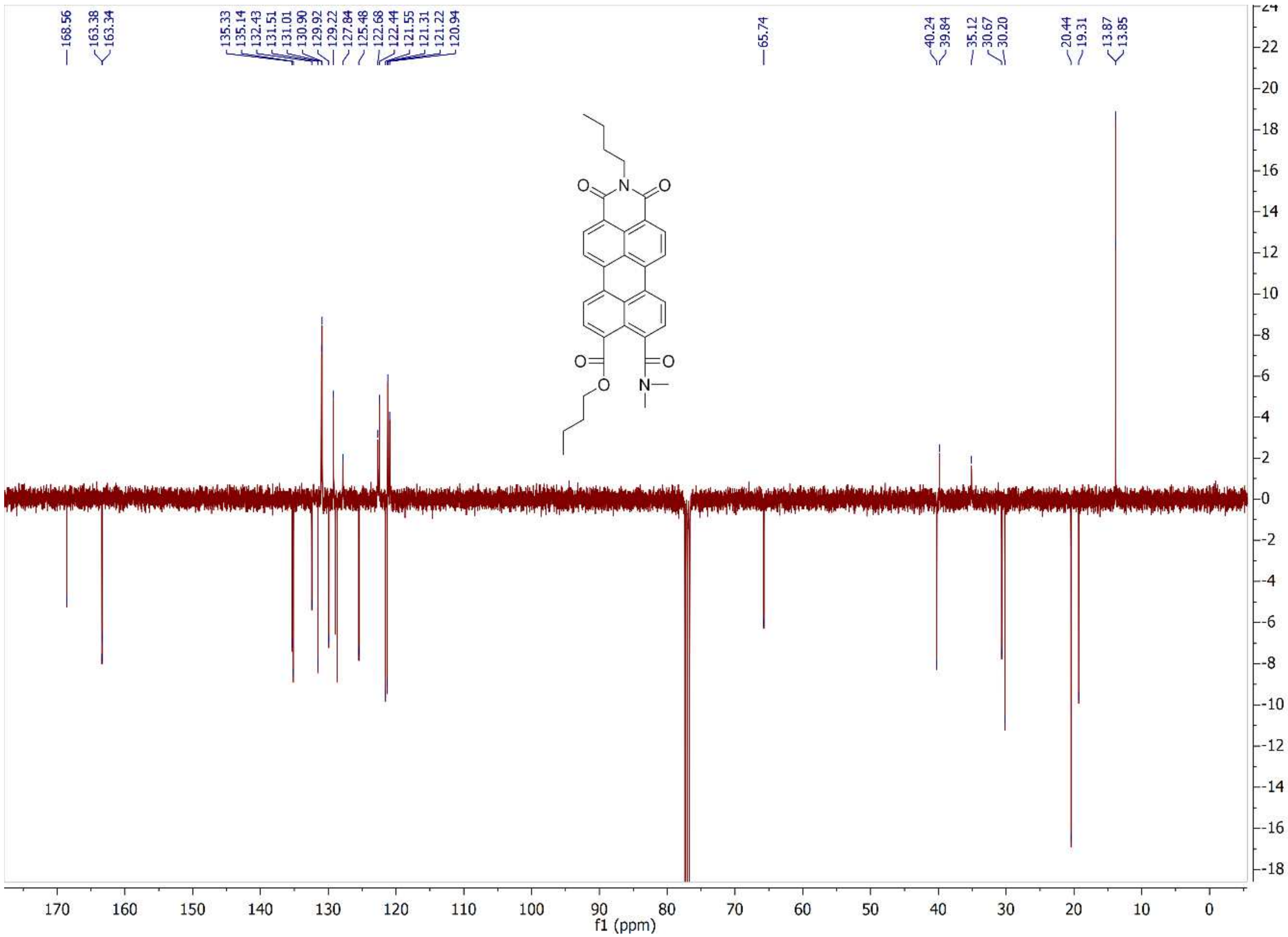
168.56
163.38
163.34
135.33
135.14
132.43
131.51
131.01
130.90
129.92
129.22
127.84
125.48
122.68
122.44
121.55
121.31
121.22
120.94
65.74
40.24
39.84
35.12
30.67
30.20
20.44
19.31
13.87
13.85
O
N
O
O
O
O
N
f1 (ppm)

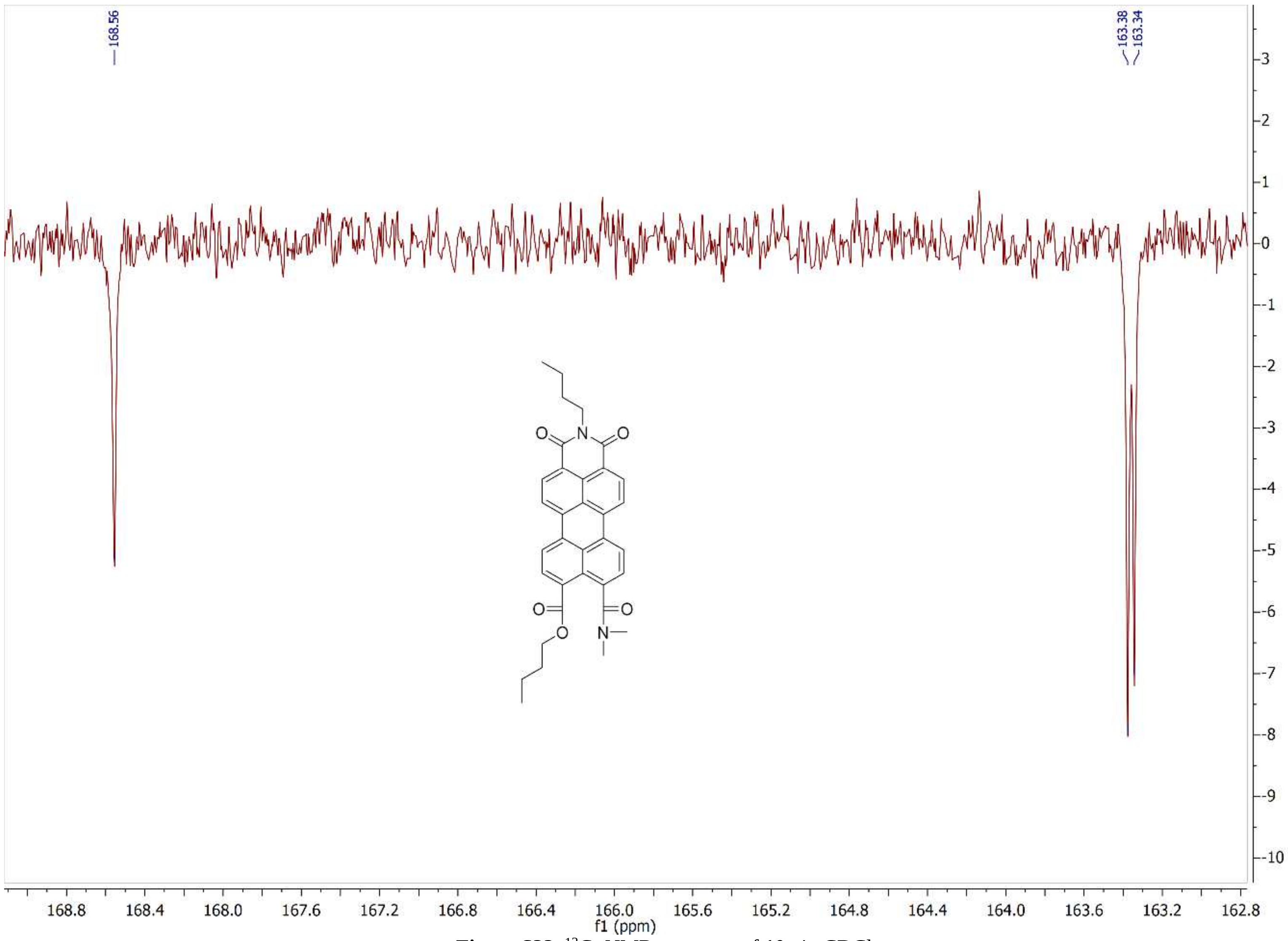

Figure S32. ^{13}C- NMR spectrum of **10a** in $CDCl_3$.

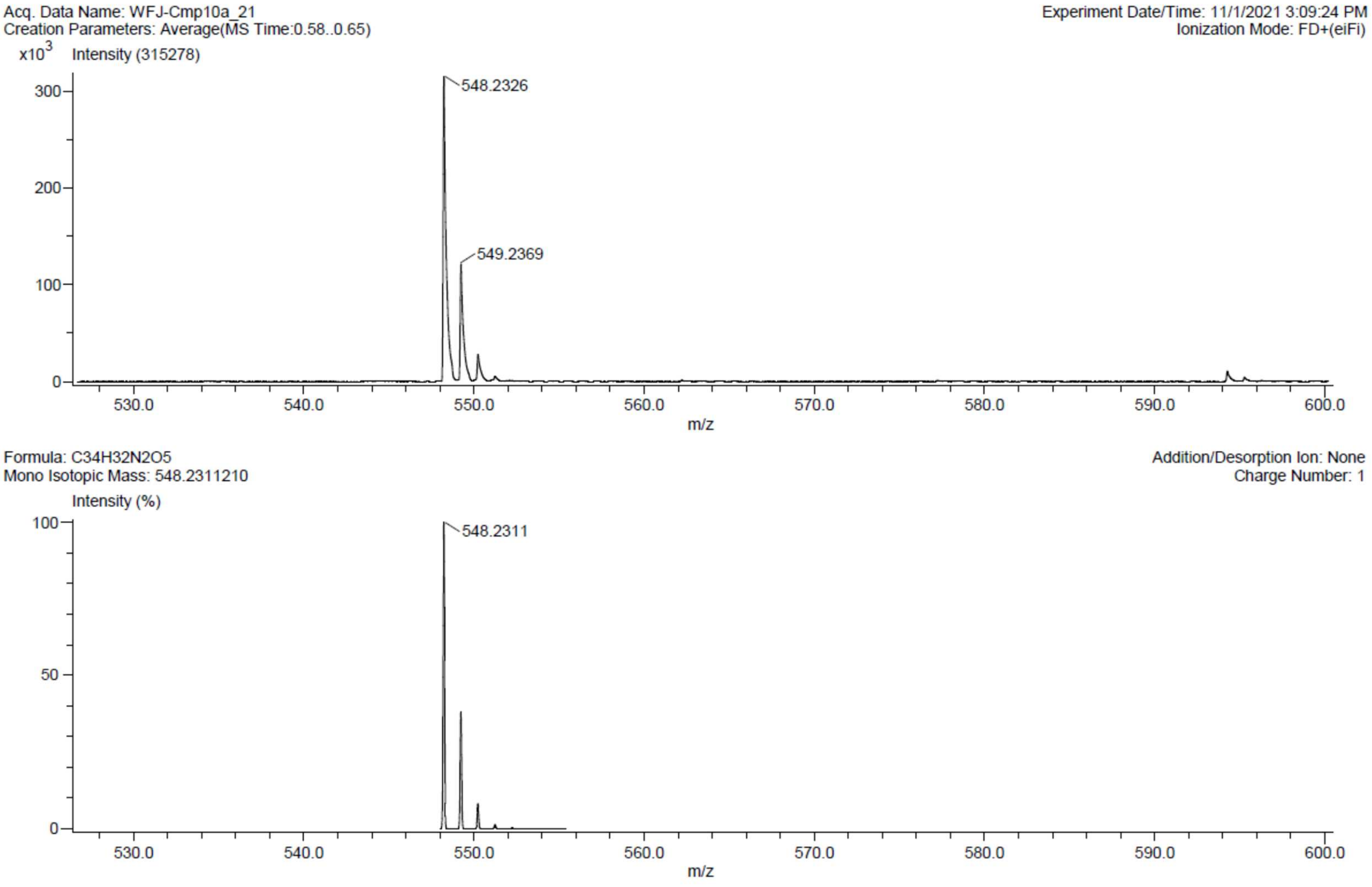
Acq. Data Name: WFJ-Cmp10a_21
Creation Parameters: Average(MS Time:0.58..0.65)
Experiment Date/Time: 11/1/2021 3:09:24 PM
Ionization Mode: FD+(eiFi)
x10^3 Intensity (315278)
548.2326
549.2369
m/z
Formula: C34H32N2O5
Mono Isotopic Mass: 548.2311210
Addition/Desorption Ion: None
Charge Number: 1
Intensity (%)
548.2311
m/z

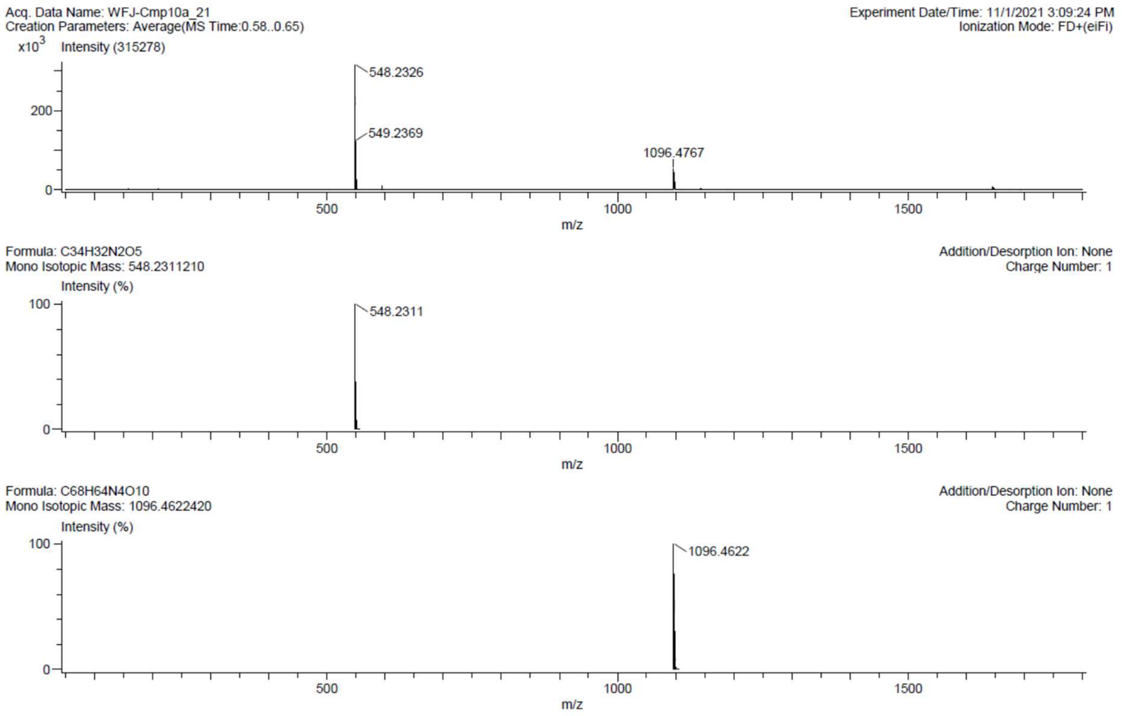

Figure S33. Mass spectrum of **10a**. Calculated masses are: **10a**; 548.2311, **10a$_2$**; 1096.4622.

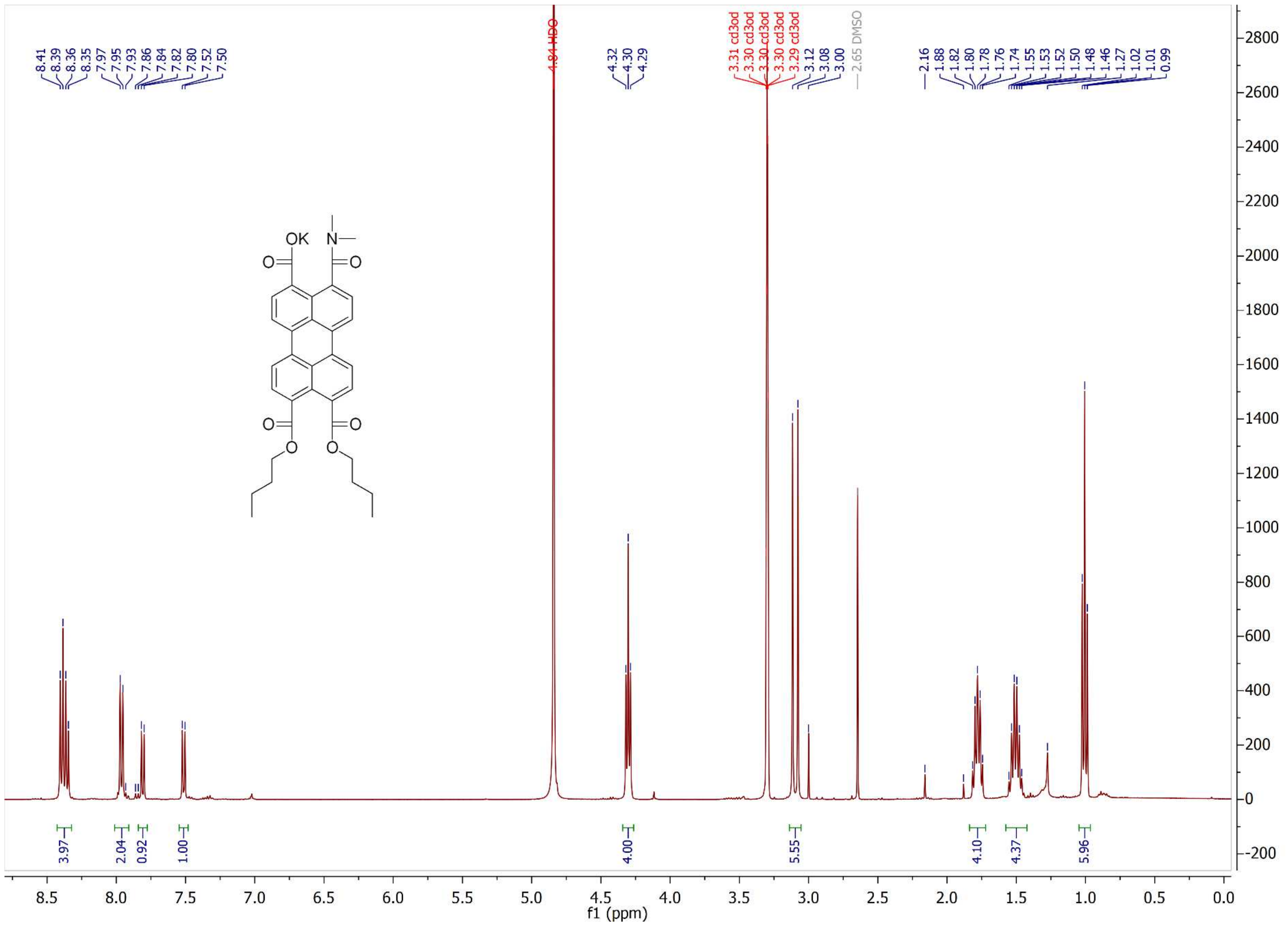
8.41
8.39
8.36
8.35
7.97
7.95
7.93
7.86
7.84
7.82
7.80
7.52
7.50
4.84 HDO
4.32
4.30
4.29
3.31 cd3od
3.30 cd3od
3.30 cd3od
3.30 cd3od
3.29 cd3od
3.12
3.08
3.00
2.65 DMSO
2.16
1.88
1.82
1.80
1.78
1.76
1.74
1.55
1.53
1.52
1.50
1.48
1.46
1.27
1.02
1.01
0.99
OK
N
O
O
O
O
O
O
3.97
2.04
0.92
1.00
4.00
5.55
4.10
4.37
5.96
f1 (ppm)

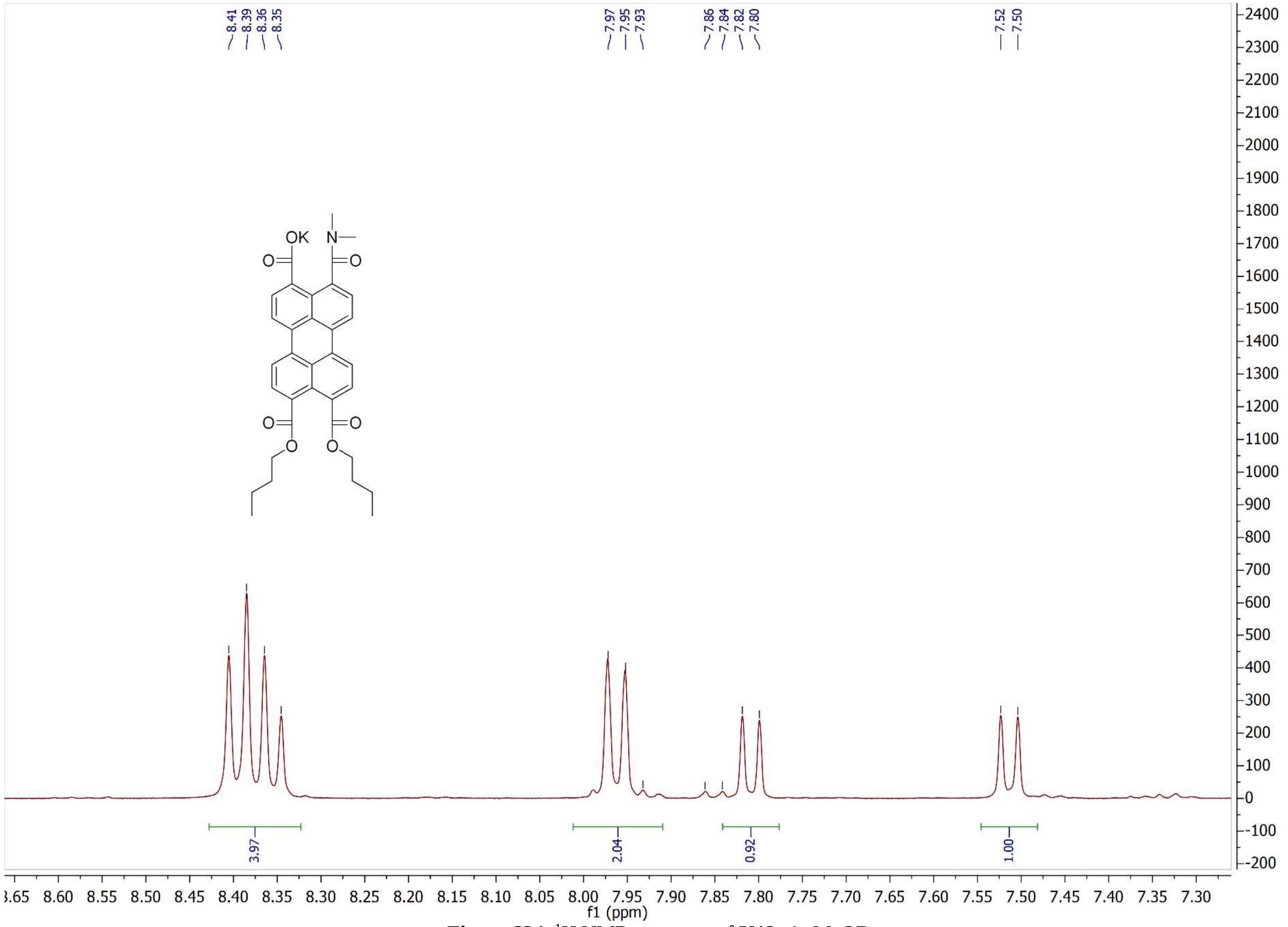

Figure S34. ^{1}H NMR spectrum of **K12a** in MeOD.

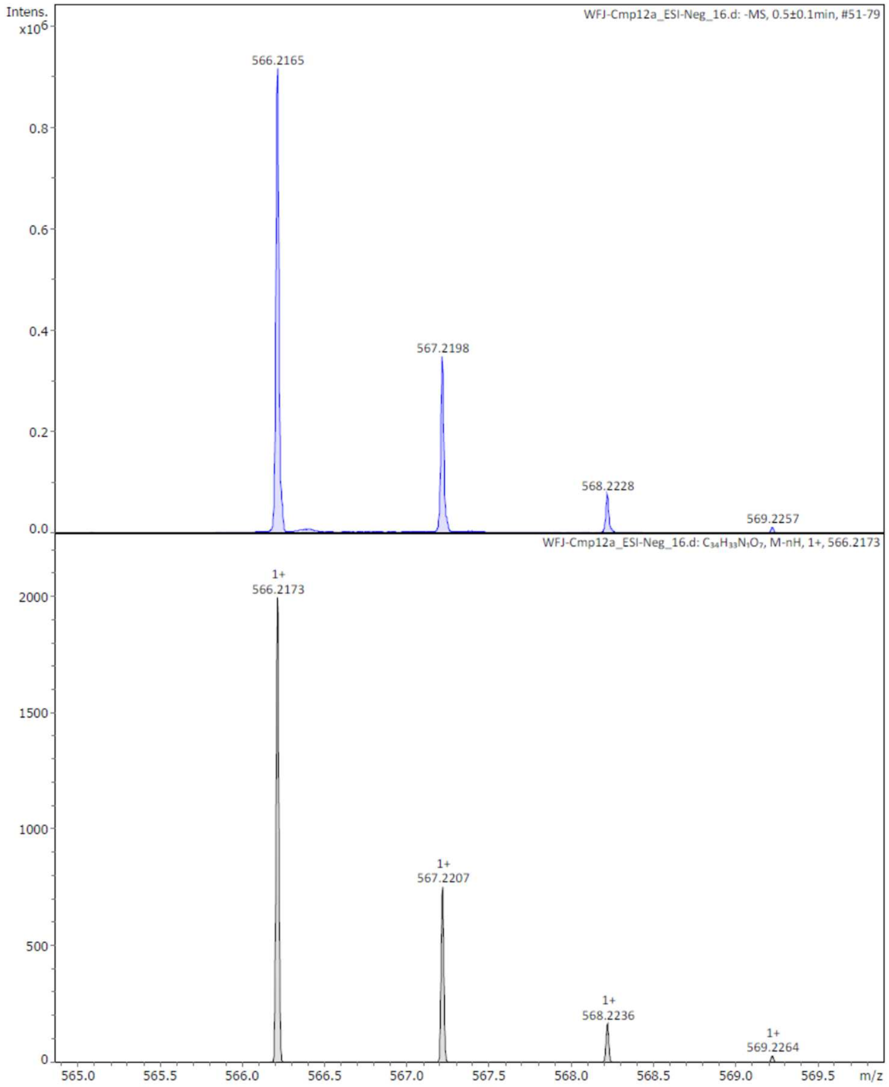

Bruker Compass DataAnalysis 5.3 printed: 11/1/2021 2:26:57 PM by: demo Page 1 of 1

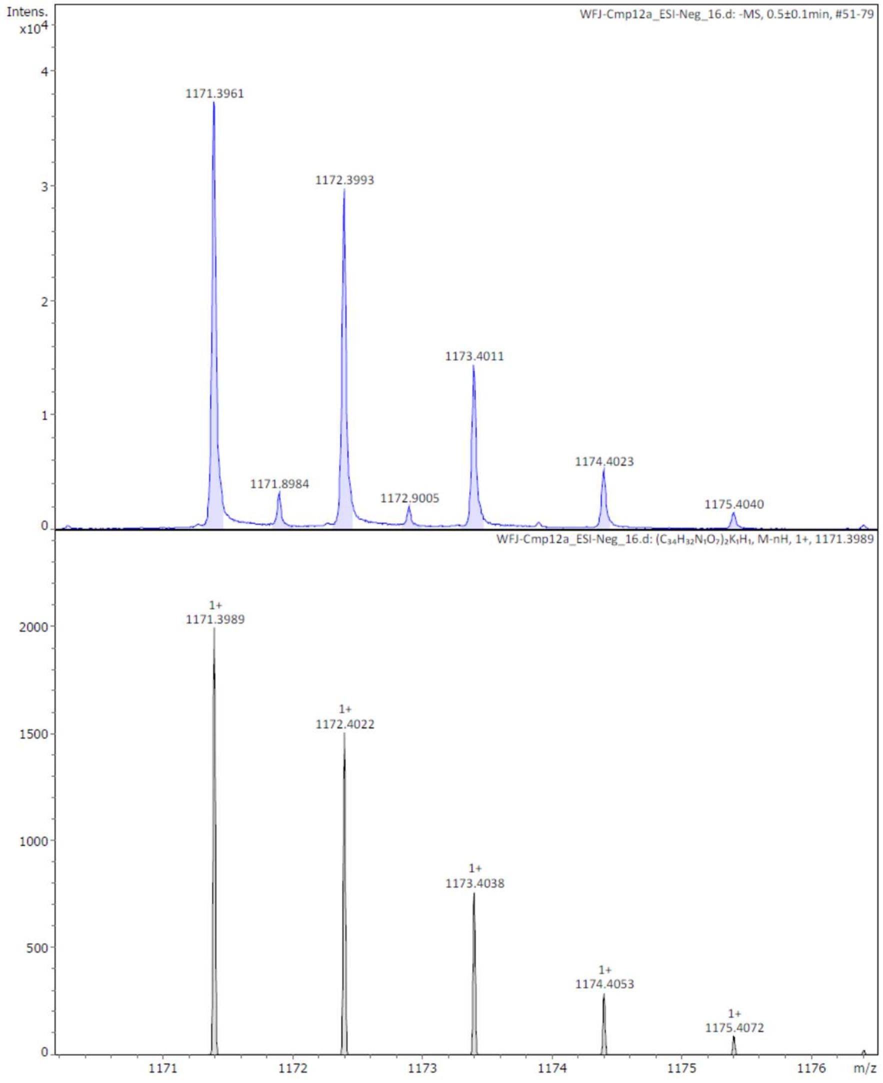

Bruker Compass DataAnalysis 5.3 printed: 11/1/2021 2:29:15 PM by: demo Page 1 of 1

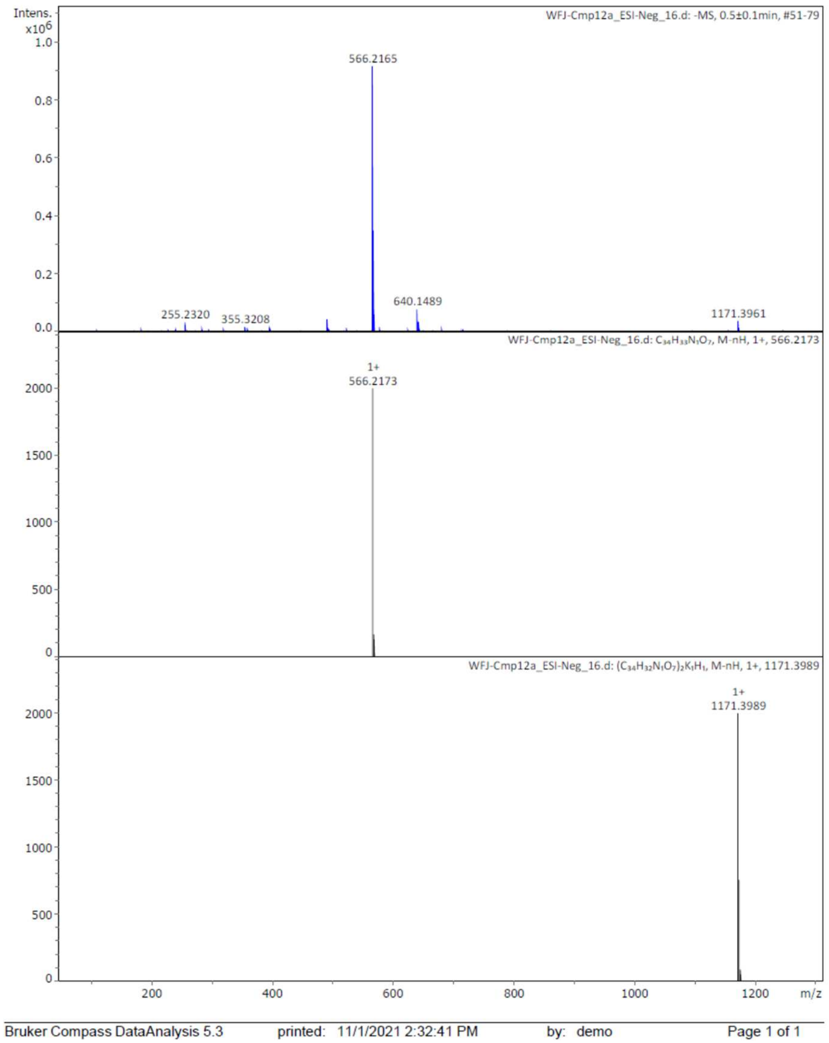

Figure S35. Mass spectrum of **12a**. Calculated masses are: **12a**; 566.2173 and .

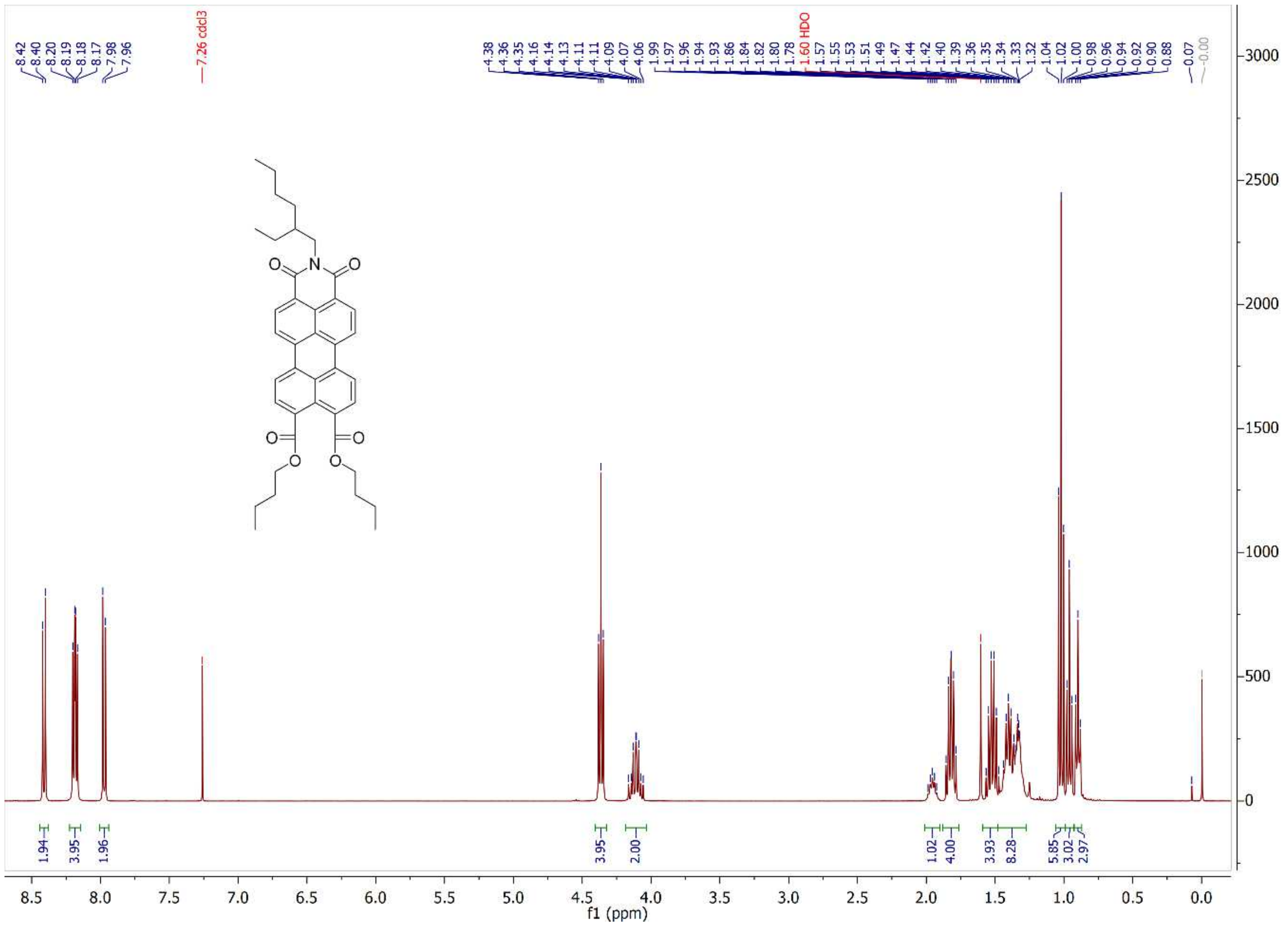
8.42
8.40
8.20
8.19
8.18
8.17
7.98
7.96
7.26 cdcl3
4.38
4.36
4.35
4.16
4.14
4.13
4.11
4.11
4.09
4.07
4.06
1.99
1.97
1.96
1.94
1.93
1.86
1.84
1.82
1.80
1.78
1.60 HDO
1.57
1.55
1.53
1.51
1.49
1.47
1.44
1.42
1.40
1.39
1.36
1.35
1.34
1.33
1.32
1.04
1.02
1.00
0.98
0.96
0.94
0.92
0.90
0.88
0.07
-0.00
1.94
3.95
1.96
3.95
2.00
1.02
4.00
3.93
8.28
5.85
3.02
2.97
f1 (ppm)

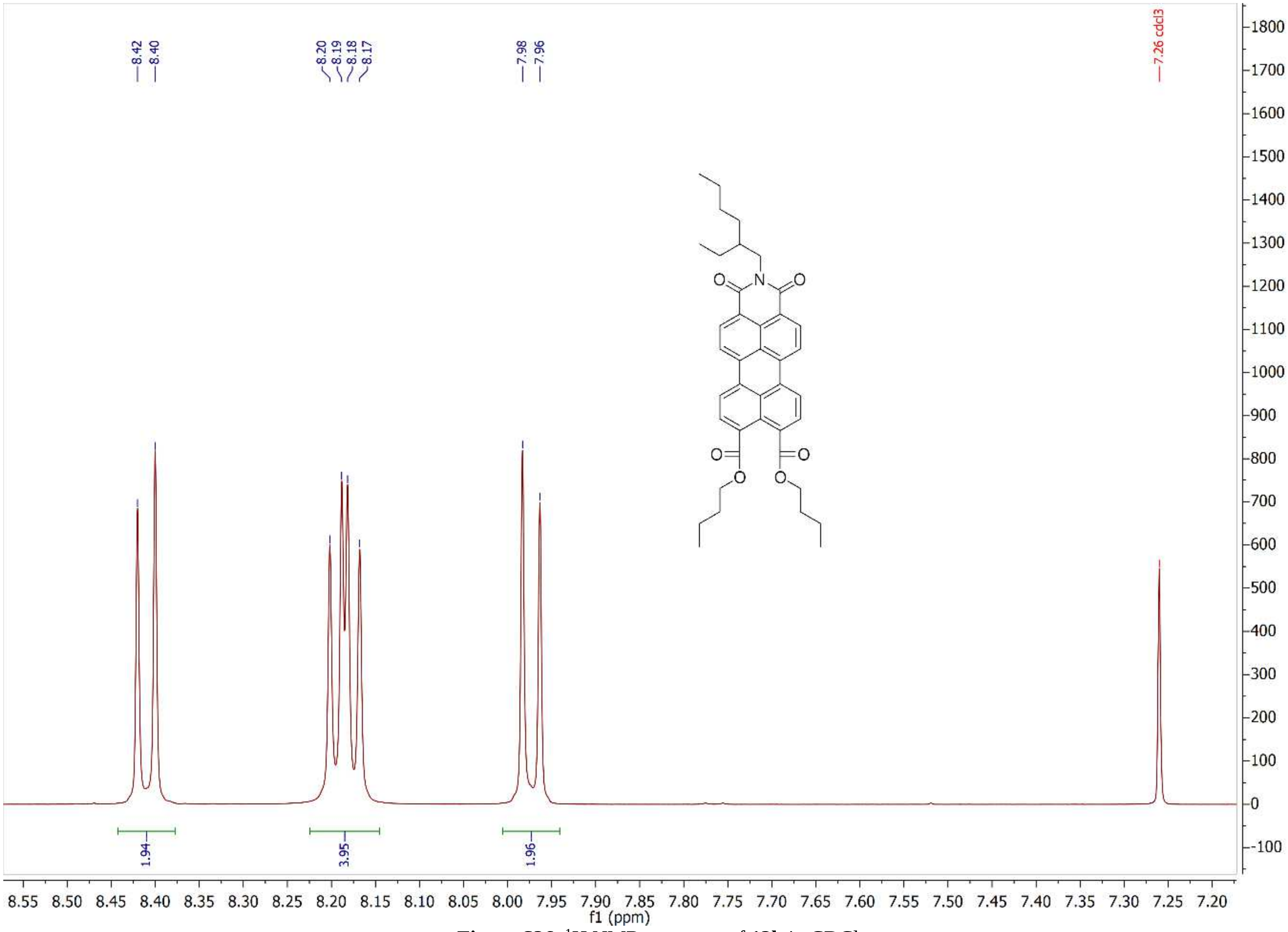

Figure S36. ^{1}H NMR spectrum of **13b** in $CDCl_3$.

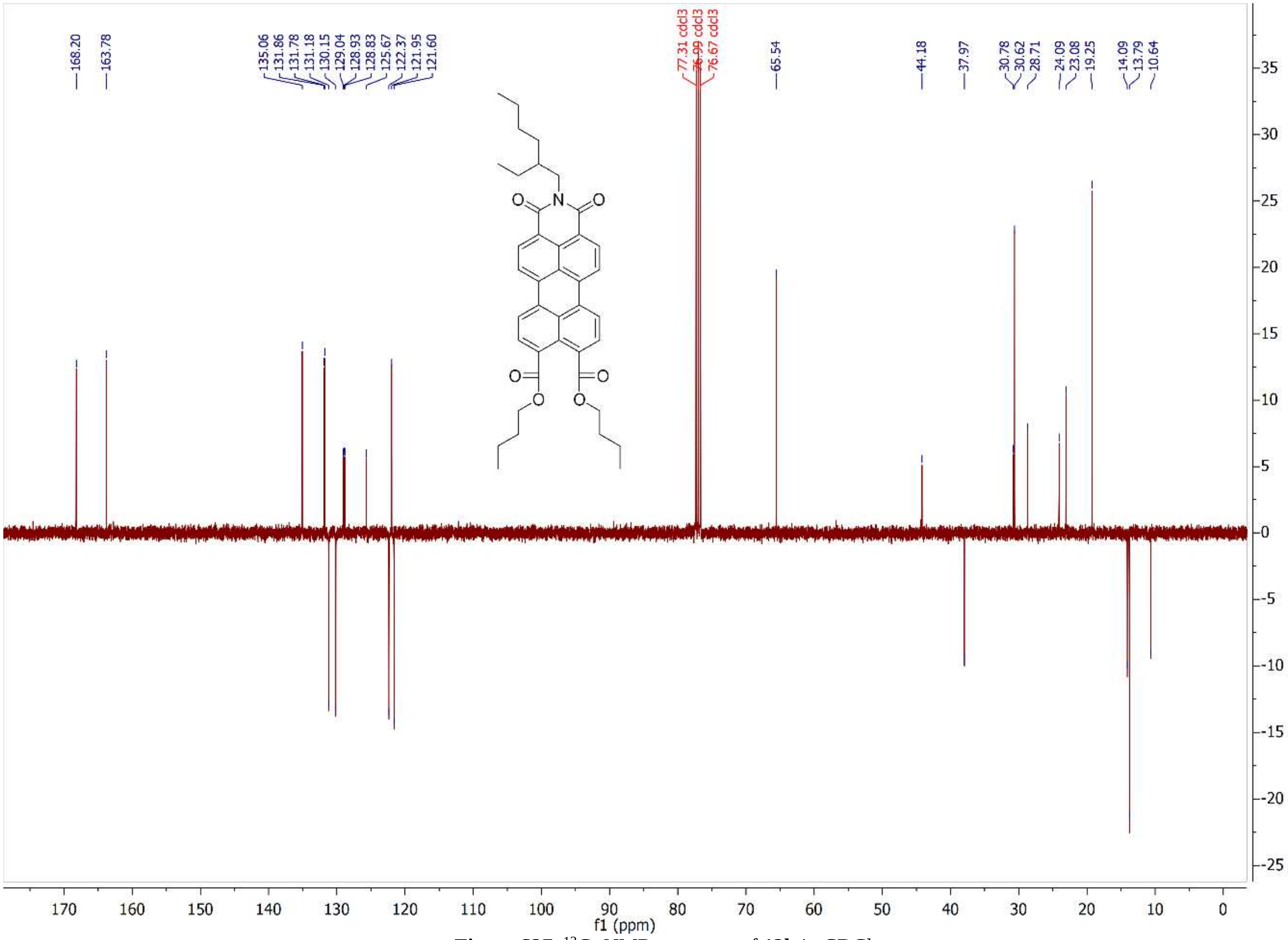

Figure S37. ^{13}C- NMR spectrum of **13b** in $CDCl_3$.

Acq. Data Name: WFJ-Cmp13b_21
Creation Parameters: Average(MS Time:0.34..0.39)

Experiment Date/Time: 11/1/2021 2:42:05 PM
Ionization Mode: FD+(eiFi)

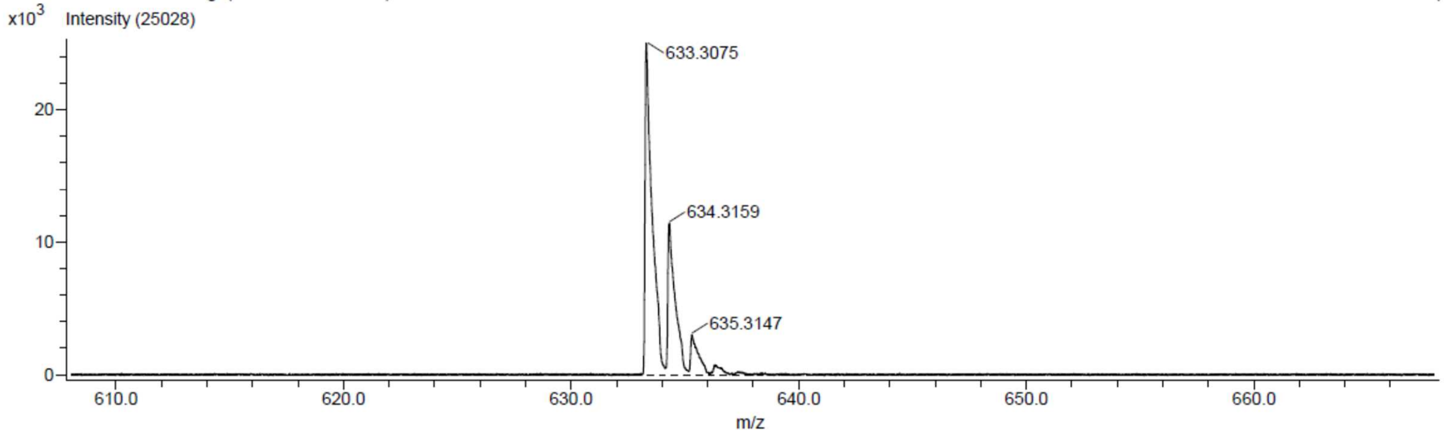

Formula: C40H43N1O6
Mono Isotopic Mass: 633.3090366

Addition/Desorption Ion: None
Charge Number: 1

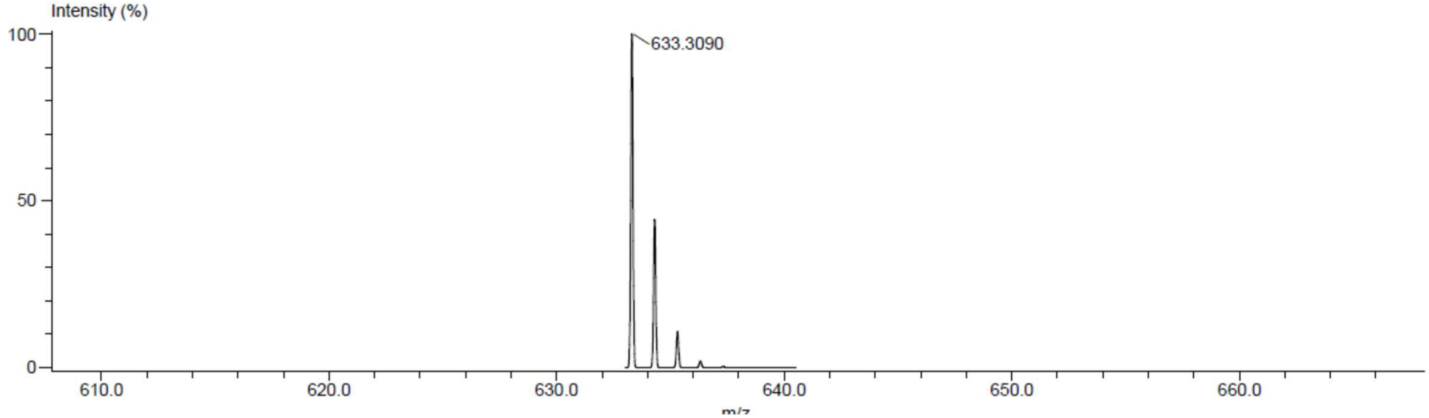

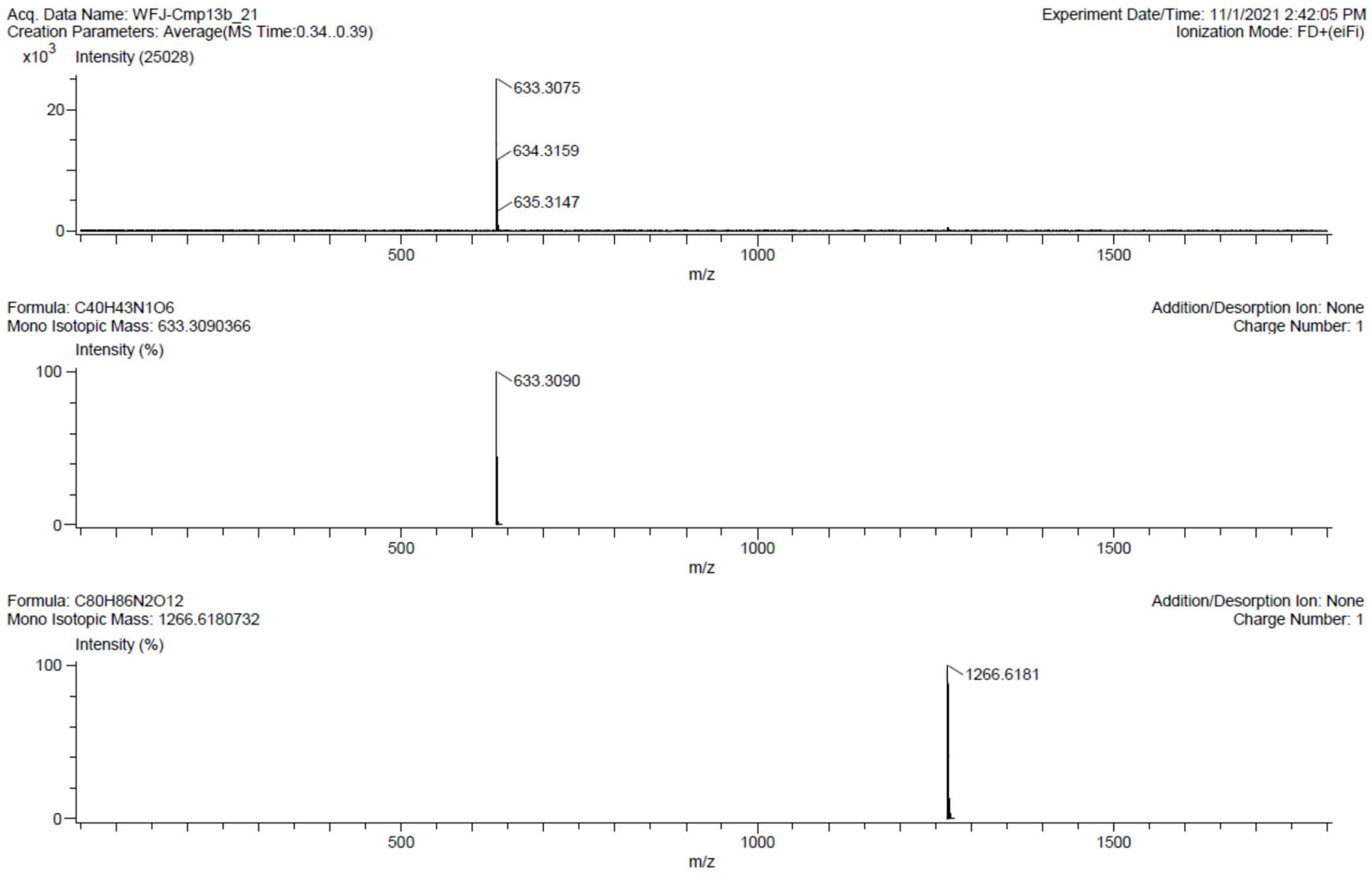

Figure S38. Mass spectrum of compound **13b**

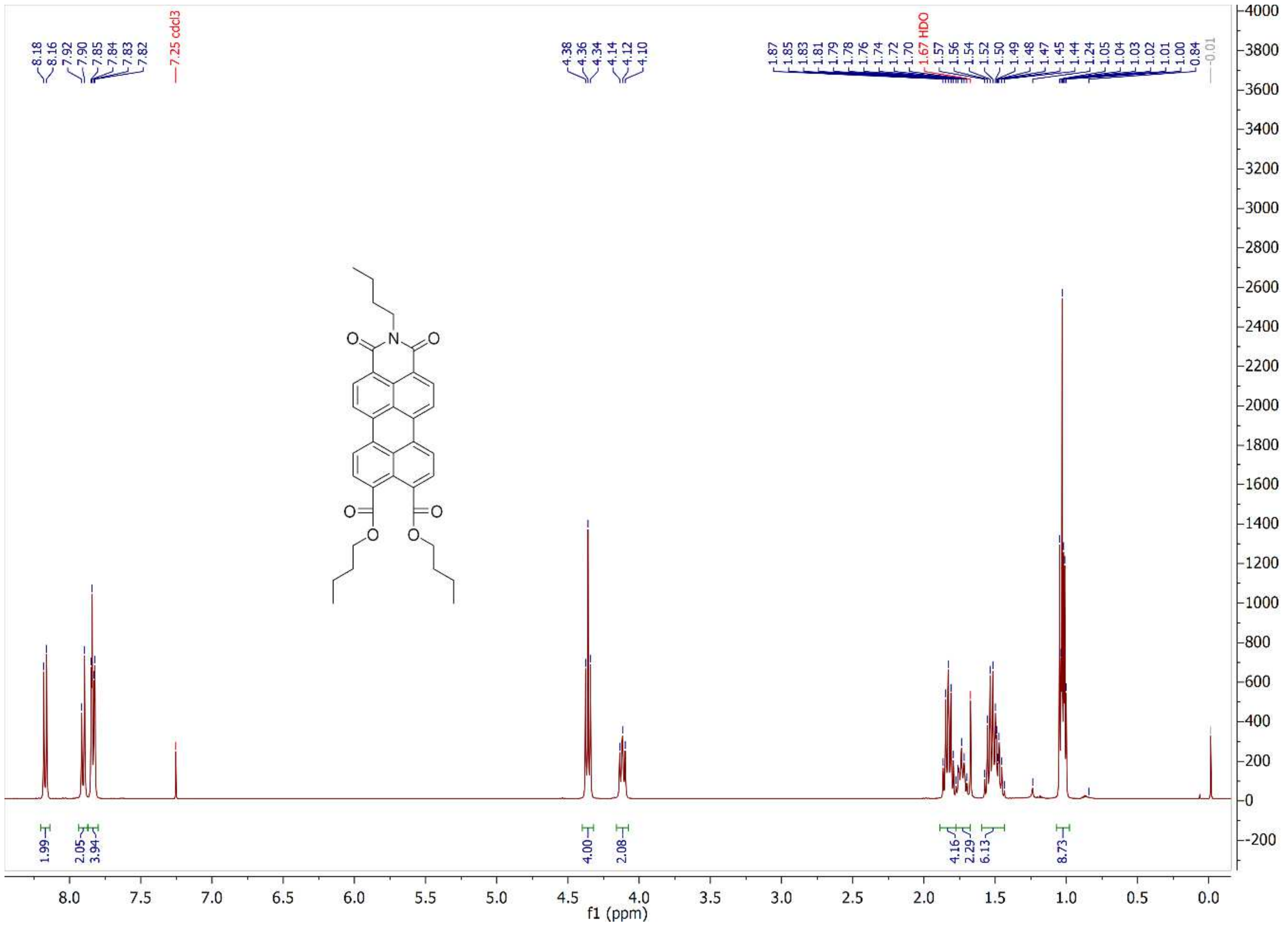
8.18
8.16
7.92
7.90
7.85
7.84
7.83
7.82
7.25 cdcl3
4.38
4.36
4.34
4.14
4.12
4.10
1.87
1.85
1.83
1.81
1.79
1.78
1.76
1.74
1.72
1.70
1.67 HDO
1.57
1.56
1.54
1.52
1.50
1.49
1.48
1.47
1.45
1.44
1.24
1.05
1.04
1.03
1.02
1.01
1.00
0.84
-0.01
1.99
2.05
3.94
4.00
2.08
4.16
2.29
6.13
8.73
f1 (ppm)

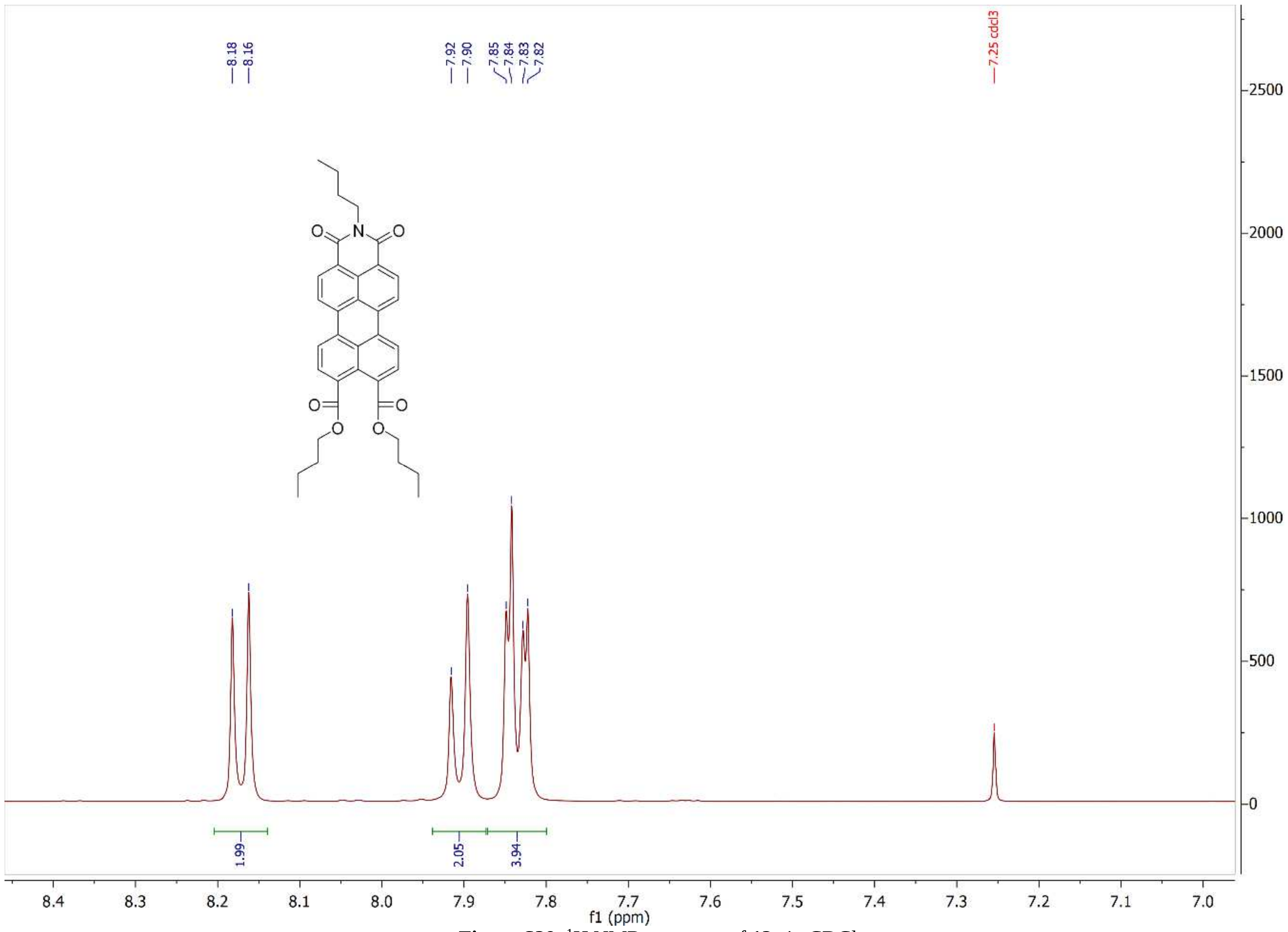

Figure S39. ^{1}H NMR spectrum of **13c** in $CDCl_3$.

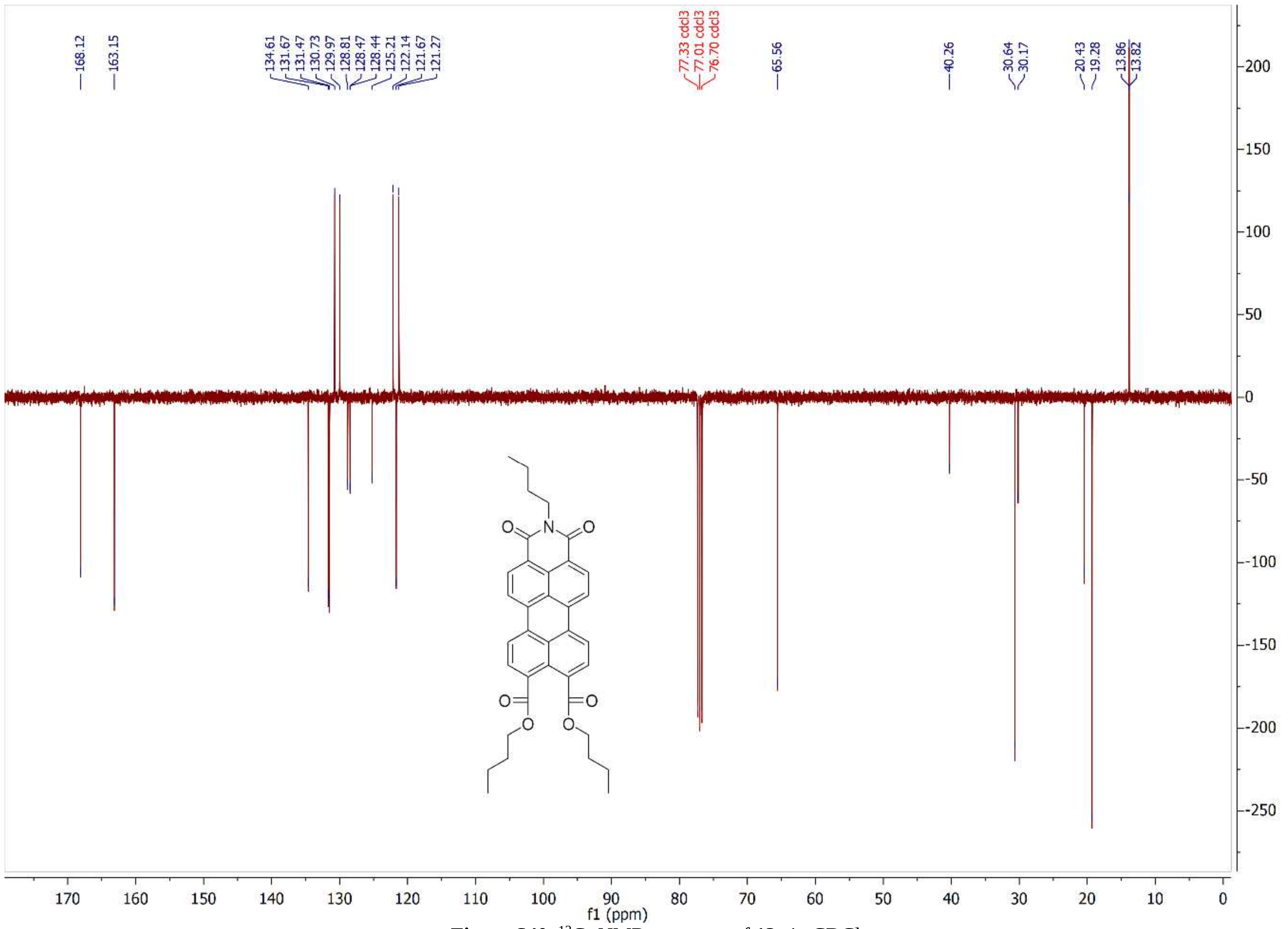

Figure S40. ^{13}C- NMR spectrum of **13c** in $CDCl_3$.

Acq. Data Name: WFJ-Cmp13c_21
Creation Parameters: Average(MS Time:0.46..0.54)

Experiment Date/Time: 11/1/2021 2:47:05 PM
Ionization Mode: FD+(eiFi)

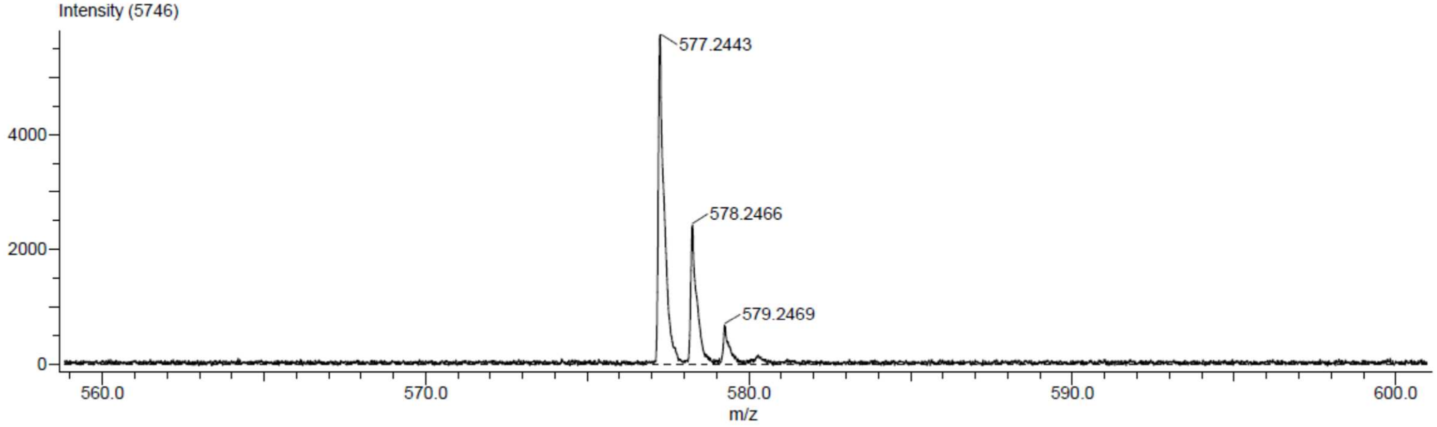

Formula: C36H35N1O6
Mono Isotopic Mass: 577.2464366

Addition/Desorption Ion: None
Charge Number: 1

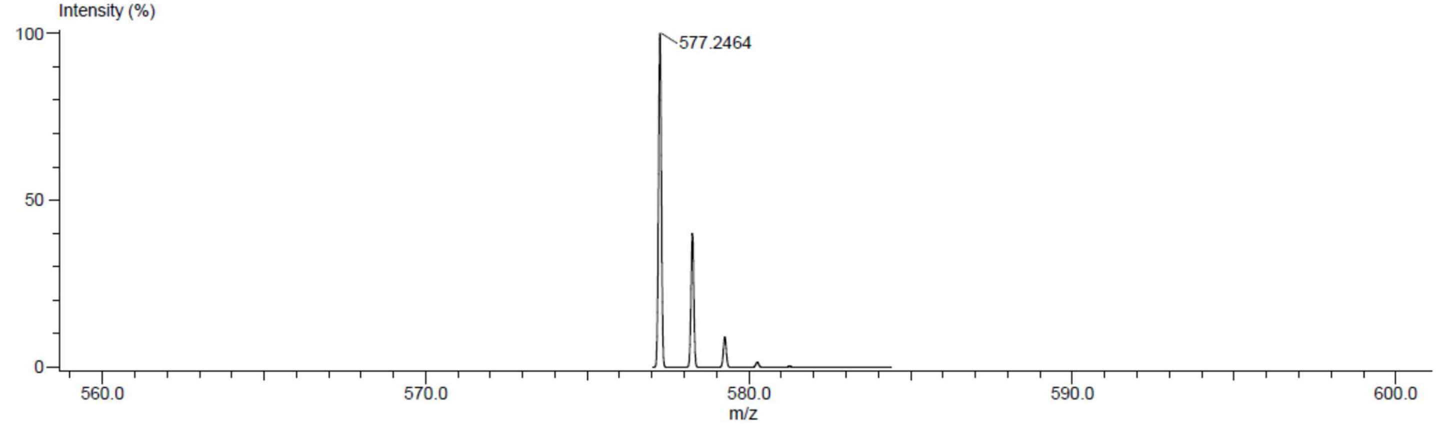

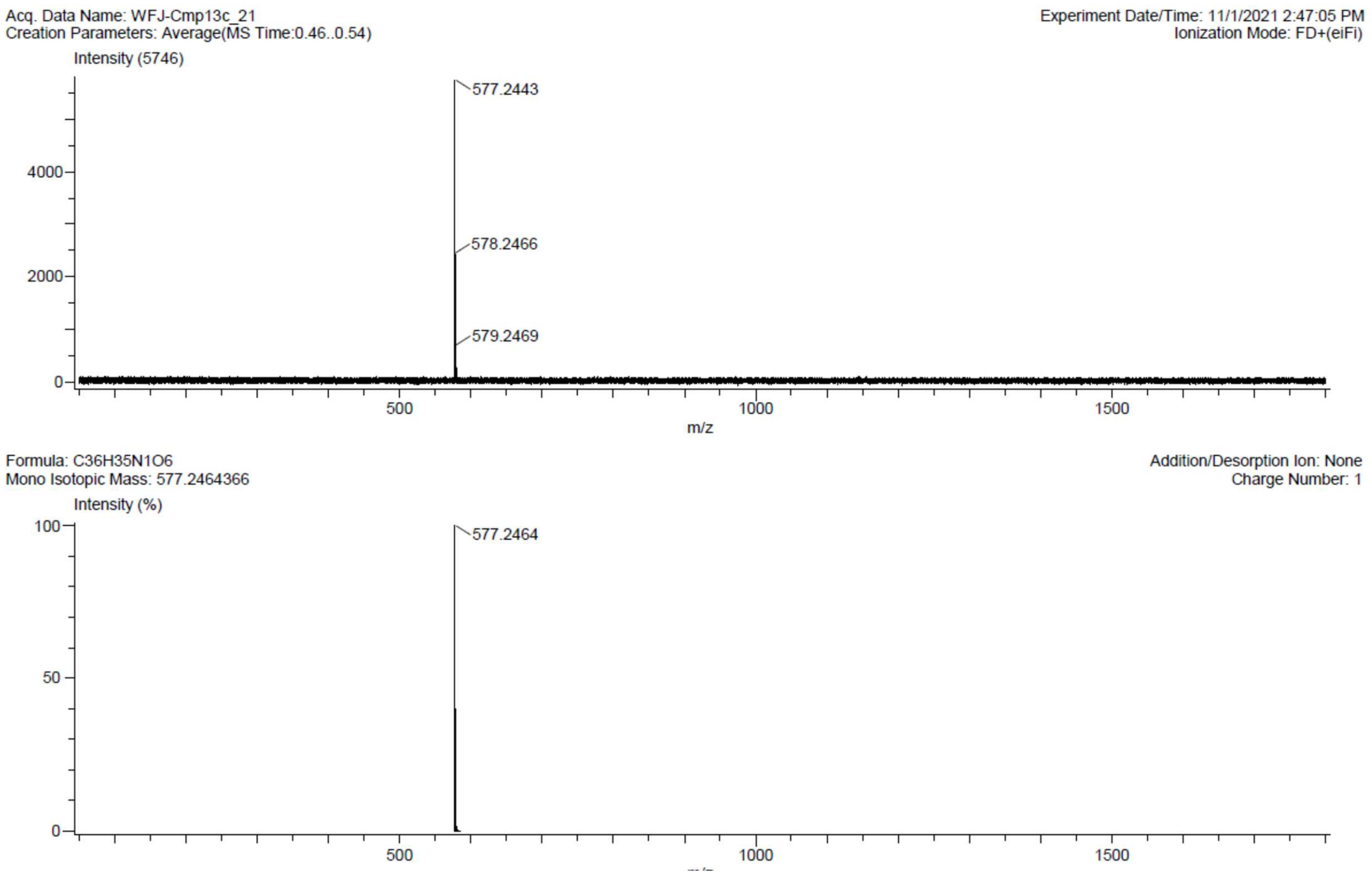

Figure S41. Mass spectrum of compound **13c**.

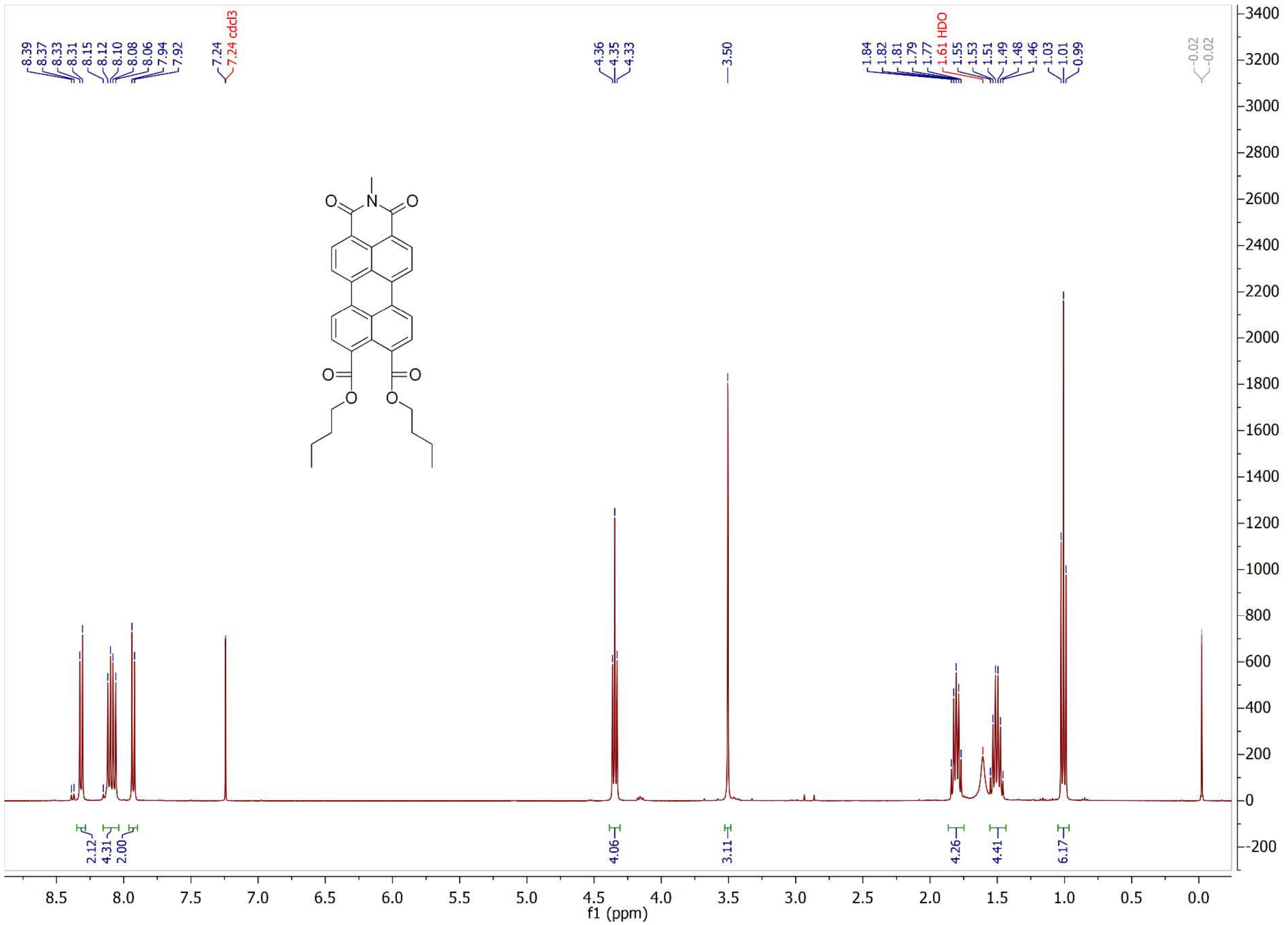
8.39
8.37
8.33
8.31
8.15
8.12
8.10
8.08
8.06
7.94
7.92
7.24
7.24 cdcl3
4.36
4.35
4.33
3.50
1.84
1.82
1.81
1.79
1.77
1.61 HDO
1.55
1.53
1.51
1.49
1.48
1.46
1.03
1.01
0.99
-0.02
-0.02
2.12
4.31
2.00
4.06
3.11
4.26
4.41
6.17
f1 (ppm)

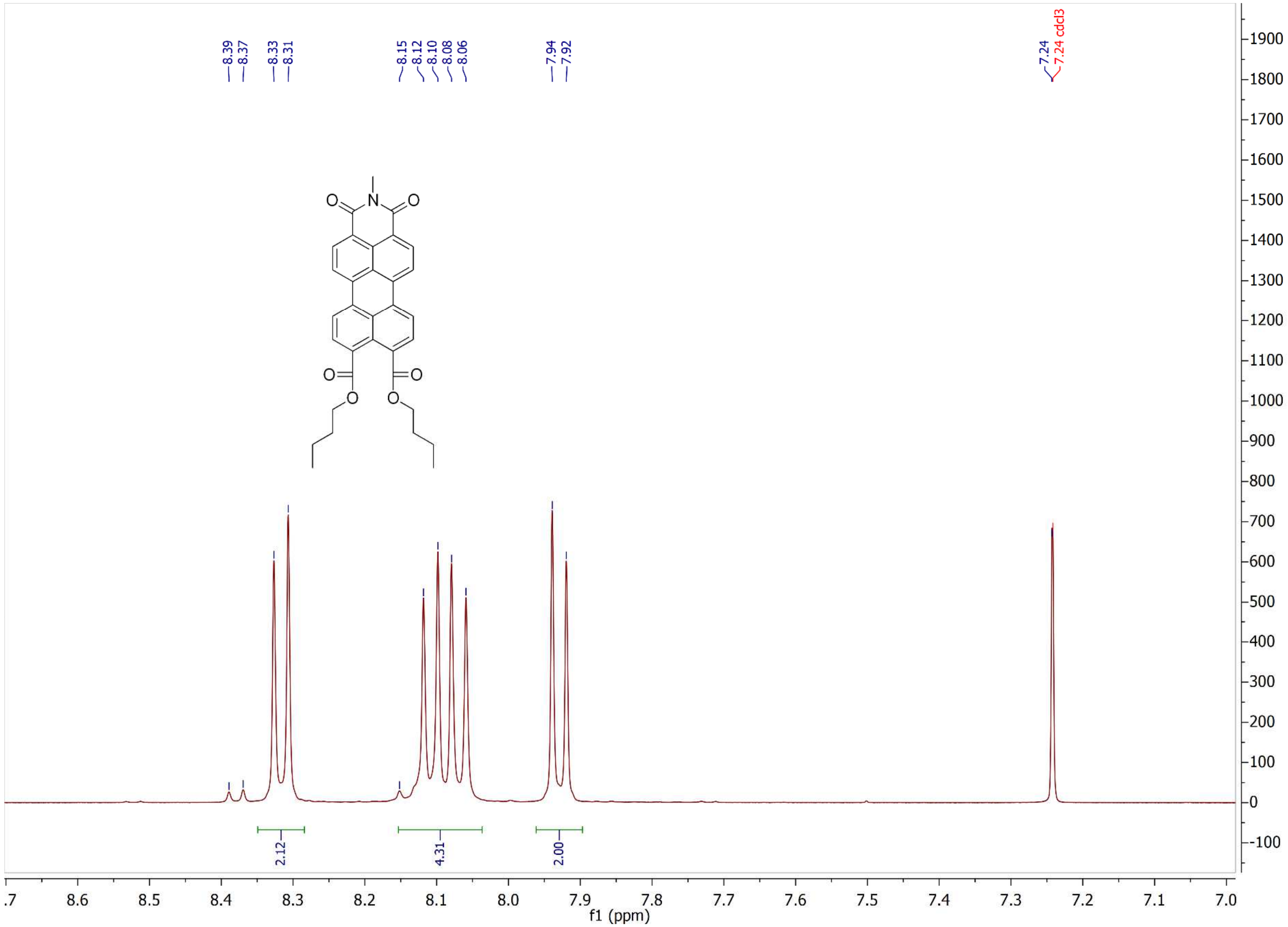

Figure S42. ^{1}H NMR spectrum of **13d** in $CDCl_3$.

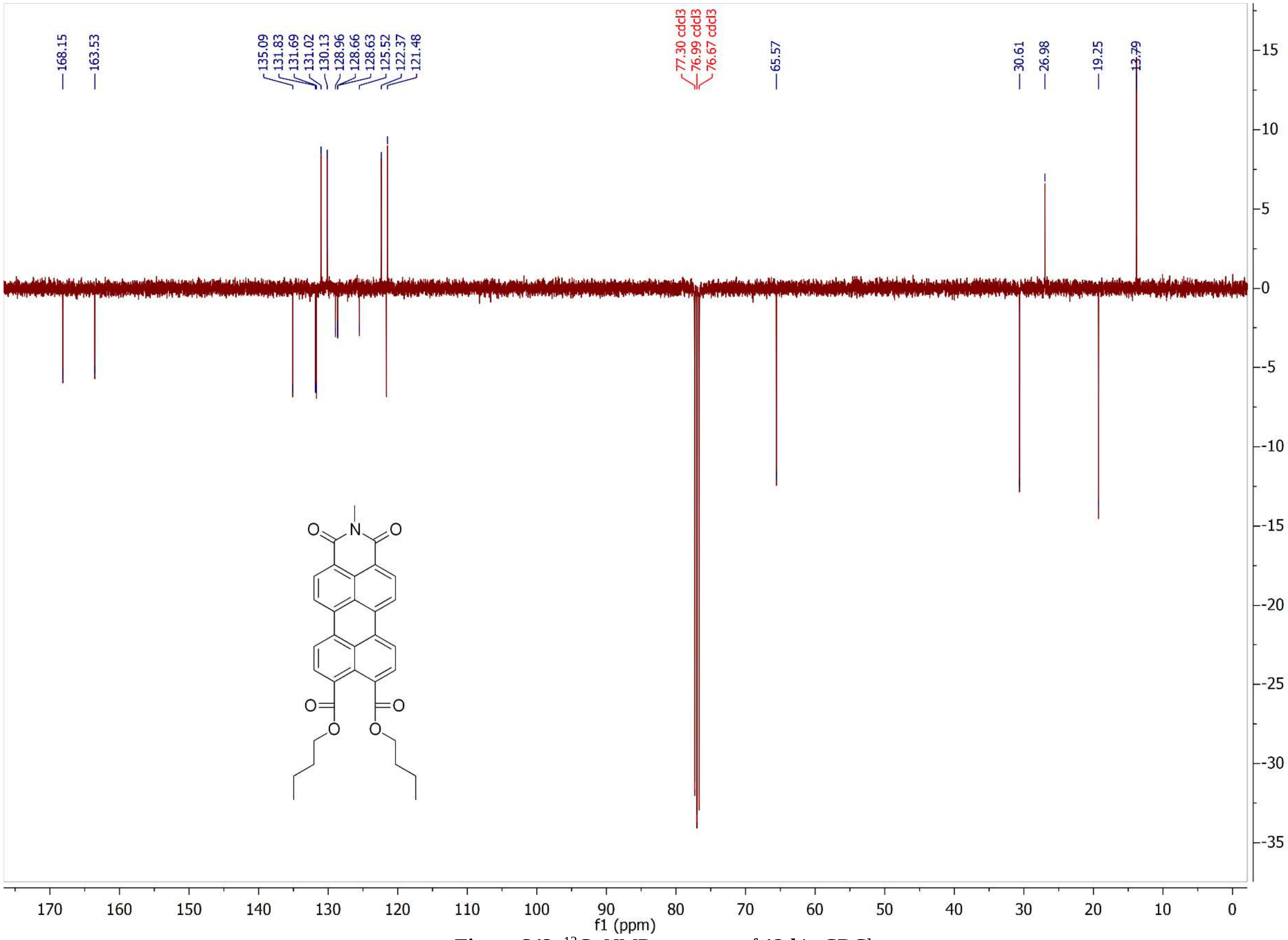

Figure S43. ^{13}C- NMR spectrum of **13d** in $CDCl_3$.

Acq. Data Name: WFJ-Cmp13d_21
Creation Parameters: Average(MS Time:0.55..0.63)

Experiment Date/Time: 11/1/2021 2:51:00 PM
Ionization Mode: FD+(eiFi)

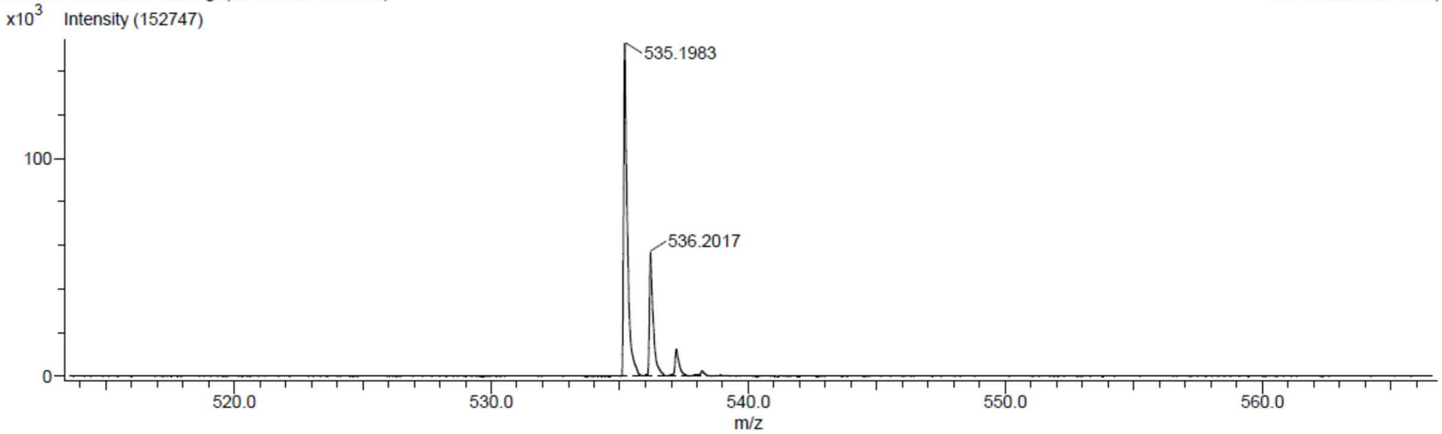

Formula: C33H29N1O6
Mono Isotopic Mass: 535.1994866

Addition/Desorption Ion: None
Charge Number: 1

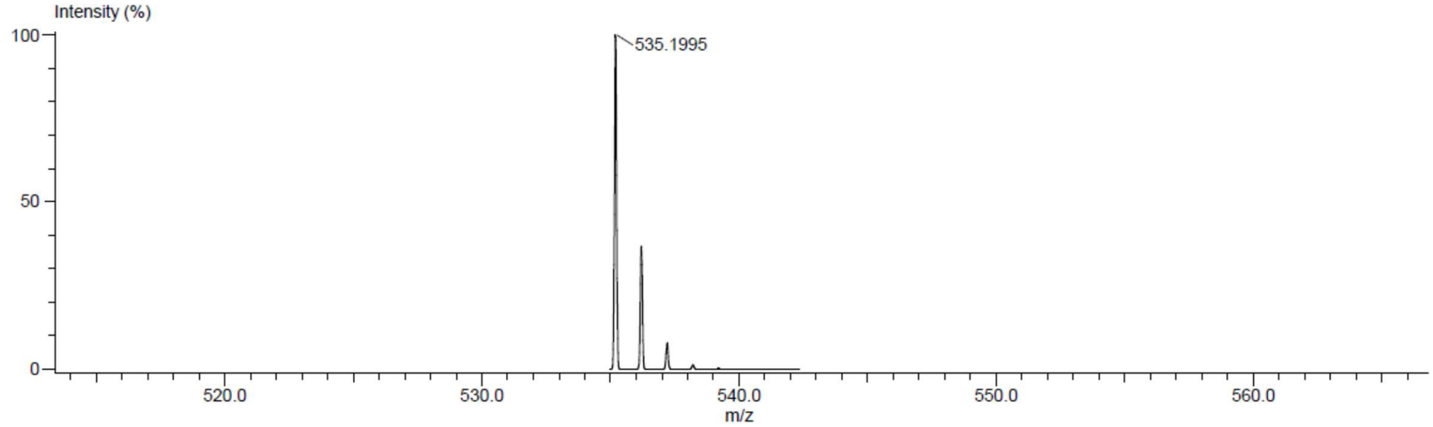

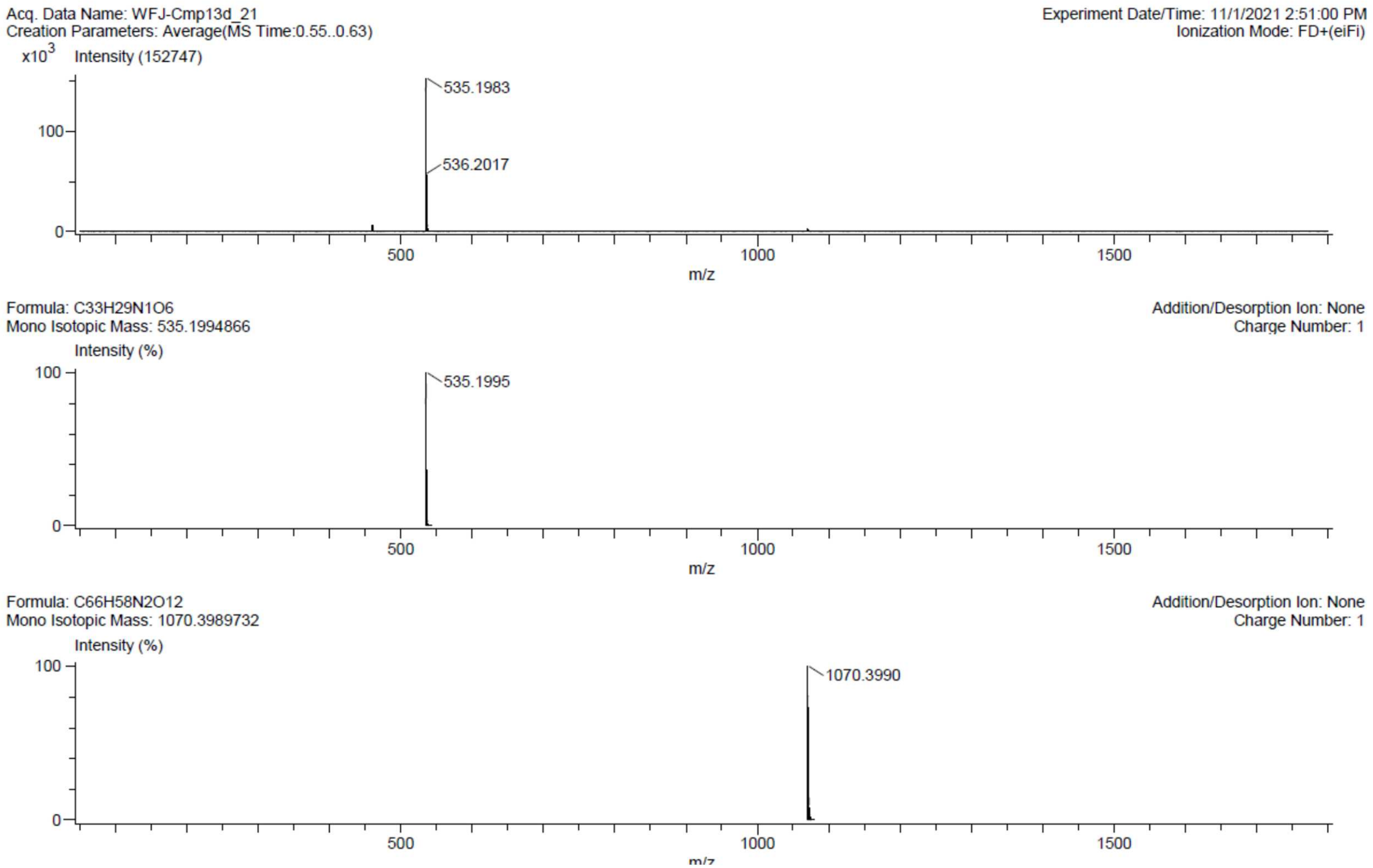

Figure S44. Mass spectrum of compound **13d**.

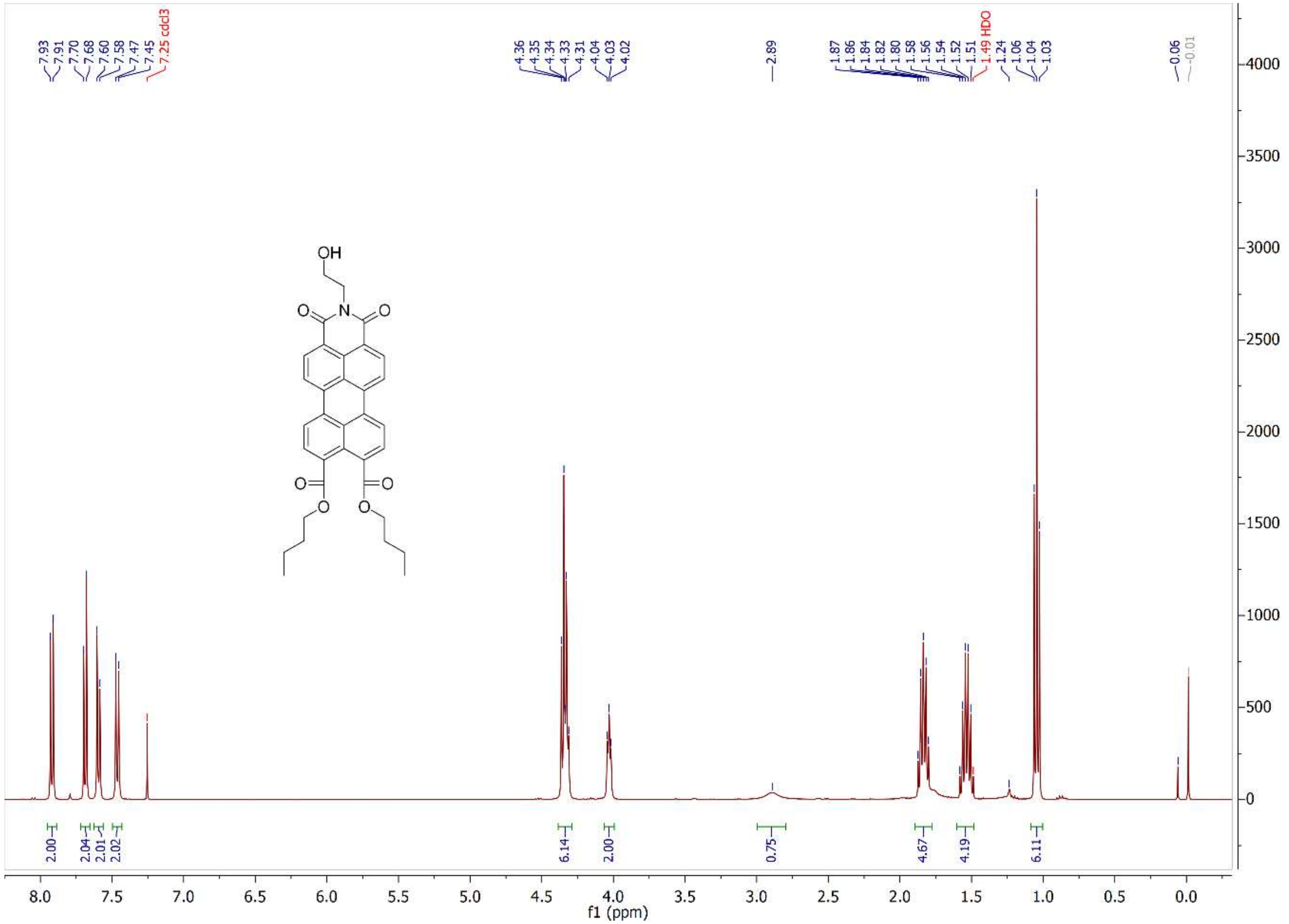

7.93
7.91
7.70
7.68
7.60
7.58
7.47
7.45
7.25 cdcl3
4.36
4.35
4.34
4.33
4.31
4.04
4.03
4.02
2.89
1.87
1.86
1.84
1.82
1.80
1.58
1.56
1.54
1.52
1.51
1.49 HDO
1.24
1.06
1.04
1.03
0.06
-0.01
OH
O
N
O
O
O
O
O
2.00
2.04
2.01
2.02
6.14
2.00
0.75
4.67
4.19
6.11
f1 (ppm)

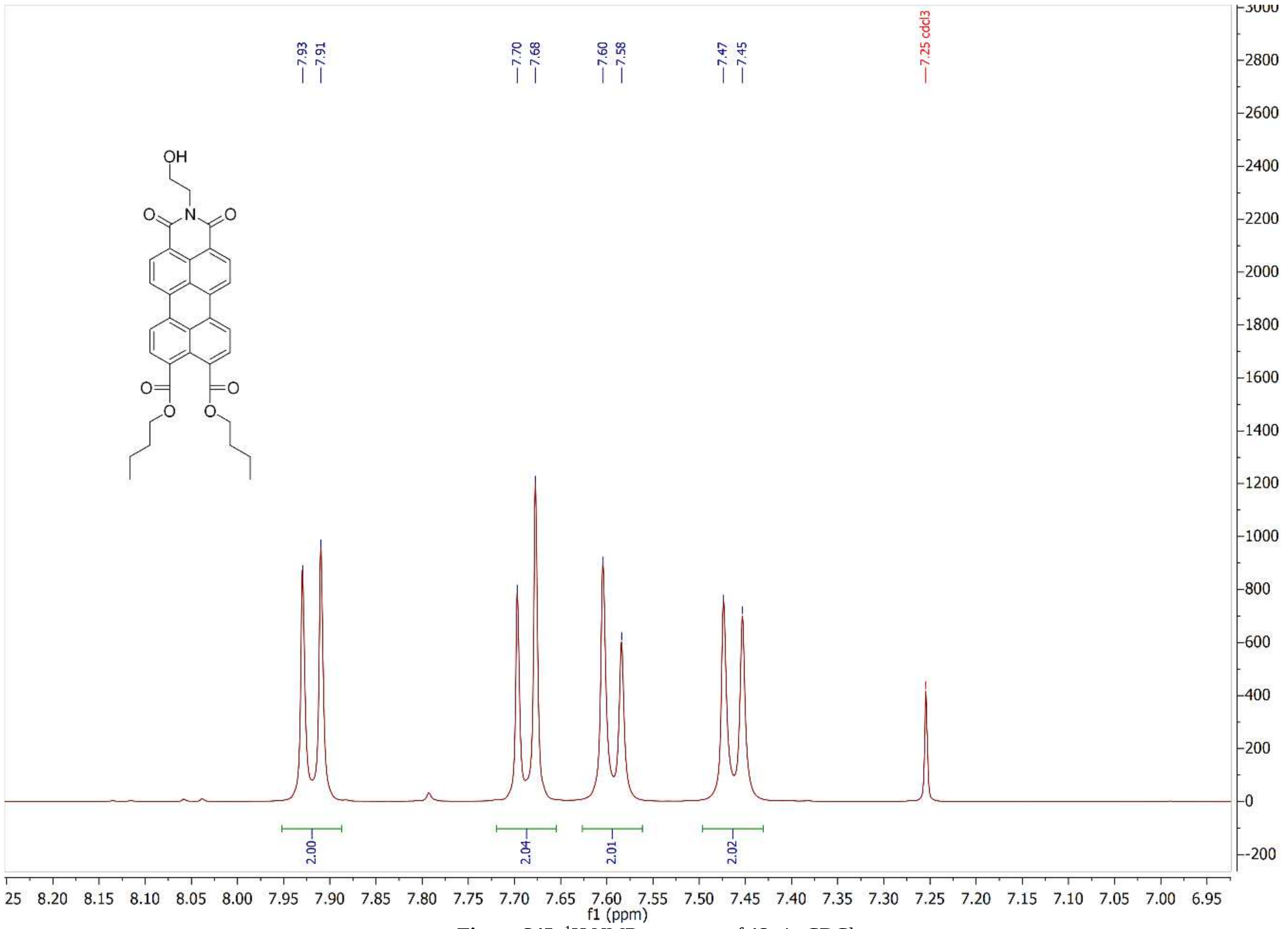

Figure S45. ^{1}H NMR spectrum of **13e** in $CDCl_3$.

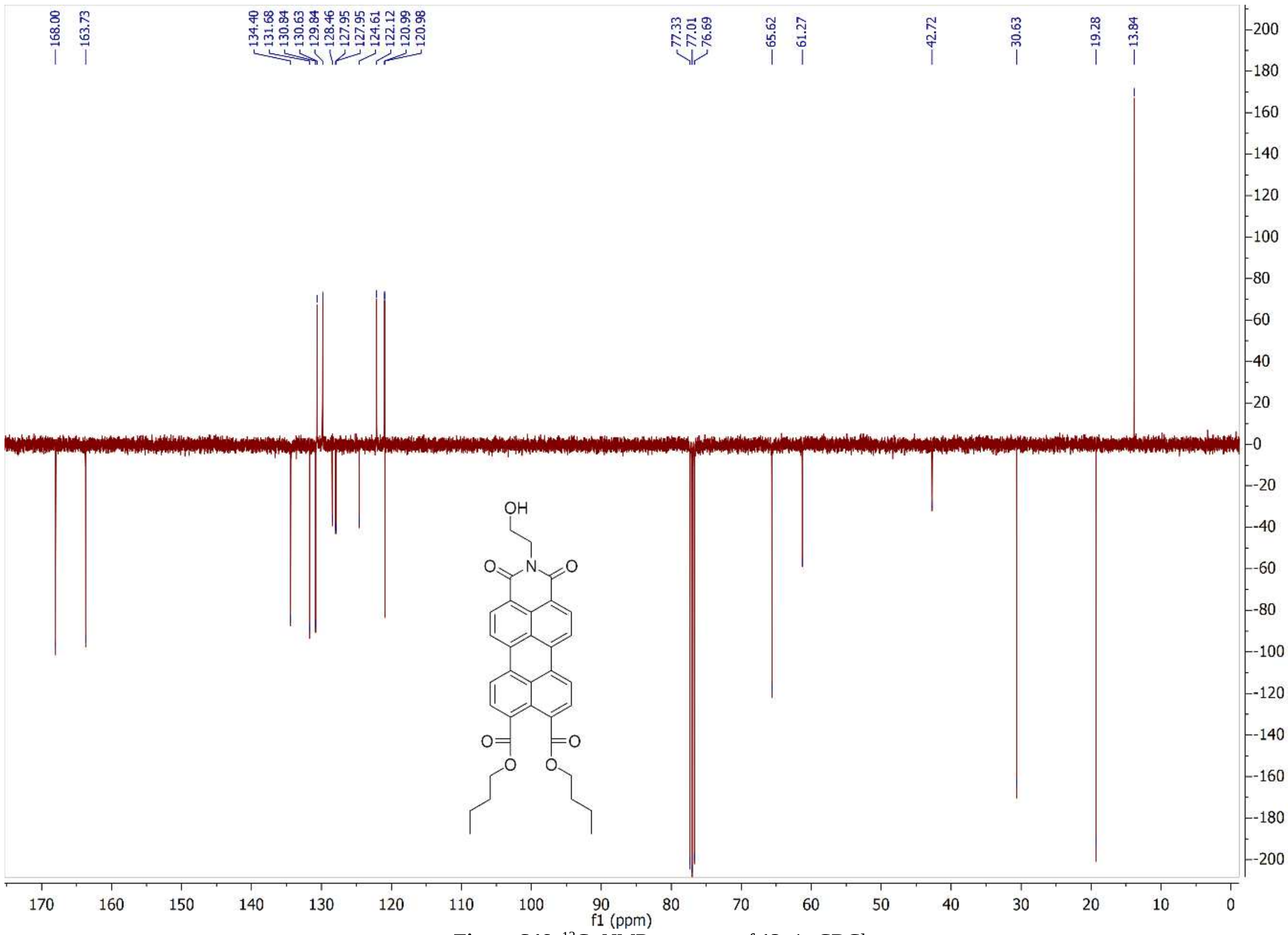

Figure S46. ^{13}C- NMR spectrum of **13e** in $CDCl_3$.

Acq. Data Name: WFJ-Cmp13e_21
Creation Parameters: Average(MS Time:0.43..0.62)

Experiment Date/Time: 11/1/2021 2:55:06 PM
Ionization Mode: FD+(eiFi)

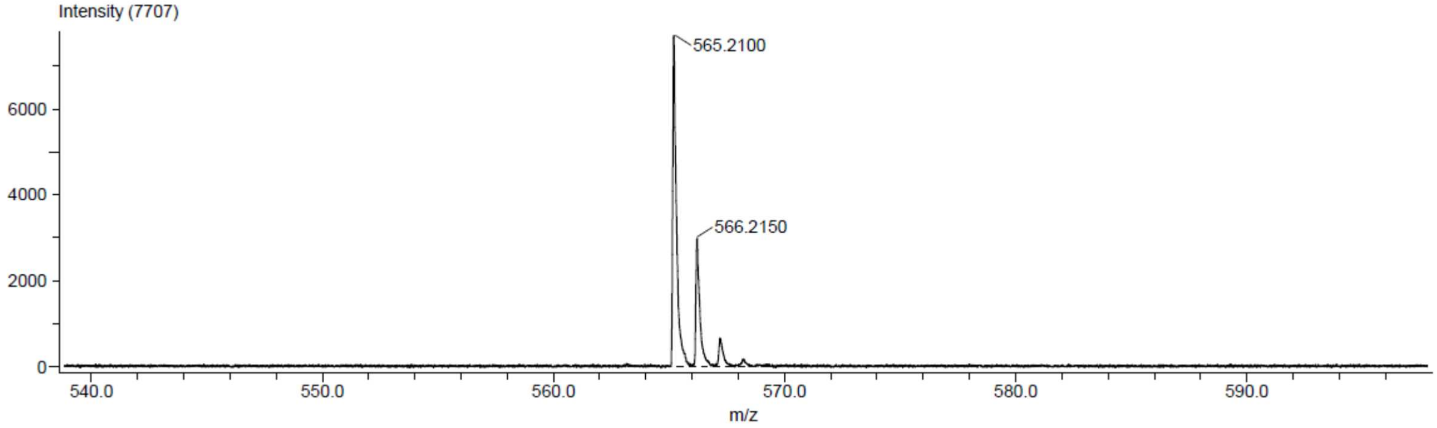

Formula: C34H31N1O7
Mono Isotopic Mass: 565.2100512

Addition/Desorption Ion: None
Charge Number: 1

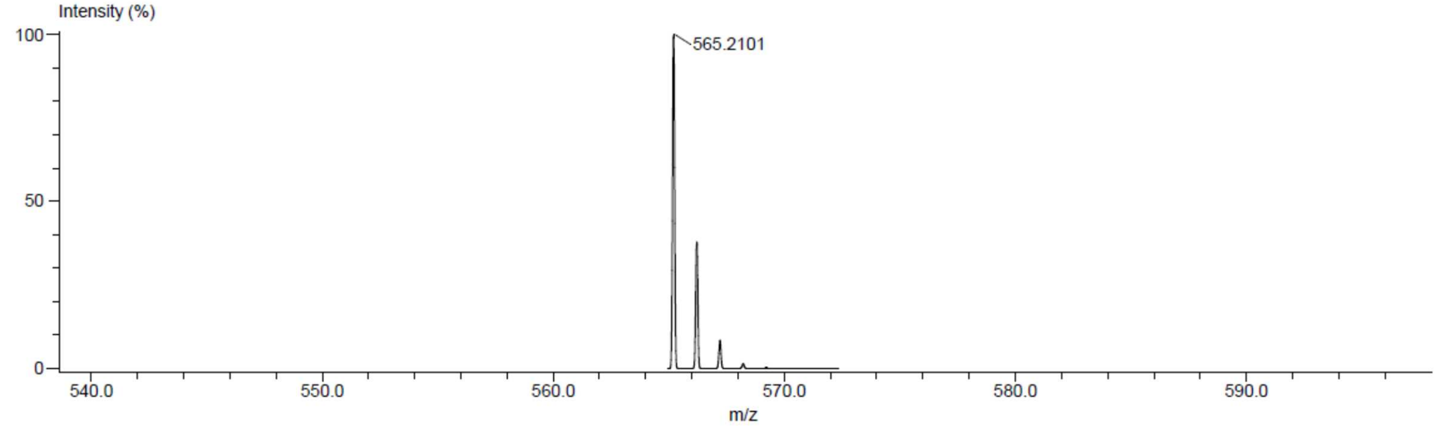

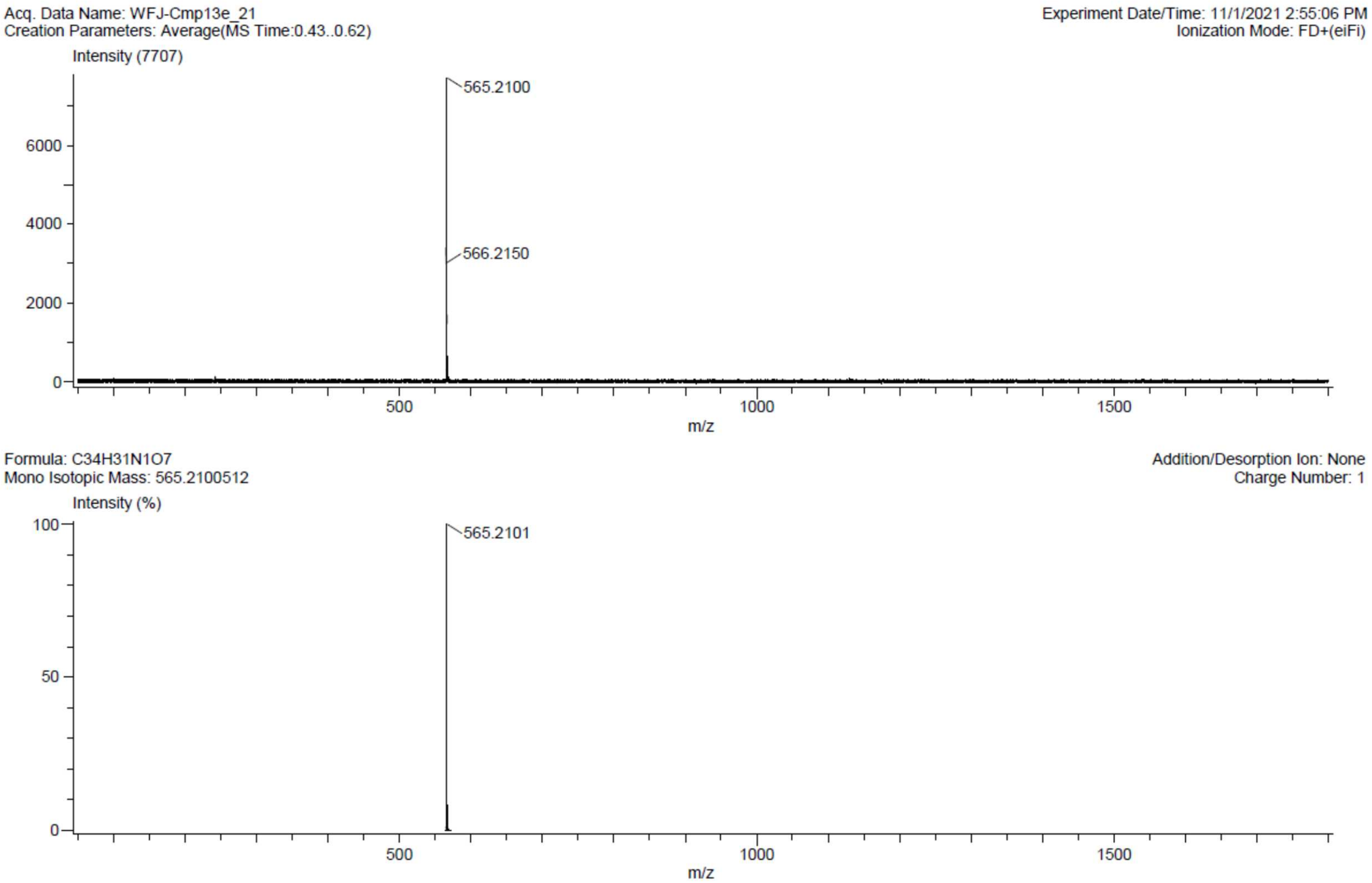

Figure S47. Mass spectrum of compound **13e**.

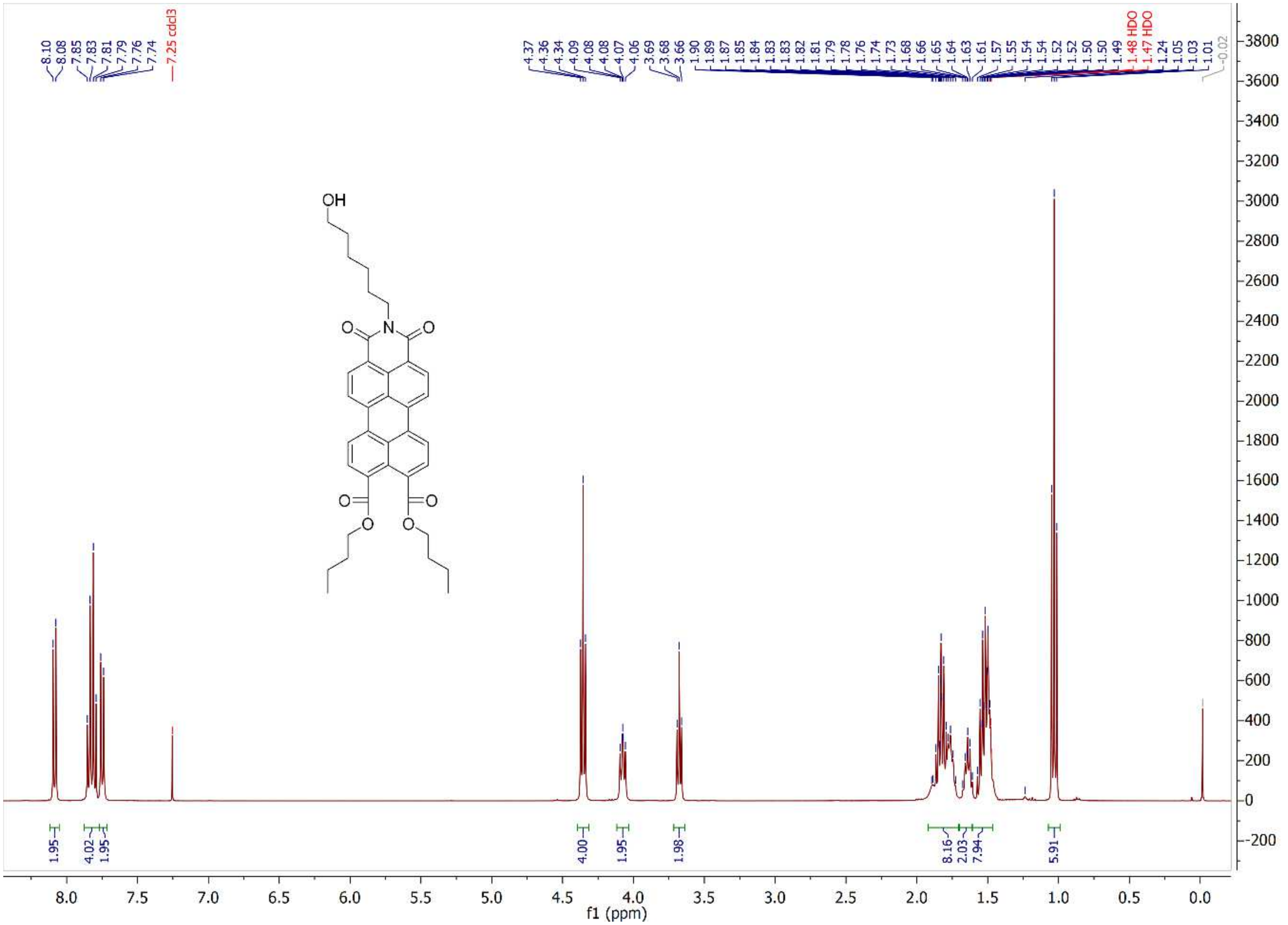
8.10
8.08
7.85
7.83
7.81
7.79
7.76
7.74
7.25 cdcl3
4.37
4.36
4.34
4.09
4.08
4.08
4.07
4.06
3.69
3.68
3.66
1.90
1.89
1.87
1.85
1.84
1.83
1.83
1.82
1.81
1.79
1.78
1.76
1.74
1.73
1.68
1.66
1.65
1.64
1.63
1.61
1.57
1.55
1.54
1.54
1.52
1.52
1.50
1.50
1.49
1.48 HDO
1.47 HDO
1.24
1.05
1.03
1.01
-0.02
OH
N
O
1.95
4.02
1.95
4.00
1.95
1.98
8.16
2.03
7.94
5.91
f1 (ppm)

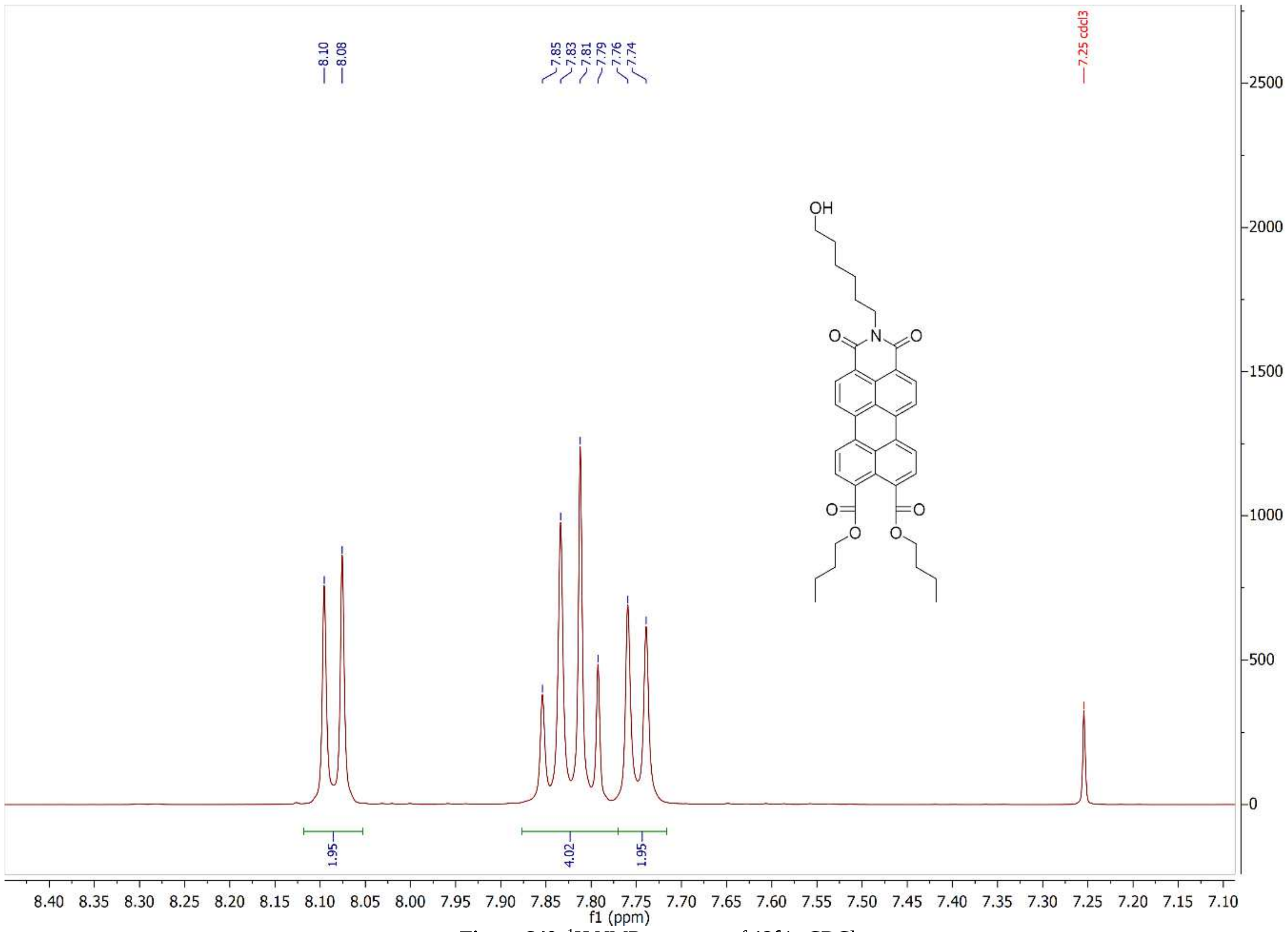

Figure S48. ^{1}H NMR spectrum of **13f** in $CDCl_3$.

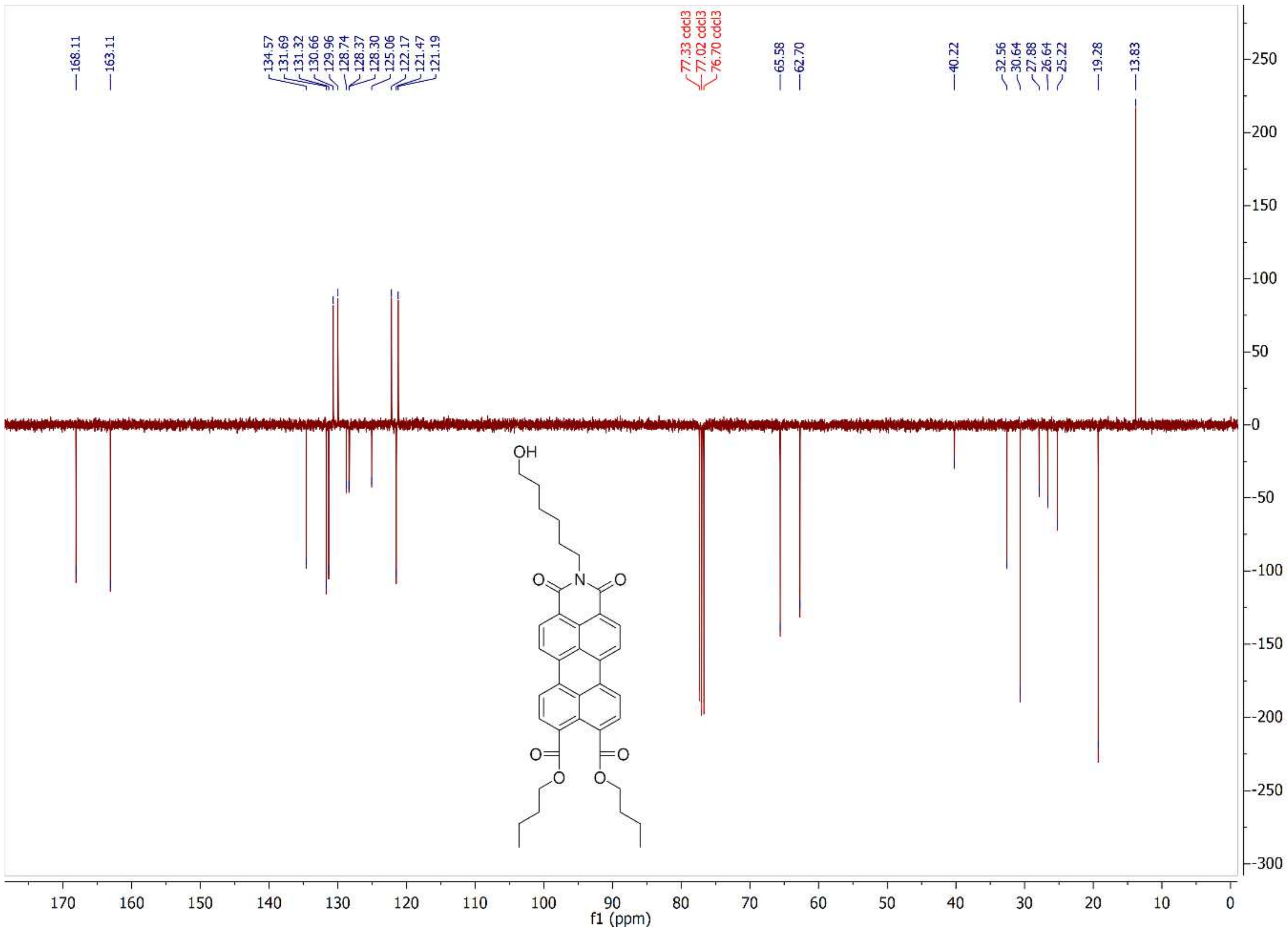

Figure S49. ^{13}C- NMR spectrum of **13f** in $CDCl_3$.

Acq. Data Name: WFJ-Cmp13f_21
Creation Parameters: Average(MS Time:0.80..0.85)

Experiment Date/Time: 11/1/2021 2:59:08 PM
Ionization Mode: FD+(eiFi)

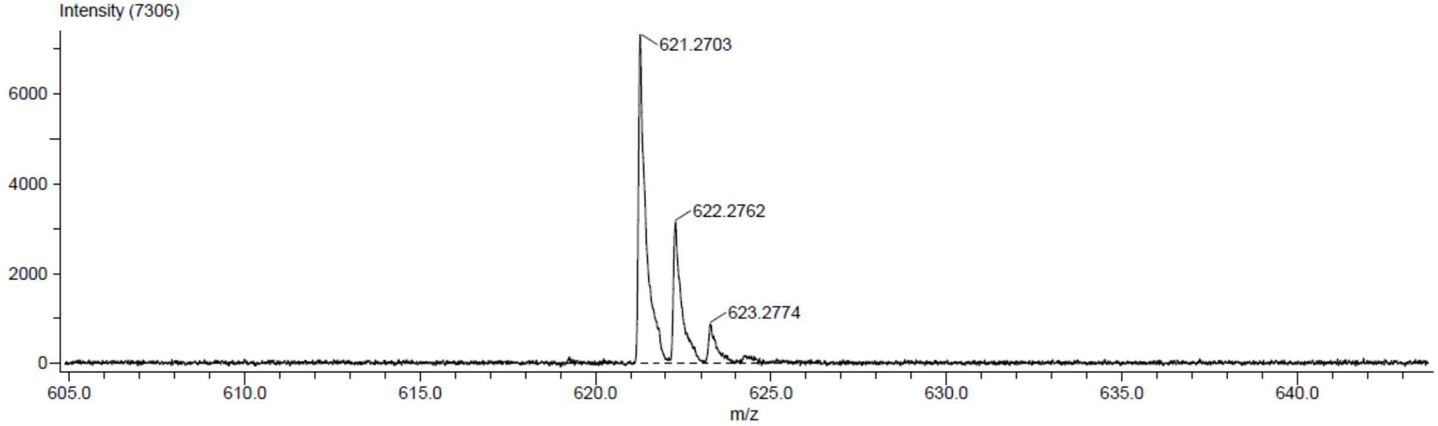

Formula: C38H39N1O7
Mono Isotopic Mass: 621.2726512

Addition/Desorption Ion: None
Charge Number: 1

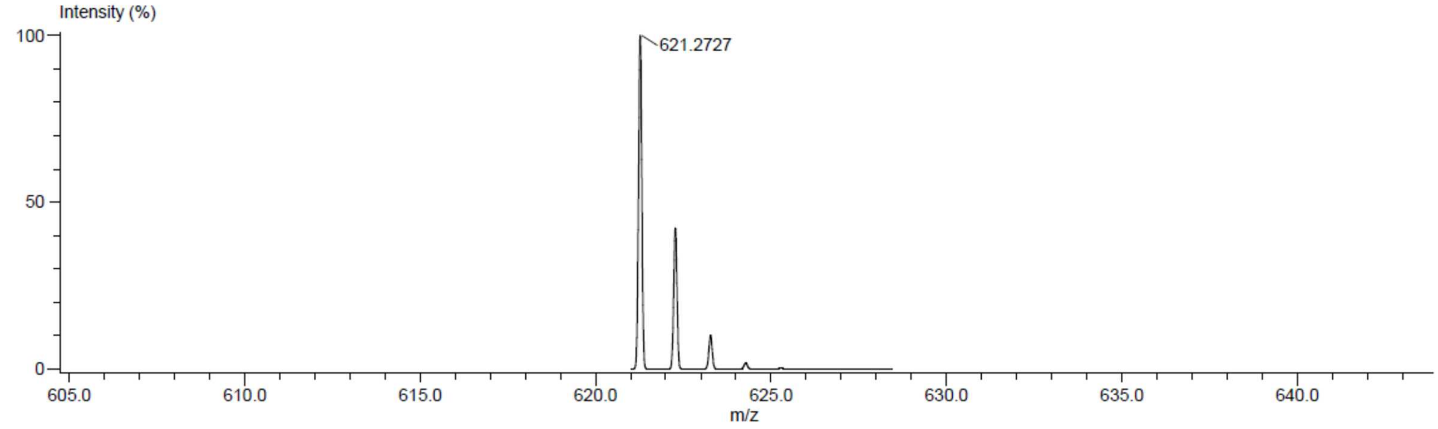

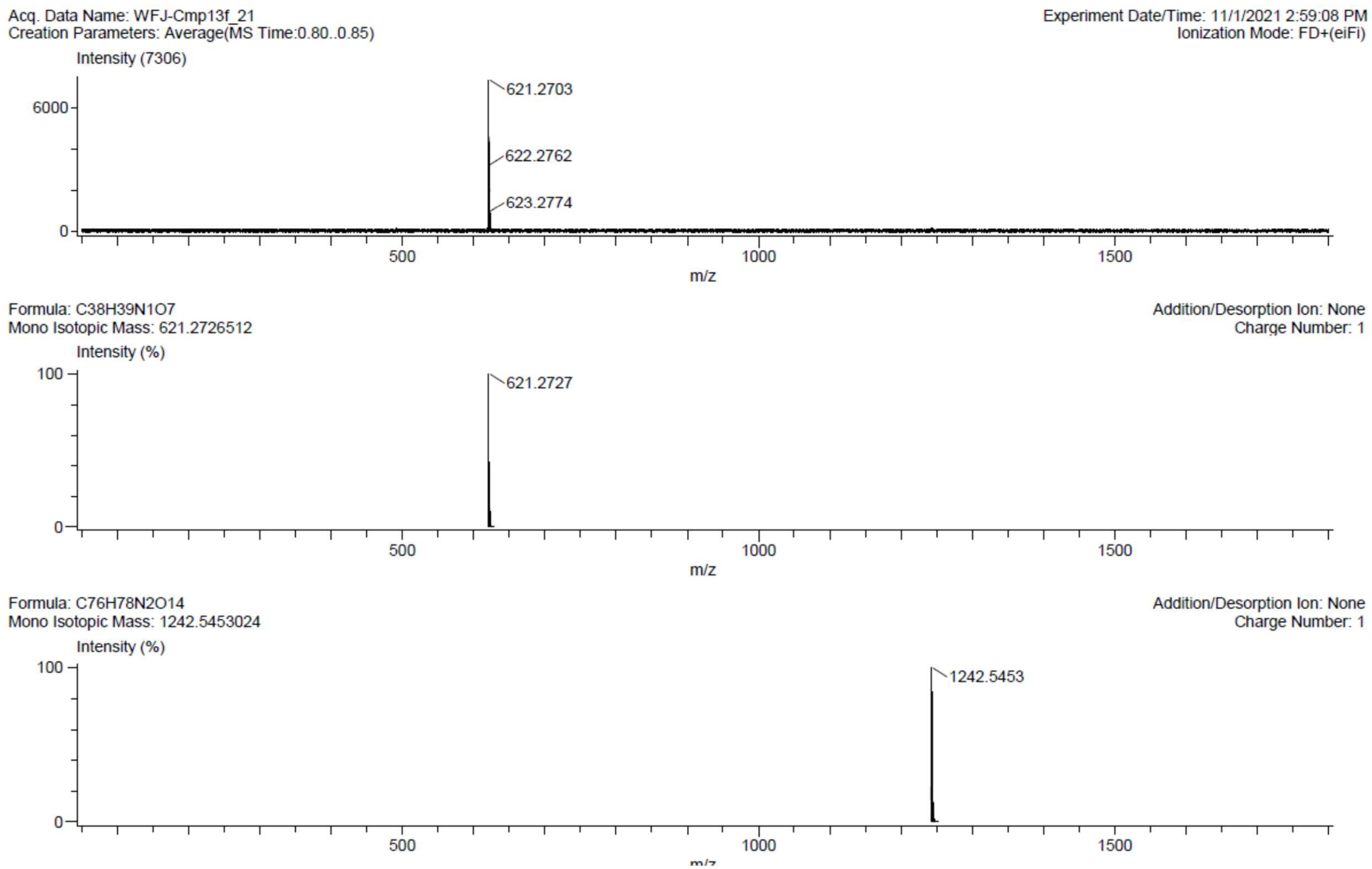

Figure S50. Mass spectrum of compound **13f**.

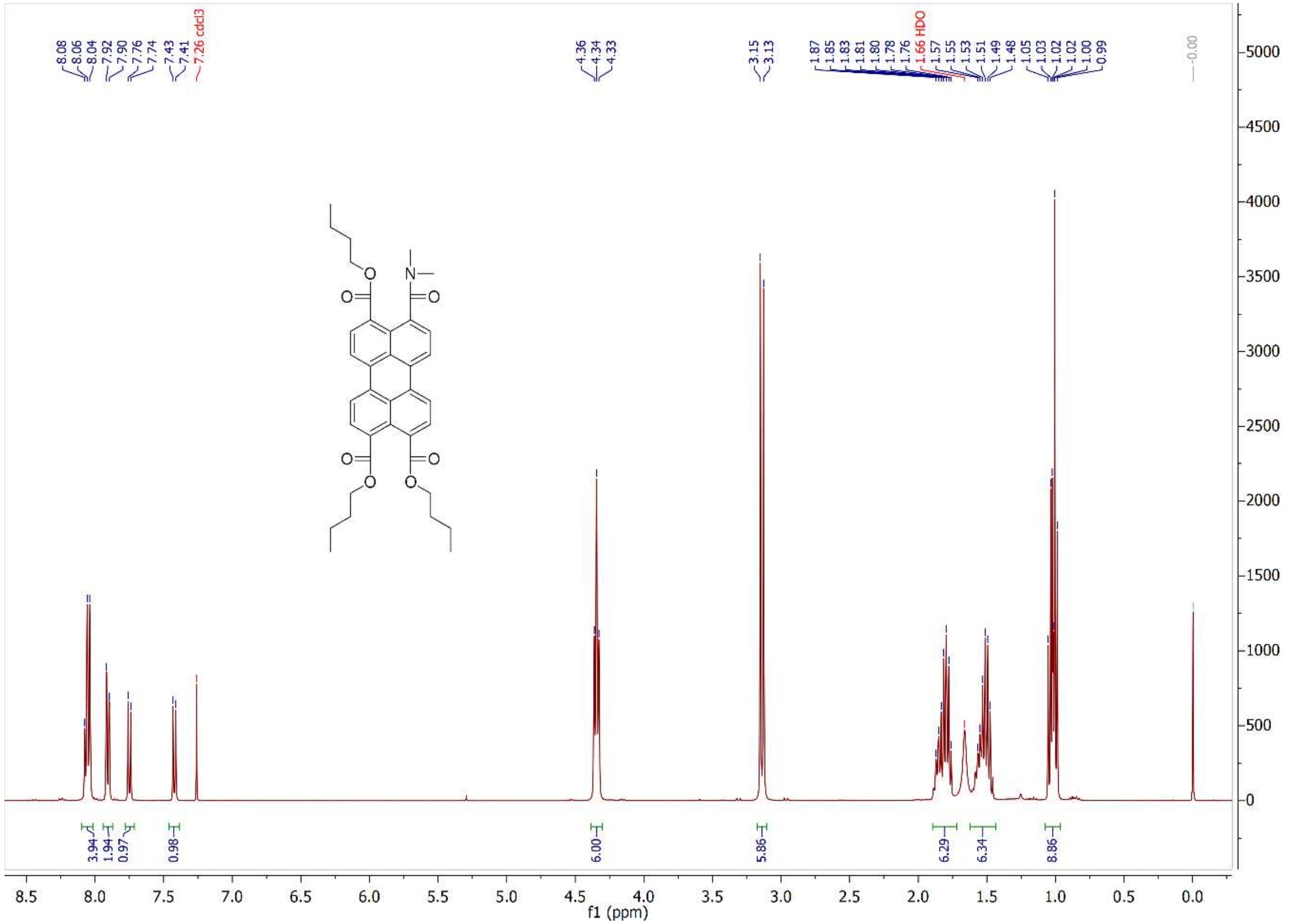

8.08
8.06
8.04
7.92
7.90
7.76
7.74
7.43
7.41
7.26 cdcl3
4.36
4.34
4.33
3.15
3.13
1.87
1.85
1.83
1.81
1.80
1.78
1.76
1.66 HDO
1.57
1.55
1.53
1.51
1.49
1.48
1.05
1.03
1.02
1.02
1.00
0.99
-0.00
3.94
1.94
0.97
0.98
6.00
5.86
6.29
6.34
8.86
f1 (ppm)

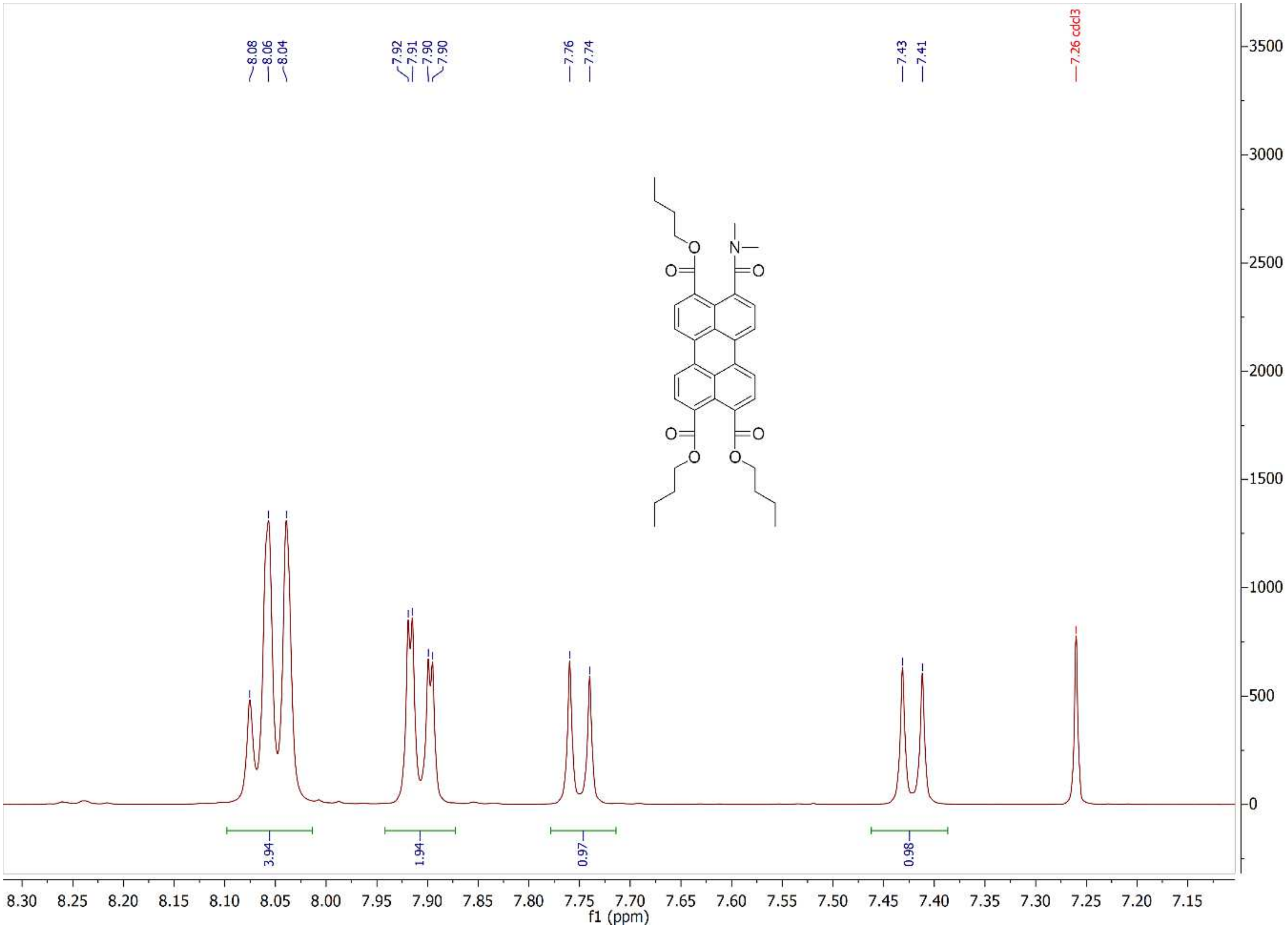

Figure S51. ^{1}H NMR spectrum of **14a** in $CDCl_3$.

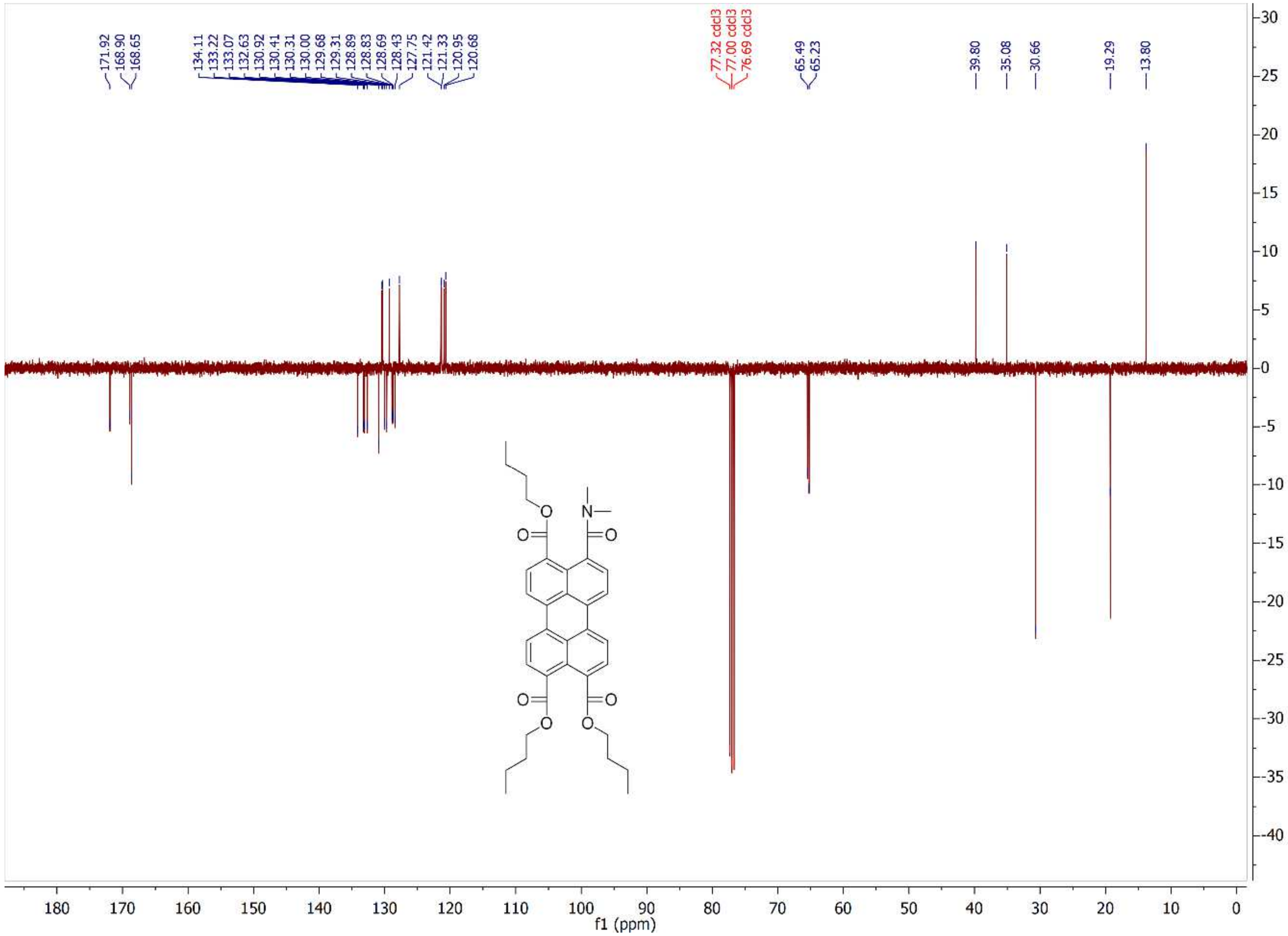

171.92
168.90
168.65
134.11
133.22
133.07
132.63
130.92
130.41
130.31
130.00
129.68
129.31
128.89
128.83
128.69
128.43
127.75
121.42
121.33
120.95
120.68
77.32 cdcl3
77.00 cdcl3
76.69 cdcl3
65.49
65.23
39.80
35.08
30.66
19.29
13.80
f1 (ppm)

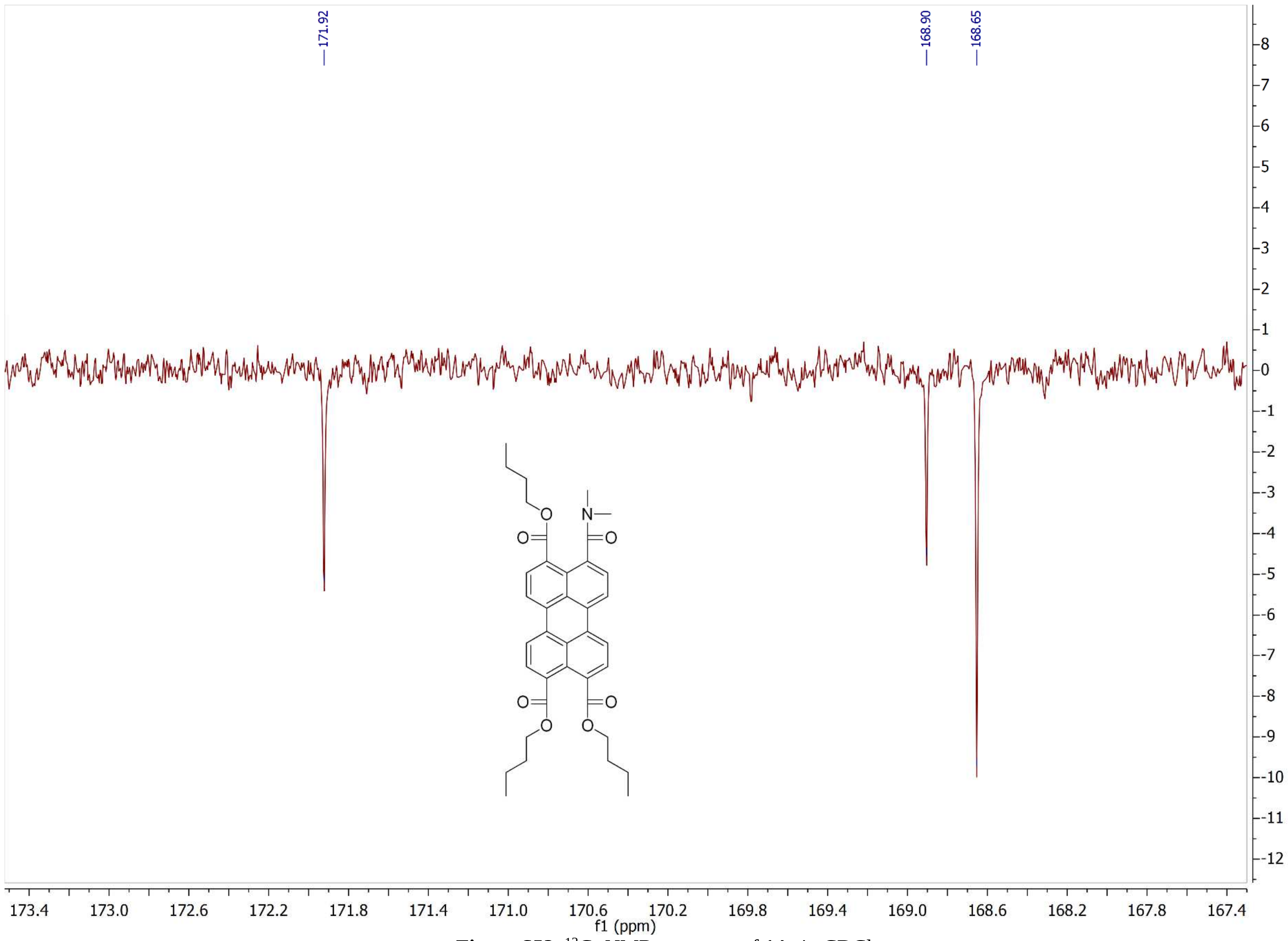

Figure S52. ^{13}C- NMR spectrum of **14a** in $CDCl_3$.

Acq. Data Name: WFJ-Cmp14a_21
Creation Parameters: Average(MS Time:0.69..0.71)

Experiment Date/Time: 11/1/2021 3:13:44 PM
Ionization Mode: FD+(eiFi)

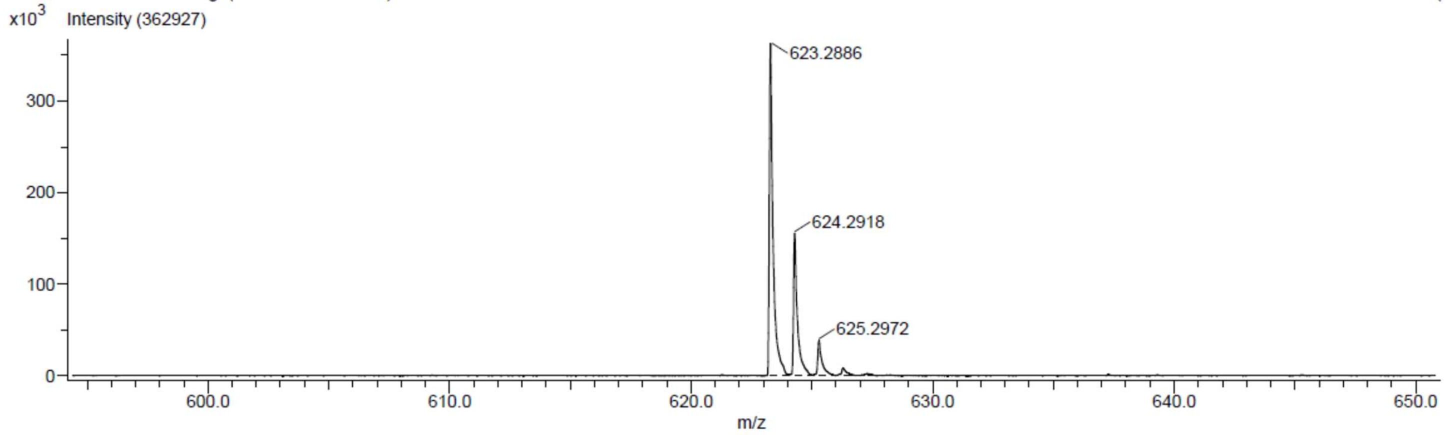

Formula: C38H41N1O7
Mono Isotopic Mass: 623.2883012

Addition/Desorption Ion: None
Charge Number: 1

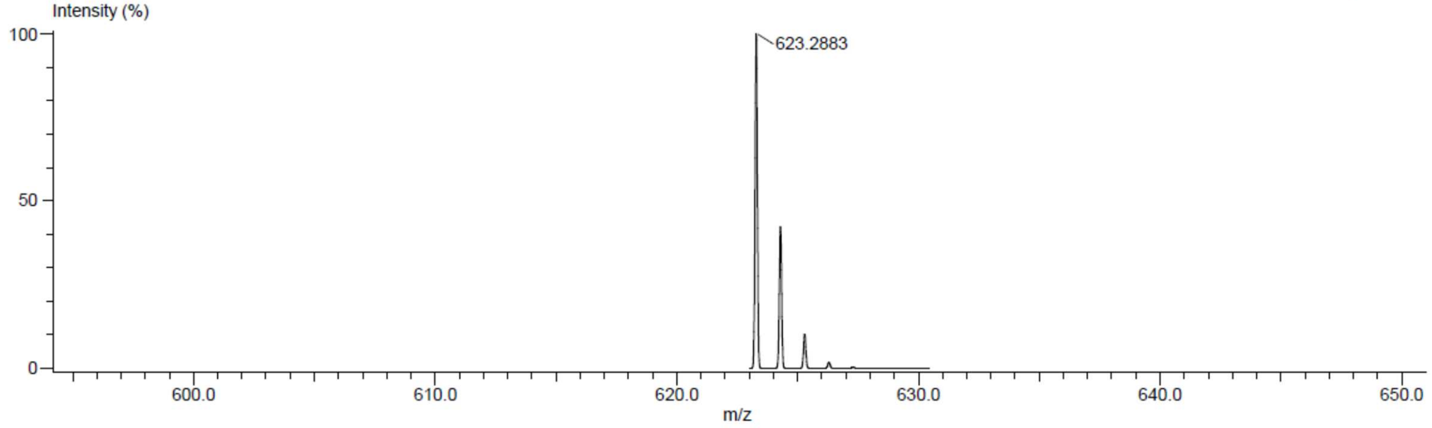

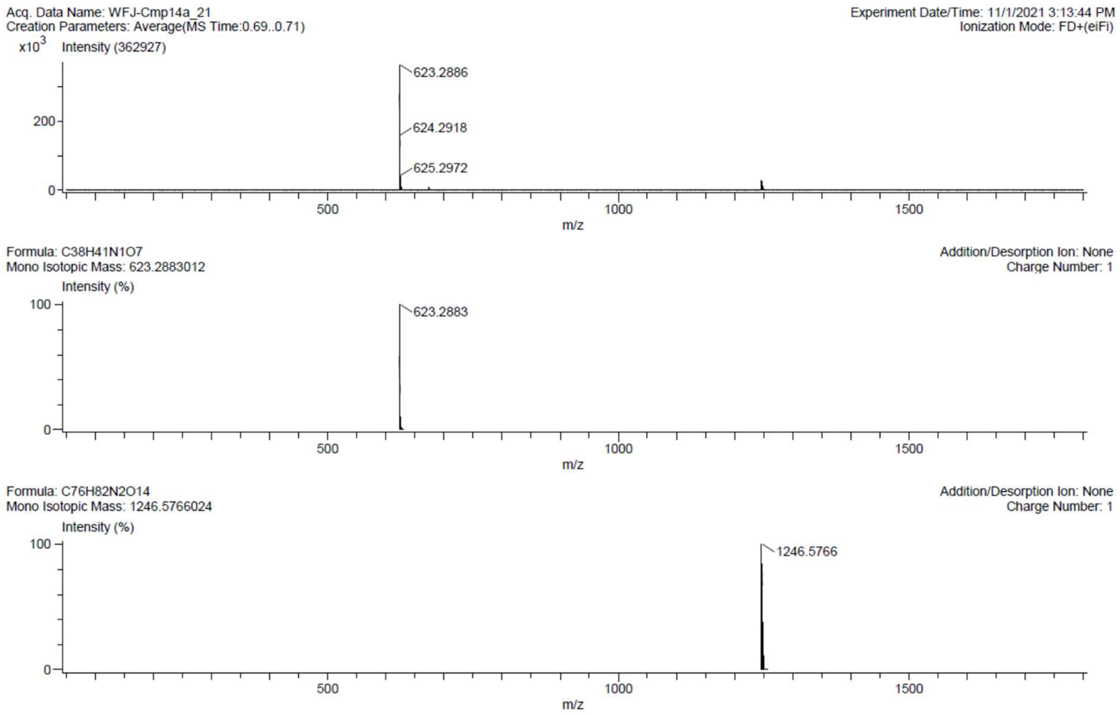

Figure S53. Mass spectrum of **14a**. Calculated masses are: **14a**; 623.2833.

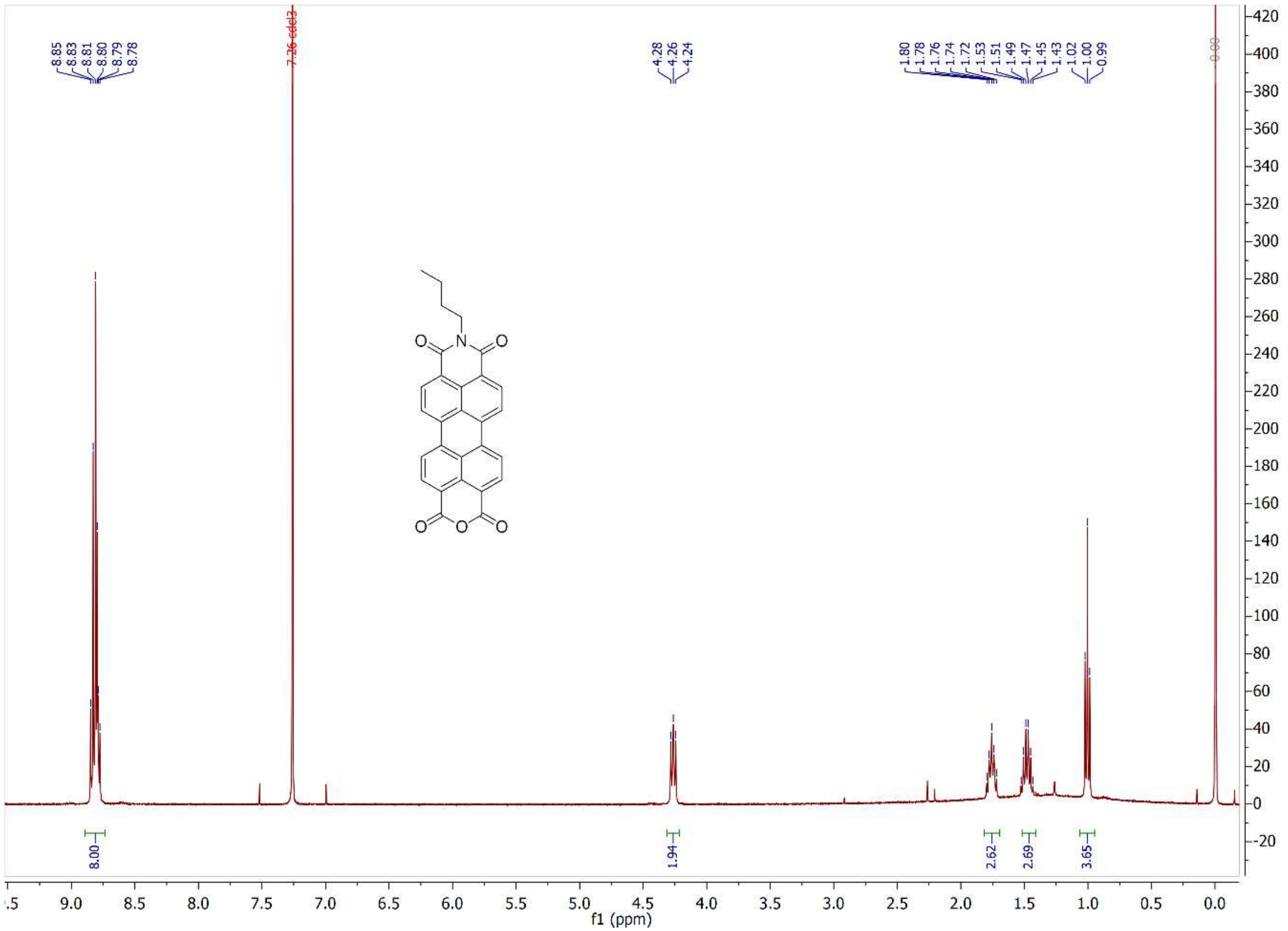
8.85
8.83
8.81
8.80
8.79
8.78
7.26 cdcl3
4.28
4.26
4.24
1.80
1.78
1.76
1.74
1.72
1.53
1.51
1.49
1.47
1.45
1.43
1.02
1.00
0.99
0.00
8.00
1.94
2.62
2.69
3.65
f1 (ppm)

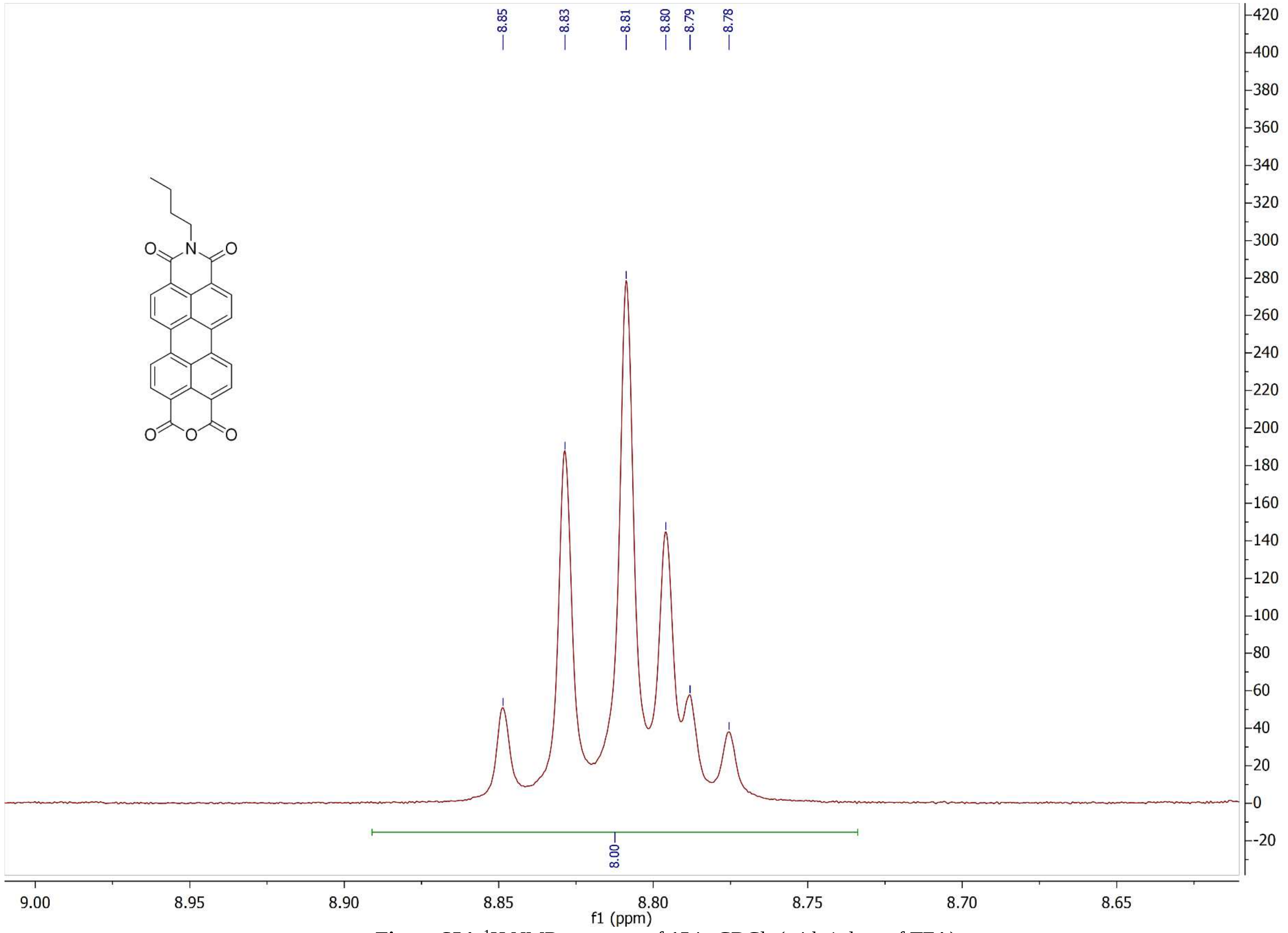

Figure S54. ^{1}H NMR spectrum of **15** in $CDCl_3$ (with 1 drop of TFA)
